# Total Synthesis of Mycoplanecin A

**DOI:** 10.1021/acs.orglett.5c02803

**Published:** 2025-08-01

**Authors:** Emanuel Papadopoulos, Lukas Junk, Uli Kazmaier

**Affiliations:** † Saarland University, Organic Chemistry I, Campus, Building C4.2, D-66123 Saarbrücken, Germany; ‡ Helmholtz Institute for Pharmaceutical Research Saarland (HIPS), Saarland University Campus, C8.1, 66123 Saarbrücken, Germany

## Abstract

The first total synthesis of mycoplanecin A, a potent antitubercular macrocyclic depsipeptide natural product targeting the DnaN sliding clamp, is described. Interesting key steps are the synthesis of the two *trans*-4-alkylated-l-prolines via an iterative Matteson homologation and an O→N acyl shift observed during the fragment coupling of the building blocks. The challenging macrocyclization of the globally deprotected linear precursor was accomplished under optimized high-temperature, high-dilution conditions. This work provides chemical access to mycoplanecin A, enabling further biological investigation and analogue development against the important pathogen *Mycobacterium tuberculosis*.

## Graphical abstract

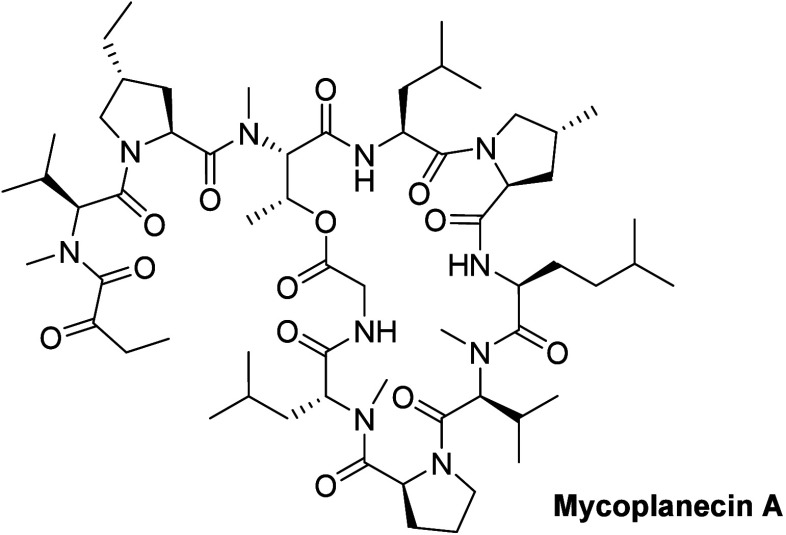

Tuberculosis (TB), caused by the pathogen *Mycobacterium tuberculosis* (*Mtb*), remains a significant global health challenge, responsible for millions of illnesses and deaths annually.^1^ The emergence and spread of multidrug-resistant and extensively drug-resistant strains severely complicate treatment efforts, creating an urgent need for novel antibiotics with distinct mechanisms of action to overcome existing resistance patterns.^2^ Natural products have historically served as a vital source of antimicrobial agents.^3^ Peptides, in particular, represent a promising class of therapeutics due to their potential for high target specificity and potency. For example, cyclomarins^4^ (Figure 1) and ilamycins^5^ are structurally related marine cyclopeptides targeting the important mycobacterial protease-associated unfoldase ClpC1,^6^ causing cell death by uncontrolled proteolytic activity of this enzyme.^7^ Based on this, BacPROTACs^8^ and Homo-BacPROTACs were developed, which ultimately initiate its proteolytic degradation by binding to ClpC1.^9^

**Figure 1:**
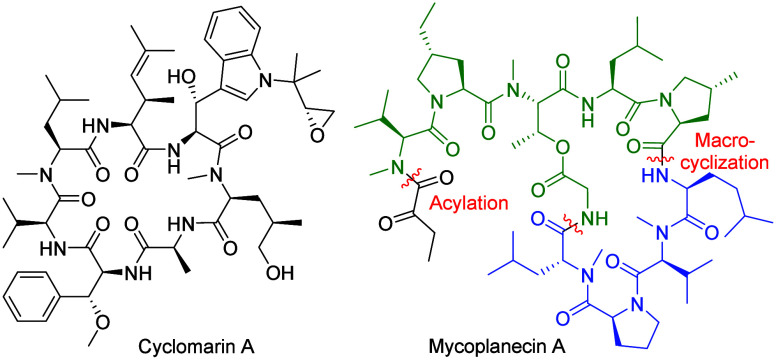
Natural products with anti tuberculosis activity.

Another interesting peptidic natural product exhibiting potent antituberculotic activity is mycoplanecin A, first isolated in 1983 from the fermentation broth of *Actinoplanes awajinensis* subsp. *mycoplanecinus*.^10^ This cyclic decadepsipeptide contains four *N*-methylated amino acids and, notably, the unusual nonproteinogenic amino acids *trans*-4-methyl- and *trans*-4-ethyl-l-proline.^11^

Despite its early discovery, significant renewed interest in mycoplanecin A has emerged by recent investigations.^12^ These studies confirmed its remarkable potency against *Mtb* with a minimum inhibitory concentration (MIC) significantly lower than that of the related griselimycins.^13^ Furthermore, this work validated the DNA polymerase III sliding clamp (DnaN), a crucial component of the bacterial replisome, as the molecular target for both mycoplanecins and griselimycins, highlighting a valuable mechanism distinct from many currently used TB drugs and suggesting potential efficacy against resistant strains. To enable further biological evaluation and modifications in the context of SAR studies, synthetic access to mycoplanecins would be required, which, interestingly, has not yet been described.

Our research group has been working on the synthesis of biologically active natural products for years, and in addition to the anti-TB natural products cyclomarin^14^ and ilamycin,^15^ we therefore also wanted to devote ourselves to the synthesis of mycoplanecins. Our plan was to synthesize mycoplanecin A by coupling two fragments (western and eastern part) between the glycine and the *N*-methylated d-leucine and to close the ring between the 4-methylproline and the homoleucine (HoLeu). The choice of cyclization site is analogous to that successfully utilized in the total synthesis of the structurally related griselimycin.^13^ The *N*-terminal α-ketobutyric acid should be introduced toward the end of the synthetic sequence to provide easy access to derivatives for SAR studies by varying the acylating reagents.

To obtain the western fragment, two unusual proline building blocks were required, each alkylated at position 4. While 4-methylproline is quite common in natural products,^16^ 4-ethylproline is virtually nonexistent, and no feasible synthesis is known for the *trans*-derivative required here. Therefore, we decided to develop a synthesis that allows the generation of differently substituted 4-*trans*-substituted prolines in order to provide access to differently substituted mycoplanecin derivatives. We chose the Matteson homologation, which allows the stereoselective introduction of different substituents into a continuously growing alkyl chain.^17^ It is therefore increasingly used in natural product synthesis^18^ and can also be used for the synthesis of amino acids.^19^

The synthesis of the desired *trans*-4-alkylproline derivatives **12** and **13** commenced with boronate **1**,^20^ employing a series of Matteson homologations as outlined in Scheme 1. The initial sequence involved introducing the required alkyl side chains to furnish intermediate boronates **2** and **3** in good yield. Their further chain extension was accomplished via a second homologation, where the generated α-chloroboronate was reduced with superhydride to introduce a methylene group. Next, the nitrogen moiety was subsequently introduced by converting **4** and **5** to their corresponding α-chloroboronates followed by displacement with NaN_3_ in DMF to generate the α-azido boronates **6** and **7**. A final homologation step was performed to install a bromine atom alpha to the boron center, yielding the corresponding α-bromo-β-azido boronic esters. It should be mentioned that the analogous homologation toward the corresponding α-chloro boronate resulted in significant lower yields due to incomplete conversion. These α-bromo-β-azido boronates obtained were subjected to a Pinnick-type oxidation to afford the corresponding α-azidocarboxylic acids. Subsequent esterification as *tert*-butyl esters^21^ provided **8** and **9** in good yields over three steps. Subsequent acidic detritylation^22^ and tosylation furnished tosylates **10** and **11**. Finally, hydrogenation of the azide moiety over Pd/C effected reduction to the amine, which underwent spontaneous intramolecular cyclization to construct the proline ring scaffold.^23^ The resulting secondary amine was directly *N*-protected to afford the desired proline derivates **12** and **13** in 26% and 21% overall yield over ten steps, starting from bromomethylboronic acid pinacol ester

**Scheme 1:**
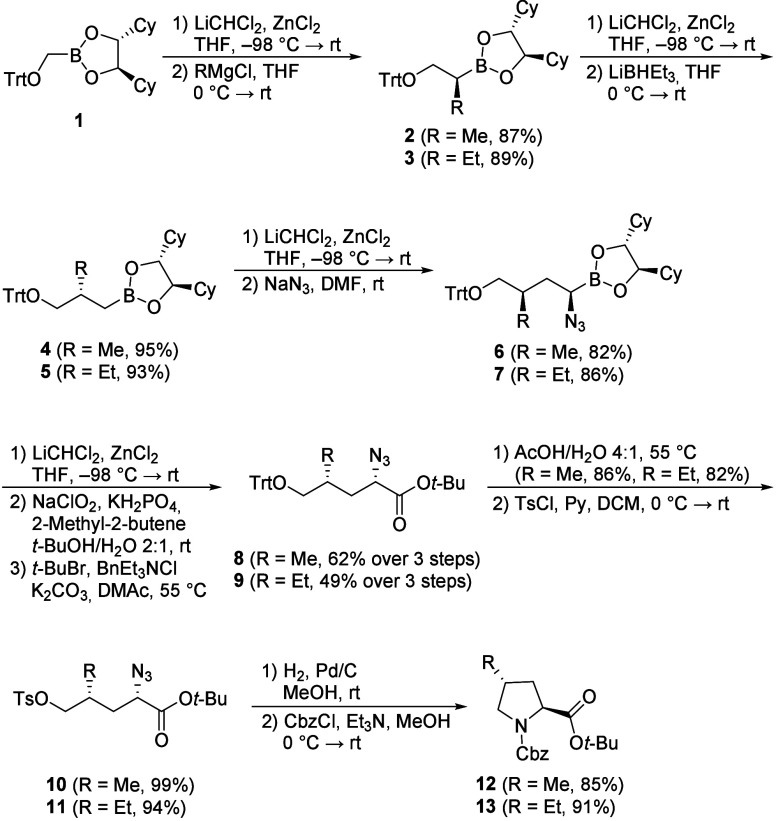
Synthesis of Protected *trans*-4-Alkyl Prolines

For the synthesis of the hexapeptide fragment, **12** was subjected to hydrogenation and the resulting amine was coupled with Cbz-Leu-OH using EDC/HOBt to afford dipeptide **14** (Scheme 2). In order to prevent potential diketopiperazine formation, the following Cbz deprotection was conducted in the presence of 1 eq. HCl. Coupling of the dipeptide hydrochloride with Cbz-(OTBS)-MeThr-OH (**S2**), mediated by isobutyl chloroformate (IBCF), provided tripeptide **15** in excellent yield. Removal of the silyl ether with TBAF unfortunately led to the formation of the corresponding oxazolidinone, whereas deprotection using catalytic acetyl chloride in MeOH^24^ yielded the desired alcohol in excellent yield. This alcohol was subsequently esterified with *C*-terminal deprotected ethyl proline **13’** (obtained by treating **13** with a mixture 1:3 of TFA in CHCl_3_) using 2-methyl-6-nitrobenzoic anhydride (MNBA) and 4-pyrrolidinylpyridine (PPY) to furnish the depsipeptide **16** without detectable epimerization (Alternative conditions, such as DIC/DMAP or EDC/PPY, led to incomplete conversion, although starting material was recoverable). Next, simultaneous removal of both Cbz protecting groups via hydrogenation induced a concomitant O→N acyl shift. This key step regenerated the MeThr secondary alcohol and formed the peptide bond corresponding to the previous ester linkage. Chain elongation by coupling the obtained free amine with Alloc-MeVal-OH (**S4**) using PyAOP gave pentapeptide **17** in good yield. Finally, esterification of the MeThr side-chain hydroxyl group in **17** with Fmoc-Gly-OH, again employing MNBA/PPY conditions, completed the synthesis of the target hexapeptide fragment **18** in overall 65% yield from **12**.

**Scheme 2:**
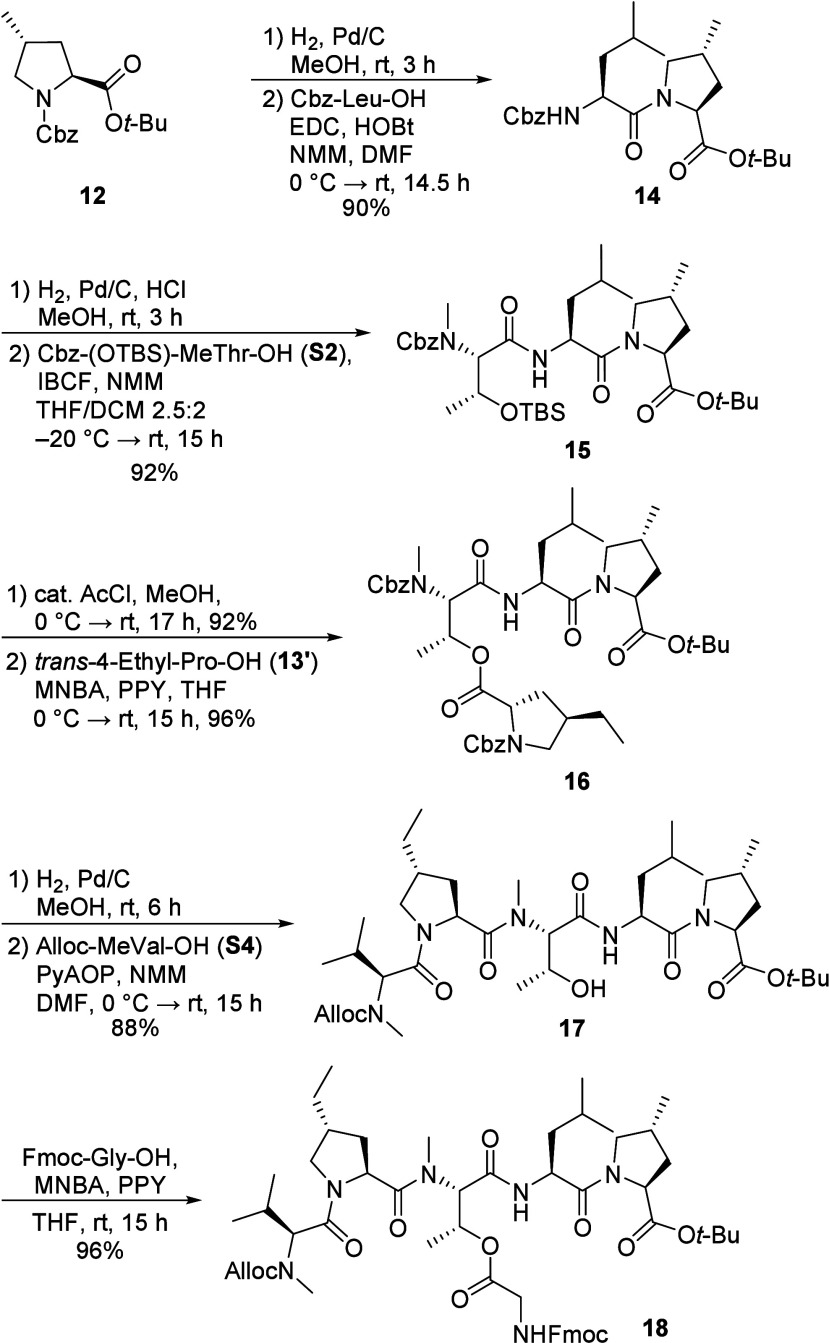
Synthesis of Hexapeptide Fragment **18**

The synthesis of tetrapeptide fragment **22** started from Boc-d-MeLeu-OMe (**19**, Scheme 3). Removal of the Boc protecting group was followed by coupling of the resulting amine with Boc-Pro-OH, mediated by an acyl *N*-methylimidazolium cation,^25^ to afford dipeptide **20**. Subsequent Boc deprotection and coupling of the amine formed with Boc-MeVal-OH using TBTU provided tripeptide **21**. Following a final acidic cleavage of the Boc group, the resulting amine was coupled with Boc-HoLeu-OH employing HATU as the coupling reagent, yielding protected tetrapeptide **22’**. (Notably, alternative conditions for this final coupling, such as using BEP or the acyl *N*-methylimidazolium cation mediated transformation, furnished the product in significantly lower yields.) Lastly, saponification of the methyl ester in **22’** generated the *C*-terminally free acid **22** (43% yield from **19**), which was used in the subsequent fragment coupling.

**Scheme 3:**
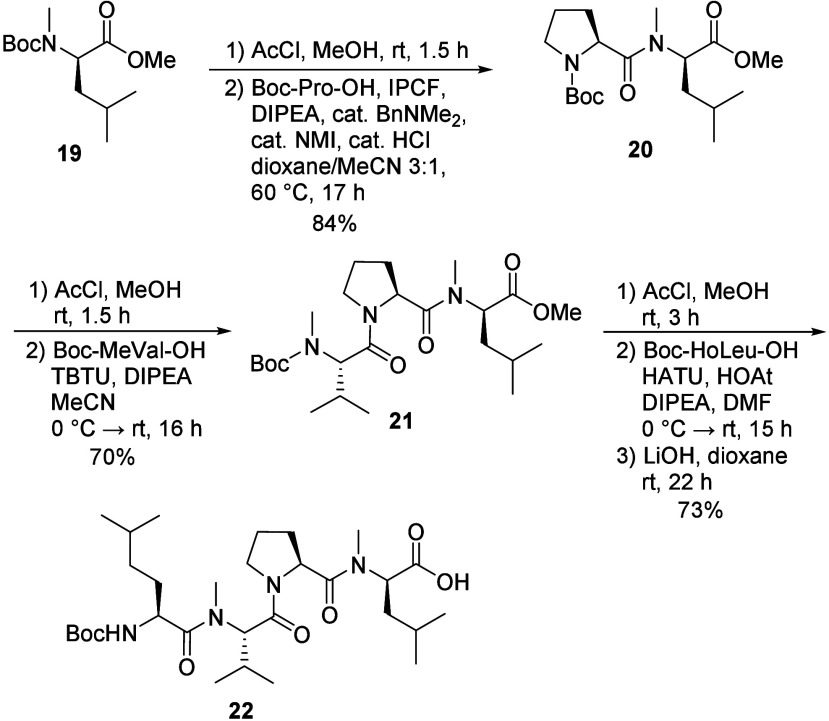
Synthesis of Tetrapeptide Fragment **22**

With peptide fragments **18** and **22** in hand, the final stages commenced with their convergent coupling (Scheme 4). First, the Fmoc protecting group of **18** was cleaved using tris­(2-aminoethyl)­amine (tren).^26^ Initial attempts to couple the resulting free amine with carboxylic acid **22** using HATU or propylphosphonic anhydride (T3P) provided the desired linear decapeptide **23** only in moderate yields. However, employing COMU as the coupling agent successfully furnished **23** in a much improved yield of 81%. Next, global deprotection of **23** was affected in a 1:1 mixture of TFA in DCM, cleaving simultaneously the *N*-terminal Boc group and the *C*-terminal *tert*-butyl ester.

**Scheme 4:**
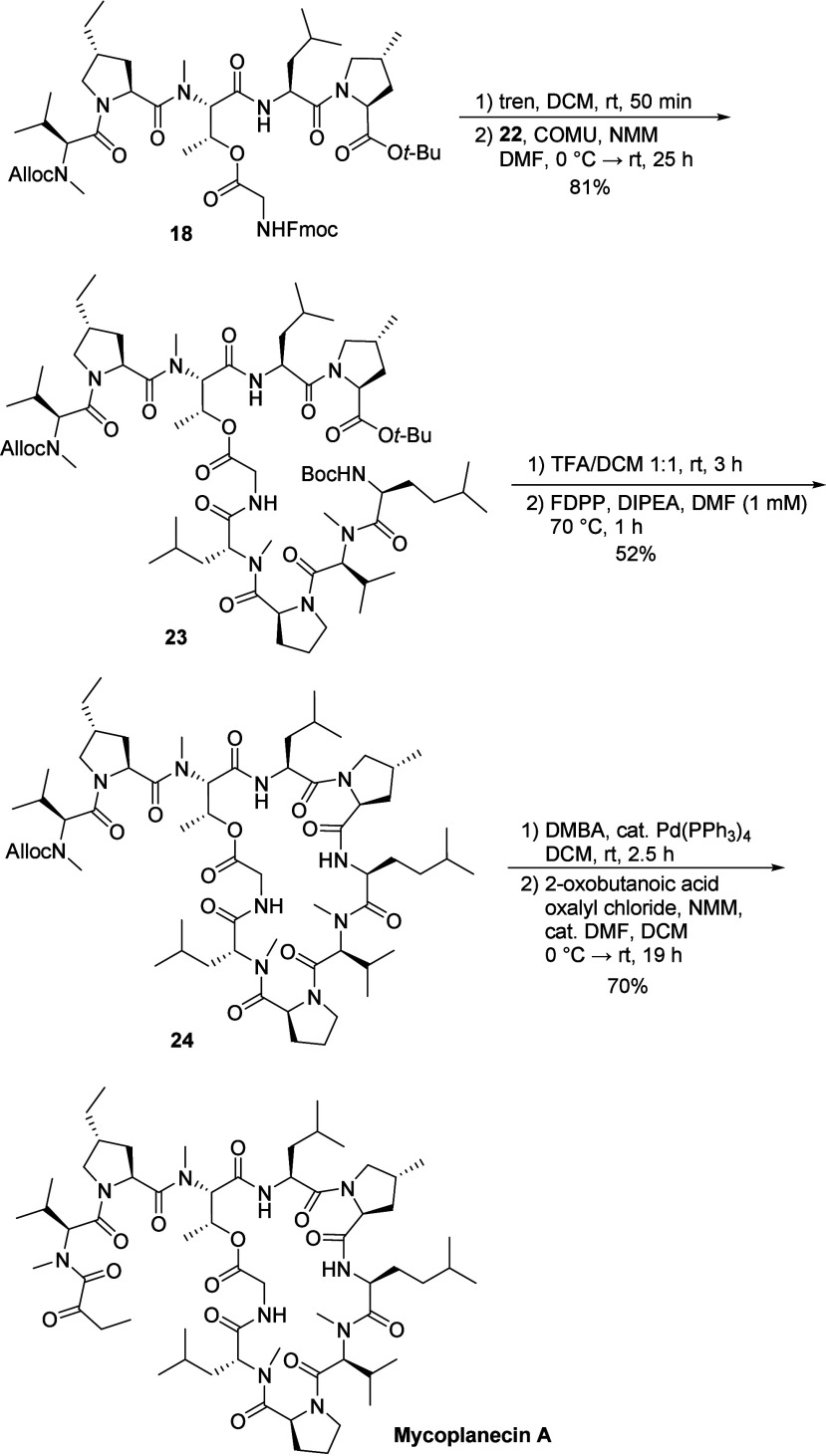
Fragment Coupling, Macrocyclization and Acylation to Mycoplanecin A

The resulting deprotected peptide was then subjected to macrocyclization under high-dilution conditions, facilitated by syringe pump addition of deprotected **23** toward the coupling reagents. Coupling agents such as PyAOP and COMU delivered **24** only in 22% yield while TFFH failed to produce any desired product. Optimization of the COMU conditions by increasing the equivalents from 4.4 to 10 eq. improved the yield modestly to 39%. A more effective strategy involved performing the cyclization at elevated temperature (70 °C). Although, this drastically reduced the yield with COMU (to 9%), it significantly improved the yield with PyAOP and FDPP (pentafluorophenyl diphenyl-phosphinate), which was finally used for cyclizations in a 1 mM scale.

However, attempts to scale up the cyclization resulted in a decreased yield of 52%. It was observed that conducting the reaction in smaller batches and adding the linear peptide solution slowly along the inner wall of the flask was beneficial for larger scale preparations. The penultimate step involved removal of the Alloc protecting group from the *N*-methylated valine residue in **24**. This was achieved using Pd­(PPh_3_)_4_ and dimethyl barbituric acid (DMBA) acting as an allyl scavenger.^27^ Finally, the liberated amine was acylated with 2-oxobutanoic acid by treatment with the corresponding acid chloride, to furnish mycoplanecin A in good yield.

In conclusion, we have accomplished the first reported total synthesis of mycoplanecin A utilizing a convergent, solution-phase approach. The synthesis was achieved in 18 steps for the longest linear sequence with an overall yield of 5.2%. A key feature of this work was the synthesis of the requisite unusual amino acids, *trans*-4-methyl-l-proline (**12**) and *trans*-4-ethyl-l-proline (**13**), via a sequence of Matteson homologations. The convergent strategy employed, particularly the final acylation step, provides a platform for (late-stage) derivatization. Such efforts toward analogue synthesis and subsequent biological evaluation are currently underway.

## Supplementary Material



## Data Availability

The data underlying this study are available in the published article and its Supporting Information.

## Notes

Detailed experimental procedures, NMR data and copies of ^1^H and ^13^C NMR spectra. (PDF)
