# Supplementary material for: Total Synthesis of Mycoplanecin A
Source: Org Lett. 2025 Aug 1;27(32):9061–5. doi: 10.1021/acs.orglett.5c02803 (PMC12362602; doi:10.1021/acs.orglett.5c02803)
Supplement: Supplementary file 1 [file ol5c02803_si_001.pdf]

Total Synthesis of Mycoplanecin A

Emanuel Papadopoulos, Lukas Junk, Uli Kazmaier*

Saarland University, Institute for Organic Chemistry, Building C4.2

P. O. Box 151150, 66041 Saarbruecken, Germany

Email: u.kazmaier@mx.uni-saarland.de

Supporting Information

List of Abbreviations 2

General Information 4

General Procedures (GP) 4

Synthesis of the Amino Acid Building Blocks 6

Synthesis of the 4-Alkylprolines[3] 6

Synthesis of the other Amino Acid Building Blocks 19

Synthesis of the Peptide Fragments 24

Synthesis of the Hexapeptide Fragment 24

Synthesis of the Tetrapeptide Fragment 30

Synthesis of Mycoplanecin A: Fragment Coupling, Cyclization & Acylation 33

NMR Spectra 39

Bibliography 128

List of Abbreviations

Ac	Acetyl
Bn	Benzyl
Boc	*tert*-Butyloxycarbonyl
Cbz	Benzyloxycarbonyl
cHex	Cyclohexane
CI	Chemical ionization
COMU	(1-Cyano-2-ethoxy-2-oxoethylidenaminooxy)dimethylamino-morpholino-carbenium-hexafluorophosphate
Cy	Cyclohexyl
CyPro	*trans*-4-Cyclohexylproline
DICHED	1,2-Dicyclohexyl-1,2-ethandiol
DIPA	Diisopropylamine
DIPEA	*N,N*-Diisopropylethylamine
DMAc	Dimethylacetamide
DMBA	Dimethylbarbituric acid
DMF	Dimethylformamide
DMSO	Dimethylsulfoxide
EDC	1-(3-Dimethylaminopropyl)-3-ethylcarbodiimide hydrochloride
ESI	Electrospray ionization
Et	Ethyl
EtOPro	*trans*-4-Ethoxyproline
EtPro	*trans*-4-Ethylproline
FDPP	Pentafluorphenyl diphenylphosphinate
Fmoc	Fluorenylmethoxycarbonyl
Gly	Glycine
HATU	*O*-(7-Azabenzotriazol-1-yl)-*N,N,N‘N‘*-tetramethyluronium hexafluorphosphate
HOAt	1-Hydroxy-7-azabenzotriazole
HOBt	1-Hydroxybenzotriazole
HoLeu	Homoleucine
HPLC	high performance liquid chromatography
HRMS	high resolution mass spectrometry
IBCF	*iso*-Butylchlorformiate
IPCF	*iso*-Propylchloroformiate
LC	liquid chromatography
LDA	Lithiumdiisopropylamide
Leu	Leucine
Lit.	Literature value
Lys	Lysine
Me	Methyl
MeOPro	*trans*-4-Methoxyproline
MePro	*trans*-4-Methylproline
MNBA	2-Methyl-6-nitrobenzoic anhydride
MS	mass spectrometry
n-Bu	*n*-Butyl
NMI	*N*-Methylimidazole
NMM	*N*-Methylmorpholine
NMR	nuclear magnetic resonance
Ph	Phenyl
PPY	4-Pyrrolidinylpyridine

Pro	Proline
PyAOP	(7-Azabenzotriazol-1-yloxy)tripyrrolidino-phosphonium hexafluorophosphate
R_f	Retention factor
sat.	saturated
TBS	*tert*-Butyldimethylsilyl
TBTU	*O*-(Benzotriazol-1-yl)-*N,N,N',N'*-tetramethyl-uronium tetrafluoroborate
t-Bu	*tert*-Butyl
Tf	Triflyl
TFA	Trifluoroacetic acid
THF	Tetrahydrofuran
Thr	Threonine
TLC	Thin layer chromatography
tren	Tris(2-aminoethyl)amine
Trt	Trityl
Ts	Tosyl
Val	Valine

General Information

All air and moisture sensitive reactions were carried out in dried glassware (> 100 °C) under N_2 or Ar atmosphere. Anhydrous solvents were purchased from Acros Organics or dried before use (THF was distilled over sodium/benzophenone) and stored under nitrogen atmosphere. The products were purified by column chromatography on silica gel columns (Machery-Nagel 60, 0.063−0.2 mm) or a Grace *Reveleris PREP Chromatography* system or a Büchi *Pure C-815 Flash* system using prepacked columns *RediSep® Rf* from *Teledyne Isco*. For reverse-phase chromatography (indicated by C_{18}-SiO_2), a Grace *Reveleris PREP Chromatography* system was used with *Büchi FlashPure Select C18* columns and MeCN/H_2O solvents. Analytical TLC was performed on pre-coated silica gel plates (Machery-Nagel, Polygram Sil G/UV_{254}). Detection was accomplished with UV light (254 nm), $KMnO_4$ solution, ninhydrin solution or cerium(IV)/ ammonium molybdate solution. Melting points were determined with a MEL-TEMP II (Laboratory devices) apparatus and are uncorrected. ^{1}H and ^{13}C NMR spectra were recorded at 293 or 298 K on a Bruker Avance II 400 MHz spectrometer [^{1}H 400 MHz and ^{13}C 100 MHz], a Bruker Avance I 500 MHz spectrometer [^{1}H 500 MHz and ^{13}C 126 MHz] or a Bruker AV 500 Neo spectrometer [^{1}H 500 MHz and ^{13}C 126 MHz]. Chemical shifts (δ) are reported in parts per million (ppm) relative to TMS or internal solvent signal. Peaks were assigned using (^{1}H, ^{1}H)-COSY, (^{1}H,^{13}C)-HSQC and (^{1}H,^{13}C)-HMBC spectra. LC/MS measurements were performed on a Shimadzu system (system controller: SCL-10A, liquid chromatograph: LC-2030C ED Plus, autosampler: SCL-6B, mass spectrometer: LCMS-2020), using an Onyx $C_{18(2)}$ column (50×4.6 mm, 3 µm particle size) from Phenomenex as the stationary phase. Detection was performed using a diode array detector (190−300 nm) and a mass detector (Shimadzu LCMS-2020) via ESI. Mass spectra were recorded with a Finnigan MAT 95 spectrometer (quadrupole) (CI), a Bruker Daltonics maXis 4G (ESI) and a UHPLC Quadrupole Orbitrap (Q Exactive) mass spectrometer from Thermo Scientific (ESI). Optical rotations were measured with a Jasco P-2000 polarimeter or a Krüss P8000-T80 polarimeter in a thermostated (20 °C ± 1 °C) cuvette, using a sodium vapor lamp (λ = 589 nm) as radiation source. $[\alpha]_D^{20}$ values are given in 10^{-1}deg cm^2 g^{-1}. (*R*,*R*)-DICHED was prepared according to known literature protocols.[1,2]

Safety Note on Azides: Azide compounds are potentially explosive and should be handled with extreme care. They can be sensitive to heat, shock, friction, and static discharge. All reactions should be performed on a small scale behind a blast shield in a well-ventilated fume hood. Care must be taken to avoid the formation of highly explosive heavy metal azides.

Safety Note on Cooling Baths: To reach the target temperature of −98 °C in **GP1** (or −82 °C for compound **8** and **9)**, liquid nitrogen was carefully added to a Dewar flask containing MeOH (or EtOAc for compound **8** and **9**) until it was partially frozen. The combination of a flammable organic solvent with liquid nitrogen in an open cooling bath can lead to condensation of liquid oxygen over time, which increases the risk of explosions. Alternatively, an immersion cryo cooler can be employed in order to avoid the risk of explosions.

General Procedures (GP)

GP1: Matteson Homologation

Under N_2 atmosphere, anhydrous DCM (3.0 eq.) was added to anhydrous THF (1.5 mL/mmol *n*-BuLi) and cooled to −98 °C (MeOH/$N_{2(l)}$). Subsequently, *n*-BuLi (1.1–1.3 eq., 2.5 M in *n*-hexane) was added slowly along the inner wall of the flask and rinsed with anhydrous THF. After 20 min, a solution of the boronic acid ester (1.0 eq.) in anhydrous THF (1.4 mL/mmol boronic acid ester) was slowly added dropwise at −98 °C, rinsing twice with anhydrous THF. After another 20 min, a suspension of in a high vacuum oven-dried $ZnCl_2$ (3.0 eq.) in anhydrous THF (0.6 mL/mmol $ZnCl_2$) was added, followed by warming to rt. The conversion to the α-chloroboronic ester was monitored via ^{1}H-NMR spectroscopy (usually 2–3 h).

<u>Variant a): One-pot reaction of the α-chloroboronic ester:</u> The reaction mixture was cooled to the indicated temperature and the corresponding nucleophile solution was added dropwise. After complete conversion was observed via ^{1}H-NMR spectroscopy, the mixture was quenched with sat. $NH_4Cl_{(aq.)}$ solution and diluted with *n*-pentane. Next, the phases were then separated, and the aqueous phase was extracted once with *n*-pentane. The combined organic phases were dried over $MgSO_4$, the solvent was removed in vacuo, and the resulting residue was purified by column chromatography.

<u>Variant b): Reaction of the isolated α-chloroboronic ester:</u> The reaction mixture was quenched with sat. NH_4Cl solution and diluted with *n*-pentane. Afterwards, the phases were then separated, and the aqueous phase was extracted once with *n*-pentane. The combined organic phases were dried over $MgSO_4$, and the solvent was removed in vacuo. Then the resulting residue was dissolved in anhydrous THF under N_2 atmosphere and cooled to the indicated temperature, whereupon the nucleophile solution was added dropwise. After complete conversion was observed via ^{1}H-NMR spectroscopy, the mixture was quenched with sat. $NH_4Cl_{(aq.)}$ solution and diluted with *n*-pentane. The phases were then separated, and the aqueous phase was extracted once with *n*-pentane. Lastly, the combined organic phases were dried over $MgSO_4$, the solvent was removed in vacuo, and the resulting residue was purified by column chromatography.

GP2: Azide reduction and Cbz cleavage with Pd/C under H_2 atmosphere

In a round-bottom flask, the corresponding azide or *N*-Cbz-protected amino acid/peptide was dissolved in MeOH and Pd/C (10 wt%) was added. The reaction vessel was evacuated six times with a water aspirator vacuum pump until the black suspension began to boil, before the vessel was then flushed with H_2 (balloon). The reaction mixture was stirred under H_2 atmosphere (balloon pressure) until complete conversion was observed via TLC or LC/MS. Subsequently, it was filtered through Celite, rinsed with MeOH and the solvent was removed in vacuo.

GP3: Boc-Cleavage of *N*-Boc-Protected Amino Acid Esters and Peptides

In a round-bottom flask, the *N*-Boc-protected amine (1.0 eq.) was dissolved in MeOH and cooled to 0 °C. Acetyl chloride (7.5–10 eq.) was added dropwise, and the mixture was subsequently warmed to rt. Alternatively, 1,4-dioxane with the addition of MeOH (7.5–10 eq.) or a 4.0 M solution of HCl in 1,4-dioxane (10 eq.) was used as the reaction medium instead of MeOH. After complete conversion was confirmed by TLC or LC/MS (1–6 h), the solvent was removed in vacuo, and the residue was dried under high vacuum.

GP4: Peptide Coupling with TBTU/HATU/HOBt+EDC/PyAOP/COMU

The carboxylic acid (1.0–2.5 eq.) and amine component (1.0 eq.) were dissolved in the indicated solvent under N_2 or Ar atmosphere and cooled to 0 °C. Subsequently, HOBt/HOAt (1.0–1.4 eq., if necessary), the coupling reagent (1.1–2.5 eq.), and the tertiary amine base (2.2–4.0 eq.) were added sequentially, before the reaction mixture was slowly warmed to rt overnight.

Afterwards, the mixture was diluted with EtOAc and washed with 1 M $KHSO_{4(aq.)}$, followed by water, sat. $NaHCO_{3(aq.)}$ solution and sat. $NaCl_{(aq.)}$ solution, before the organic phase was dried over $MgSO_4$ and the solvent was removed in vacuo. If DMF was used as the solvent, the organic phase was washed with 1 M $LiCl_{(aq.)}$ instead of water. Finally, the resulting residue was purified by column chromatography.

Synthesis of the Amino Acid Building Blocks

Synthesis of the 4-Alkylprolines[3]

(4*R*,5*R*)-4,5-Dicyclohexyl-2-[(trityloxy)methyl]-1,3,2-dioxaborolane [1][4]

Under N_2 atmosphere, a solution of 49.5 g (190 mmol, 1.05 eq.) of triphenylmethanol in 360 mL anhydrous DMSO was treated portionwise with 8.72 g (60 wt% in mineral oil, 223 mmol, 1.2 eq.) of NaH. After stirring the reaction mixture for 16 h, 40.0 g (181 mmol, 1.0 eq.) of bromomethylboronic acid pinacol ester were added via a transfer cannula at 0 °C, warming to rt after complete addition. After 3 d, 380 mL of sat. $NH_4Cl_{(aq.)}$ solution was added to the reaction mixture (exothermic) and it was extracted twice with 250 mL of Et_2O. The combined organic phases were washed with water and the solvent was removed in vacuo. Next, the resulting residue was dissolved in 500 mL of Et_2O, treated with 500 mL of 1 M $NaOH_{(aq.)}$, followed by 61.6 g (452 mmol, 2.5 eq.) of pentaerythritol, and then stirred vigorously. After 21 h, the phases were separated, and the aqueous phase was carefully neutralized with 1 M $HCl_{(aq.)}$ at 0 °C (pH = 6). The precipitated white solid was filtered off, washed with water, dried under high vacuum, and then suspended in 380 mL of *n*-pentane and treated with 24.3 g (107 mmol, 0.7 eq.) of (*R*,*R*)-DICHED (1,2-dicyclohexyl-1,2-ethandiol), followed by 37.7 g (306 mmol, 2.0 eq.) of anhydrous $MgSO_4$. After 4 h, an additional 3.46 g (15.3 mmol, 0.1 eq.) and after 16.5 h another 3.45 g (15.3 mmol, 0.1 eq.) of (*R*,*R*)-DICHED were added to the mixture. After complete conversion was observed via TLC (25.5 h), the suspension was filtered, and the filter cake was rinsed with Et_2O. The filtrate was freed from

the solvent in vacuo and the residue was purified by column chromatography (*n*-pentane/Et_2O 9:1). In total, 66.6 g (131 mmol, 86%) of the boronic ester **1** were isolated as a colorless, crystalline solid.

$[\alpha]_D^{20}$ = +53.9 (c = 1.0, $CHCl_3$); Lit.: $[\alpha]_D^{20}$ = +49.7 (c = 1.0, $CHCl_3$)[5]

Melting range: 113–116 °C; Lit.: 108–110 °C[5] **R$_f$** = 0.55 (*n*-Pentane/EtOAc 95:5)

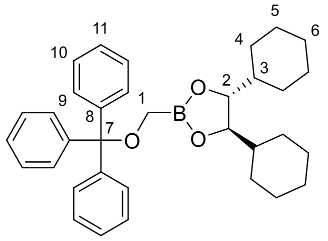

^{1}H-NMR (500 MHz, $CDCl_3$): δ = 7.49 (m, 6 H, 9-H), 7.29 (m, 6 H, 10-H), 7.22 (m, 3 H, 11-H), 3.95 (m, 2 H, 2-H), 2.95 (d, $^2J_{1a,1b}$ = 15.7 Hz, 1 H, 1-H$_a$), 2.86 (d, $^2J_{1a,1b}$ = 15.7 Hz, 1 H, 1-H$_b$), 1.75–1.86 (m, 6 H, 4-H‴, 5-H″, 5-H‴),1.64 (m, 2 H, 4-H″), 1.70 (m, 2 H, 6-H′),1.38 (m, 2 H, 3-H), 1.15–1.32 (m, 6 H, 5-H, 5-H′, 6-H), 1.05 (m, 4 H, 4-H, 4-H′).

^{13}C-NMR (126 MHz, $CDCl_3$): δ = 144.3 (s, C-8), 129.0 (d, C-9), 127.8 (d, C-10), 126.9 (d, C-11), 87.8 (s, C-7), 83.9 (d, C-2), 50.6 (t, C-1), 43.1 (d, C-3), 28.5 (t, C-4′),27.5 (t, C-4), 26.6 (t, C-6), 26.2 (t, C-5′), 26.0 (t, C-5).

HRMS (CI): Calculated for $C_{34}H_{41}BO_3^+$ $[M]^+$: 508.3143, found: 508.3147.

(4*R*,5*R*)-4,5-Dicyclohexyl-2-[(*S*)-1-(trityloxy)propan-2-yl]-1,3,2-dioxaborolane [2]

According to **GP1a**, 10.0 g (19.67 mmol, 1.0 eq.) of boronic acid ester **1** were reacted with 3.8 mL (59.06 mmol, 3.0 eq., ρ = 1.320 g/mL) anhydrous DCM, 9.0 mL (2.5 M in *n*-hexane, 22.50 mmol, 1.1 eq.) *n*-BuLi, and 8.06 g (59.14 mmol, 3.0 eq.) $ZnCl_2$. After 2.5 h, the mixture was cooled to 0 °C and 16.4 mL (3.0 M in THF, 49.20 mmol, 2.5 eq.) MeMgCl were slowly added dropwise. After the addition was complete, the mixture was slowly warmed to rt. After 1.5 days, the mixture was cooled again to 0 °C, and another 3.2 mL (3.0 M in THF, 9.600 mmol, 0.5 eq.) of MeMgCl were slowly added dropwise before slowly warming to rt. After 4 h, the mixture was worked up according to **GP1a**. Automated column chromatography (SiO_2, *n*-Pentane/Et_2O 10:0 ⟶ 8:2) yielded 9.19 g (17.14 mmol, 87%) of the boronic acid ester **2** as a colorless resin.

$[\alpha]_D^{20}$ = +42.2 (c = 1.0, $CHCl_3$); Lit.: $[\alpha]_D^{20}$ = +36.4 (c = 1.0, $CHCl_3$)[5]

R$_f$ = 0.55 (*n*-Pentane/Et_2O 9:1)

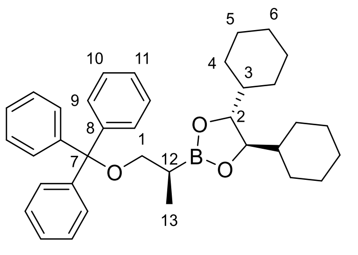

^{1}H-NMR (400 MHz, $CDCl_3$): δ = 7.45 (d, $^3J_{9,10}$ = 7.3 Hz, 6 H, 9-H), 7.26 (m, 6 H, 10-H), 7.20 (m, 3 H, 11-H), 3.84 (m, 2 H, 2-H), 3.17 (dd, $^2J_{1a,1b}$ = 8.4 Hz, $^3J_{1a,12}$ = 6.5 Hz, 1 H, 1-H_a), 3.03 (t, $^2J_{1b,1a}$ ≈ $^3J_{1b,12}$ = 7.6 Hz, 1 H, 1-H_b), 1.67–1.78 (m, 6 H, 4-H‴, 5-H″, 5-H‴),1.55–1.67 (m, 4 H, 4-H″, 6-H′), 1.46 (m, 1 H, 12-H), 1.28 (m, 2 H, 3-H), 1.10–1.22 (m, 6 H, 5-H, 5-H′, 6-H), 1.00 (d, $^3J_{13,12}$ = 7.4 Hz, 3 H, 13-H), 0.97–1.09 (m, 2 H, 4-H″), 0.92 (m, 2 H, 4-H).

^{13}C-NMR (101 MHz, $CDCl_3$): δ = 144.6 (s, C-8), 128.8 (d, C-9), 127.6 (d, C-10), 126.7 (d, C-11), 86.2 (s, C-7), 83.3 (d, C-2), 66.7 (t, C-1), 43.0 (d, C-3), 28.3 (t, C-4′), 27.4 (t, C-4), 26.5 (t, C-6), 26.1 (t, C-5′), 25.9 (t, C-5), 18.3 (d, C-12), 13.0 (q, C-13).

HRMS (CI): Calculated for $C_{36}H_{45}BO_3^+$ $[M]^+$: 536.3456, found: 536.3459.

(4*R*,5*R*)-4,5-Dicyclohexyl-2-[(*S*)-1-(trityloxy)butan-2-yl]-1,3,2-dioxaborolane [3]

According to **GP1a**, 10.0 g (19.67 mmol, 1.0 eq.) of boronic acid ester **1** were reacted with 3.8 mL (59.06 mmol, 3.0 eq., ρ = 1.320 g/mL) anhydrous DCM, 9.0 mL (2.5 M in *n*-hexane, 22.50 mmol, 1.1 eq.) *n*-BuLi and 8.04 g (59.01 mmol, 3.0 eq.) $ZnCl_2$. After 3 h, the reaction was cooled to 0 °C and 18.2 mL (2.7 M in THF, 49.14 mmol, 2.5 eq.) EtMgCl was slowly added dropwise. After the addition was completed, the reaction was warmed to rt and cooled back to 0 °C after 4.5 d to slowly add another 5.0 mL (2.0 M in THF, 10.00 mmol, 0.5 eq.) EtMgCl dropwise before warming to rt. After 2 d, the reaction was worked up accordingly. Column chromatographic purification (SiO_2, *n*-pentane/Et_2O 9:1) afforded 9.60 g (17.44 mmol, 89%) of the boronic acid ester **3** as a colorless, waxy solid.

$[\alpha]_D^{20}$ = +44.7 (c = 1.0, $CHCl_3$) **R_f** = 0.37 (*n*-Pentane/EtOAc 95:5)

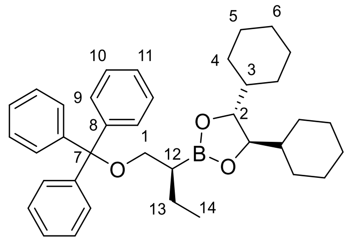

^{1}H-NMR (500 MHz, $CDCl_3$): δ = 7.45 (m, 6 H, 9-H), 7.26 (m, 6 H, 10-H), 7.20 (m, 3 H, 11-H), 3.85 (m, 2 H, 2-H), 3.23 (d, $^2J_{1a,1b}$ = 8.3 Hz, $^3J_{1a,12}$ = 6.3 Hz, 1 H, 1-H_a), 3.03 (dd, $^2J_{1b,1a}$ = 8.3 Hz $^3J_{1b,12}$ = 7.3 Hz, 1 H, 1-H_b), 1.67–1.81 (m, 6 H, 4-H‴, 5-H″, 5-H‴), 1.56–1.67 (m, 4 H, 4-H″, 6-H′), 1.48 (m, 2 H, 13-H), 1.25–1.41 (m, 3 H, 3-H, 12-H), 1.09–1.21 (m, 6 H, 5-H, 5-H′, 6-H), 0.90–1.09 (m, 4 H, 4-H, 4-H′), 0.83 (t, $^3J_{14,13}$ = 7.3 Hz, 3 H, 14-H).

^{13}C-NMR (101 MHz, $CDCl_3$): δ = 144.7 (s, C-8), 128.9 (d, C-9), 127.7 (d, C-10), 126.8 (d, C-11), 86.3 (s, C-7), 83.6 (d, C-2), 64.9 (t, C-1), 43.2 (d, C-3), 28.6 (t, C-4'), 27.7 (t, C-4), 26.6 (t, C-6), 26.2 (t, C-5'), 26.0 (t, C-5), 21.2 (t, C-13), 13.8 (q, C-14).

The signal of C-12 lies beneath the noise of the spectrum.

HRMS (CI): Calculated for $C_{37}H_{48}BO_3^+$ $[M+H]^+$: 551.3691, found: 551.3676.

(4*R*,5*R*)-4,5-Dicyclohexyl-2-[(*R*)-2-methyl-3-(trityloxy)propyl]-1,3,2-dioxaborolane [4]

According to **GP1b**, 8.51 g (15.87 mmol, 1.0 eq.) of boronic acid ester **2** were reacted with 3.0 mL (46.63 mmol, 2.9 eq., ρ = 1.320 g/mL) anhydrous DCM, 7.5 mL (2.5 M in *n*-hexane, 18.75 mmol, 1.2 eq.) *n*-BuLi and 6.60 g (48.40 mmol, 3.1 eq.) $ZnCl_2$. After 3.5 h, aqueous work-up was carried out accordingly and the residue obtained was reacted according to **GP1b** in 40 mL anhydrous THF with 19.0 mL (1.0 M in THF, 19.00 mmol, 1.2 eq.) Superhydride® at 0 °C, adding after 1.5 h another 1.6 mL (1.0 M in THF, 1.60 mmol, 0.1 eq.) Superhydride® at 0 °C. After a further 1.5 h of stirring at rt, the reaction was worked up accordingly. Column chromatographic purification (SiO_2, *n*-pentane/Et_2O 95:5) yielded 8.27 g (15.02 mmol, 95%) of the boronic acid ester **4** as a colorless solid.

$[\alpha]_D^{20}$ = +20.4 (c = 1.0, $CHCl_3$) **Melting range**: 82–85 °C

$\mathbf{R_f}$ = 0.54 (*n*-Pentane/Et_2O 9:1)

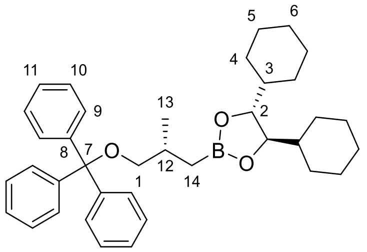

¹H-NMR (500 MHz, $CDCl_3$): δ = 7.44 (d, $^3J_{9,10}$ = 7.1 Hz, 6 H, 9-H), 7.27 (t, $^3J_{10,11}$ = 7.3 Hz, 6 H, 10-H), 7.21 (t, $^3J_{11,10}$ = 7.2 Hz, 3 H, 11-H), 3.77 (m, 2 H, 2-H), 2.91 (dd, $^2J_{1a,1b}$ = 8.5 Hz, $^3J_{1a,12}$ = 6.1 Hz, 1 H, 1-H_a), 2.81 (dd, $^2J_{1b,1a}$ = 8.5 Hz, $^3J_{1b,12}$ = 7.0 Hz, 1 H, 1-H_b), 1.69–1.78 (m, 6 H, 4-H‴, 5-H″, 5-H‴), 2.08 (m, 1 H, 12-H), 1.66 (m, 2 H, 6-H′), 1.55 (m, 2 H, 4-H″), 1.08–1.34 (m, 8 H, 3-H, 5-H, 5-H′, 6-H′), 0.98 (d, $^3J_{13,12}$ = 6.5 Hz, 3 H, 13-H), 0.79–1.06 (m, 5 H, 4-H, 4-H′, 14-H_a), 0.68 (dd, $^2J_{14b,14a}$ = 15.7 Hz, $^3J_{14b,12}$ = 8.7 Hz, 1 H, 14-H_b).

¹³C-NMR (101 MHz, $CDCl_3$): δ = 144.7 (s, C-8), 129.0 (d, C-9), 127.7 (d, C-10), 126.8 (d, C-11), 86.2 (s, C-7), 83.4 (d, C-2), 70.4 (t, C-1), 43.1 (d, C-3), 30.6 (d, C-12), 28.6 (t, C-4′), 27.6 (t, C-4), 26.6 (t, C-6), 26.1 (t, C-5′), 26.0 (t, C-5), 20.0 (q, C-13), 16.0 (t, C-14).

HRMS (ESI): Calculated for $C_{37}H_{47}BNaO_3^+$ $[M+Na]^+$: 573.3510, found: 573.3511.

(4*R*,5*R*)-4,5-Dicyclohexyl-2-[(*R*)-2-methyl-3-(trityloxy)propyl]-1,3,2-dioxaborolane [5]

According to **GP1b**, 9.59 g (17.41 mmol, 1.0 eq.) boronic acid ester **3** were reacted with 3.4 mL (52.84 mmol, 3.0 eq., ρ = 1.320 g/mL) anhydrous DCM, 8.5 mL (2.5 M in *n*-hexane, 21.25 mmol, 1.2 eq.) *n*-BuLi and 7.25 g (53.17 mmol, 3.1 eq.) $ZnCl_2$. After 4 h was worked up accordingly and the residue obtained was reacted according to **GP1b** in 44 mL anhydrous THF with 21.0 mL (1.0 M in THF, 21.00 mmol, 1.2 eq.) Superhydride® at 0 °C. After 2.5 h the reaction was worked up accordingly. Column chromatographic purification (SiO_2, *n*-pentane/Et_2O 9:1) afforded 9.15 g (16.21 mmol, 93%) of the boronic acid ester **5** as a colorless, opaque resin.

$[\alpha]_D^{20}$ = +15.3 (c = 1.0, $CHCl_3$) $\mathbf{R_f}$ = 0.41 (*n*-Pentane/EtOAc 95:5)

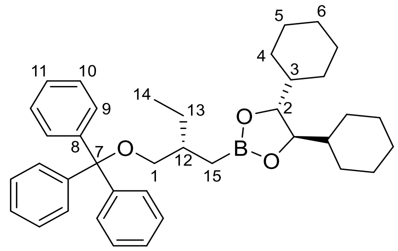

^{1}H-NMR (500 MHz, $CDCl_3$): δ = 7.44 (m, 6 H, 9-H), 7.27 (m, 6 H, 10-H), 7.21 (m, 3 H, 11-H), 3.74 (m, 2 H, 2-H), 2.94 (dd, $^2J_{1a,1b}$ = 8.7 Hz, $^3J_{1a,12}$ = 5.8 Hz, 1 H, 1-H_a), 2.90 (dd, $^2J_{1b,1a}$ = 8.7 Hz, $^3J_{1b,12}$ = 6.5 Hz, 1 H, 1-H_b), 1.91 (sept, $^3J_{12,1}$ ≈ $^3J_{12,13}$ ≈ $^3J_{12,15}$ = 6.4 Hz, 1 H, 12-H), 1.68–1.80 (m, 6 H, 4-H‴, 5-H″, 5-H‴), 1.66 (m, 2 H, 6-H′), 1.48–1.58 (m, 3 H, 4-H″,13-H′), 1.30 (m, 1 H, 13-H), 1.05–1.25 (m, 8 H, 3-H, 5-H, 5-H′, 6-H), 0.94 (m, 4 H, 4-H, 4-H′), 0.85 (m, 2 H, 15-H), 0.77 (t, $^3J_{14,13}$ = 7.4 Hz, 3 H, 14-H).

^{13}C-NMR (126 MHz, $CDCl_3$): δ = 144.7 (s, C-8), 129.0 (d, C-9), 127.7 (d, C-10), 126.8 (d, C-11), 86.2 (s, C-7), 83.5 (d, C-2), 67.9 (t, C-1), 43.1 (d, C-3), 36.9 (d, C-12), 28.6 (t, C-4'), 27.7 (t, C-4), 26.6 (2 t, C-6, C-13), 26.1 (t, C-5'), 26.0 (t, C-5), 11.3 (q, C-14).

The signal of C-15 lies beneath the noise of the spectrum.

HRMS (ESI): Calculated for $C_{38}H_{49}BNaO_3^+$ $[M+Na]^+$: 587.3667, found: 587.3660.

(4*R*,5*R*)-2-[(1*R*,3*R*)-1-Azido-3-methyl-4-(trityloxy)butyl]-4,5-dicyclohexyl-1,3,2-dioxaborolane [6]

According to **GP1b**, 8.25 g (14.99 mmol, 1.0 eq.) of boronic acid ester **4** were reacted with 3.0 mL (46.63 mmol, 3.1 eq., ρ = 1.320 g/mL) anhydrous DCM, 7.0 mL (2.5 M in *n*-hexane, 17.50 mmol, 1.2 eq.) *n*-BuLi and 6.23 g (45.72 mmol, 3.1 eq.) $ZnCl_2$. After 2.5 h, the reaction was worked up aqueously. The residue obtained was dissolved in 74 mL of anhydrous DMF under N_2 atmosphere, to which 5.36 g (82.49 mmol, 5.5 eq.) NaN_3 were added and stirred for 19.5 h.

Contrary to **GP1b**, the yellow suspension was then quenched with sat. $NH_4Cl_{(aq.)}$ solution and diluted with *n*-pentane and a little H_2O until two clear phases were obtained. After the phases were separated, the aqueous phase was extracted once with *n*-pentane and the combined organic phases were washed once with 1 M $LiCl_{(aq.)}$ before drying over $MgSO_4$. The solvent was then removed in vacuo and the residue was purified by column chromatography[1] (SiO_2, *n*-pentane/Et_2O 9:1). A total of 7.44 g (12.29 mmol, 82%) of the α-azidoboronic acid ester **6** could be isolated as a colorless, opaque resin.

$[\alpha]_D^{20}$ = +16.0 (c = 1.0, $CHCl_3$) **R_f** = 0.35 (*n*-Pentane/EtOAc 95:5)

[1] It should be noted, that the α-azidoboronic acid ester are not stable to SiO_2, thus conducting the chromatographic quickly is advised. The boronic acid esters can also be used without purification.

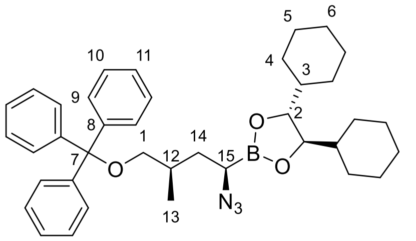

^{1}H-NMR (500 MHz, $CDCl_3$): δ = 7.44 (m, 6 H, 9-H), 7.28 (m, 6 H, 10-H), 7.23 (m, 3 H, 11-H), 3.93 (m, 2 H, 2-H), 2.90–3.05 (m, 3 H, 1-H, 15-H), 1.98 (m, 1 H, 12-H), 1.87 (m, 1 H, 14-H_a), 1.71–1.81 (m, 6 H, 4-H‴, 5-H″, 5-H‴), 1.67 (m, 2 H, 6-H′), 1.57 (m, 2 H, 4-H″), 1.49 (m, 1 H, 14-H_b), 1.32 (m, 2 H, 3-H), 1.13–1.29 (m, 6 H, 5-H, 5-H′, 6-H), 1.02 (d, $^{3}J_{13,12}$ = 6.7 Hz, 3 H, 13-H), 0.92–1.12 (m, 4 H, 4-H, 4-H′).

^{13}C-NMR (101 MHz, $CDCl_3$): δ = 144.4 (s, C-8), 128.9 (d, C-9), 127.8 (d, C-10), 127.0 (d, C-11), 86.3 (s, C-7), 84.3 (d, C-2), 67.2 (t, C-1), 43.0 (d, C-3), 34.7 (t, C-14), 31.6 (d, C-12), 28.4 (t, C-4′),27.4 (t, C-4), 26.5 (t, C-6), 26.1 (t, C-5′),25.9 (t, C-5), 18.2 (q, C-13).

The signal of C-15 lies beneath the noise of the spectrum.

HRMS (CI): Calculated for $C_{38}H_{50}BNO_3^+$ $[M+2H-N_2]^+$: 579.3878, found: 579.3850.

(4*R*,5*R*)-2-[(1*R*,3*R*)-1-Azido-3-((trityloxy)methyl)pentyl]-4,5-dicyclohexyl-1,3,2-dioxaborolane [7]

According to **GP1b**, 9.14 g (16.20 mmol, 1.0 eq.) boronic acid ester **5** were reacted with 3.2 mL (49.74 mmol, 3.1 eq., ρ = 1.320 g/mL) anhydrous DCM, 8.0 mL (2.5 M in *n*-hexane, 20.00 mmol, 1.2 eq.) *n*-BuLi and 6.73 g (49.39 mmol, 3.1 eq.) $ZnCl_2$. After 2.5 h, the reaction was worked up aqueously. The residue obtained was dissolved in 80 mL anhydrous DMF under N_2 atmosphere, to which 5.79 g (89.07 mmol, 5.5 eq.) NaN_3 was added and stirred for 16 h.

Contrary to **GP1b**, the yellow-orange suspension was quenched with sat. $NH_4Cl_{(aq.)}$ solution and diluted with *n*-pentane and a little water until two clear phases were obtained. After the phases were separated, the aqueous phase was extracted once with *n*-pentane and the combined organic phases were washed once with 1 M $LiCl_{(aq.)}$ before drying over $MgSO_4$. The solvent was then removed in vacuo and the residue was purified by column chromatography[2] (SiO_2, *n*-pentane/Et_2O 9:1). A total of 8.65 g (13.95 mmol, 86%) of the α-azido-boronic acid ester **7** was isolated as a pale-yellow, opaque resin.

$[\alpha]_D^{20}$ = +10.5 (c = 1.0, $CHCl_3$) **R_f** = 0.38 (*n*-Pentane/EtOAc 95:5)

[2] It should be noted, that the α-azidoboronic acid ester are not stable to SiO_2, thus conducting the chromatographic quickly is advised. The boronic acid esters can also be used without purification.

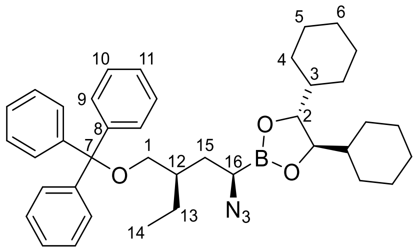

^{1}H-NMR (500 MHz, $CDCl_3$): δ = 7.44 (m, 6 H, 9-H), 7.29 (m, 6 H, 10-H), 7.23 (m, 3 H, 11-H), 3.93 (m, 2 H, 2-H), 3.08 (dd, $^2J_{1a,1b}$ = 9.2 Hz, $^3J_{1a,12}$ = 4.3 Hz, 1 H, 1-H_a), 3.02 (dd, $^2J_{1b,1a}$ = 9.2 Hz, $^3J_{1b,12}$ = 4.6 Hz, 1 H, 1-H_b), 2.90 (dd, $^3J_{16,15a/b}$ = 11.0 Hz, $^3J_{16,15b/a}$ = 4.3 Hz, 1 H, 16-H), 1.71–1.84 (m, 8 H, 4-H‴, 5-H″, 5-H‴, 12-H, 15-H_a), 1.66 (m, 2 H, 6-H′),1.53–1.63 (m, 4 H, 4-H″, 13-H_a, 15-H_b), 1.29–1.45 (m, 3 H, 3-H, 13-H_b), 1.15–1.28 (m, 6 H, 5-H, 5-H′, 6-H), 0.91–1.15 (m, 4 H, 4-H, 4-H′),0.81 (t, $^3J_{14,13}$ = 7.4 Hz, 3 H, 14-H).

^{13}C-NMR (126 MHz, $CDCl_3$): δ = 144.4 (s, C-8), 128.9 (d, C-9), 127.8 (d, C-10), 127.0 (d, C-11), 86.2 (s, C-7), 84.2 (d, C-2), 63.6 (t, C-1), 43.0 (d, C-3), 37.8 (d, C-12), 32.4 (t, C-15), 28.4 (t, C-4'), 27.4 (t, C-4), 26.5 (t, C-6), 26.1 (t, C-5'), 26.0 (t, C-5), 25.1 (t, C-13), 11.5 (q, C-14).

The signal of C-15 lies beneath the noise of the spectrum.

HRMS (ESI): Calculated for $C_{39}H_{50}BN_3NaO_3^+$ $[M+Na]^+$: 642.3837, found: 642.3837.

tert-Butyl (2*S*,4*R*)-2-Azido-4-methyl-5-(trityloxy)pentanoate [8]

Homologation: Under N_2 atmosphere, 2.6 mL (18.42 mmol, 1.5 eq., ρ = 0.717 g/mL) DIPA was dissolved in 5.0 mL anhydrous THF and cooled to −40 °C, before 6.5 mL (2.5 M in *n*-hexane, 16.25 mmol, 1.3 eq.) *n*-BuLi was added. Stirring was then continued for 10 min at −40 °C and then for 20 min at rt. The LDA solution thus prepared was slowly added by transfer cannula to a solution of 7.44 g (12.28 mmol, 1.0 eq.) boronic acid ester **6** and 2.6 mL (37.24 mmol, 3.0 eq., ρ = 2.490 g/mL) CH_2Br_2 in 17 mL anhydrous THF at −82 °C (EtOAc/$N_{2(l)}$). After stirring further at −82 °C for 1 h, a suspension of 5.12 g (37.58 mmol, 3.1 eq.) of $ZnCl_2$, heated beforehand under high vacuum, in 23 mL of anhydrous THF was added before subsequent slow warming to rt overnight. After 18 h, sat. $NH_4Cl_{(aq.)}$ solution was added to the reaction mixture and diluted with water and *n*-pentane. The phases were then separated, and the aqueous phase was extracted once with *n*-pentane before the combined organic phases were dried over $MgSO_4$ and the solvent was removed in vacuo.

Oxidation: The previously obtained α-bromoboronic acid ester was dissolved in 238 mL *t*-BuOH and mixed with 52 mL (491 mmol, 40 eq., ρ = 0.662 g/mL) 2-methyl-2-butene. A solution of 13.9 g (80 wt%, 123 mmol, 10 eq.) $NaClO_2$ and 16.8 g (123 mmol, 10 eq.) KH_2PO_4 in 118 mL water was then added dropwise. After 4 d, the yellow emulsion was concentrated in vacuo and acidified with 10 wt% aqueous citric acid solution to a pH of about 3 before extracting three times with Et_2O. The combined etheric phases were washed with sat. $Na_2S_2O_{3(aq.)}$ solution, dried over $MgSO_4$ and the solvent was removed in vacuo. The residue was then purified by automated column chromatography (SiO_2, *n*-pentane/EtOAc 10:0 ⟶ 7:3).

Esterification:[6] The previously isolated α-azidocarboxylic acid was dissolved in 122 mL DMAc and 56 mL (495 mmol, 40 eq., ρ = 1.210 g/mL) *t*-BuBr, 2.80 g (12.29 mmol, 1.0 eq.) $BnNEt_3Cl$ and 44.1 g (319 mmol, 26 eq.) K_2CO_3 were added. The suspension was then stirred for 5 h at 55 °C. After cooling to rt, water was added to the mixture until a clear solution was obtained. This was extracted three times with Et_2O, the combined etheric phases were washed twice with water and once with sat. $NaCl_{(aq.)}$ solution before drying over $MgSO_4$ and removing the solvent in vacuo. Automated column chromatography (SiO_2, cHex/EtOAc 10:0 ⟶ 8:2) afforded 3.60 g (7.63 mmol, 62%) of the α-azido-*tert*-butyl ester **8** as a colorless oil.

$[\alpha]_D^{20}$ = −1.6 (c = 1.0, $CHCl_3$) **R_f** = 0.69 (*n*-Pentane/EtOAc 1:1)

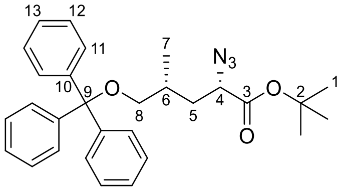

^{1}H-NMR (400 MHz, $CDCl_3$): δ = 7.44 (m, 6 H, 11-H). 7.29 (m, 6 H, 12-H), 7.23 (m, 3 H, 13-H), 3.57 (dd, $^3J_{4,5a}$ = 9.5 Hz, $^3J_{4,5b}$ = 5.1 Hz, 1 H, 4-H), 2.99 (m, 2 H, 8-H), 1.90–2.03 (m, 2 H, 5-H_b, 6-H), 1.55 (m, 1 H, 5-H_a), 1.48 (s, 9 H, 1-H), 1.04 (d, $^3J_{7,6}$ = 6.7 Hz, 3 H, 7-H).

^{13}C-NMR (126 MHz, $CDCl_3$): δ = 170.2 (s, C-3), 144.3 (s, C-10), 128.8 (d, C-11), 127.9 (d, C-12), 127.1 (d, C-13), 86.4 (s, C-9), 82.7 (s, C-2), 67.0 (t, C-8), 61.0 (d, C-4), 35.5 (t, C-5), 31.0 (d, C-6), 28.1 (q, C-1), 18.1 (q, C-7).

HRMS (ESI): Calculated for $C_{29}H_{33}N_3NaO_3^+$ $[M+Na]^+$: 494.2414, found: 494.2396.

tert-Butyl (2*S*,4*R*)-2-Azido-4-((trityloxy)methyl)hexanoate [9]

Homologation: Under N_2 atmosphere, 2.5 mL DIPA (17.71 mmol, 1.5 eq., ρ = 0.717 g/mL) were dissolved in 4.9 mL anhydrous THF and cooled to −40 °C before 6.5 mL (2.5 M in *n*-hexane, 16.25 mmol, 1.3 eq.) of *n*-BuLi was added. Stirring was then continued for 10 min at −40 °C and then for 20 min at rt. The LDA solution thus prepared was slowly added by transfer cannula to a solution of 7.37 g (11.90 mmol, 1.0 eq.) of boronic acid ester **7** and 2.5 mL (35.81 mmol, 3.0 eq., ρ = 2.490 g/mL) CH_2Br_2 in 17 mL anhydrous THF at −82 °C (EtOAc/$N_{2(l)}$). After stirring further at −82 °C for 1 h, a suspension of 4.96 g (36.39 mmol, 3.1 eq.) of $ZnCl_2$, heated under high vacuum beforehand, in 22 mL of anhydrous THF was added, followed by slow warming to rt overnight. After 16 h, sat. $NH4Cl_{(aq.)}$ was added to the reaction mixture and diluted with n-pentane and a little H_2O and. The phases were then separated, and the aqueous phase was extracted once with *n*-pentane before the combined organic phases were dried over $MgSO_4$ and the solvent was removed in vacuo.

Oxidation: The previously obtained α-bromoboronic acid ester was dissolved in 225 mL *t*-BuOH and mixed with 50 mL (472 mmol, 40 eq., ρ = 0.662 g/mL) 2-methyl-2-butene. A solution of 13.5 g (80 wt%, 120 mmol, 10 eq.) $NaClO_2$ and 16.2 g (120 mmol, 10 eq.) KH_2PO_4 in 114 mL water was then added dropwise. After 4 d, the yellow emulsion was concentrated in vacuo and acidified with 10 wt% aqueous citric acid solution to a pH of about 3 before

extracting three times with Et_2O. The combined etheric phases were washed with sat. $Na_2S_2O_{3(aq.)}$ solution, dried over $MgSO_4$ and the solvent was removed in vacuo. The residue was then purified by automated column chromatography (SiO_2, cHex/EtOAc 10:0 ⟶ 6:4).

Esterification:[6] The previously isolated α-azidocarboxylic acid was dissolved in 106 mL DMAc and 48 mL (424 mmol, 36 eq., ρ = 1.210 g/mL) *t*-BuBr, 2.40 g (10.55 mmol, 0.9 eq.) $BnNEt_3Cl$ and 37.9 g (274 mmol, 23 eq.) K_2CO_3 were added. The suspension was then stirred for 4.5 h at 55 °C. After cooling to rt, water was added to the mixture until a clear solution was obtained. This was extracted three times with Et_2O, the combined etheric phases were washed twice with water and once with sat. $NaCl_{(aq.)}$ solution before drying over $MgSO_4$ and removing the solvent in vacuo. Automated column chromatography (SiO_2, cHex/EtOAc 10:0 ⟶ 8:2) afforded 2.83 g (5.83 mmol, 49%) of the α-azido-*tert*-butyl ester **9** as a colorless oil.

$[\alpha]_D^{20}$ = −5.4 (c = 1.0, $CHCl_3$) **R_f** = 0.60 (*n*-Pentane/EtOAc 75:25)

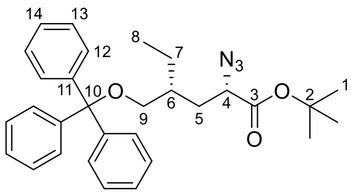

^{1}H-NMR (400 MHz, $CDCl_3$): δ = 7.45 (m, 6 H, 12-H). 7.30 (m, 6 H, 13-H), 7.24 (m, 3 H, 14-H), 3.51 (dd, $^3J_{4,5b}$ = 10.4 Hz, $^3J_{4,5a}$ = 4.1 Hz, 1 H, 4-H), 3.09 (dd, $^2J_{9a,9b}$ = 9.4 Hz, $^3J_{9a,6}$ = 4.1 Hz, 1 H, 9-H$_a$), 3.04 (dd, $^2J_{9b,9a}$ = 9.4 Hz, $^3J_{9b,6}$ = 4.1 Hz, 1 H, 9-H$_b$), 1.95 (ddd, $^2J_{5a,5b}$ = 14.6 Hz, $^3J_{5a,6}$ = 10.3 Hz, $^3J_{5a,4}$ = 3.8 Hz 1 H, 5-H$_a$), 1.61–1.71 (m, 2 H, 5-H$_b$, 6-H), 1.55 (m, 1 H, 7-H$_b$), 1.47 (s, 9 H, 1-H), 1.39 (m, 1 H, 7-H$_a$), 0.83 (t, $^3J_{8,7}$ = 7.4 Hz, 3 H, 8-H).

^{13}C-NMR (101 MHz, $CDCl_3$): δ = 170.4 (s, C-3), 144.2 (s, C-11), 128.9 (d, C-12), 127.9 (d, C-13), 127.1 (d, C-14), 86.4 (s, C-10), 82.7 (s, C-2), 63.8 (t, C-9), 60.8 (d, C-4), 37.3 (d, C-6), 33.3 (t, C-5), 28.2 (q, C-1), 25.2 (t, C-7), 11.6 (q, C-8).

HRMS (ESI): Calculated for $C_{30}H_{35}N_3NaO_3^+$ $[M+Na]^+$: 508.2571, found: 508.2566.

tert-Butyl (2*S*,4*R*)-2-Azido-5-hydroxy-4-methylpentanoate [10’][7]

3.59 g (7.61 mmol, 1.0 eq.) of the α-azido-*tert*-butyl ester **8** were dissolved in 30 mL AcOH, 7.5 mL water were added, and the mixture was heated in an oil bath to 55 °C. After 2 h, the reaction was cooled to rt and carefully neutralized (strong gas evolution) with sat. $NaHCO_{3(aq.)}$ solution. The aqueous phase was extracted three times with Et_2O, and the combined organic phases were dried over $MgSO_4$. The solvent was then removed in vacuo and the residue was purified by automated column chromatography (cHex/EtOAc 1:0 ⟶ 6:4). 1.50 g (6.54 mmol, 86%) of the alcohol **10’** was isolated as a pale-yellow liquid.

$[\alpha]_D^{20}$ = −12.0 (c = 1.0, $CHCl_3$) **R_f** = 0.30 (*n*-Pentane/EtOAc 1:1)

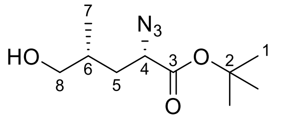

^{1}H-NMR (500 MHz, $CDCl_3$): δ =3.82 (dd, $^3J_{4,5b}$ = 8.6 Hz, $^3J_{4,5a}$ = 5.8 Hz, 1 H, 4-H). 3.55 (dd, $^2J_{8a,8b}$ = 10.8 Hz, $^3J_{8a,6}$ = 5.4 Hz, 1 H, 8-H_a), 3.51 (dd, $^2J_{8b,8a}$ = 10.8 Hz, $^3J_{8b,6}$ = 5.9 Hz, 1 H, 8-H_b), 1.92 (ddd, $^2J_{5a,5b}$ = 13.9 Hz, $^3J_{5a,6}$ = 7.2 Hz, $^3J_{5a,4}$ = 5.8 Hz, 1 H, 5-H_a), 1.83 (oct, $^3J_{6,5a} \approx {}^3J_{6,5b} \approx {}^3J_{6,7} \approx {}^3J_{6,8}$ = 6.4 Hz, 1 H, 6-H), 0.99 (d, $^3J_{7,6}$ = 6.8 Hz, 3 H, 7-H), 1.50 (s, 9 H, 1-H), 1.61 (ddd, $^2J_{5b,5a}$ = 14.5 Hz, $^3J_{5b,4}$ = 8.4 Hz, $^3J_{5b,6}$ = 6.3 Hz, 1 H, 5-H_b), 1.77 (bs, 1 H, OH).

^{13}C-NMR (126 MHz, $CDCl_3$): δ = 170.0 (s, C-3), 83.1 (s, C-2), 67.3 (t, C-8), 61.0 (d, C-4), 35.0 (t, C-5), 32.8 (d, C-6), 28.1 (q, C-1), 17.2 (q, C-7).

HRMS (CI): Calculated for $C_{10}H_{19}N_3NaO_3^+$ $[M+Na]^+$: 252.1319, found: 252.1318.

tert-Butyl (2*S*,4*R*)-2-Azido-4-(hydroxymethyl)hexanoate [11'][7]

2.77 g (5.70 mmol, 1.0 eq.) of the α-azido-*tert*-butyl ester **9** were dissolved in 23 mL AcOH, mixed with 5.8 mL H_2O and heated in an oil bath to 55 °C. After 3.5 h, the reaction was cooled to rt and carefully neutralized (strong gas evolution) with sat. $NaHCO_{3(aq.)}$ solution. The aqueous phase was extracted three times with Et_2O, and the combined organic phases were dried over $MgSO_4$. The solvent was then removed in vacuo and the residue was purified by automated column chromatography (cHex/EtOAc 1:0 ⟶ 6:4). 1.14 g (4.69 mmol, 82%) of the alcohol **11'** was isolated as a pale-yellow liquid.

$[\alpha]_D^{20}$ = −38.6 (c = 1.0, $CHCl_3$) **R_f** = 0.30 (*n*-Pentane/Et_2O 1:1)

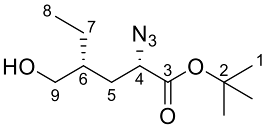

^{1}H-NMR (500 MHz, $CDCl_3$): δ = 3.86 (dd, $^3J_{4,5b}$ = 9.4 Hz, $^3J_{4,5a}$ = 4.9 Hz, 1 H, 4-H), 3.64 (dd, $^2J_{9a,9b}$ = 10.9 Hz, $^3J_{9a,6}$ = 4.6 Hz, 1 H, 9-H_a), 3.58 (dd, $^2J_{9b,9a}$ = 11.0 Hz, $^3J_{9b,6}$ = 5.5 Hz, 1 H, 9-H_b), 1.84 (ddd, $^2J_{5a,5b}$ = 13.7 Hz, $^3J_{5a,6}$ = 8.6 Hz, $^3J_{5a,4}$ = 4.9 Hz, 1 H, 5-H_a), 1.80 (bs, 1 H, OH), 1.72 (ddd, $^2J_{5b,5a}$ = 14.2 Hz, $^3J_{5b,4}$ = 9.4 Hz, $^3J_{5b,6}$ = 4.7 Hz, 1 H, 5-H_b), 1.60 (m, 1 H, 6-H), 1.49 (s, 9 H, 1-H), 1.44 (m, 1 H, 7-H_a), 1.33 (m, 1 H, 7-H_b), 0.93 (t, $^3J_{8,7}$ = 7.5 Hz, 3 H, 8-H).

^{13}C-NMR (126 MHz, $CDCl_3$): δ = 170.2 (s, C-3), 83.0 (s, C-2), 64.6 (t, C-9), 61.0 (d, C-4), 39.0 (d, C-6), 32.7 (t, C-5), 28.1 (q, C-1), 24.2 (t, C-7), 11.5 (q, C-8).

HRMS (CI): Calculated for $C_{11}H_{22}N_3O_3^+$ $[M+H]^+$: 244.1656, found: 244.1650.

tert-Butyl (2*S*,4*R*)-2-Azido-4-methyl-5-(tosyloxy)pentanoate [10]

To a solution of 1.50 g (6.52 mmol, 1.0 eq.) of alcohol **10'** in 32 mL DCM, 2.73 mL (19.57 mmol, 3.0 eq., ρ = 0.726 g/mL) Et_3N followed by 2.49 g (13.04 mmol, 2.0 eq.) tosyl chloride were added at 0 °C and slowly warmed to rt after the addition was complete. After 3 d, the reaction mixture was washed with sat. $NaHCO_{3(aq.)}$ solution and the aqueous phase was extracted three times with DCM before the combined organic phases were dried over $MgSO_4$. The solvent was then removed in vacuo and the residue was purified by column chromatography (*n*-pentane/Et2O 8:2). 2.48 g (6.47 mmol, 99%) of the tosylate **10** was isolated as a yellow oil.

$[\alpha]_D^{20}$ = −19.4 (c = 1.0, $CHCl_3$) **R$_f$** = 0.64 (*n*-Pentane/EtOAc 1:1)

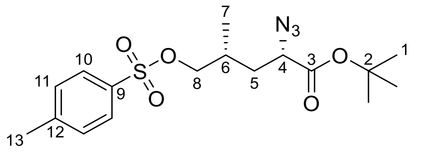

^{1}H-NMR (500 MHz, $CDCl_3$): δ = 7.79 (d, $^3J_{10,11}$ = 8.2 Hz, 2 H, 10-H). 7.36 (d, $^3J_{11,10}$ = 8.0 Hz, 2 H, 11-H), 3.90 (m, 2 H, 8-H), 3.64 (dd, $^3J_{4,5b}$ = 8.8 Hz, $^3J_{4,5a}$ = 5.8 Hz, 1 H, 4-H), 2.45 (s, 3 H, 13-H), 1.98 (oct, $^3J_{6,5a} \approx {}^3J_{6,5b} \approx {}^3J_{6,7} \approx {}^3J_{6,8}$= 6.4 Hz, 1 H, 6-H), 1.83 (ddd, $^3J_{5a,5b}$ = 13.2 Hz, $^3J_{5a,6}$ = 7.2 Hz, $^3J_{5a,4}$ = 6.5 Hz, 1 H, 5-H$_a$), 1.55 (ddd, $^3J_{5b,5a}$ = 14.5 Hz, $^3J_{5b,4}$ = 8.9 Hz, $^3J_{5b,6}$ = 6.6 Hz, 1 H, 5-H$_b$), 1.49 (s, 9 H, 1-H), 0.97 (d, $^3J_{7,6}$ = 6.8 Hz, 3 H, 7-H)

^{13}C-NMR (126 MHz, $CDCl_3$): δ = 169.4 (s, C-3), 145.1 (s, C-12), 132.9 (s, C-9), 130.0 (d, C-11), 128.1 (d, C-10), 83.3 (s, C-2), 73.7 (t, C-8), 60.4 (d, C-4), 34.2 (t, C-5), 30.1 (d, C-6), 28.1 (q, C-1), 21.8 (q, C-13), 17.0 (q, C-7).

HRMS (CI): Calculated for $C_{17}H_{26}N_3O_5S^+$ $[M+H]^+$: 384.1588, found: 384.1587.

tert-Butyl (2*S*,4*R*)-2-Azido-4-((tosyloxy)methyl)hexanoate [11]

To a solution of 1.13 g (4.65 mmol, 1.0 eq.) of the alcohol **11’** in 23 mL DCM, 1.95 mL (13.96 mmol, 3.0 eq., ρ = 0.726 g/mL) Et_3N, followed by 1.79 g (9.37 mmol, 2.0 eq.) tosyl chloride, were added at 0 °C and slowly warmed to rt after the addition was complete. After 3 d, the reaction mixture was washed with sat. $NaHCO_{3(aq.)}$ solution and the aqueous phase was extracted three times with DCM before the combined organic phases were dried over $MgSO_4$. The solvent was then removed in vacuo and the residue was purified by column chromatography (*n*-pentane/Et_2O 9:1 ⟶ 8:2). 1.74 g (4.37 mmol, 94%) of the tosylate **11** was isolated as a yellow oil.

$[\alpha]_D^{20}$ = −28.8 (c = 1.0, $CHCl_3$) **R$_f$** = 0.64 (*n*-Pentane/EtOAc 1:1)

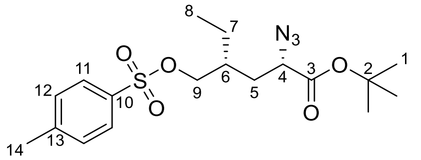

^{1}H-NMR (400 MHz, $CDCl_3$): δ = 7.79 (d, $^3J_{11,12}$ = 8.3 Hz, 2 H, 11-H), 7.35 (d, $^3J_{12,11}$ = 8.0 Hz, 2 H, 12-H), 3.97 (d, $^3J_{9,6}$ = 4.2 Hz, 2 H, 9-H), 3.63 (dd, $^3J_{4,5b}$ = 9.6 Hz, $^3J_{4,5a}$ = 4.5 Hz, 1 H, 4-H), 2.45 (s, 3 H, 14-H), 1.70−1.80 (m, 2 H, 5-H$_a$, 6-H), 1.65 (m, 1 H, 5-H$_b$), 1.49 (s, 9 H, 1-H), 1.41 (m, 1 H, 7-H$_a$), 1.34 (m, 1 H, 7-H$_b$), 0.83 (t, $^3J_{8,7}$ = 7.4 Hz, 3 H, 8-H).

^{13}C-NMR (101 MHz, $CDCl_3$): δ = 169.5 (s, C-3), 145.1 (s, C-13), 132.8 (s, C-10), 130.0 (d, C-12), 128.1 (d, C-11), 83.2 (s, C-2), 71.1 (t, C-9), 60.3 (d, C-4), 36.2 (d, C-6), 32.1 (t, C-5), 28.1 (q, C-1), 24.0 (t, C-7), 21.8 (q, C-14), 11.1 (q, C-8).

HRMS (CI): Calculated for $C_{18}H_{28}N_3O_5S^+$ $[M+H]^+$: 398.1744, found: 398.1748.

tert-Butyl (2*S*,4*R*)-*N*-Benzyloxycarbonyl-4-Methyl-prolinate [12]

According to **GP2**, 1.11 g (2.89 mmol, 1.0 eq.) of the tosylate **10** were reacted in 12 mL MeOH with 111 mg Pd/C (10 wt%) under H_2 atmosphere. After 23 h, the residue obtained was dissolved in 12 mL MeOH and cooled to 0 °C. Subsequently, 1.21 mL (8.67 mmol, 3.0 eq., ρ = 0.726 g/mL) Et_3N and dropwise 0.46 mL (3.22 mmol, 1.1 eq., ρ = 1.195 g/mL) CbzCl were added to the solution and slowly warmed to rt after the addition was completed. After 3 d, the solvent was removed in vacuo and the residue was taken up in EtOAc. The organic phase was washed three times with 10 wt% aqueous citric acid solution and twice with water. It was then dried over $MgSO_4$ and the solvent was removed in vacuo. The residue obtained was purified by automated column chromatography (cHex/EtOAc 1:0 ⟶ 8:2), resulting in the isolation of 787 mg (1.17 mmol, 85%) of the proline derivative **12** as a colorless oil.

$[\alpha]_D^{20}$ = −30.7 (c = 1.0, $CHCl_3$)   **R_f** = 0.60 (*n*-Pentane/EtOAc 1:1)

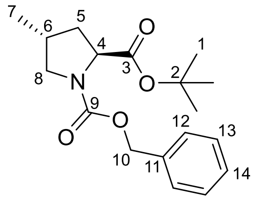

^{1}H-NMR (500 MHz, DMSO-d_6, 373 K): δ = 7.33–7.37 (m, 4 H, 12-H, 13-H), 7.30 (m, 1 H, 14-H), 5.07 (s, 2 H, 10-H), 4.21 (dd, $^3J_{4,5b}$ = 8.9 Hz, $^3J_{4,5a}$ = 2.4 Hz, 1 H, 4-H), 3.62 (dd, $^2J_{8a,8b}$ = 10.2 Hz, $^3J_{8a,6}$ = 7.6 Hz, 1 H, 8-H_a), 2.98 (dd, $^2J_{8b,8a}$ = 10.2 Hz, $^3J_{8b,6}$ = 7.9 Hz, 1 H, 8-H_b), 2.33 (m, 1 H, 6-H), 2.00 (ddd, $^2J_{5a,5b}$ = 12.6 Hz, $^3J_{5a,6}$ = 6.5 Hz, $^3J_{5a,4}$ = 2.5 Hz, 1 H, 5-H_a), 1.86 (m, 1 H, 5-H_b), 1.38 (s, 9 H, 1-H), 1.01 (d, $^3J_{7,6}$ = 6.6 Hz, 3 H, 7-H).

^{13}C-NMR (126 MHz, DMSO-d_6, 373 K): δ = 170.7 (s, C-3), 153.3 (s, C-9), 136.5 (s, C-11), 127.7 (d, C-13), 127.1 (d, C-14), 126.8 (d, C-12), 80.0 (s, C-2), 65.5 (t, C-10), 59.2 (d, C-4), 52.9 (t, C-8), 37.2 (t, C-5), 30.4 (d, C-6), 27.1 (q, C-1), 16.7 (q, C-7).

HRMS (CI): Calculated for $C_{18}H_{26}NO_4^+$ $[M+H]^+$: 320.1856, found: 320.1868.

tert-Butyl (2*S*,4*R*)-*N*-benzyloxycarbonyl-4-ethyl-prolinate [13]

According to **GP2**, 922 mg (2.32 mmol, 1.0 eq.) of the tosylate **11** were reacted in 12 mL MeOH with 99.1 mg Pd/C (10 wt%) under H_2 atmosphere. After 1.5 d, the resulting residue was dissolved in 12 mL MeOH and cooled to 0 °C. Subsequently, 970 µL (6.96 mmol, 3.0 eq., ρ = 0.726 g/mL) Et_3N and 361 µL (2.53 mmol, 1.1 eq., ρ = 1.195 g/mL) CbzCl were added dropwise to the solution and slowly warmed to rt after the addition was completed. After 3 h, the solvent was removed in vacuo and the residue was taken up in EtOAc. The organic phase was washed three times with 10 wt% aqueous citric acid solution and twice with water. It was then dried over $MgSO_4$, and the solvent was removed in vacuo. Lastly, the residue obtained was purified by automated column chromatography (cHex/EtOAc 100:0 ⟶ 75:25), resulting in the isolation of 702 mg (2.10 mmol, 91%) of the proline derivative **13** as a colorless oil.

$[\alpha]_D^{20}$ = −28.0 (c = 1.0, $CHCl_3$)   **R_f** = 0.65 (*n*-Pentane/EtOAc 1:1)

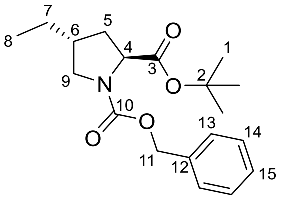

^{1}H-NMR (500 MHz, DMSO-*d*$_6$, 373 K): δ = 7.32–7.37 (m, 4 H, 13-H, 14-H), 7.30 (m, 1 H, 15-H), 5.07 (s, 2 H, 10-H), 4.21 (dd, $^{3}J_{4,5b}$ = 9.4 Hz, $^{3}J_{4,5a}$ = 2.3 Hz, 1 H, 4-H), 3.62 (dd, $^{2}J_{9a,9b}$ = 10.3 Hz, $^{3}J_{9a,6}$ = 7.7 Hz, 1 H, 9-H$_a$), 3.03 (dd, $^{2}J_{9b,9a}$ = 10.2 Hz, $^{3}J_{9b,6}$ = 8.2 Hz, 1 H, 9-H$_b$), 2.16 (m, 1 H, 6-H), 2.01 (ddd, $^{2}J_{5a,5b}$ = 12.1 Hz, $^{3}J_{5a,6}$ = 6.2 Hz, $^{3}J_{5a,4}$ = 2.3 Hz, 1 H, 5-H$_a$), 1.88 (m, 1 H, 5-H$_b$), 1.38 (s, 9 H, 1-H), 1.33–1.43 (m, 2 H, 7-H), 0.89 (t, $^{3}J_{7,6}$ = 7.4 Hz, 3 H, 8-H).

^{13}C-NMR (126 MHz, DMSO-*d*$_6$, 373 K): δ = 170.8 (s, C-3), 153.3 (s, C-10), 136.5 (s, C-12), 127.7 (d, C-14), 127.1 (d, C-15), 126.8 (d, C-13), 80.0 (s, C-2), 65.5 (t, C-11), 59.1 (d, C-4), 51.8 (t, C-9), 37.5 (d, C-5), 34.9 (t, C-6), 27.1 (q, C-1), 24.8 (t, C-7), 11.3 (q, C-8).

HRMS (CI): Calculated for $C_{19}H_{28}NO_4^+$ $[M+H]^+$: 334.2013, found: 334.2004.

(2*S*,4*R*)-*N*-Benzyloxycarbonyl-4-ethyl-proline [13']

1.12 g (3.37 mmol, 1.0 eq.) of the proline derivative **13** were dissolved in a mixture of 17 mL $CHCl_3$ and 5.6 mL TFA and heated to 50 °C. After 6 h, the yellow solution was cooled to rt, the solvent was removed in vacuo and the residue was co-evaporated three times with toluene. Automated column chromatography (cHex/EtOAc/AcOH 100:0:0 ⟶ 50:50:2) afforded 841 mg (3.03 mmol, 90%) of the carboxylic acid **13'** as a colorless oil.

$[\alpha]_D^{20}$ = −37.7 (c = 1.0, $CHCl_3$) **R$_f$** = 0.43 (*n*-Pentane/EtOAc 1:1)

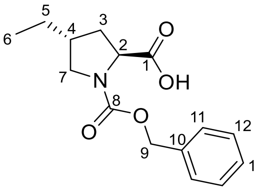

Main rotamer:

^{1}H-NMR (500 MHz, $CDCl_3$): δ = 8.33 (bs, 1 H, COOH), 7.26–7.40 (m, 5 H, 11-H, 12-H, 13-H), 5.17 (m, 2 H, 9-H), 4.46 (d, $^{3}J_{2,3a} \approx {}^{3}J_{2,3b}$ = 8.6 Hz, 1 H, 2-H), 3.72 (dd, $^{2}J_{7a,7b}$ = 10.4 Hz, $^{3}J_{7a,4}$ = 7.4 Hz, 1 H, 7-H$_a$), 3.00 (t, $^{2}J_{7b,7a} \approx {}^{3}J_{7b,4}$ = 9.8 Hz, 1 H, 7-H$_b$), 2.18–2.36 (m, 2 H, 3-H$_b$, 4-H), 1.77 (q, $^{2}J_{3a,3b} \approx {}^{3}J_{3a,2} \approx {}^{3}J_{3a,4}$ = 10.9 Hz, 1 H, 3-H$_a$), 0.93 (t, $^{3}J_{6,5}$ = 7.4 Hz, 3 H, 6-H), 1.40 (m, 2 H, 5-H).

^{13}C-NMR (126 MHz, $CDCl_3$): δ = 178.3 (s, C-1), 156.2 (s, C-8), 136.3 (s, C-10), 128.7 (d, C-12), 128.1 (d, C-13), 127.8 (d, C-11), 67.8 (t, C-9), 59.7 (d, C-2), 52.1 (t, C-7), 39.4 (t, C-4), 34.9 (t, C-3), 25.9 (t, C-5), 12.6 (q, C-6).

Minor rotamer (selected signals):

^{1}H-NMR (500 MHz, $CDCl_3$): δ = 5.20 (d, $^{2}J_{9a,9b}$ = 12.4 Hz, 2 H, 9-H), 4.42 (d, $^{3}J_{2,3a}$ ≈ $^{3}J_{2,3b}$ = 8.9 Hz, 1 H, 2-H), 3.80 (dd, $^{2}J_{7b,7a}$ = 10.3 Hz, $^{3}J_{7b,4}$ = 7.6 Hz, 1 H, 7-H_a), 3.07 (t, $^{2}J_{7b,7a}$ ≈ $^{3}J_{7b,4}$ = 9.4 Hz, 1 H, 7-H_b), 1.90 (q, $^{2}J_{3b,3a}$ ≈ $^{3}J_{3b,2}$ ≈ $^{3}J_{3b,4}$ = 10.4 Hz, 1 H, 3-H_b).

^{13}C-NMR (126 MHz, $CDCl_3$): δ = 176.0 (s, C-1), 154.5 (s, C-8), 136.6 (s, C-10), 128.5 (d, C-12), 128.3 (d, C-13), 128.0 (d, C-11), 67.2 (t, C-9), 59.0 (d, C-2), 52.2 (t, C-7), 38.3 (d, C-4), 36.6 (t, C-3), 26.0 (t, C-5).

Rotamer ratio: 6:4

HRMS (ESI): Calculated for $C_{15}H_{20}NO_4^+$ $[M+H]^+$: 278.1387, found: 278.1384.

Synthesis of the other Amino Acid Building Blocks

N-Benzyloxycarbonyl-*O*-*tert*-butyldimethylsilyl-L-threonine [S1][10]

Under N_2 atmosphere, a solution of 5.56 g (81.67 mmol, 3.0 eq.) imidazole in 7 mL anhydrous DMF was added to a solution of 6.89 g (27.21 mmol, 1.0 eq.) Cbz-L-Thr-OH in 16 mL anhydrous DMF at 0 °C, followed by a solution of 12.3 g (81.61 mmol, 3.0 eq.) TBS-Cl in 22 mL anhydrous DMF, and warmed to rt after complete addition. After 27 h, the yellow solution was poured into approx. 300 mL ice water and extracted three times with Et_2O and dried over Na_2SO_4. After the solvent was removed in vacuo, the residue was dissolved in 54 mL THF, mixed with 54 mL 0.5 M $KOH_{(aq.)}$ and stirred for 4 h at 0 °C. The mixture was then extracted once with Et_2O, whereby this organic phase was discarded. The aqueous phase was acidified with 1 M $HCl_{(aq.)}$ solution to a pH of 2–3 and then extracted three times with Et_2O. Finally, the combined etheric phases were washed once with sat. $NaCl_{(aq.)}$ solution and dried over Na_2SO_4. After removing the solvent in vacuo, 9.38 g (25.53 mmol, 94%) of Cbz-*O*-TBS-L-Thr-OH (**S1**) was isolated as a colorless solid.

$[\alpha]_D^{20}$ = +10.1 (c = 1.0, $CHCl_3$); Lit.: $[\alpha]_D^{23}$ = +13.2 (c = 1.0, $CHCl_3$)[11]

R_f = 0.79 (DCM/MeOH 95:5) **Melting range**: 157–159 °C (Lit.: 150.5–152.5 °C)[10]

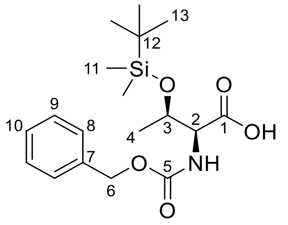

^{1}H-NMR (500 MHz, $CDCl_3$): δ = 7.36–7.40 (m, 3 H, 8-H, 10-H), 7.30–7.37 (m, 2 H, 9-H), 5.53 (d, $^{3}J_{NH,2}$ = 8.1 Hz, 1 H, NH), 5.14 (s, 2 H, 6-H), 4.48 (qd, $^{3}J_{3,4}$ = 6.3 Hz, $^{3}J_{3,2}$ = 2.8 Hz, 1 H, 3-H), 4.33 (dd, $^{3}J_{2,NH}$ = 8.1 Hz, $^{3}J_{2,3}$ = 2.9 Hz, 1 H, 2-H), 1.21 (d, $^{3}J_{4,3}$ = 6.3 Hz, 3 H, 4-H), 0.88 (s, 9 H, 13-H), −0.10 (m, 6 H, 11-H).

^{13}**C-NMR** (126 MHz, $CDCl_3$): δ = 173.6 (s, C-1), 156.6 (s, C-5), 136.3 (s, C-7), 128.7 (d, C-9), 128.4 (d, C-10), 128.3 (d, C-8), 68.6 (d, C-3), 67.4 (t, C-6), 59.4 (d, C-2), 25.8 (q, C-13), 19.6 (q, C-4), 18.0 (s, C-12), −4.5 (q, C-11), −5.1 (q, C-11').

HRMS (ESI): Calculated for $C_{18}H_{29}NNaO_5Si^+$ $[M+Na]^+$: 390.1707, found: 390.1707.

N-Benzyloxycarbonyl-*N*-methyl-*O*-*tert*-butyldimethylsilyl-L-threonine [S2]

Under an N_2-atmosphere, 2.63 g (7.15 mmol, 1.0 eq.) of Cbz-*O*-TBS-L-Thr-OH (**S1**) was dissolved in 36 mL anhydrous THF and cooled to 0 °C. Subsequently, the solution was treated with 2.30 mL (36.78 mmol, 5.1 eq., ρ = 2.270 g/mL) methyl iodide, followed by portionwise addition of 1.44 g (60 wt% in mineral oil, 35.98 mmol, 5.0 eq.) NaH. After the addition was complete, the reaction mixture was slowly warmed to rt overnight (19 h).

The reaction mixture was then carefully quenched with H_2O until no further gas evolution was observed. Subsequently, the mixture was extracted once with *n*-pentane, and the aqueous phase was acidified to a pH of approx. 2 with 1 M $HCl_{(aq.)}$, resulting in the formation of a colorless precipitate. Next, the aqueous phase was extracted three times with EtOAc, and the combined EtOAc phases were washed twice with sat. $Na_2S_2O_{3(aq.)}$ and once with sat. $NaCl_{(aq.)}$ solution, before the now nearly colorless organic phase was dried over $MgSO_4$ and the solvent was removed in vacuo. The residue obtained was purified by automated column chromatography (SiO_2, cHex/EtOAc/AcOH 100:0:0 ⟶ 50:50:1), whereby the isolated fraction was co-evaporated three times with toluene. A total of 2.20 g (5.78 mmol, 81%) of *N*-methylated amino acid **S2** was isolated as a colorless, highly viscous oil

$[\alpha]_D^{20}$ = +13.4 (c = 1.0, $CHCl_3$) **R$_f$** = 0.07 (*n*-Pentane/EtOAc/AcOH 80:20:1)

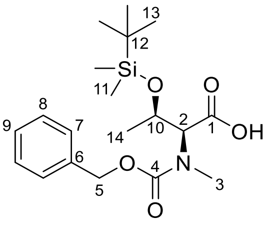

Main rotamer:

^{1}**H-NMR** (400 MHz, $CDCl_3$): δ = 7.27–7.41 (m, 5 H, 7-H, 8-H, 9-H), 5.16 (m, 2 H, 5-H), 4.93 (d, $^3J_{2,10}$ = 3.9 Hz, 1 H, 2-H), 4.61 (qd, $^3J_{10,14}$ = 6.3 Hz, $^3J_{10,2}$ = 4.0 Hz, 1 H, 10-H), 3.12 (s, 3 H, 3-H), 1.20 (d, $^3J_{14,10}$ = 6.3 Hz, 3 H, 14-H), 0.84 (s, 9 H, 13-H), 0.08 (s, 3 H, 11-H), 0.04 (s, 3 H, 11'-H).

^{13}**C-NMR** (101 MHz, $CDCl_3$): δ = 175.5 (s, C-1), 157.8 (s, C-4), 136.6 (s, C-6), 128.6 (d, C-8), 128.1 (d, C-9), 127.8 (d, C-7), 69.1 (d, C-10), 67.8 (t, C-5), 64.1 (d, C-2), 33.7 (q, C-3), 25.8 (q, C-13), 20.6 (q, C-14), 17.9 (s, C-12), −4.3 (q, C-11), −5.2 (q, C-11').

Minor rotamer (selected signals):

^{1}**H-NMR** (400 MHz, $CDCl_3$): δ = 4.77 (d, $^3J_{2,10}$ = 4.0 Hz, 1 H, 2-H), 4.53 (qd, $^3J_{10,14}$ = 6.4 Hz, $^3J_{10,2}$ = 4.0, 1 H, 10-H), 3.11 (s, 3 H, 3-H), 1.16 (d, $^3J_{14,10}$ = 6.3 Hz, 3 H, 14-H), 0.06 (s, 3 H, 11-H'), 0.02 (s, 3 H, 11-H).

^{13}**C-NMR** (101 MHz, $CDCl_3$): δ = 156.6 (s, C-4), 136.5 (s, C-6), 128.3 (d, C-9), 69.0 (d, C-10), 67.9 (t, C-5), 63.7 (d, C-2), 33.7 (q, C-3), 20.7 (q, C-14).

Rotamer ratio: 8:2

HRMS (CI): Calculated for $C_{19}H_{32}NO_5Si^+$ $[M+H]^+$: 382.2044, found: 382.2059.

N-Allyloxycarbonyl-L-valine [S3][12]

To a solution of 5.87 g (50.11 mmol, 1.0 eq.) L-Val-OH and 10.4 g (75.25 mmol, 1.5 eq.) K_2CO_3 in 110 mL water and 90 mL THF, a solution of 6.3 mL (59.06 mmol, 1.2 eq., ρ = 1.13 g/mL) allyl chloroformate in 20 mL THF was slowly added dropwise at 0 °C over a period of 15 min. After the addition was complete, the reaction was slowly warmed to rt and stirred for 3 d. The reaction mixture was concentrated in vacuo and extracted twice with Et_2O. The aqueous phase was acidified with 6 M $HCl_{(aq.)}$ to a pH of 1, whereby a colorless solid precipitated. After the suspension was extracted three times with DCM, the combined DCM phases were dried over $MgSO_4$. Removal of the solvent in vacuo afforded 9.99 g (49.65 mmol, 99%) of Alloc-L-Val-OH (**S3**) as a pale-yellow resin.

$[\alpha]_D^{20}$ = −17.3 (c = 1.0, $CHCl_3$); Lit.: $[\alpha]_D^{20}$ = −19.6 (c = 0.7, $CHCl_3$)[13]

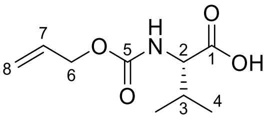

Main rotamer:

^{1}**H-NMR** (400 MHz, $CDCl_3$): δ = 8.92 (bs, 1 H, COOH), 5.94 (m, 1 H, 7-H), 5.17–5.42 (m, 3 H, NH, 8-H), 4.61 (d, $^3J_{6,7}$ = 5.7 Hz, 2 H, 6-H), 4.36 (dd, $^3J_{2,NH}$ = 9.1 Hz, $^3J_{2,3}$ = 4.5 Hz, 1 H, 2-H), 2.26 (m, 1 H, 3-H), 1.03 (d, $^3J_{4',3}$ = 6.8 Hz, 3 H, 4-H'), 0.96 (d, $^3J_{4,3}$ = 6.8 Hz, 3 H, 4-H).

^{13}**C-NMR** (101 MHz, $CDCl_3$): δ = 176.9 (s, C-1), 156.4 (s, C-5), 132.6 (d, C-7), 118.2 (t, C-8), 66.2 (t, C-6), 58.9 (d, C-2), 31.1 (d, C-3), 19.2 (q, C-4), 17.5 (q, C-4').

Minor rotamer (selected signals):

^{1}**H-NMR** (400 MHz, $CDCl_3$): δ = 6.12 (d, $^3J_{NH,2}$ = 8.6 Hz, 1 H, NH), 4.20 (m, 1 H, 2-H).

^{13}**C-NMR** (101 MHz, $CDCl_3$): δ = 176.6 (s, C-1), 132.2 (d, C-7), 66.6 (d, C-6), 59.8 (d, C-2).

Rotamer ratio: 8:2

HRMS (ESI): Calculated for $C_9H_{16}NO_4^+$ $[M+H]^+$: 202.1074, found: 202.1073.

N-Allyloxycarbonyl-*N*-methyl-L-valine [S4]

Under an N_2-atmosphere, 1.99 g (9.90 mmol, 1.0 eq.) of Alloc-L-Val-OH (**S3**) was dissolved in 30 mL anhydrous THF and cooled to 0 °C. Subsequently, the solution was treated with 3.6 mL (57.57 mmol, 5.8 eq., ρ = 2.270 g/mL) methyl iodide, followed by portionwise addition of 1.99 g

(60 wt% in mineral oil, 49.76 mmol, 5.0 eq.) NaH. After the addition was complete, the reaction mixture was slowly warmed to rt overnight (22.5 h).

The reaction mixture was then carefully quenched with H_2O until no further gas evolution was observed and afterwards condensed in vacuo. Subsequently, the mixture was extracted once with *n*-pentane, and the aqueous phase was acidified to a pH of approx. 1 with 1 M $KHSO_{4(aq.)}$, resulting in the formation of a colorless precipitate. Next, the aqueous phase was extracted three times with Et_2O, and the combined Et_2O phases were washed once with sat. $Na_2S_2O_{3(aq.)}$ and once with sat. $NaCl_{(aq.)}$ solution, before the now nearly colorless organic phase was dried over $MgSO_4$ and the solvent was removed in vacuo. The residue obtained was purified by automated column chromatography (SiO_2, cHex/EtOAc/AcOH 100:0:0 ⟶ 80:20:0.4), whereby the isolated fraction was co-evaporated three times with toluene. A total of 1.83 g (8.54 mmol, 86%) of Alloc-*N*-Me-Val-OH (**S4**) was isolated as a yellow oil.

$[\alpha]_D^{20}$ = −79.5 (c = 1.0, $CHCl_3$) **R_f** = 0.16 (*n*-Pentane/EtOAc/AcOH 80:20:1)

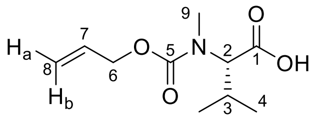

Main rotamer:

¹H-NMR (400 MHz, $CDCl_3$): δ = 9.45 (bs, 1 H, COOH), 5.93 (ddt, $^3J_{7,8b}$ = 16.9 Hz, $^3J_{7,8a}$ = 11.3 Hz, $^3J_{7,6}$ = 5.3 Hz, 1 H, 7-H), 5.31 (d, $^3J_{8b,7}$ = 17.2 Hz, 1 H, 8-H_b), 5.21 (m, 1 H, 8-H_a), 4.63 (dt, $^3J_{6,7}$ = 5.4 Hz, $^4J_{6,8}$ = 1.5 Hz, 2 H, 6-H), 4.36 (d, $^3J_{2,3}$ =10.4 Hz, 1 H, 2-H), 2.93 (s, 3 H, 9-H), 2.24 (m, 1 H, 3-H), 1.04 (d, $^3J_{4',3}$ = 6.0 Hz, 3 H, 4-H), 0.93 (d, $^3J_{4,3}$ = 6.7 Hz, 3 H, 4-H').

¹³C-NMR (101 MHz, $CDCl_3$): δ = 175.5 (s, C-1), 157.4 (s, C-5), 132.7 (d, C-7), 117.8 (t, C-8), 66.8 (t, C-6), 64.4 (d, C-2), 30.8 (q, C-9), 27.6 (d, C-3), 19.8 (q, C-4), 19.2 (q, C-4').

Minor rotamer (selected signals):

¹H-NMR (400 MHz, $CDCl_3$): δ = 4.31 (d, $^3J_{2,3}$ = 10.4 Hz, 1 H, 2-H).

¹³C-NMR (101 MHz, $CDCl_3$): δ = 176.1 (s, C-1), 156.3 (s, C-5), 132.8 (d, C-7), 117.5 (t, C-8), 66.6 (t, C-6), 65.4 (d, C-2), 31.7 (q, C-9), 27.7 (d, C-3'), 19.9 (q, C-4).

Rotamer ratio: 6:4

HRMS (CI): Calculated for $C_{10}H_{18}NO_4^+$ $[M+H]^+$: 216.1230, found: 216.1234.

N-*tert*-Butyloxycarbonyl-*N*-methyl-D-leucine [S5][8]

Under an N_2-atmosphere, 5.81 g (25.11 mmol, 1.0 eq.) Boc-D-Leu-OH was dissolved in 80 mL anhydrous THF and cooled to 0 °C. Subsequently, the solution was treated with 4.6 mL (73.57 mmol, 2.9 eq., ρ = 2.270 g/mL) methyl iodide, followed by portionwise addition of 6.00 g (60 wt% in mineral oil, 151 mmol, 6.0 eq.) NaH. After the addition was complete, the reaction mixture was slowly warmed to rt overnight (19 h).

The reaction mixture was then carefully quenched with H_2O until no further gas evolution was observed. Subsequently, the mixture was extracted once with *n*-pentane, and the aqueous phase was acidified to a pH of approx. 2–3 with 1 M $KHSO4_{(aq.)}$, resulting in the formation of a colorless precipitate. Next, the aqueous phase was extracted three times with Et_2O, and the combined Et_2O phases were washed once with sat. $Na_2S_2O_{3(aq.)}$ and once with sat. $NaCl_{(aq.)}$ solution, before the now nearly colorless organic phase was dried over $MgSO_4$ and the solvent was removed in vacuo. A total of 5.77 g (23.52 mmol, 94%) of Boc-*N*-Me-D-Leu-OH (**S5**) was isolated as a yellow oil.

$[\alpha]_D^{20}$ = +28.5 (c = 1.0, $CHCl_3$); Lit.: $[\alpha]_D^{20}$ = +33.5 (c = 0.86, $CHCl_3$)[9]

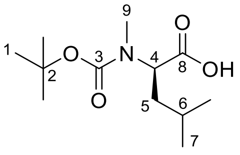

Rotamer 1:

^{1}H-NMR (400 MHz, $CDCl_3$): δ = 8.22 (bs, 1 H, COOH). 4.61 (dd, $^3J_{4,5a}$ = 10.9 Hz, $^3J_{4,5b}$ = 4.7 Hz, 1 H, 4-H), 2.78 (s, 3 H, 9-H), 1.72 (m, 2 H, 5-H), 1.57 (m, 1 H, 6-H), 1.46 (s, 9 H, 1-H), 0.96 (d, $^3J_{7',6}$ = 7.2 Hz, 3 H, 7-H'), 0.93 (d, $^3J_{7,6}$ = 7.2 Hz, 3 H, 7-H).

^{13}C-NMR (101 MHz, $CDCl_3$): δ = 177.9 (s, C-8), 155.7 (s, C-3), 80.5 (s, C-2), 57.0 (d, C-4), 37.3 (t, C-5), 30.5 (q, C-9), 28.3 (q, C-1), 24.6 (d, C-6), 23.2 (q, C-7), 21.1 (q, C-7').

Rotamer 2 (selected signals):

^{1}H-NMR (400 MHz, $CDCl_3$): δ = 4.83 (t, $^3J_{4,5a} \approx {}^3J_{4,5b}$ = 8.0 Hz, 1 H, 4-H), 2.81 (s, 3 H, 9-H), 1.45 (s, 9 H, 1-H).

^{13}C-NMR (101 MHz, $CDCl_3$): δ = 177.7 (s, C-8), 156.6 (s, C-3), 80.6 (s, C-2), 56.3 (d, C-4), 37.8 (t, C-5), 30.8 (q, C-9), 24.9 (d, C-6), 23.2 (q, C-7), 21.3 (q, C-7').

Rotamer ratio: 1:1

HRMS (ESI): Calculated for $C_{12}H_{24}NO_4^+$ $[M+H]^+$: 246.1700, found: 246.1698.

Methyl *N*-*tert*-butyloxycarbonyl-*N*-methyl-D-leucinate [19]

A solution of 5.73 g (23.37 mmol, 1.0 eq.) Boc-*N*-Me-D-Leu-OH (**S5**) in 24 mL DMF was treated with 9.69 g (70.10 mmol, 3.0 eq.) K_2CO_3 and 3.65 mL (58.43 mmol, 2.5 eq., ρ = 2.270 g/mL) methyl iodide at 0 °C and slowly warmed to rt after addition. After 19 h, the mixture was diluted with EtOAc and washed twice with sat. $NaHCO_{3(aq.)}$ and once with sat. $NaCl_{(aq.)}$ solution. The mixture was then dried over $MgSO_4$, and the solvent was removed in vacuo. Automated column chromatography (cHex/EtOAc 1:0 ⟶ 1:1) afforded 5.59 g (21.6 mmol, 92%) of Boc-*N*-Me-D-Leu-OMe (**19**) as a pale-yellow liquid.

$[\alpha]_D^{20}$ = +38.6 (c = 1.0, $CHCl_3$)   **R_f** = 0.50 (*n*-Pentane/EtOAc 8:2)

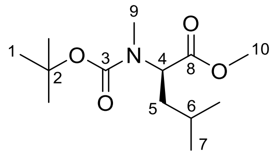

Rotamer 1:

^{1}H-NMR (500 MHz, $CDCl_3$): δ = 4.57 (dd, $^{3}J_{4,5a}$ = 10.9 Hz, $^{3}J_{4,5b}$ = 4.8 Hz, 1 H, 4-H), 3.70 (s, 3 H, 10-H), 2.76 (s, 3 H, 9-H), 1.66 (m, 2 H, 5-H), 1.54 (m, 1 H, 6-H), 1.44 (s, 9 H, 1-H), 0.94 (d, $^{3}J_{7,6}$ = 6.7 Hz, 3 H, 7-H), 0.93 (d, $^{3}J_{7',6}$ = 6.4 Hz, 3 H, 7-H'),

^{13}C-NMR (126 MHz, $CDCl_3$): δ = 172.9 (s, C-8), 155.8 (s, C-3), 80.1 (s, C-2), 56.0 (d, C-4), 52.1 (q, C-10), 37.8 (t, C-5), 30.5 (q, C-9), 28.5 (q, C-1), 24.8 (d, C-6), 23.4 (q, C-7'), 21.4 (q, C-7).

Rotamer 2 (selected signals):

^{1}H-NMR (500 MHz, $CDCl_3$): δ = 4.87 (t, $^{3}J_{4,5a} \approx {}^{3}J_{4,5b}$ = 8.0 Hz, 1 H, 4-H), 3.69 (s, 3 H, 10-H), 1.46 (s, 9 H, 1-H), 2.80 (s, 3 H, 9-H).

^{13}C-NMR (126 MHz, $CDCl_3$): δ = 173.2 (s, C-8), 156.5 (s, C-3), 80.3 (s, C-2), 57.4 (d, C-4), 38.2 (t, C-5), 30.7 (q, C-9), 25.1 (d, C-6).

Rotamer ratio: 1:1

HRMS (ESI): Calculated for $C_{13}H_{26}NO_4^+$ $[M+H]^+$: 260.1856, found: 260.1852.

Synthesis of the Peptide Fragments

Synthesis of the Hexapeptide Fragment

Cbz-L-Leu-L-MePro-O*t*-Bu [14]

According to **GP2**, 1.01 g (3.17 mmol, 1.0 eq.) of Cbz-L-MePro-O*t*-Bu (**12**) was hydrogenated with 100 mg (10 wt%) Pd/C in 13 mL MeOH for 3 h. The resulting free amine was coupled according to **GP4** with 1.16 g (4.35 mmol, 1.4 eq.) Cbz-L-Leu-OH, 682 mg (4.45 mmol, 1.4 eq.) HOBt, 850 mg (4.44 mmol, 1.4 eq.) EDC, and 871 µL (7.92 mmol, 2.5 eq., ρ = 0.920 g/mL) NMM for 14.5 h. Automated column chromatography (SiO_2, cHex/EtOAc 10:0 ⟶ 7:3) of the crude product yielded 1.24 g (2.87 mmol, 90%) of dipeptide **14** as a colorless resin.

$[\alpha]_D^{20}$ = −48.7 (c = 1.0, $CHCl_3$) **R_f** = 0.57 (*n*-Pentane/EtOAc 1:1)

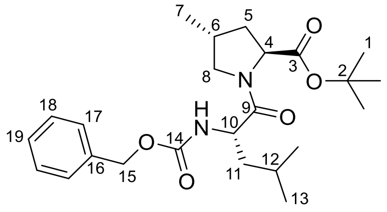

^{1}H-NMR (500 MHz, DMSO-d_6, 373 K): δ = 7.26–7.39 (m, 5 H, 17-H, 18-H, 19-H), 6.95 (bs, 1 H, NH), 5.04 (m, 2 H, 15-H), 4.24–4.35 (m, 2 H, 4-H, 10-H), 3.66 (dd, $^{2}J_{8a,8b}$ = 9.6 Hz, $^{3}J_{8a,6}$ = 7.2 Hz, 1 H, 8-H_a), 3.21 (bs, 1 H, 8-H_b), 2.41 (m, 1 H, 6-H), 1.96 (m, 1 H, 5-H_a), 1.69–1.81 (m, 2 H, 5-H_b,

12-H), 1.51 (m, 2 H, 11-H), 1.40 (s, 9 H, 1-H), 1.00 (d, $^{3}J_{7,6}$ = 6.7 Hz, 3 H, 7-H), 0.92 (d, $^{3}J_{13,12}$ = 6.6 Hz, 6 H, 13-H).

^{13}C-NMR (126 MHz, DMSO-d_6, 373 K): δ = 170.2 (s, C-9), 170.1 (s, C-3), 155.3 (s, C-14), 136.7 (d, C-16), 127.7 (d, C-18), 127.1 (d, C-19), 127.0 (d, C-17), 79.8 (s, C-2), 65.0 (t, C-15), 58.9 (d, C-4), 52.7 (t, C-8), 50.4 (d, C-10), 35.6 (t, C-5), 31.6 (d, C-6), 27.2 (q, C-1), 23.7 (d, C-12), 22.4 (q, C-13), 21.1 (q, C-13'), 16.7 (q, C-7).

The signal of C-11 lies beneath the solvent peak.

HRMS (ESI): Calculated for $C_{24}H_{37}N_2O_5^+$ $[M+H]^+$: 433.2700, found: 433.2697.

Cbz-*O*-TBS-*N*-Me-L-Thr-L-Leu-L-MePro-O*t*-Bu [15]

According to **GP2**, 1.12 g (2.58 mmol, 1.0 Äq.) of the dipeptide **14** was hydrogenated with 117 mg (10 wt%) Pd/C in 9.2 mL MeOH for 3 h. After addition of Pd/C, 650 µL (4.0 M in 1,4-dioxane, 2.60 mmol, 1.0 eq.) of HCl was added before stirring under H_2 atmosphere.

Then, under an Ar atmosphere, 988 mg (2.59 mmol, 1.0 eq.) of Cbz-*O*-TBS-*N*-Me-L-Thr-OH (**S2**) was dissolved in 14 mL of anhydrous THF and cooled to −20 °C before 625 µL (5.68 mmol, 2.2 eq., ρ = 0.920 g/mL) NMM was added. Subsequently, 340 µL (2.59 mmol, 1.0 eq., ρ = 1.040 g/mL) IBCF was added dropwise, and the resulting colorless suspension was stirred for 15 min at −20 °C. A solution of the previously obtained free amine in 8 mL DCM was then added dropwise, followed by two rinses with 1.5 mL of DCM each, and the mixture was slowly warmed to rt.

After 1.5 d, the reaction mixture was concentrated in vacuo and the residue was taken up in EtOAc before washing with 1 M $KHSO_{4(aq.)}$, H_2O, sat. $NaHCO_{3(aq.)}$ and sat. $NaCl_{(aq.)}$ solution. The organic phase was then dried over $MgSO_4$ and concentrated in vacuo. Automated column chromatography of the residue (SiO_2, cHex/EtOAc 100:0 ⟶ 65:35) yielded 1.58 g (2.39 mmol, 92%) of tripeptide **15** as a colorless foam.

$[\alpha]_D^{20}$ = −42.7 (c = 1.0, $CHCl_3$) **R_f** = 0.57 (*n*-Pentane/EtOAc 1:1)

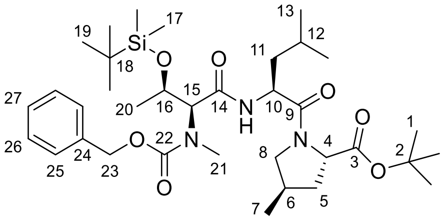

^{1}H-NMR (500 MHz, DMSO-d_6, 373 K): δ = 7.72 (d, $^{3}J_{NH,10}$ = 7.6 Hz, 1 H, NH), 7.33–7.38 (m, 4 H, 25-H, 26-H), 7.31 (m, 1 H, 27-H), 5.11 (s, 2 H, 23-H), 4.58 (m, 1 H, 10-H), 4.54 (d, $^{3}J_{15,16}$ = 6.2 Hz, 1 H, 15-H), 4.39 (m, 1 H, 16-H), 4.26 (m, 1 H, 4-H), 3.68 (t, $^{2}J_{8a,8b}$ ≈ $^{3}J_{8a,6}$ = 8.1 Hz, 1 H, 8-H_a), 3.25 (t, $^{2}J_{8b,8a}$ ≈ $^{3}J_{8b,6}$ = 8.2 Hz, 1 H, 8-H_b), 2.99 (s, 3 H, 21-H), 2.43 (m, 1 H, 6-H), 1.98 (m, 1 H, 5-H_a), 1.77 (m, 1 H, 5-H_b), 1.66 (m, 1 H, 12-H), 1.51 (m, 2 H, 11-H), 1.41 (s, 9 H, 1-H), 1.14 (d, $^{3}J_{20,16}$ = 6.0 Hz, 3 H, 20-H), 1.03 (d, $^{3}J_{6,7}$ = 6.6 Hz, 3 H, 7-H), 0.90 (d, $^{3}J_{13,12}$ = 6.1 Hz, 6 H, 13-H), 0.83 (s, 9 H, 19-H), 0.07 (s, 3 H, 17-H'), 0.02 (s, 3 H, 17-H).

^{13}C-NMR (126 MHz, DMSO-*d*$_6$, 373 K): δ = 170.2 (s, C-3), 169.6 (s, C-9), 168.0 (s, C-14), 155.7 (s, C-22), 136.5 (s, C-24), 127. 7 (d, C-26), 127.0 (d, C-27), 126.7 (d, C-25), 79.8 (s, C-2), 66.7 (d, C-16), 65.9 (t, C-23), 63.6 (d, C-15), 58.9 (d, C-4), 52.7 (t, C-8), 48.3 (d, C-10), 35.6 (t, C-5), 31.5 (d, C-6), 31.5 (q, C-21), 27.2 (q, C-1), 25.1 (q, C-19), 23.7 (d, C-12), 22.4 (q, C-13), 21.1 (q, C-13'), 20.3 (q, C-20), 16.9 (s, C-18), 16.8 (q, C-7), −4.9 (q, C-17), −5.6 (q, C-17').

HRMS (CI): Calculated for $C_{31}H_{50}N_3O_7Si^+$ $[M-C_4H_9]^+$: 604.3413, found: 604.3438.

Cbz-*N*-Me-L-Thr-L-Leu-L-MePro-O*t*-Bu [16'][15]

To a solution of 100 mg (152 µmol, 1.0 eq.) of tripeptide **15** in 1.5 mL of anhydrous MeOH, 4.80 µL (67.5 µmol, 0.45 eq., ρ = 1.104 g/mL) acetyl chloride was added under an Ar atmosphere at 0 °C. The colorless solution was slowly warmed to rt and after 17 h diluted with EtOAc. Subsequently, it was washed with saturated $NaHCO_{3(aq.)}$ solution and water before the organic phase was dried over $MgSO_4$ and concentrated in vacuo. After automated column chromatography (C_{18}-SiO_2, H_2O/MeCN 9:1 ⟶ 0:10) and subsequent lyophilization of the residue, 76.0 mg (139 µmol, 92%) of the alcohol **16'** was isolated as a colorless lyophilizate.

$[\alpha]_D^{20}$ = −108.4 (c = 0.5, $CHCl_3$) **R$_f$** = 0.12 (*n*-Pentane/EtOAc 1:1)

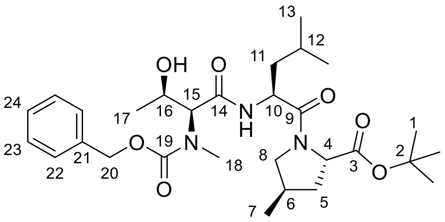

Main rotamer:

^{1}H NMR (500 MHz, $CDCl_3$): δ = 7.29−7.40 (m, 5 H, 22-H, 23-H, 24-H), 6.67 (d, $^3J_{NH,10}$ = 8.7 Hz, 1 H, NH), 5.17 (m, 2 H, 20-H), 4.76 (m, 1 H, 10-H), 4.64 (m, 1 H, 15-H), 4.33−4.47 (m, 2 H, 4-H, 16-H), 3.71 (t, $^2J_{8a,8b} \approx {}^3J_{8a,6}$ = 8.5 Hz, 1 H, 8-H$_a$), 3.25 (t, $^2J_{8b,8a} \approx {}^3J_{8b,6}$ = 8.9 Hz, 1 H, 8-H$_b$), 3.02 (s, 3 H, 18-H), 2.49 (m, 1 H, 6-H), 2.06 (ddd, $^2J_{5a,5b}$ = 12.9 Hz, $^3J_{5a,6}$ = 6.4 Hz, $^3J_{5a,4}$ = 3.0 Hz, 1 H, 5-H$_a$), 1.80 (dt, $^2J_{5b,5a}$ = 12.8 Hz, $^3J_{5b,4} \approx {}^3J_{5b,6}$ = 9.3 Hz, 1 H, 5-H$_b$), 1.62 (m, 1 H, 12-H), 1.52 (m, 2 H, 11-H), 1.45 (s, 9 H, 1-H), 1.18 (d, $^3J_{17,16}$ = 6.4 Hz, 3 H, 17-H), 1.08 (d, $^3J_{7,6}$ = 6.6 Hz, 3 H, 7-H), 0.98 (d, $^3J_{13,12}$ = 6.4 Hz, 3 H, 13-H), 0.92 (d, $^3J_{13',12}$ = 6.6 Hz, 3 H, 13-H'),

^{13}C-NMR (126 MHz, $CDCl_3$): δ = 171.0 (s, C-3), 170.7 (s, C-9), 170.4 (s, C-14), 157.8 (s, C-19), 136.5 (s, C-21), 128.7 (d, C-23), 128.3 (d, C-24), 127.9 (d, C-22), 81.6 (s, C-2), 68.0 (t, C-20), 67.2 (d, C-16), 63.0 (d, C-15), 59.8 (d, C-4), 53.6 (t, C-8), 49.0 (d, C-10), 41.7 (t, C-11), 36.8 (t, C-5), 32.9 (d, C-6), 32.7 (q, C-18), 28.1 (q, C-1), 25.0 (d, C-12), 23.5 (q, C-13), 21.8 (q, C-13'), 18.9 (q, C-17), 17.5 (q, C-7).

Minor rotamer (selected signals):

^{1}H NMR (500 MHz, $CDCl_3$): δ = 6.48 (d, $^3J_{NH,10}$ = 9.0 Hz, 1 H, NH), 4.53 (bs, 1 H, 15-H), 3.83 (dd, $^2J_{8a,8b}$ = 11.6 Hz, $^3J_{8a,6}$ = 7.9, 1 H, 8-H$_a$), 1.46 (s, 9 H, 1-H), 0.89 (d, $^3J_{13,12}$ = 6.6 Hz, 3 H, 13-H).

^{13}C-NMR (126 MHz, $CDCl_3$): δ = 156.5 (s, C-19), 136.2 (s, C-21), 83.1 (s, C-2), 68.2 (t, C-20), 62.9 (d, C-15), 60.3 (d, C-4), 53.5 (t, C-8), 43.6 (t, C-11), 39.3 (t, C-5), 33.1 (q, C-18), 30.1 (d, C-6), 28.0 (q, C-1), 24.9 (d, C-12), 23.6 (q, C-13'), 21.6 (q, C-13), 18.6 (q, C-17), 17.6 (q, C-7).

HRMS (ESI): Calculated for $C_{29}H_{46}N_3O_7^+$ $[M+H]^+$: 548.3330, found: 548.3308.

Cbz-*O*-(EtPro-Cbz)-*N*-Me-L-Thr-L-Leu-L-MePro-O*t*-Bu [16]

A solution of 127 mg (231 µmol, 1.0 eq.) of tripeptide **16'**and 88.4 mg (319 µmol, 1.4 eq.) Cbz-L-EtPro-OH (**13'**) in 1.2 mL anhydrous THF was treated with 43.6 mg (294 µmol, 1.3 eq.) PPY, followed by 112 mg (326 µmol, 1.4 eq) MNBA under Ar atmosphere at 0 °C.

After addition, the reaction mixture was slowly warmed to rt overnight (15.5 h) and then concentrated in vacuo. Finally, the resulting residue was purified by automated column chromatography (SiO_2, cHex/EtOAc 10:0 ⟶ 4:6). Lyophilization yielded 179 mg (222 µmol, 96%) of the depsipeptide **16** as a colorless lyophilizate.

$[\alpha]_D^{20}$ = −79.4 (c = 0.5, $CHCl_3$)

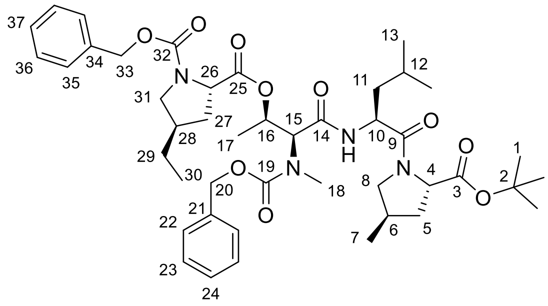

Main rotamer:

^{1}H-NMR (400 MHz, $CDCl_3$): δ = 7.18−7.43 (m, 10 H, 22-H, 23-H, 24-H, 35-H, 36-H, 37-H), 6.59 (m, 1 H, NH), 5.42 (m, 1 H, 16-H), 5.15 (m, 2 H, 20-H), 5.08 (m, 2 H, 33-H), 4.50−4.81 (m, 2 H, 10-H, 15-H), 4.43 (m, 1 H, 4-H), 4.29 (m, 1 H, 26-H), 3.57−3.73 (m, 2 H, 8-H_a, 31-H_a), 3.24 (m, 1 H, 8-H_b), 3.02 (m, 1 H, 31-H_b), 2.86 (s, 3 H, 18-H), 2.48 (m, 1 H, 6-H), 1.98−2.20 (m, 3 H, 5-H_a, 27-H_a, 28-H), 1.74−1.87 (m, 2 H, 5-H_b, 27-H_a), 1.45 (s, 9 H, 1-H), 1.42−1.63 (m, 3 H, 11-H, 12-H), 1.35 (m, 2 H, 29-H), 1.24 (d, $^3J_{17,16}$ = 6.3 Hz, 3 H, 17-H), 1.07 (d, $^3J_{7,6}$ = 6.6 Hz, 3 H, 7-H), 0.93 (d, $^3J_{13,12}$ = 6.2 Hz, 3 H, 13-H), 0.83−0.91 (m, 6 H, 13-H', 30-H).

^{13}C-NMR (126 MHz, $CDCl_3$): δ = 171.6 (s, C-25), 171.1 (s, C-3), 170.5 (s, C-9), 167.8 (s, C-14), 157.5 (s, C-19), 154.8 (s, C-32), 136.9 (s, C-34), 136.4 (s, C-21), 128.6 (d, C-36), 128.5 (d, C-23), 128.4 (d, C-24), 128.3 (d, C-37), 128.1 (d, C-35), 128.0 (d, C-22), 81.5 (s, C-2), 68.3 (d, C-16), 68.0 (t, C-20), 67.1 (t, C-33), 62.1 (d, C-15), 59.8 (d, C-4), 59.2 (d, C-26), 53.6 (t, C-8), 52.3 (t, C-31), 49.0 (d, C-10), 41.6 (t, C-11), 38.1 (d, C-28), 36.8 (t, C-5), 35.6 (t, C-27), 32.6 (d, C-6), 30.4 (q, C-18), 28.1 (q, C-1), 26.1 (t, C-29), 24.9 (d, C-12), 23.5 (q, C-13), 21.8 (q, C-13'), 17.7 (q, C-17), 17.5 (q, C-7), 12.5 (q, C-30).

Minor rotamer (selected signals):

^{1}H-NMR (400 MHz, $CDCl_3$): δ = 6.39 (d, $^3J_{NH,10}$ = 8.7 Hz, 1 H, NH), 6.35 (d, $^3J_{NH,10}$ = 8.6 Hz, 1 H, NH), 2.73 (s, 3 H, 18-H), 2.67 (s, 3 H, 18-H), 1.19 (d, $^3J_{17,16}$ = 6.3 Hz, 3 H, 17-H), 1.11 (d, $^3J_{17,16}$ = 6.1 Hz, 3 H, 17-H), 0.94 (d, $^3J_{13',12}$ = 6.1 Hz, 3 H, 13-H).

^{13}C-NMR (126 MHz, $CDCl_3$): δ = 171.7 (s, C-25), 168.0 (s, C-14), 157.3 (s, C-19), 154.3 (s, C-32), 62.2 (d, C-15), 59.6 (d, C-26), 51.8 (t, C-31), 39.2 (d, C-28), 36.6 (t, C-5), 30.8 (q, C-18), 28.0 (q, C-1), 21.6 (q, C-13).

HRMS (ESI): Calculated for $C_{44}H_{63}N_4O_{10}^+$ $[M+H]^+$: 807.4539, found: 807.4513.

Alloc-*N*-Me-L-Val-L-EtPro-*N*-Me-L-Thr-L-Leu-L-MePro-O*t*-Bu [17]

According to **GP2**, 1.38 g (1.71 mmol, 1.0 eq.) of the depsipeptide **16** was hydrogenated with 132 mg (10 wt%) Pd/C in 10 mL MeOH for 6 h. The resulting amine was then coupled according to **GP4** with 917 mg (4.26 mmol, 2.5 eq.) Alloc-*N*-Me-L-Val-OH (**S4**), 2.22 g (4.26 mmol, 2.5 eq.) PyAOP, and 658 µL (5.98 mmol, 3.5 eq., ρ = 0.920 g/mL) NMM in 5.8 mL anhydrous DMF for 15 h. Automated column chromatography (SiO_2, cHex/EtOAc 1:0 ⟶ 0:1) yielded 1.10 g (1.50 mmol, 88%) of the pentapeptide **17** as a colorless foam.

$[\alpha]_D^{20}$ = −136.6 (c = 0.5, $CHCl_3$) **R_f** = 0.04 (*n*-Pentane/EtOAc 1:1)

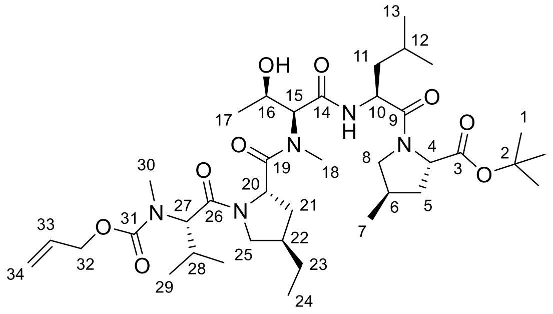

Main rotamer:

^{1}H-NMR (500 MHz, DMSO-*d*6, 373 K): δ = 8.26 (bs, 1 H, NH), 5.96 (ddd, $^3J_{33,34a}$ =17.4 Hz, $^3J_{33,34b}$ =10.8 Hz, $^3J_{33,32}$ =5.3 Hz, 1 H, 33-H), 5.31 (dd, $^3J_{34a,33}$ = 17.3 Hz, $^4J_{34a,32}$ = 1.9 Hz, 1 H, 34-H_a), 5.22 (dd, $^3J_{34b,33}$ = 10.5 Hz, $^4J_{34b,32}$ = 1.7 Hz, 1 H, 34-H_b), 4.99 (bs, 1 H, 20-H), 4.59 (dt, $^3J_{32,33}$ = 5.4 Hz, $^4J_{32,34}$ = 1.6 Hz, 2 H, 32-H), 4.46–4.57 (m, 2 H, 10-H, 27-H), 4.40 (d, $^3J_{15.16}$ =8.4 Hz, 1 H, 15-H), 4.26 (m, 1 H, 4-H), 4.06 (m, 1 H, 16-H), 3.82 (m, 1 H, 25-H_a), 3.69 (m, 1 H, 8-H_a), 3.23–3.48 (m, 2 H, 8-H_b, 25-H_b), 2.87 (s, 3 H, 30-H), 2.77 (s, 3 H, 18-H), 2.44 (m, 2 H, 6-H, 22-H), 2.18 (m, 1 H, 28-H), 2.04 (m, 1 H, 21-H_a), 1.97 (m, 1 H, 5-H_a), 1.76–1.85 (m, 2 H, 5-H_b, 21-H_b), 1.70 (m, 1 H, 12-H), 1.61 (m, 1 H, 11-H_a), 1.51 (m, 1 H, 11-H_b), 1.40 (s, 9 H, 1-H), 1.35 (quint, $^3J_{23,22} \approx {}^3J_{23,24}$ = 7.4 Hz, 2 H, 23-H), 1.14 (m, 3 H, 17-H), 1.04 (d, $^3J_{7,6}$ = 6.7 Hz, 3 H, 7-H), 0.87–0.94 (m, 12 H, 13-H, 24-H, 29-H), 0.85 (d, $^3J_{29,28}$ = 6.7 Hz, 3 H, 29-H).

^{13}C-NMR (126 MHz, DMSO-*d*6, 373 K): δ = 172.0 (s, C-19), 170.3 (s, C-3), 169.5 (s, C-9), 168.1 (s, C-14), 168.0 (s, C-26), 155.5 (s, C-31), 132.9 (d, C-33), 116.5 (t, C-34), 79.7 (s, C-2), 65.1 (t, C-32), 64.6 (d, C-15), 62.6 (d, C-16), 60.6 (d, C-27), 58.9 (d, C-4), 55.0 (d, C-20), 52.7 (t, C-8), 51.9 (t, C-25), 48.3 (d, C-10), 38.7 (d, C-22), 35.6 (t, C-5), 34.2 (t, C-21), 31.6 (d, C-6), 28.7 (q, C-30),

28.4 (q, C-18), 27.1 (q, C-1), 26.8 (d, C-28), 25.1 (t, C-23), 23.5 (d, C-12), 22.4 (q, C-13'), 20.8 (q, C-13), 20.0 (q, C-17), 18.2 (q, C-29'), 17.8 (q, C-29), 16.8 (q, C-7), 11.3 (q, C-24).

Minor rotamer (selected signals):

^{1}H-NMR (500 MHz, DMSO-*d*$_6$, 373 K): δ = 7.49 (bs, 1 H, NH), 4.88 (bs, 1 H, 20-H), 4.35 (m, 1 H, 15-H), 4.20 (m, 1 H, 16-H), 3.13 (s, 3 H, 18-H), 2.84 (s, 3 H, 30-H), 2.33 (s, 1 H, 22-H).

^{13}C-NMR (126 MHz, DMSO-*d*$_6$, 373 K): δ = 64.4 (d, C-16), 56.1 (d, C-20), 23.7 (d, C-12), 21.1 (q, C-13), 19.7 (q, C-17), 18.5 (q, C-29).

The signal of C-11 lies beneath the solvent peak.

HRMS (CI): Calculated for $C_{38}H_{66}N_5O_9^+$ [M+H]$^+$: 736.4855, found: 736.4831.

Alloc-*N*-Me-L-Val-L-EtPro-*O*-(Gly-Fmoc)-*N*-Me-L-Thr-L-Leu-L-MePro-O*t*-Bu [18]

A solution of 95.2 mg (129 µmol, 1.0 eq.) of alcohol **17** and 78.3 mg (263 µmol, 2.0 eq.) Fmoc-Gly-OH in 1.3 mL anhydrous THF was treated with 21.6 mg (145 µmol, 1.1 eq.) PPY, followed by 112 mg (326 µmol, 1.4 eq) MNBA under Ar atmosphere at rt.

After 15 h the mixture was concentrated in vacuo. Automated column chromatography (C_{18}-SiO_2; H_2O/MeCN 9:1 ⟶ 0:10) of the obtained residue and lyophilization yielded 126 mg (124 µmol, 96%) of the depsipeptide **18** as a colorless lyophilizate.

$[\alpha]_D^{20}$ = −114.2 (c = 0.5, $CHCl_3$) **R$_f$** = 0.04 (*n*-Pentane/EtOAc 1:1)

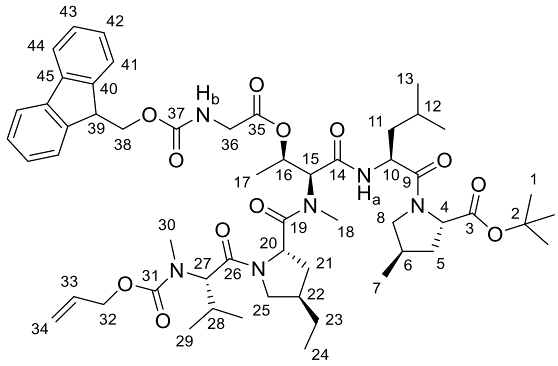

Main rotamer:

^{1}H-NMR (400 MHz, $CDCl_3$): δ = 7.75 (d, $^3J_{44,43}$ = 7.5 Hz, 2 H, 44-H), 7.59 (d, $^3J_{41,42}$ = 7.2 Hz, 2 H, 41-H), 7.39 (m, 2 H, 43-H), 7.30 (m, 2 H, 42-H), 6.71 (dd, $^3J_{NHb,36b}$ = 7.5 Hz, $^3J_{NHb,36b}$ = 5.3 Hz, 1 H, NH$_b$), 6.42 (d, $^3J_{NHa,10}$ = 8.8 Hz, 1 H, NH$_a$), 5.93 (m, 1 H, 33-H), 5.53 (m, 1 H, 16-H), 5.27 (m, 2 H, 34-H), 5.18 (m, 1 H, 15-H), 4.67−4.87 (m, 2 H, 10-H, 20-H), 4.53−4.66 (m, 3 H, 27-H, 32-H), 4.45 (dd, $^3J_{4,5a/b}$ = 8.9 Hz, $^3J_{4,5b/a}$ = 3.0 Hz, 1 H, 4-H), 4.35 (m, 2 H, 38-H), 4.25 (t, $^3J_{39,38}$ = 7.5 Hz, 1 H, 39-H), 4.07 (dd, $^2J_{36a,36b}$ =17.9 Hz, $^3J_{36a,NHb}$ = 7.3 Hz, 1 H, 36-H$_a$), 3.95 (m, 1 H, 25-H$_a$), 3.82 (m, 1 H, 36-H$_b$), 3.67 (m, 1 H, 8-H$_a$), 3.51 (dd, $^2J_{25b,25a}$ = 9.8 Hz, $^3J_{25b,22}$ = 8.0 Hz, 1 H, 25-H$_b$), 3.23 (t, $^2J_{8b,8a} \approx {}^3J_{8b,6}$ =9.0 Hz, 1 H, 8-H$_b$), 3.03 (s, 3 H, 18-H), 2.91 (s, 3 H, 30-H), 2.35−2.62 (m, 2 H, 6-H, 22-H), 2.22 (m, 1 H, 28-H), 2.05 (m, 1 H, 5-H$_a$), 1.73−1.88 (m, 3 H, 5-H$_b$, 21-H), 1.46−1.59 (m, 3 H, 11-H, 12-H), 1.44 (s, 9 H, 1-H), 1.36 (m, 2 H, 23-H), 1.28 (d, $^3J_{17,16}$ =

6.3 Hz, 3 H, 17-H), 1.07 (d, $^3J_{7,6}$ = 6.6 Hz, 3 H, 7-H), 0.82–1.00 (m, 15 H, 13-H, 13-H', 24-H, 29-H, 29-H').

^{13}C-NMR (126 MHz, $CDCl_3$): δ = 172.5 (s, C-19), 171.5 (s, C-3), 171.0 (s, C-9), 170.2 (s, C-26), 170.0 (s, C-35), 167.7 (s, C-14), 157.2 (s, C-37), 157.1 (s, C-31), 144.2 (s, C-40), 141.4 (d, C-45), 133.0 (d, C-33), 127.8 (d, C-43), 127.2 (d, C-42), 125.3 (d, C-41), 120.0 (d, C-44), 117.4 (t, C-34), 81.6 (s, C-2), 67.2 (t, C-38), 67.1 (d, C-16), 66.4 (t, C-32), 61.3 (d, C-27), 60.1 (d, C-15), 59.8 (d, C-4), 57.4 (d, C-20), 53.6 (t, C-8), 52.8 (t, C-25), 48.8 (d, C-10), 47.3 (d, C-39), 43.2 (t, C-36), 42.9 (t, C-36), 39.4 (d, C-22), 36.8 (t, C-5), 34.2 (t, C-21), 32.6 (d, C-6), 31.2 (q, C-18), 29.7 (q, C-30), 29.5 (q, C-30), 28.1 (q, C-1), 27.9 (d, C-28), 26.1 (t, C-23), 25.0 (d, C-12), 23.6 (q, C-13'), 21.7 (q, C-13), 19.1 (q, C-29'), 18.9 (q, C-29), 17.5 (q, C-7, C-17), 12.5 (q, C-24).

Minor rotamer (selecetd signals):

^{1}H-NMR (400 MHz, $CDCl_3$): δ = 7.76 (d, $^3J_{44,43}$ = 7.5 Hz, 2 H, 44-H), 7.61 (d, $^3J_{41,42}$ = 7.2 Hz, 2 H, 41-H), 6.62 (m, 1 H, NH_b), 6.56 (d, $^3J_{NHa,10}$ = 8.9 Hz, 1 H, NH_a), 2.95 (s, 3 H, 30-H), 2.89 (s, 3 H, 30-H), 2.75 (s, 3 H, 18-H), 1.42 (s, 9 H, 1-H), 1.01 (d, $^3J_{29,28}$ = 6.6 Hz, 3 H, 29-H).

^{13}C-NMR (126 MHz, $CDCl_3$): δ = 173.5 (s, C-19), 132.8 (d, C-33), 127.9 (d, C-43), 127.1 (d, C-42), 125.2 (d, C-41), 120.1 (d, C-44), 117.3 (t, C-34), 81.1 (s, C-2), 68.6 (d, C-16), 67.4 (t, C-38), 66.7 (t, C-32), 61.2 (d, C-27), 60.2 (d, C-15), 59.9 (d, C-4), 57.1 (d, C-20), 53.5 (t, C-8), 52.7 (t, C-25), 49.5 (d, C-10), 47.2 (d, C-39), 41.6 (t, C-11), 39.3 (d, C-22), 36.7 (t, C-5), 32.8 (d, C-6), 31.3 (q, C-18), 29.9 (q, C-18), 26.2 (t, C-23), 24.7 (d, C-12), 23.7 (q, C-13'), 21.2 (q, C-13).

HRMS (ESI): Calculated for $C_{55}H_{79}N_6O_{12}^+$ $[M+H]^+$: 1015.5750, found: 1015.5733.

Synthesis of the Tetrapeptide Fragment

Boc-L-Pro-*N*-Me-D-Leu-OMe [20][14]

Under N_2 atmosphere, 3.09 g (14.37 mmol, 1.4 eq.) of Boc-L-Pro-OH was dissolved in 51 mL 1,4-dioxane and treated with 3.93 mL (22.56 mmol, 2.2 eq., ρ = 0.742 g/mL) DIPEA, followed by 152 µL (1.02 mmol, 0.1 eq., ρ = 0.910 g/mL) $BnNMe_2$. The solution was heated in an oil bath to 60 °C and treated dropwise with 14.3 mL (1.0 M in toluene, 14.30 mmol, 1.4 eq.) IPCF solution. After stirring at 60 °C for 15 min, a solution of 2.00 g (10.22 mmol, 1.0 eq.) *N*-Me-D-Leu-OMe·HCl, 163 µL (2.05 mmol, 0.2 eq., ρ = 1.030 g/mL) NMI, and 256 µL (4.0 M in 1,4-dioxane, 1.02 mmol, 0.1 eq.) HCl, dissolved in a mixture of 26 mL 1,4-dioxane and 42 mL MeCN, was added (rinsed twice with a total of 26 mL 1,4-dioxane). Stirring was continued at 60 °C for 17 h, and then the mixture was cooled to rt.

After concentration in vacuo, the residue was diluted with EtOAc. The organic phase was washed with 1 M $KHSO_{4(aq.)}$, water, sat. $NaHCO_{3(aq.)}$ and sat. $NaCl_{(aq.)}$ solution. Then it was dried over $MgSO_4$, the solvent was removed in vacuo, and the residue was purified by automated column chromatography (cHex/EtOAc 10:0 ⟶ 4:6). A total of 3.02 g (8.48 mmol, 84%) of dipeptide **20** was isolated as a colorless solid.

$[\alpha]_D^{20}$ = +12.4 (c = 1.0, $CHCl_3$) **Melting range**: 119–120 °C

R_f = 0.30 (*n*-Pentane/EtOAc 1:1)

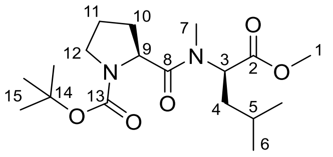

¹H-NMR (500 MHz, DMSO-d_6, 373 K): δ = 4.91 (bs, 1 H, 3-H), 4.62 (bs, 1 H, 9-H), 3.64 (s, 3 H, 1-H), 3.36 (m, 3 H, 12-H), 2.97 (s, 3 H, 7-H), 2.22 (m, 1 H, 10-H_a), 1.76–1.88 (m, 2 H, 11-H), 1.63–1.76 (m, 3 H, 4-H, 10-H_b), 1.49 (m, 1 H, 5-H), 1.36 (s, 9 H, 15-H), 0.92 (d, $^3J_{6,5}$ = 6.6 Hz, 3 H, 6-H), 0.88 (d, $^3J_{6',5}$ = 6.9 Hz, 3 H, 6-H′),

¹³C-NMR (126 MHz, DMSO-d_6, 373 K): δ = 173.2 (s, C-8), 171.9 (s, C-2), 153.8 (s, C-13), 78.9 (s, C-14), 57.3 (d, C-9), 55.8 (d, C-3), 52.0 (q, C-1), 46.9 (t, C-12), 38.0 (t, C-4), 32.2 (q, C-7), 30.1 (t, C-10), 28.5 (q, C-15), 25.0 (d, C-5), 23.3 (q, C-6; t C-11), 21.8 (q, C-6).

HRMS (CI): Calculated for $C_{18}H_{32}N_2O_5^+$ $[M]^+$: 356.2311, found: 356.2306.

Boc-*N*-Me-L-Val-L-Pro-*N*-Me-D-Leu-OMe [21]

According to **GP3**, 1.52 g (4.26 mmol, 1.0 eq.) of dipeptide **20** was reacted with 2.30 mL (31.70 mmol, 7.5 eq., ρ = 1.104 g/mL) acetyl chloride in 11 mL MeOH for 1.5 h. Contrary to **GP3**, **20** was added only after acetyl chloride. The resulting peptide hydrochloride was reacted according to **GP4** with 985 mg (4.26 mmol, 1.0 eq.) Boc-*N*-Me-L-Val-OH, 1.51 g (4.70 mmol, 1.1 eq.) TBTU, and 1.64 mL (9.39 mmol, 2.2 eq., ρ = 0.742 g/mL) DIPEA in 29 mL anhydrous MeCN for 17 h. Afterwards, the mixture was concentrated in vacuo, the residue was taken up in EtOAc, and the following workup was performed accordingly. Instead of 1 M $KHSO_{4(aq.)}$, 1 M $HCl_{(aq.)}$ was used in the first washing step. Automated column chromatography (SiO_2, DCM/MeOH 100:0 ⟶ 97:3) of the crude product yielded 1.39 g (2.96 mmol, 70%) of tripeptide **21** as a colorless resin.

$[\alpha]_D^{20}$ = −76.0 (c = 1.0, $CHCl_3$) **R_f** = 0.47 (DCM/MeOH 95:5)

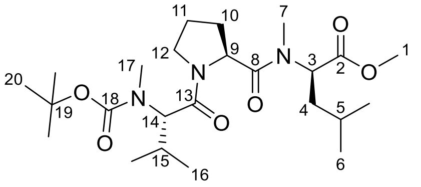

¹H-NMR (500 MHz, DMSO-d_6, 373 K): δ = 4.71–4.85 (m, 2 H, 3-H, 9-H), 4.42 (bs, 1 H, 14-H), 3.64 (s, 3 H, 1-H), 3.63 (s, 3 H, 7-H), 3.54–3.70 (m, 2 H, 12-H), 2.73 (s, 3 H, 17-H), 2.11–2.24 (m, 2 H, 10-H_a, 15-H), 1.97 (m, 1 H, 11-H_a), 1.87 (m, 1 H, 11-H_b), 1.62–1.80 (m, 3 H, 4-H, 10-H_b), 1.53 (m, 1 H, 5-H), 1.44 (s, 9 H, 20-H), 0.89 (d, $^3J_{16,15}$ = 6.4 Hz, 3 H, 16-H), 0.85–0.95 (m, 6 H, 6-H), 0.81 (d, $^3J_{16',15}$ = 6.7 Hz, 3 H, 16-H').

¹³C-NMR (126 MHz, DMSO-d_6, 373 K): δ = 171.4 (s, C-8), 170.9 (s, C-2), 167.5 (s, C-13), 154.8 (s, C-18ff), 78.8 (s, C-19), 60.6 (d, C-14), 56.1 (d, C-9), 55.5 (d, C-3), 51.0 (q, C-1), 46.2 (t, C-12), 37.0 (t, C-4), 32.0 (q, C-7), 28.6 (q, C-17), 27.8 (t, C-10), 27.6 (q, C-20), 26.6 (d, C-15), 24.0 (d, C-5), 23.6 (t, C-11), 22.3 (q, C-6'), 20.9 (q, C-6), 18.6 (q, C-16'), 17.7 (q, C-16).

HRMS (CI): Calculated for $C_{24}H_{44}N_3O_6^+$ $[M+H]^+$: 470.3225, found: 470.3214.

Boc-L-HoLeu-*N*-Me-L-Val-L-Pro-*N*-Me-D-Leu-OMe [22']

According to **GP3**, 1.34 g (2.85 mmol, 1.0 eq.) of tripeptide **21** was reacted with 2.1 mL (28.94 mmol, 10 eq., ρ = 1.104 g/mL) acetyl chloride and 1.15 mL (28.5 mmol, 10 eq., ρ = 0.792 g/mL) MeOH in 7.1 mL 1,4-dioxane for 4 h. The resulting peptide hydrochloride was reacted according to **GP4** with 826 mg (3.37 mmol, 1.2 eq.) Boc-L-HoLeu-OH, 387 mg (2.85 mmol, 1.0 eq.) HOAt, 2.16 g (5.69 mmol, 2.0 eq.) HATU, and 1.98 mL (11.38 mmol, 4.0 eq., ρ = 0.742 g/mL) DIPEA in 15 mL anhydrous DMF for 15 h. Automated column chromatography (SiO_2, cHex/EtOAc 10:0 ⟶ 4:6) of the crude product yielded 1.28 g (2.15 mmol, 76%) of the tetrapeptide **22'** as a colorless resin.

$[\alpha]_D^{20}$ = −85.9 (c = 1.0, $CHCl_3$) **R_f** = 0.25 (*n*-Pentane/EtOAc 1:1)

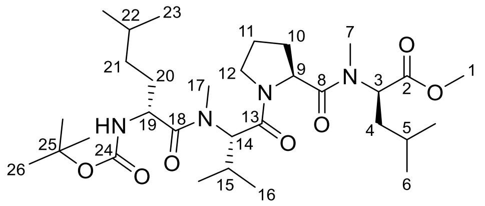

¹H-NMR (500 MHz, DMSO-*d*₆, 373 K): δ = 6.33 (bs, 1 H, NH), 4.94 (d, $^3J_{14,15}$ = 10.8 Hz, 1 H, 14-H), 4.77 (bs, 2 H, 3-H, 9-H), 4.39 (q, $^3J_{19,NH}$ ≈ $^3J_{19,20}$ = 7.5 Hz, 1 H, 19-H), 3.67 (m, 1 H, 12-H_a), 3.64 (s, 3 H, 1-H), 3.53 (q, $^2J_{12b,12a}$ ≈ $^3J_{12b,11}$ = 7.4 Hz, 1 H, 12-H_b), 3.03 (s, 3 H, 7-H), 2.99 (s, 3 H, 17-H), 2.08–2.25 (m, 2 H, 10-H_a, 15-H), 1.96 (m, 1 H, 11-H_a), 1.64–1.84 (m, 4 H, 4-H, 10-H_b, 11-H_b), 1.46–1.63 (m, 4 H, 5-H, 20-H, 22-H), 1.39 (s, 9 H, 26-H), 1.19 (m, 2 H, 21-H), 0.90 (d, $^3J_{16',15}$ = 6.5 Hz, 3 H, 16-H'), 0.87–0.97 (m, 6 H, 6-H), 0.86 (d, $^3J_{23,22}$ = 6.6 Hz, 6 H, 23-H), 0.78 (d, $^3J_{16,15}$ = 6.7 Hz, 3 H, 16-H).

¹³C-NMR (126 MHz, DMSO-*d*₆, 373 K): δ = 172.5 (s, C-18), 171.4 (s, C-8), 170.9 (s, C-2), 167.3 (s, C-13), 154.6 (s, C-24), 77.7 (s, C-25), 58.7 (d, C-14), 56.1 (d, C-9), 55.5 (d, C-3), 51.0 (q, C-1), 50.5 (d, C-19), 46.3 (t, C-12), 37.0 (t, C-4), 33.7 (t, C-21), 31.9 (q, C-7), 29.4 (q, C-17), 28.8 (t, C-20), 27.8 (t, C-10), 27.7 (q, C-26), 26.7 (d, C-22), 26.2 (d, C-15), 24.0 (d, C-5), 23.7 (t, C-11), 22.3 (q, C-6), 21.8 (q, C-23), 21.6 (q, C-23'), 20.9 (q, C-6'), 18.6 (q, C-16), 17.6 (q, C-16').

HRMS (CI): Calculated for $C_{31}H_{57}N_4O_7^+$ $[M]^+$: 597.4222, found: 597.4234.

Boc-L-HoLeu-*N*-Me-L-Val-L-Pro-*N*-Me-D-Leu-OH [22]

102 mg (170 µmol, 1.0 eq.) of tetrapeptide **22'** was dissolved in 0.85 mL 1,4-dioxane and treated with 188 µL (1.0 M, 188 µmol, 1.1 eq.) of freshly prepared $LiOH_{(aq.)}$ solution. After 3 h, the mixture was acidified with 1 M $HCl_{(aq.)}$ (pH = 1) and extracted three times with EtOAc. Next the combined organic phases were dried over $MgSO_4$ and the solvent was removed in vacuo. Lyophilization of the residue yielded 94.4 mg (162 µmol, 95%) of carboxylic acid **22** as a colorless lyophilizate.

$[\alpha]_D^{20}$ = −75.8 (c = 1.0, $CHCl_3$)

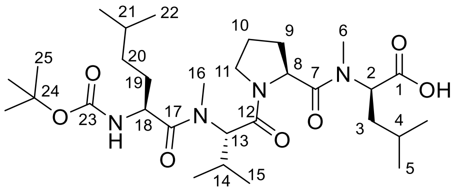

Main rotamer:

¹H-NMR (400 MHz, DMSO-d_6): δ = 12.65 (bs, 1 H, COOH), 7.02 (d, $^3J_{NH,18}$ = 8.1 Hz, 1 H, NH), 4.90 (d, $^3J_{13,14}$ = 10.9 Hz, 1 H, 13-H), 4.84 (dd, $^3J_{2,3a/b}$ = 11.3 Hz, $^3J_{2,3b/a}$ = 4.4 Hz, 1 H, 2-H), 4.76 (dd, $^3J_{8,9a/b}$ = 8.5 Hz, $^3J_{8,9b/a}$ = 4.7 Hz, 1 H, 8-H), 4.30 (q, $^3J_{18,NH}$ ≈ $^3J_{18,19}$ =7.5 Hz, 1 H, 18-H), 3.62 (m, 1 H, 11-H), 3.49 (m, 1 H, 11-H'), 2.98 (s, 3 H, 16-H), 2.96 (s, 3 H, 6-H), 2.14 (m, 2 H, 9-H_a, 14-H), 1.86 (m, 1 H, 10-H_a), 1.74 (m, 2 H, 3-H_a, 10-H_b), 1.63 (m, 1 H, 9-H_b), 1.38–1.60 (m, 5 H, 3-H_b, 4-H, 19-H_a, 19-H_b, 21-H), 1.35 (s, 9 H, 25-H), 1.18 (m, 1 H, 20-H_a), 1.06 (m, 1 H, 20-H_b), 0.87 (m, 9 H, 5-H, 22-H, 22-H'), 0.72 (d, $^3J_{15,14}$ = 6.6 Hz, 3 H, 15-H).

¹³C-NMR (101 MHz, DMSO-d_6): δ = 173.3 (s, C-17), 172.9 (s, C-1), 171.5 (s, C-7), 167.6 (s, C-12), 155.5 (s, C-23), 77.9 (s, C-24), 58.7 (d, C-13), 56.8 (d, C-8), 54.7 (d, C-2), 50.6 (d, C-18), 46.8 (t, C-11), 37.2 (t, C-3), 34.4 (t, C-20), 31.7 (q, C-6), 29.8 (q, C-16), 29.0 (t, C-19), 28.4 (t, C-9), 28.1 (q, C-25), 27.3 (d, C-21), 26.6 (d, C-14), 24.5 (d, C-4), 24.2 (t, C-10), 23.3 (q, C-5), 22.6 (q, C-22), 22.4 (q, C-22'), 21.1 (q, C-5'), 19.2 (q, C-15), 18.0 (q, C-15').

Minor rotamer (selected signals):

¹H-NMR (400 MHz, DMSO-d_6): δ = 6.61 (d, $^3J_{NH,18}$ = 8.3 Hz, 1 H, NH), 4.71 (dd, $^3J_{8,9a/b}$ = 8.6 Hz, $^3J_{8,9b/a}$ = 4.6 Hz, 1 H, 8-H), 4.57 (dd, $^3J_{2,3a/b}$ = 9.5 Hz, $^3J_{2,3b/a}$ = 5.1 Hz, 1 H, 2-H), 2.66 (s, 1 H, 16-H).

HRMS (ESI): Calculated for $C_{30}H_{55}N_4O_7^+$ $[M+H]^+$: 583.4065, found: 583.4074.

Synthesis of Mycoplanecin A: Fragment Coupling, Cyclization & Acylation

Alloc-*N*-Me-L-Val-L-EtPro-*O*-(Gly-*N*-Me-D-Leu-L-Pro-*N*-Me-L-Val-L-HoLeu-Boc)-*N*-Me-L-Thr-L-Leu-L-MePro-O*t*-Bu [23]

A solution of 426 mg (420 µmol, 1.0 eq.) of hexapeptide **18** in 4.2 mL DCM was treated with 630 µL (4.20 mmol, 10 eq., ρ = 0.976 g/mL) tren (10 eq.) at rt and stirred for 50 min. Subsequently, the now colorless suspension was diluted with EtOAc and washed twice with water and twice with a phosphate buffer (pH = 5.5). Afterwards, the organic phase was dried over $MgSO_4$ and the solvent was removed in vacuo.

The resulting free amine was then coupled according to **GP4** with 294 mg (505 µmol, 1.2 eq.) of carboxylic acid **22**, 218 mg (509 µmol, 1.2 eq.) COMU, and 115 µL (1.05 mmol, 2.5 eq., ρ = 0.920 g/mL) NMM in 4.2 mL anhydrous DMF for 25 h. After automated column chromatography (C_{18}-SiO_2, H_2O/MeCN 9:1 ⟶ 0:10), 461 mg (340 µmol, 81%) of decapeptide **23** was isolated as a colorless foam.

$[\alpha]_D^{20} = -100.0$ (c = 0.5, $CHCl_3$)

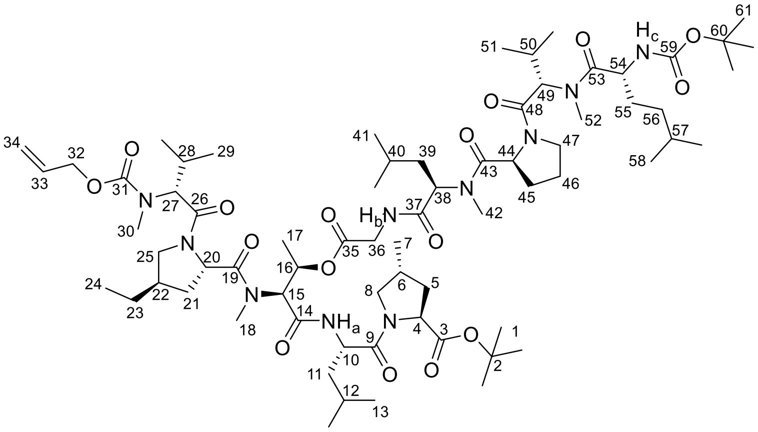

^{1}H-NMR (500 MHz, $CDCl_3$): δ = 8.82 (d, $^3J_{NHa,10}$ = 8.1 Hz, 1 H, NH_a), 7.63 (t, $^3J_{NHb,36}$ = 6.1 Hz, 1 H, NH_b), 5.92 (m, 1 H, 33-H), 5.46 (m, 1 H, 16-H), 5.35 (dd, $^3J_{38,39a/b}$ = 11.3 Hz, $^3J_{38,39b/a}$ = 4.4 Hz, 1 H, 38-H), 5.29 (dq, $^3J_{34a,33}$ = 16.9 Hz, $^2J_{34a,34b} \approx {}^4J_{34a,32}$ = 1.6 Hz, 1 H, 34-H_a), 5.20 (dq, $^3J_{34b,33}$ = 10.5 Hz, $^2J_{34b,34a} \approx {}^4J_{34b,32}$ = 1.4 Hz, 1 H, 34-H_b), 5.15 (d, $^3J_{NHc,54}$ = 9.2 Hz, 1 H, NH_c), 5.01 (d, $^3J_{49,50}$ =11.0 Hz, 1 H, 49-H), 4.86 (m, 1 H, 20-H), 4.81 (d, $^3J_{15,16}$ = 9.7 Hz, 1 H, 15-H), 4.57−4.71 (m, 5 H, 10-H, 27-H, 32-H, 44-H), 4.54 (m, 1 H, 54-H), 4.41 (dd, $^3J_{4,5b}$ = 8.9 Hz, $^3J_{4,5a}$ = 3.0 Hz, 1 H, 4-H), 4.03 (m, 1 H, 36-H_a), 3.88−3.97 (m, 2 H, 25-H_a, 47-H_a), 3.67−3.77 (m, 3 H, 8-H_a, 36-H_b, 47-H_b), 3.64 (dd, $^2J_{25b,25a}$ = 10.3 Hz, $^3J_{25b,22}$ = 6.3 Hz, 1 H, 25-H_b), 3.30 (m, 1 H, 8-H_b), 3.04 (s, 3 H, 52-H), 3.03 (s, 3 H, 42-H), 2.95 (s, 3 H, 30-H), 2.73 (s, 3 H, 18-H), 2.59 (m, 1 H, 22-H), 2.48 (m, 1 H, 6-H), 2.08−2.34 (m, 5 H, 21-H_a, 28-H, 45-H_a, 46-H_a, 50-H), 2.02 (ddd, $^2J_{5a,5b}$ = 12.8 Hz, $^3J_{5a,6}$ = 6.4 Hz, $^3J_{5a,4}$ 3.1 Hz, 1 H, 5-H_a), 1.85−1.99 (m, 3 H, 39-H_a, 45-H_b, 46-H_b), 1.70−1.84 (m, 3 H, 5-H_b, 11-H_a, 21-H_b), 1.58−1.70 (m, 3 H, 12-H, 39-H_b, 55-H_a), 1.45−1.57 (m, 4 H, 11-H_b, 40-H, 55-H_a, 57-H), 1.42 (s, 18 H, 1-H, 61-H), 1.36 (m, 2 H, 23-H), 1.31 (d, $^3J_{17,16}$ = 6.1 Hz, 3 H, 17-H), 1.20 (m, 2 H, 56-H), 1.08 (d, $^3J_{7,6}$ = 6.7 Hz, 3 H, 7-H), 0.97 (m, 3 H, 29-H), 0.88−0.94 (m, 15 H, 13-H, 13-H', 24-H, 41-H, 58-H), 0.84−0.88 (m, 12 H, 29-H', 41-H', 51-H, 58-H'), 0.81 (d, $^3J_{51,50}$ = 6.7 Hz, 3 H, 51-H').

^{13}C-NMR (126 MHz, $CDCl_3$): δ = 173.9 (s, C-53), 173.4 (s, C-43), 173.4 (s, C-19), 171.5 (s, C-3), 171.3 (s, C-37), 170.8 (s, C-9), 170.1 (s, C-26), 169.7 (s, C-48), 168.8 (s, C-35), 167.4 (s, C-14), 157.2 (s, C-31), 155.8 (s, C-59), 133.1 (d, C-33), 117.3 (t, C-34), 81.1 (s, C-2), 79.8 (s, C-60), 67.9 (d, C-16), 66.4 (t, C-32), 63.6 (d, C-15), 61.2 (d, C-27), 59.9 (d, C-4), 59.5 (d, C-49), 56.7 (d, C-44), 55.2 (d, C-38), 54.9 (d, C-20), 53.5 (t, C-8), 52.9 (t, C-25), 50.9 (d, C-54), 49.4 (d, C-10), 48.0 (t, C-47), 41.3 (t, C-36), 40.2 (d, C-22), 40.1 (t, C-11), 36.7 (t, C-5), 35.6 (t, C-21; C-39), 34.5 (t, C-56), 32.8 (d, C-6), 31.2 (q, C-42), 30.7 (t, C-55), 30.4 (q, C-52), 29.6 (q, C-18), 29.5 (q, C-30), 28.9 (t, C-45), 28.4 (q, C-61), 28.11 (d, C-28), 28.06 (q, C-1), 28.0 (d, C-57), 27.7 (d, C-50), 26.3 (t, C-23), 25.8 (t, C-46), 25.2 (d, C-40), 24.7 (d, 12), 23.6 (q, C-13), 23.5 (q, C-41), 22.9 (q, C-58), 22.5 (q, C-58'), 21.5 (q, C-41'), 21.3 (q, C-13'), 19.2 (q, C-29), 19.0 (q, C-29'), 18.9 (q, C-51), 18.6 (q, C-51'), 18.5 (q, C-17), 17.5 (q, C-7), 12.5 (q, C-24).

HRMS (ESI): Calculated for $C_{70}H_{121}N_{10}O_{16}^+$ $[M+H]^+$: 1357.8957, found: 1357.8949.

Alloc-*N*-Me-L-Val-L-EtPro-*N*-Me-*cyclo*-*O*-(Gly-*N*-Me-D-Leu-L-Pro-*N*-Me-L-Val-L-HoLeu-L-MePro-L-Leu)-L-Thr [24]

459 mg (338 µmol, 1.0 eq.) of decapeptide **23** were dissolved with 3.6 mL of a 1:1 mixture of DCM/TFA. After 2 h, the solvent was removed in vacuo and the residue was co-evaporated three times with $CHCl_3$. Lastly, the obtained residue was lyophilized.

The obtained lyophilizate was reacted in seven batches, whereby a macrocyclization was exemplarily carried out as follows: Under an N_2-atmosphere, 89.4 µL (513 µmol, 11 eq., ρ = 0.742 g/mL) of DIPEA and 89.7 mg (233 µmol, 5.0 eq.) of FDPP were dissolved in 38 mL abs. DMF and heated in an oil bath to 70 °C. To this solution, a solution of 65.9 mg (46.6 µmol, 1.0 eq.) of the previously deprotected linear peptide in 10 mL abs. DMF was added dropwise over 20 min using a syringe pump. After stirring for another 20 min at 70 °C, the mixture was cooled to rt and concentrated in vacuo.

The batches were then combined and worked up. For this, the residue was taken up in EtOAc, washed with 1 M $HCl_{(aq.)}$, 1 M $LiCl_{(aq.)}$, sat. $NaHCO_{3(aq.)}$- and sat. $NaCl_{(aq.)}$ solution. The organic phase was dried over $MgSO_4$ and the solvent was removed in vacuo. After automated column chromatography (C_{18}-SiO_2, H_2O/MeCN 9:1 → 0:10) and lyophilization, 208 mg (176 µmol, 52%) of the cyclic peptide **24** was isolated as a colorless lyophilizate.

$[\alpha]_D^{20}$ = −66.4 (c = 0.5, $CHCl_3$)

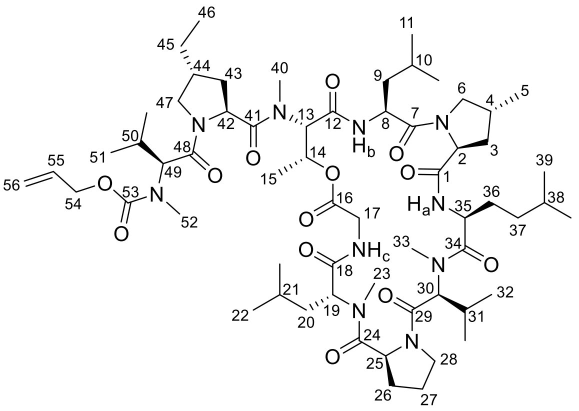

Main rotamer

^{1}H-NMR (500 MHz, $CDCl_3$): δ = 8.76 (m, 1 H, NH_c), 7.90 (d, $^3J_{NHa,35}$ = 9.5 Hz, 1 H, NH_a), 6.47 (d, $^3J_{NHb,8}$ = 7.8 Hz, 1 H, NH_b), 5.92 (m, 2 H, 14-H, 55-H), 5.59 (d, $^3J_{13,14}$ = 1 H, 13-H), 5.30 (m, 1 H, 56-H_a), 5.21 (m, 1 H, 56-H_b), 5.16 (dd, $^3J_{19,20a/b}$ = 8.6 Hz, $^3J_{19,20b/a}$ = 5.9 Hz, 1 H, 19-H), 4.95 (m, 1 H, 42), 4.83 (m, 1 H, 35-H), 4.77–4.81 (m, 2 H, 25-H, 30-H), 4.56–4.63 (m, 4 H, 2-H, 49-H, 54-H), 4.37 (m, 1 H, 8-H), 4.24 (dd, $^2J_{17a,17b}$ = 17.1 Hz, $^3J_{17a,NHc}$ = 6.6 Hz, 1 H, 17-H_a), 4.02 (dd, $^2J_{17b,17a}$ = 17.1 Hz, $^3J_{17b,NHc}$ = 3.9 Hz, 1 H, 17-H_b), 3.94 (m, 1 H, 47-H_a), 3.69 (ddd, $^2J_{28a,28b}$ = 12.1 Hz, $^3J_{28a,27a/b}$ = 8.3 Hz, $^3J_{28a,27b/a}$ = 3.7 Hz, 1 H, 28-H_a), 3.59 (dt, $^2J_{28b,28a}$ = 12.0 Hz, $^3J_{28b,27a}$ ≈ $^3J_{28b,27b}$ = 8.3 Hz, 1 H, 28-H_b), 3.50 (t, $^2J_{6a,6b}$ ≈ $^3J_{6a,4}$ = 8.5 Hz, 1 H, 6-H_a), 3.37 (dd, $^2J_{47b,47a}$ = 10.2 Hz, $^3J_{47b,44}$ = 8.1 Hz, 1 H, 47-H_b), 3.29 (s, 3 H, 33-H), 3.12 (s, 3 H, 40-H), 3.09 (m, 1 H, 6-H_b), 2.92 (s, 3 H, 52-H), 2.85 (s, 3 H, 23-H), 2.55 (m, 1 H, 3-H_a), 2.39–2.52 (m, 2 H, 4-H, 44-H), 2.20–2.36 (m, 3 H, 26-H_a, 31-H, 50-H), 2.10 (m, 1 H, 43-H_a), 1.97 (m, 1 H, 26-H_b), 1.97 (m, 1 H, 27-H_a), 1.68–1.85 (m, 5 H, 10-H, 20-H_a, 27-H, 36-H_a, 43-H_b), 1.52 (m, 2 H, 9-H_a, 38-H), 1.34 (d,

$^3J_{15,14}$ = 6.3 Hz, 3 H, 15-H), 1.30–1.47 (m, 6 H, 3-H$_b$, 20-H$_b$, 21-H, 36-H$_b$, 45-H), 1.18–1.29 (m, 2 H, 9-H$_b$, 37-H), 1.08 (d, $^3J_{5,4}$ = 6.4 Hz, 3 H, 5-H), 1.04 (m, 1 H, 37-H), 0.95 (d, $^3J_{11b,10}$ = 6.6 Hz, 3 H, 11-H'), 0.84–0.93 (m, 27 H, 11-H, 22-H, 22-H', 32-H', 39-H, 39-H', 46-H, 51-H, 51-H'), 0.75 (d, $^3J_{32,31}$ = 6.7 Hz, 3 H, 32-H).

^{13}C-NMR (126 MHz, $CDCl_3$): δ = 174.6 (s, C-34), 173.7 (s, C-7), 173.6 (s, C-41), 171.6 (s, C-18), 170.7 (s, C-16), 170.6 (s, C-1), 170.0 (s, C-24), 169.4 (s, C-48), 169.2 (s, C-12), 168.9 (s, C-29), 156.2 (s, C-53), 133.1 (d, C-55), 118.5 (t, C-56), 69.3 (d, C-14), 66.3 (t, C-54), 61.5 (d, C-49), 60.4 (d, C-2), 60.0 (d, C-13), 58.8 (d, C-25), 57.7 (d, C-30), 56.3 (d, C-42), 55.1 (d, C-19), 53.5 (t, C-6), 52.8 (t, C-47), 51.1 (d, C-8), 48.7 (d, C-35), 47.0 (t, C-28), 43.0 (t, C-17), 40.3 (t, C-9), 39.5 (d, C-44), 37.0 (t, C-20), 34.7 (t, C-37), 34.6 (t, C-43), 34.3 (q, C-40), 33.7 (d, C-4), 33.2 (t, C-3), 31.6 (t, C-26), 31.0 (q, C-33), 30.9 (t, C-36), 30.6 (q, C-23), 29.5 (q, C-52), 28.4 (d, C-31), 28.1 (d, C-38), 27.7 (d, C-50), 26.3 (t, C-45), 25.5 (d, C-10), 24.7 (d, C-21), 23.6 (q, C-11'), 23.4 (q, C-11), 22.9 (q, C-22), 22.5 (q, C-39, C-39'), 22.0 (t, C-27), 21.0 (q, C-22'), 19.1 (q, C-51), 18.8 (q, C-32), 18.6 (q, C-32'), 18.5 (q, C-51'), 17.9 (q, C-15), 16.9 (q, C-5), 12.5 (q, C-46).

Minor rotamer (selected signals):

^{1}H-NMR (500 MHz, $CDCl_3$): δ = 7.87 (d, $^3J_{NHa,35}$ = 9.5 Hz, 1 H, NH$_a$), 6.52 (d, $^3J_{NHb,8}$ = 7.8 Hz,1 H, NH$_b$), 5.57 (d, $^3J_{13,14}$ = 3.2 Hz, 1 H, 13-H), 4.44 (d, $^3J_{49,50}$ = 10.8 Hz, 1 H, 49-H), 2.87 (s, 3 H, 52-H).

^{13}C-NMR (126 MHz, $CDCl_3$): δ = 170.8 (s, C-16), 168.5 (s, C-48), 157.0 (s, C-53), 132.8 (d, C-55), 117.3 (t, C-56), 69.2 (d, C-14), 66.6 (d, C-54), 62.2 (d, C-49), 60.1 (d, C-13), 56.6 (d, C-42), 52.6 (t, C-47), 29.7 (d, C-52), 27.5 (d, C-50), 26.5 (t, C-45), 12.6 (q, C-46).

HRMS (ESI): Calculated for $C_{61}H_{102}N_{10}NaO_{13}^+$ [M+Na]$^+$: 1205.7520, found: 1205.7508.

Mycoplanecin A

Under an N_2-atmosphere 208 mg (176 µmol, 1.0 eq.) of the Alloc-protected amine **24** and 134 mg (860 µmol, 4.9 eq.) DMBA were dissolved in 2.3 mL anhydrous DCM. Afterwards, 10.9 mg (9.41 µmol, 5 mol%) $Pd(PPh_3)_4$ was added, and the resulting orange solution was stirred for 2.5 h. Next, the mixture was diluted with EtOAc, washed three times with sat. $NaHCO_3$ solution, dried over $MgSO_4$ and the solvent was evaporated in vacuo.

Preparation of the acid chloride solution: 112 mg (1.09 mmol, 6.2 eq.) of 2-oxobutyric acid and 94.0 µL (1.07 mmol, 6.1 eq., ρ = 1.450 g/mL) of oxalyl chloride were dissolved in 1.0 mL anhydrous DCM under an N_2-atmosphere and treated with a few drops of anhydrous DMF, resulting in a vigorous gas evolution. After 2.5 h, the acid chloride solution was added slowly dropwise to a solution of the previously obtained free amine and 306 µL (1.76 mmol, 10 eq., ρ = 0.742 g/mL) DIPEA in 1.0 mL anhydrous at 0 °C, where a white smoke formed during addition.

After 19 h, the mixture was diluted with EtOAc, washed with 1 M $HCl_{(aq.)}$, H_2O, sat. $NaHCO_{3(aq.)}$ and sat. $NaCl_{(aq.)}$ solution and dried over $MgSO_4$. The solvent was evaporated in vacuo and the residue was purified by automated column chromatography (C_{18}-SiO_2,

H_2O/MeCN 9:1 ⟶ 0:10), followed by preparative HPLC purification (H_2O/MeCN 3:7 ⟶ 0:10). After lyophilization, 145 mg (123 µmol, 70%) of Mycoplanecin A (**M45**) was isolated as a colorless lyophilizate.

$[\alpha]_D^{20}$ = −57.5 (c = 0.4, $CHCl_3$); Lit.: $[\alpha]_D^{25}$ = −66 (c = 0.4, $CHCl_3$)[16]

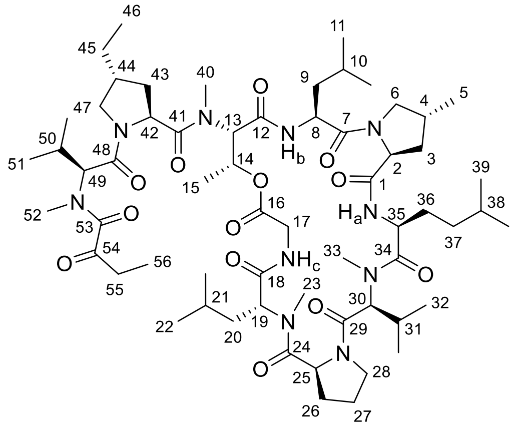

¹H-NMR (500 MHz, $CDCl_3$): δ = 8.79 (dd, $^3J_{NHc,17b}$ = 6.5 Hz, $^3J_{NHc,17b}$ = 3.9 Hz, 1 H, NH_c). 7.90 (d, $^3J_{NHa,35}$ = 9.3 Hz, 1 H, NH_a), 6.47 (d, $^3J_{NHb,8}$ =7.8 Hz, 1 H, NH_b), 5.92 (qd, $^3J_{14,15}$ =9.5 Hz, $^3J_{14,13}$ 3.2 Hz, 1 H, 14-H), 5.59 (d, $^3J_{13,14}$ = 3.2 Hz, 1 H, 13-H), 5.17 (dd, $^3J_{19,20a/b}$ = 8.7 Hz, $^3J_{19,20b/a}$ = 5.9 Hz, 1 H, 19-H), 4.92–4.97 (m, 2 H, 42-H, 49-H), 4.84 (m, 1 H, 35-H), 4.77–4.83 (m, 2 H, 25-H, 30-H), 4.59 (d, $^3J_{2,3}$ = 8.1, 1 H, 2-H), 4.38 (m, 1 H, 8-H), 4.26 (dd, $^2J_{17a,17b}$ = 17.0 Hz, $^3J_{17a,NHc}$ = 6.5 Hz, 1 H, 17-H_a), 4.02 (dd, $^2J_{17b,17a}$ = 17.1 Hz, $^3J_{17b,NHc}$ = 3.9 Hz, 1 H, 17-H_b), 3.97 (dd, $^2J_{47a,47b}$ = 10.1 Hz, $^3J_{47a,44}$ = 7.6 Hz, 1 H, 47-H_a), 3.70 (ddd, $^2J_{28a,28b}$ = 12.1 Hz, $^3J_{28a,27a/b}$ = 8.4 Hz, $^3J_{28a,27b/a}$ = 3.5 Hz, 1 H, 28-H_a), 3.60 (dt, $^2J_{28b,28a}$ = 12.0 Hz, $^3J_{28b,27}$ =7.9 Hz, 1 H, 28-H_b), 3.51 (m, 1 H, 6-H_a), 3.35 (dd, $^2J_{47b,47a}$ = 10.2 Hz, $^3J_{47b,44}$ = 8.2 Hz, 1 H, 47-H_b), 3.30 (s, 3 H, 33-H), 3.13 (s, 3 H, 40-H), 3.10 (m, 1 H, 6-H_b), 2.97 (s, 3 H, 52-H), 2.86 (s, 3 H, 23-H), 2.77 (m, 2 H, 55-H), 2.56 (dd, $^2J_{3a,3b}$ = 12.3 Hz, $^3J_{3a,4}$ = 6.1 Hz, 1 H, 3-H_a), 2.42–2.52 (m, 2 H, 4-H, 44-H), 2.21–2.37 (m, 3 H, 26-H_b, 31-H, 50-H), 2.11 (ddd, $^2J_{43a,43b}$ = 12.6 Hz, $^3J_{43a,44}$ = 6.6 Hz, $^3J_{43a,42}$ = 2.6 Hz, 1 H, 43-H_b), 1.98 (m, 1 H, 26-H_b), 1.89 (m, 1 H, 27-H_a), 1.70–1.86 (m, 5 H, 10-H, 20-H_a, 27-H_b, 36-H_a, 43-H_b), 1.52 (m, 2 H, 9-H_a, 38-H), 1.34–1.46 (m, 6 H, 3-H_a, 20-H_b, 21-H, 36-H_b, 45-H), 1.35 (d, $^3J_{15,14}$ = 6.2 Hz, 3 H, 15-H), 1.31 (m, 1 H, 9-H_b), 1.21 (m, 1 H, 37-H_a), 1.15 (t, $^3J_{56,55}$ = 7.3 Hz, 3 H, 56-H), 1.09 (d, $^3J_{5,4}$= 6.5 Hz, 3 H, 5-H), 1.04 (m, 1 H, 37-H_b), 0.99 (d, $^3J_{51,50}$ = 6.5 Hz, 3 H, 51-H), 0.96 (d, $^3J_{11,10}$ = 6.7 Hz, 3 H, 11-H), 0.93 (d, $^3J_{11',10}$ = 6.5 Hz, 3 H, 11-H'), 0.84–0.94 (m, 21 H, 22-H, 22-H', 32-H', 39-H, 39-H', 46-H, 51-H'), 0.76 (d, $^3J_{32,31}$ =6.7 Hz, 3 H, 32-H).

¹³C-NMR (126 MHz, $CDCl_3$): δ = 201.9 (s, C-54), 174.7 (s, C-34), 173.7 (s, C-7), 173.5 (s, C-41), 171.7 (s, C-18), 170.8 (s, C-16), 170.6 (s, C-1), 170.0 (s, C-24), 169.1 (s, C-12), 168.9 (s, C-29), 168.2 (s, C-53), 168.0 (s, C-48), 69.2 (d, C-14), 60.4 (d, C-2), 60.1 (d, C-13), 59.0 (d, C-49), 58.8 (d, C-25), 57.7 (d, C-30), 56.6 (d, C-42), 55.1 (d, C-19), 53.5 (t, C-6), 52.9 (t, C-47), 51.2 (d, C-8), 48.7 (d, C-35), 47.0 (t, C-28), 43.1 (t, C-17), 40.3 (t, C-9), 39.5 (d, C-44), 37.0 (t, C-20), 34.7 (t, C-37), 34.5 (t, C-43), 34.3 (q, C-40), 33.8 (d, C-4), 33.7 (t, C-55), 33.3 (t, C-3), 31.6 (t, C-26), 31.0 (q, C-33), 30.9 (q, C-52), 30.9 (t, C-36), 30.6 (q, C-23), 28.4 (d, C-31), 28.2 (d, C-38), 27.3 (d, C-50), 26.3 (t, C-45), 25.5 (d, C-10), 24.7 (d, C-21), 23.6 (q, C-11'), 23.4 (q, C-11), 23.0 (q, C-22'), 22.5 (q, C-39, C-39'), 22.0 (t, C-27), 21.1 (q, C-22), 19.1 (q, C-51'), 19.0 (q, C-32'), 18.6 (q, C-32), 18.5 (q, C-51), 17.9 (q, C-15), 16.9 (q, C-5), 12.6 (q, C-46), 7.0 (q, C-56).

HRMS (ESI): Calculated for $C_{61}H_{102}N_{10}NaO_{13}^{+}$ [M+Na]$^{+}$: 1205.7520, found: 1205.7511.

NMR Spectra

(4*R*,5*R*)-4,5-Dicyclohexyl-2-[(trityloxy)methyl]-1,3,2-dioxaborolane [1]

^{1}H-NMR (500 MHz, $CDCl_3$):

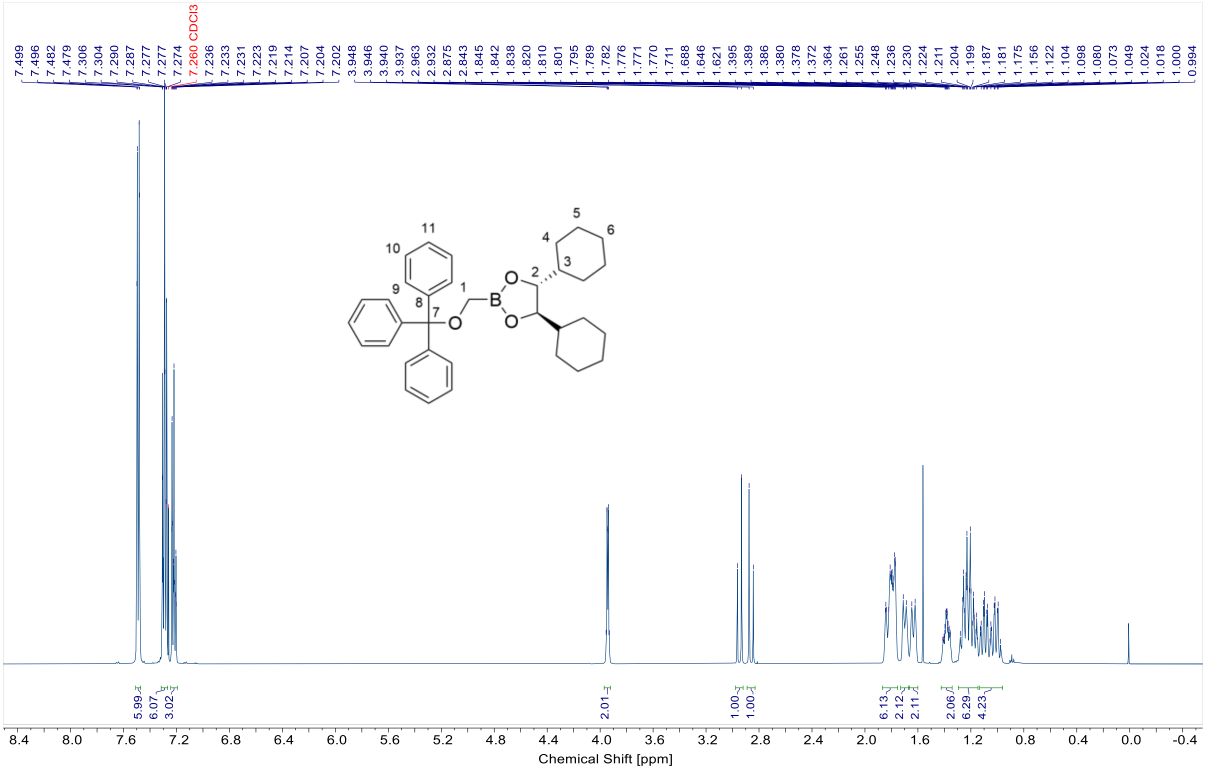

^{13}C-NMR (126 MHz, $CDCl_3$):

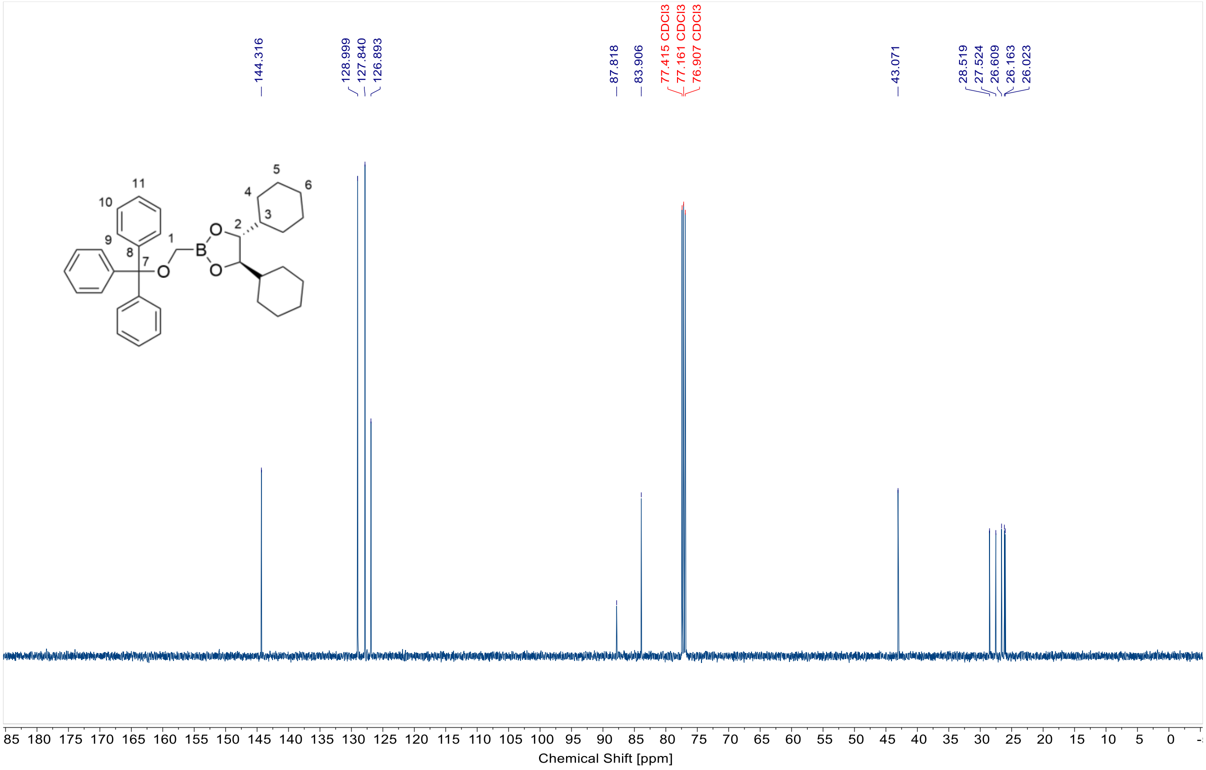

(^{1}H,^{1}H)-COSY ($CDCl_3$):

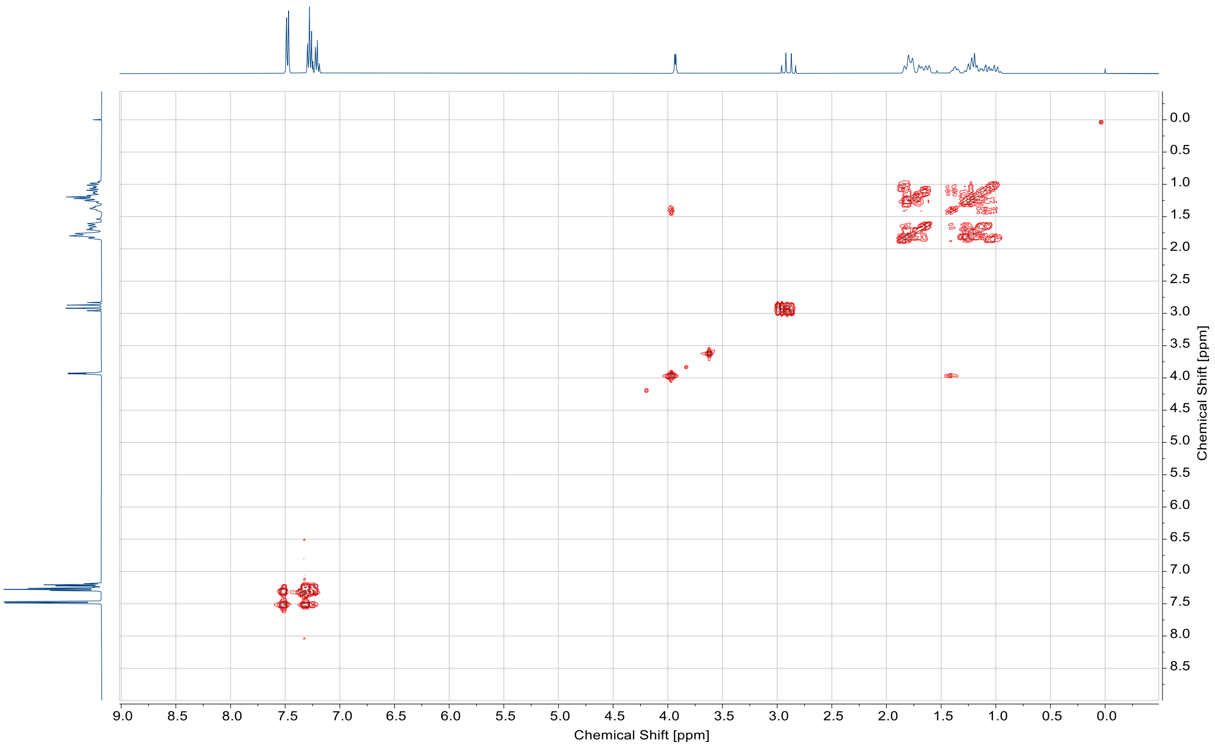

(^{1}H,^{13}C)-HSQC ($CDCl_3$):

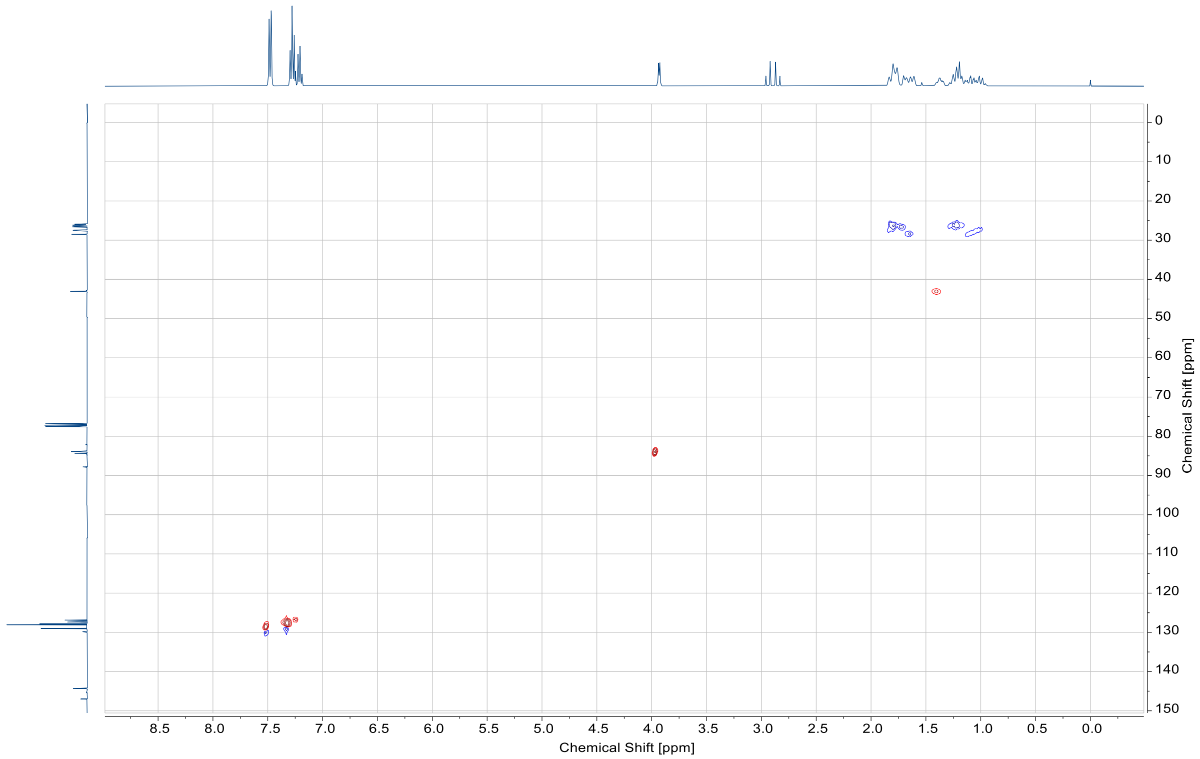

(^{1}H,^{13}C)-HMBC ($CDCl_3$):

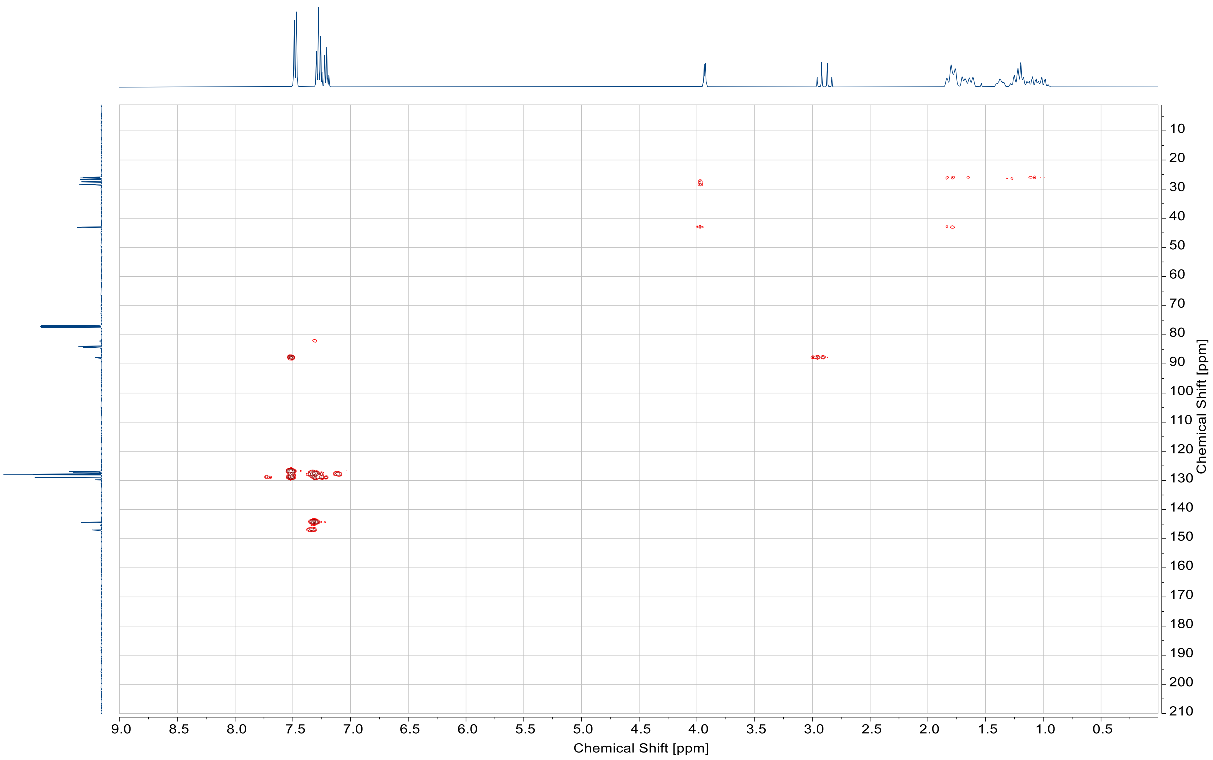

(4*R*,5*R*)-4,5-Dicyclohexyl-2-[(*S*)-1-(trityloxy)propan-2-yl]-1,3,2-dioxaborolane [2]

^{1}H-NMR (400 MHz, $CDCl_3$):

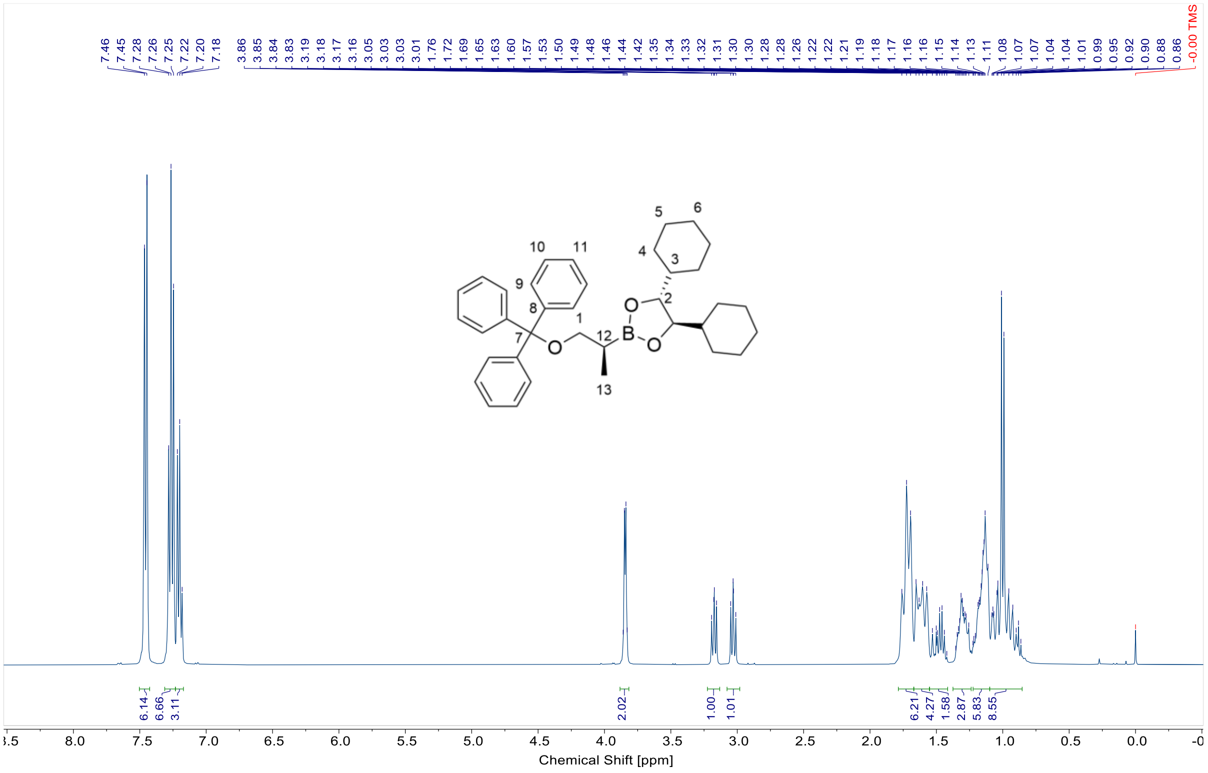

^{13}C-NMR (101 MHz, $CDCl_3$):

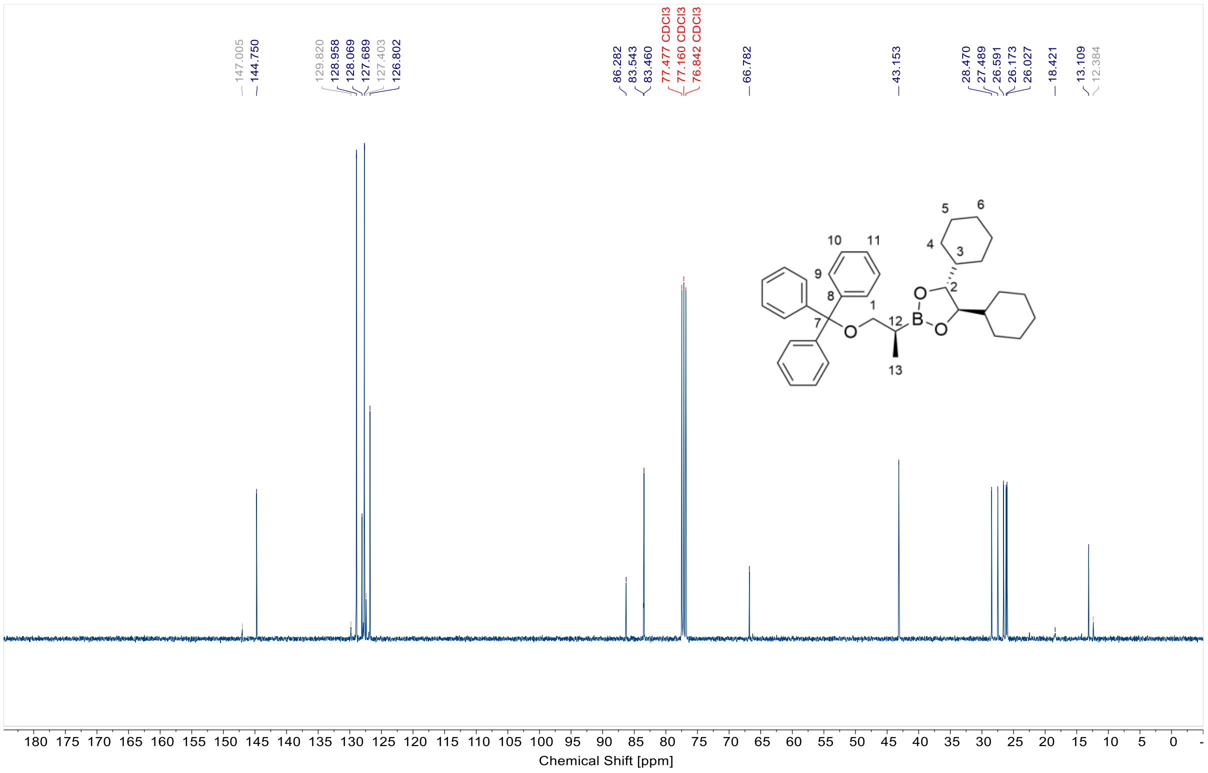

(^{1}H,^{1}H)-COSY ($CDCl_3$):

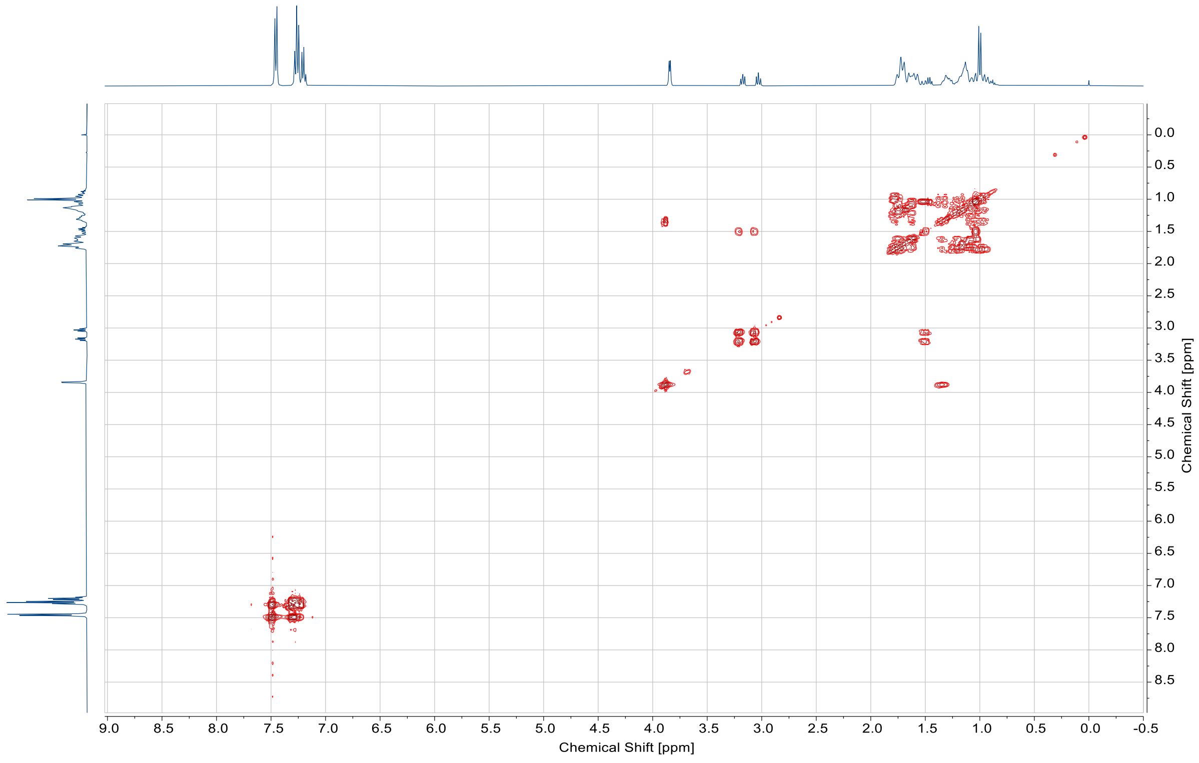

(^{1}H,^{13}C)-HSQC ($CDCl_3$):

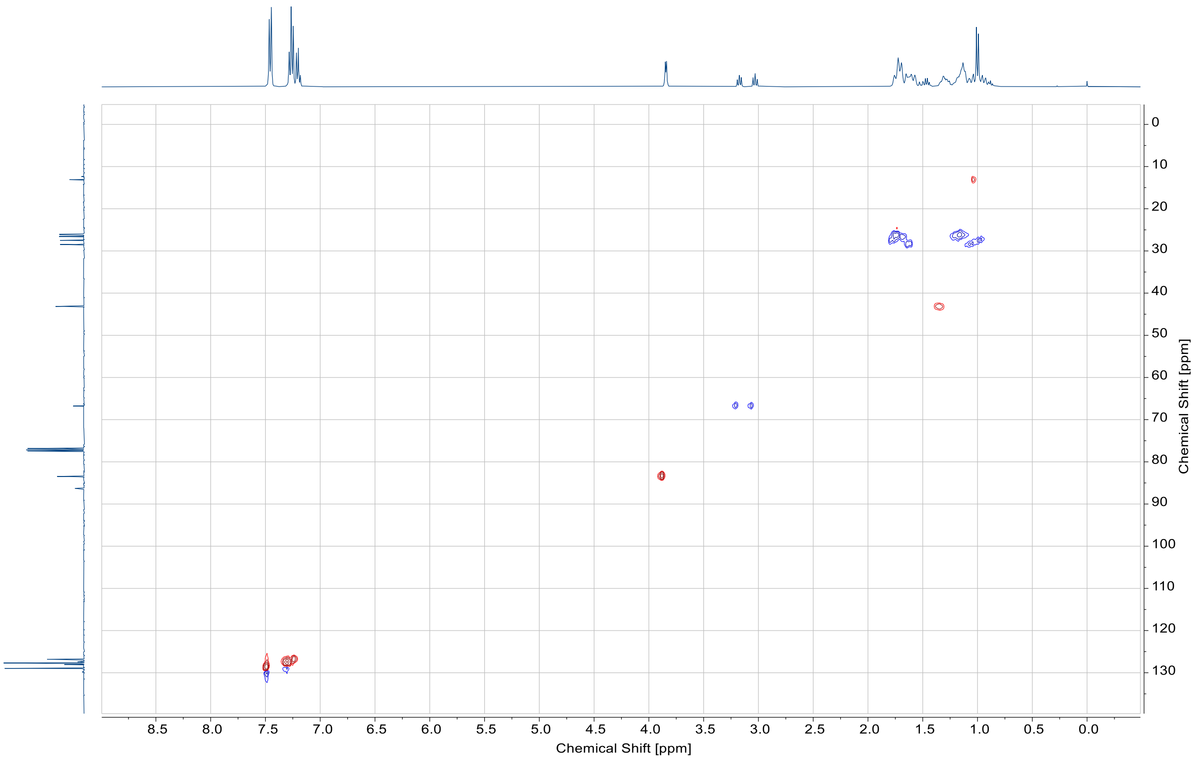

(^{1}H,^{13}C)-HMBC ($CDCl_3$):

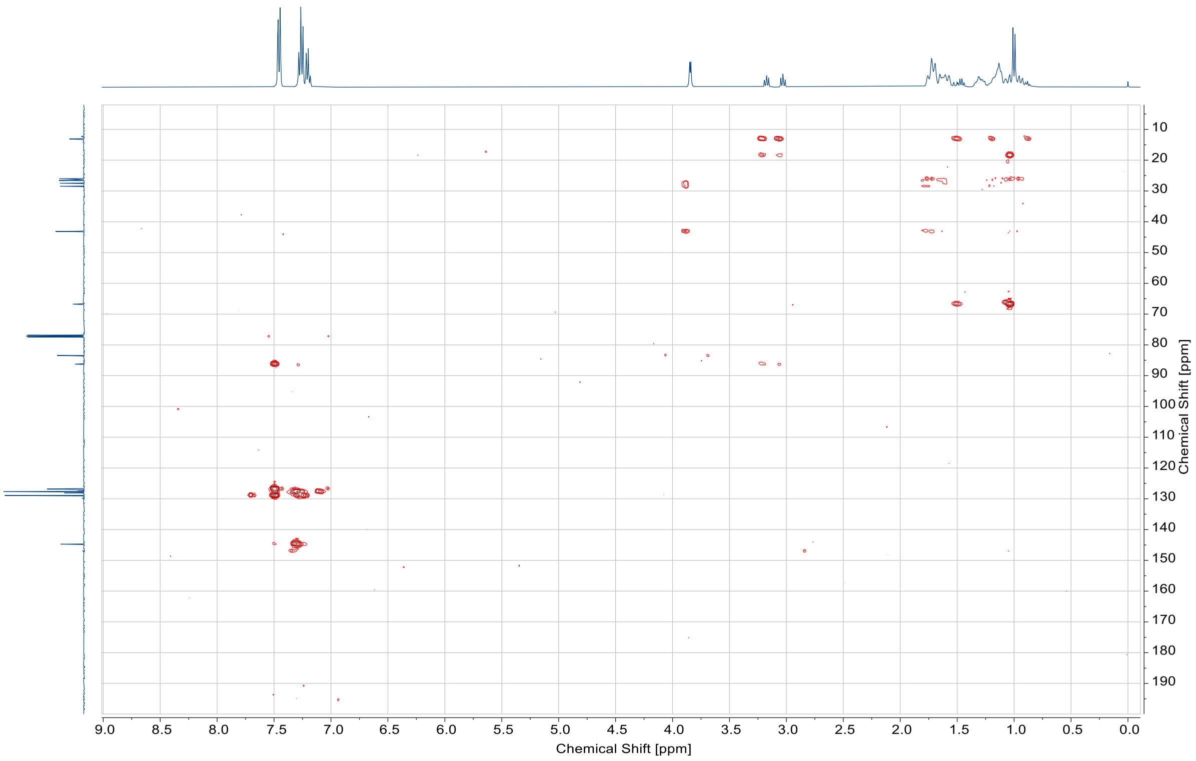

(4*R*,5*R*)-4,5-Dicyclohexyl-2-[(*S*)-1-(trityloxy)butan-2-yl]-1,3,2-dioxaborolane [3]

^{1}H-NMR (400 MHz, $CDCl_3$):

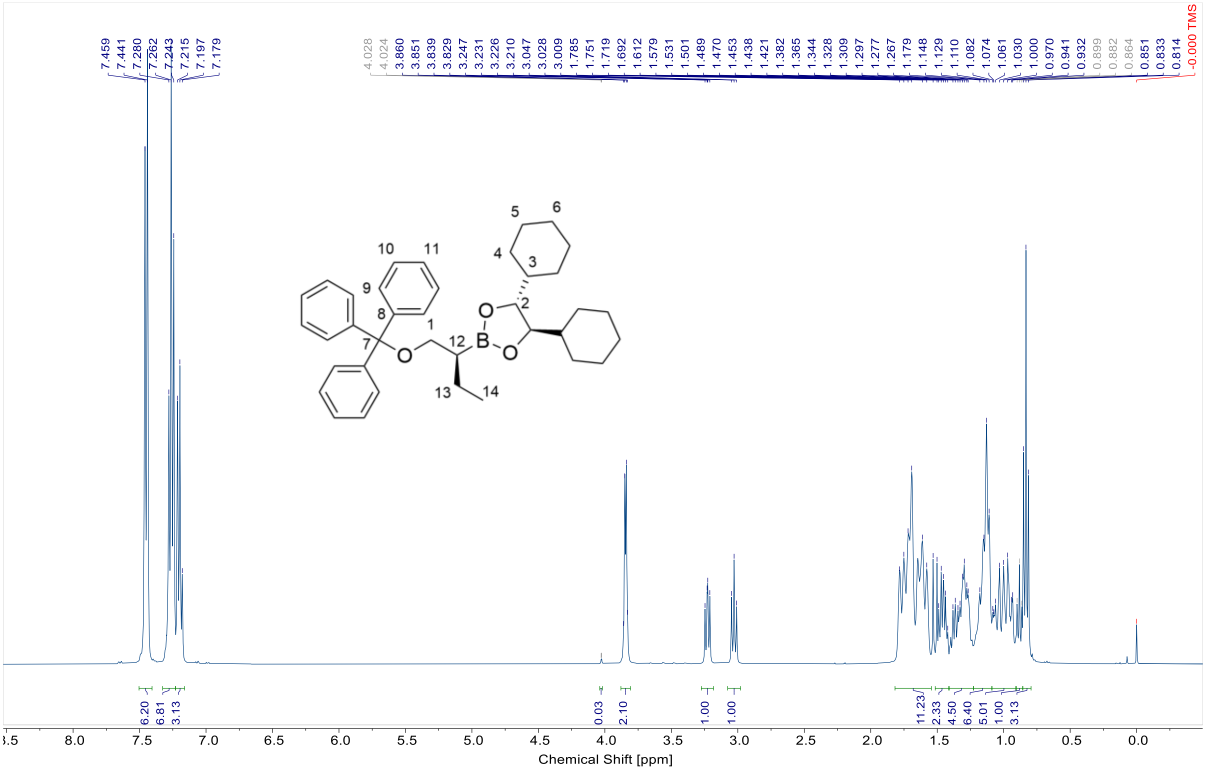

^{13}C-NMR (101 MHz, $CDCl_3$):

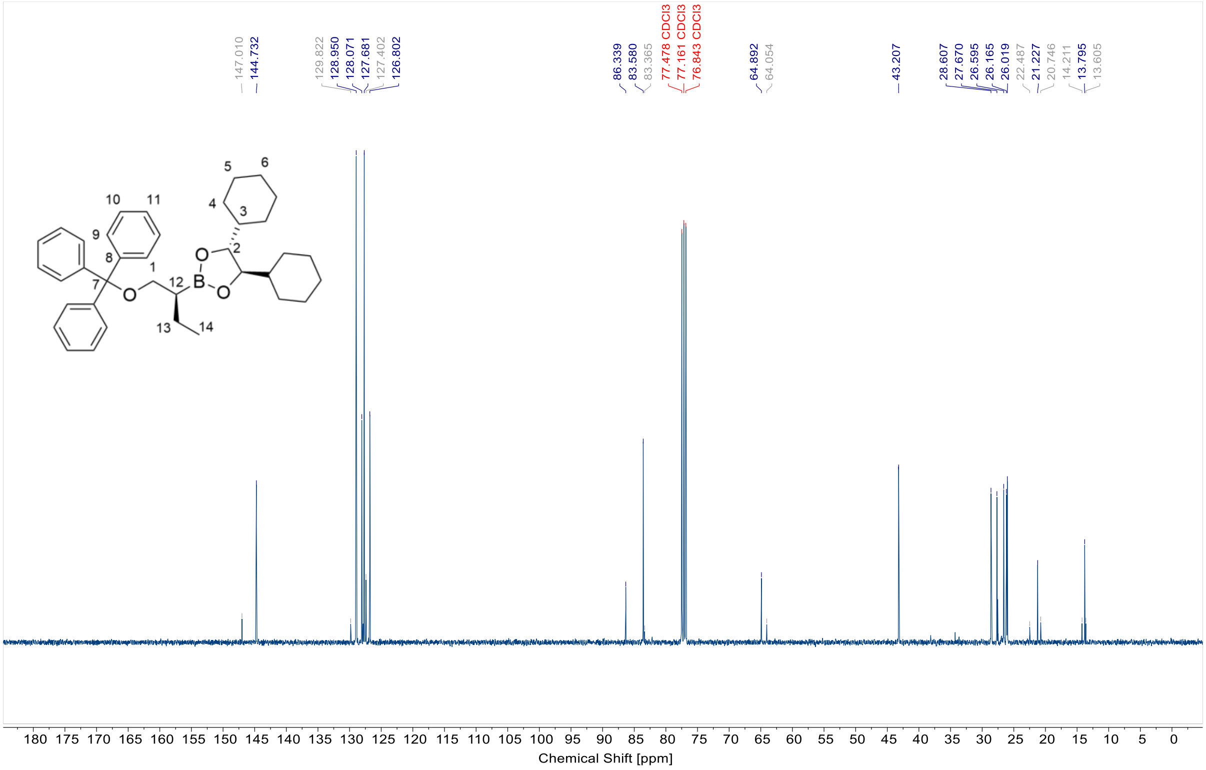

(^{1}H,^{1}H)-COSY ($CDCl_3$):

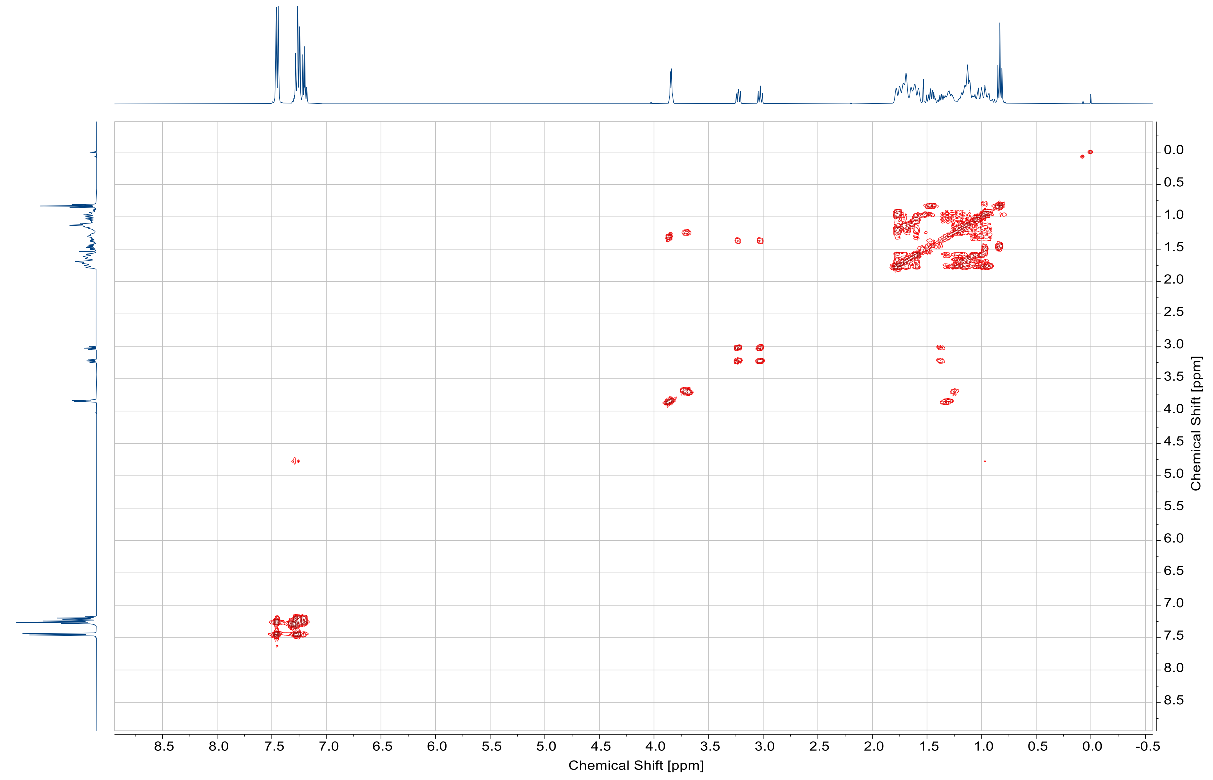

(^{1}H,^{13}C)-HSQC ($CDCl_3$):

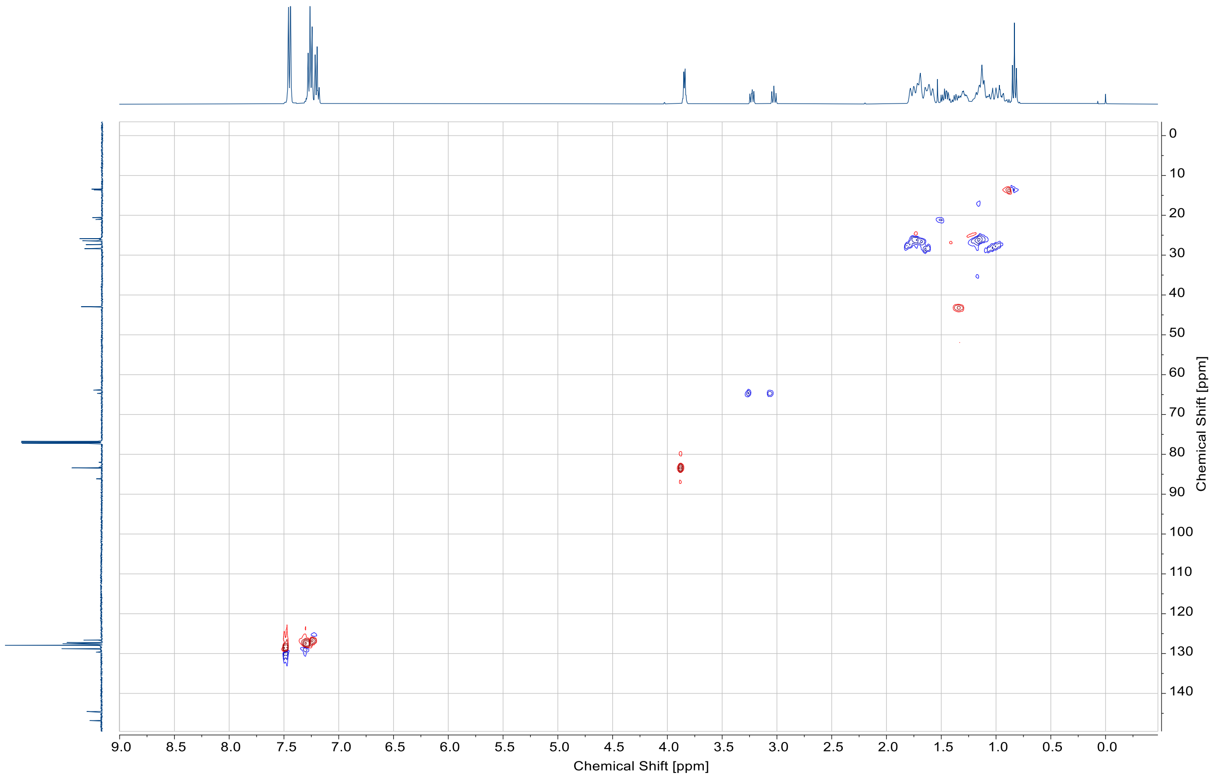

(^{1}H,^{13}C)-HMBC ($CDCl_3$):

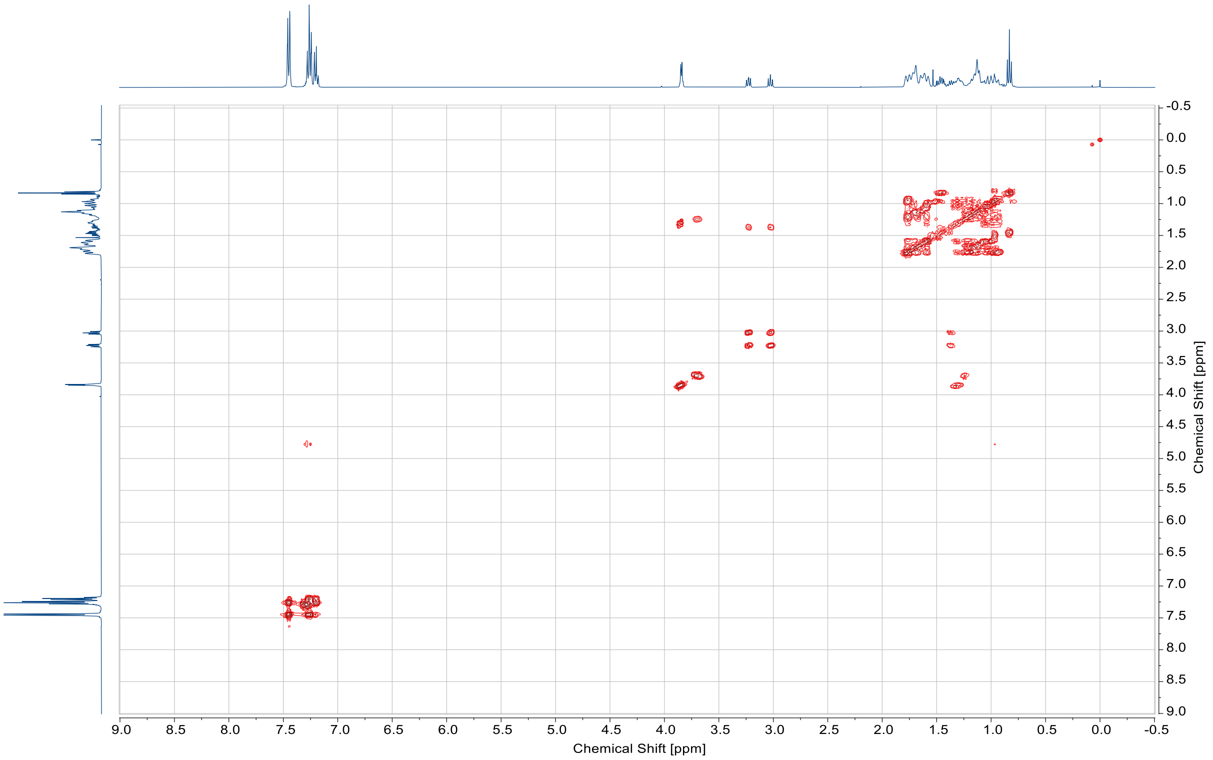

(4*R*,5*R*)-4,5-Dicyclohexyl-2-[(*R*)-2-methyl-3-(trityloxy)propyl]-1,3,2-dioxaborolane [4]

^{1}H-NMR (500 MHz, $CDCl_3$):

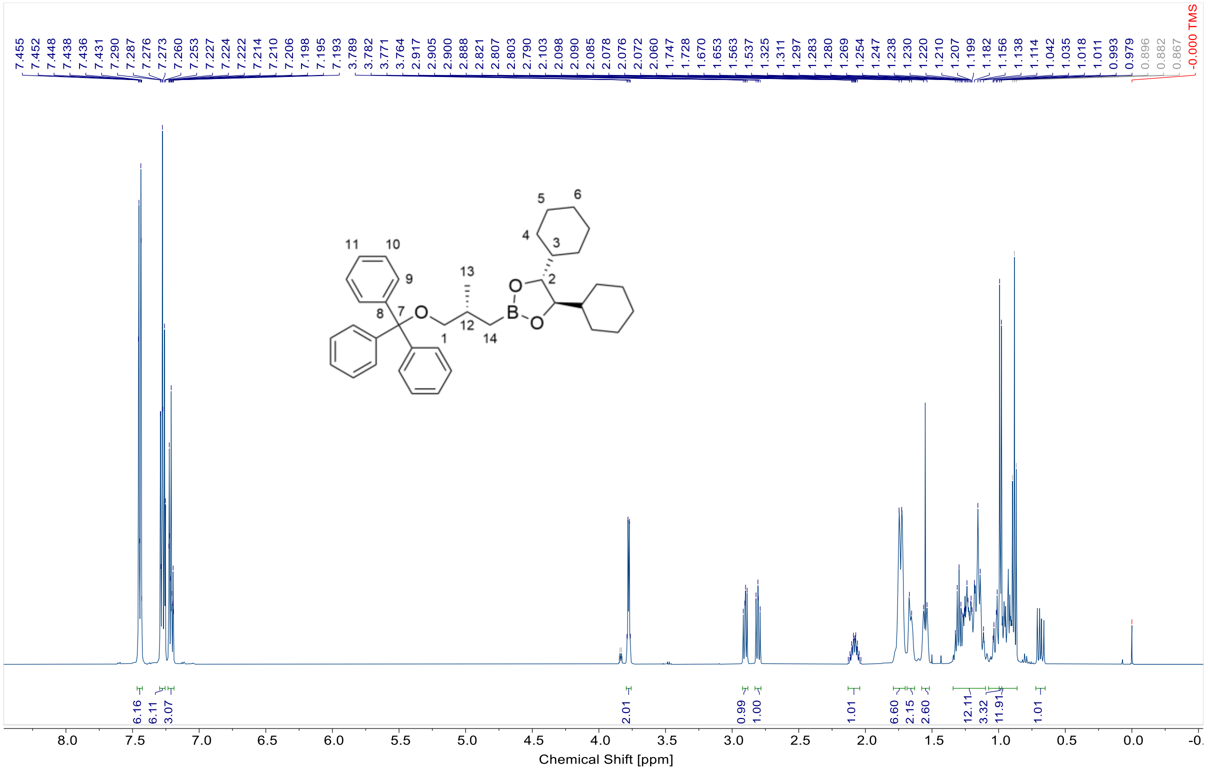

^{13}C-NMR (101 MHz, $CDCl_3$):

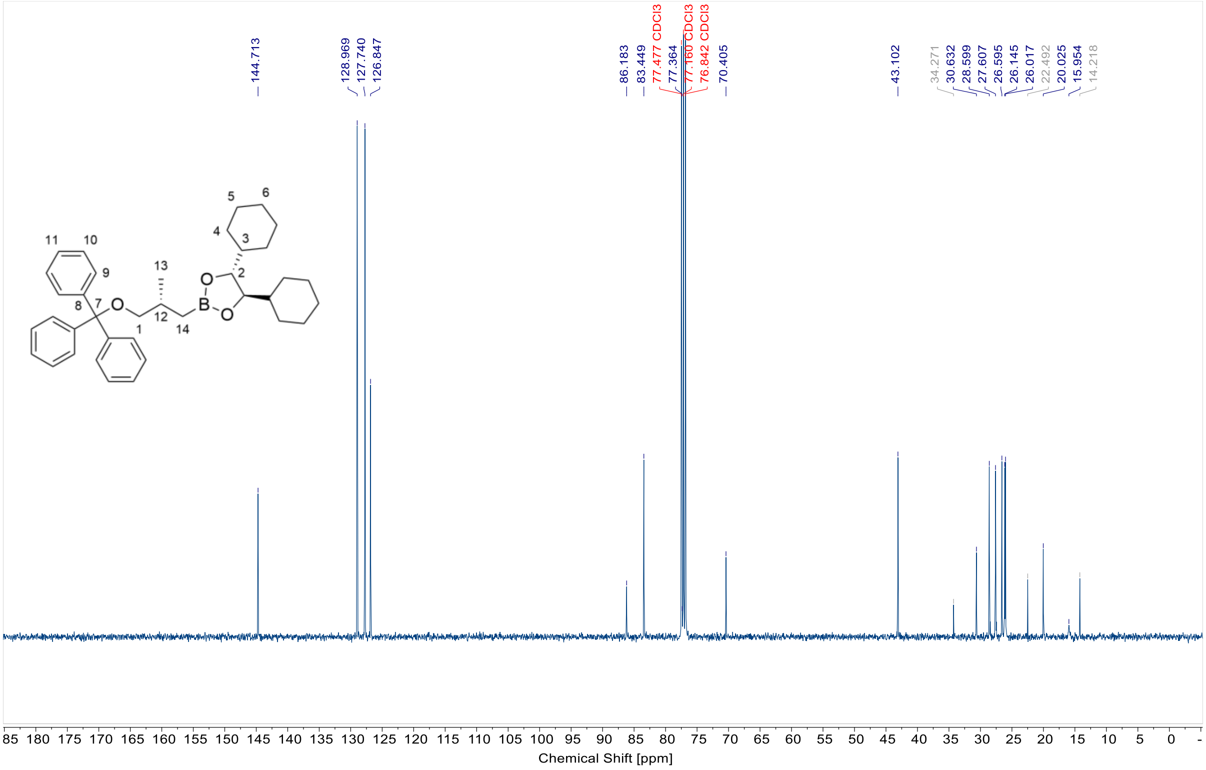

(^{1}H,^{1}H)-COSY ($CDCl_3$):

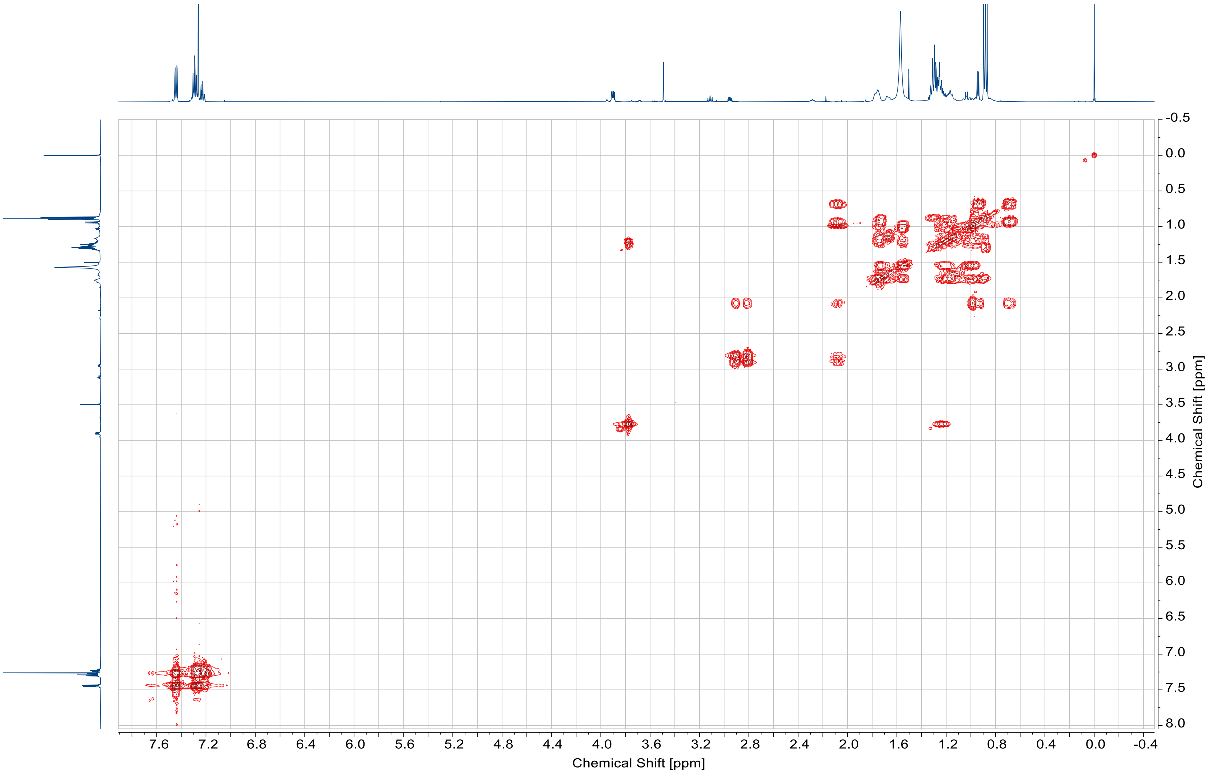

(^{1}H,^{13}C)-HSQC (CDCl$_3$):

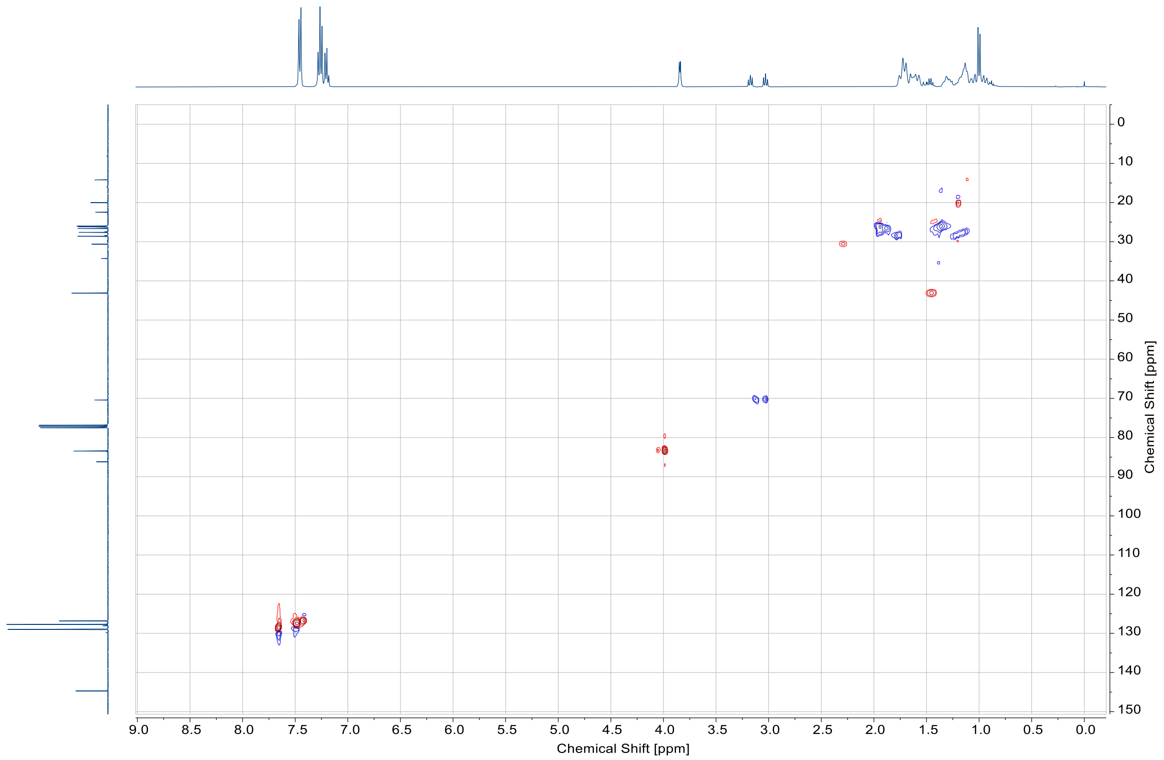

(^{1}H,^{13}C)-HMBC (CDCl$_3$):

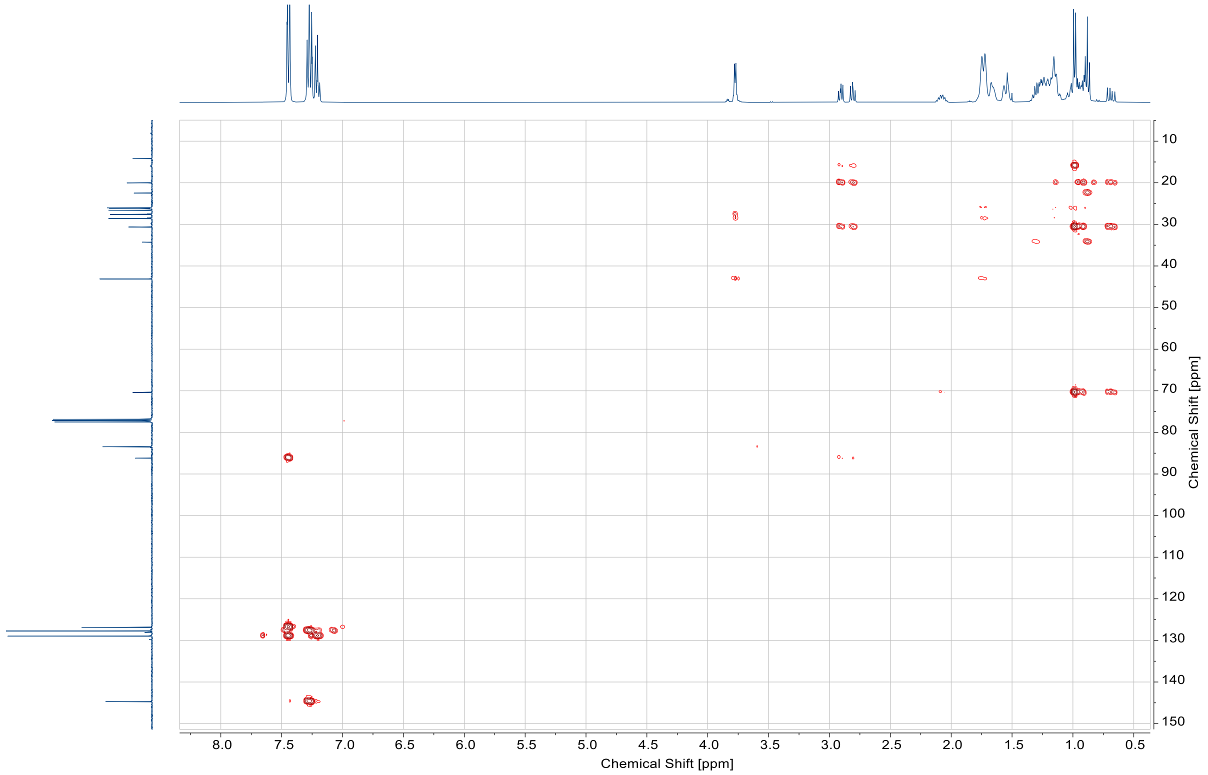

(4*R*,5*R*)-4,5-Dicyclohexyl-2-[(*R*)-2-methyl-3-(trityloxy)propyl]-1,3,2-dioxaborolane [5]

^{1}H-NMR (500 MHz, $CDCl_3$):

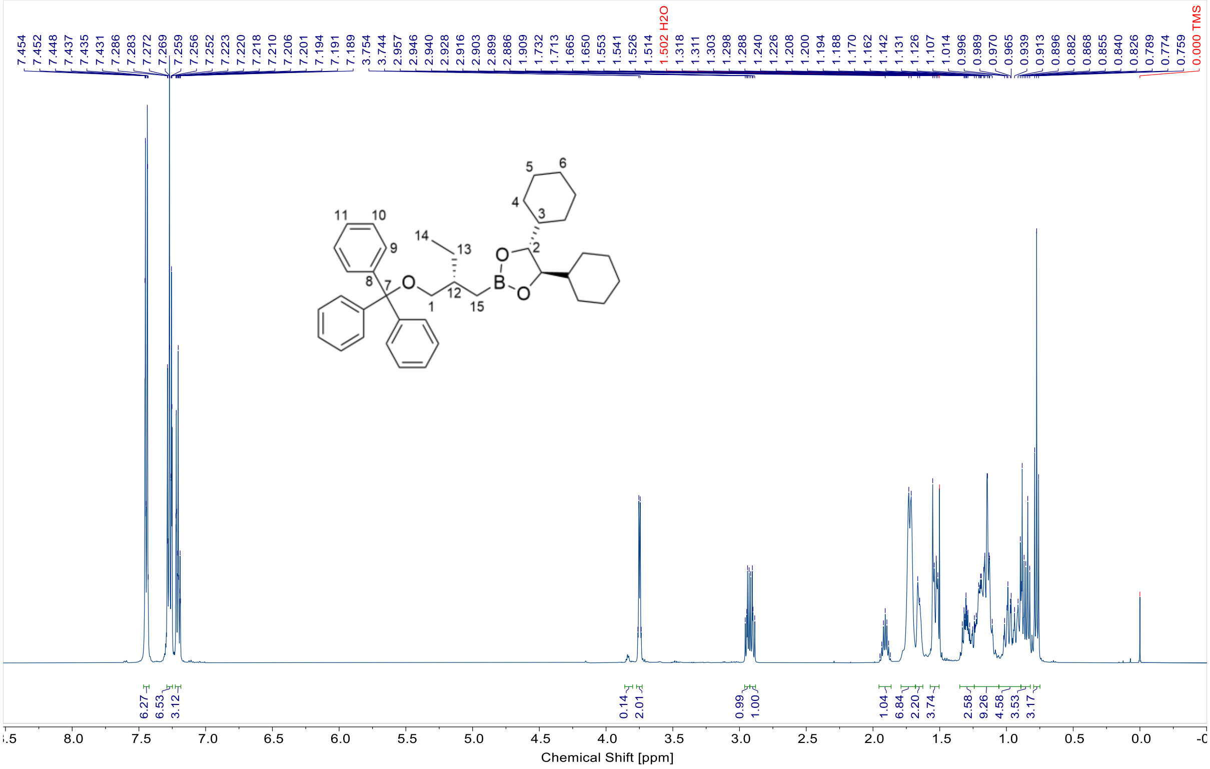

^{13}C-NMR (126 MHz, $CDCl_3$):

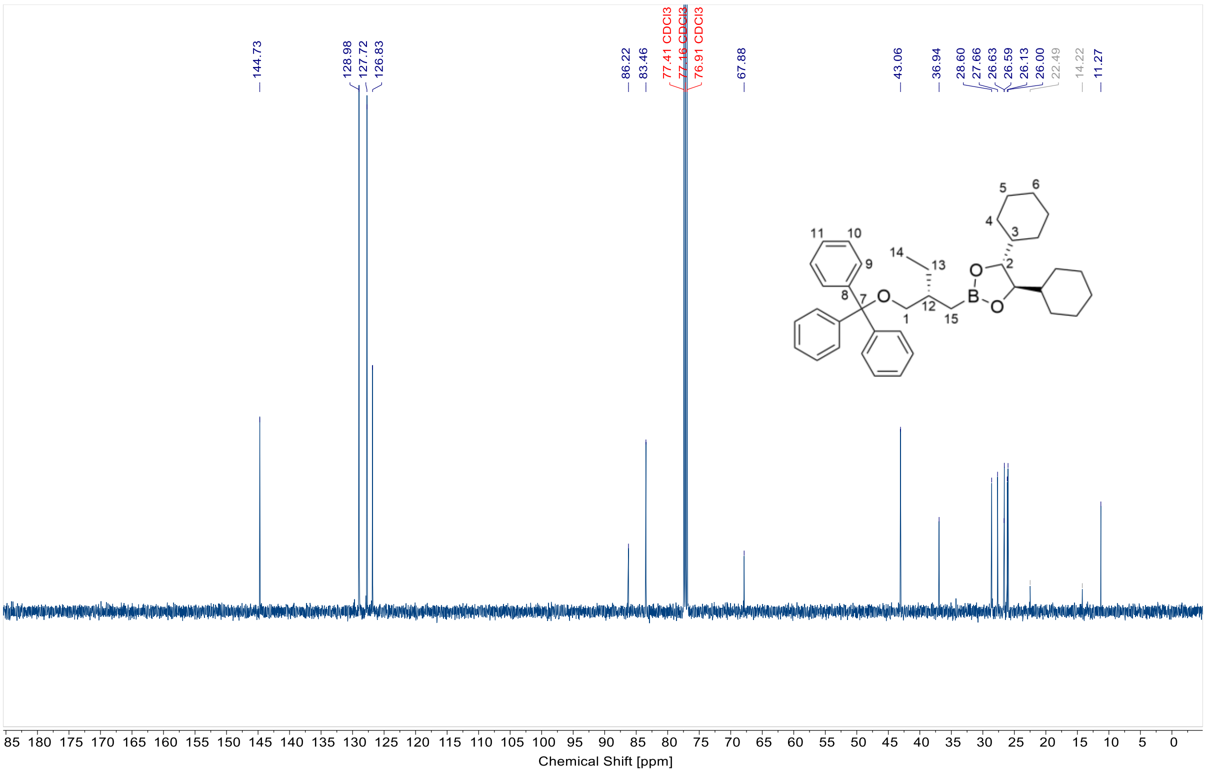

(^{1}H,^{1}H)-COSY (CDCl$_3$):

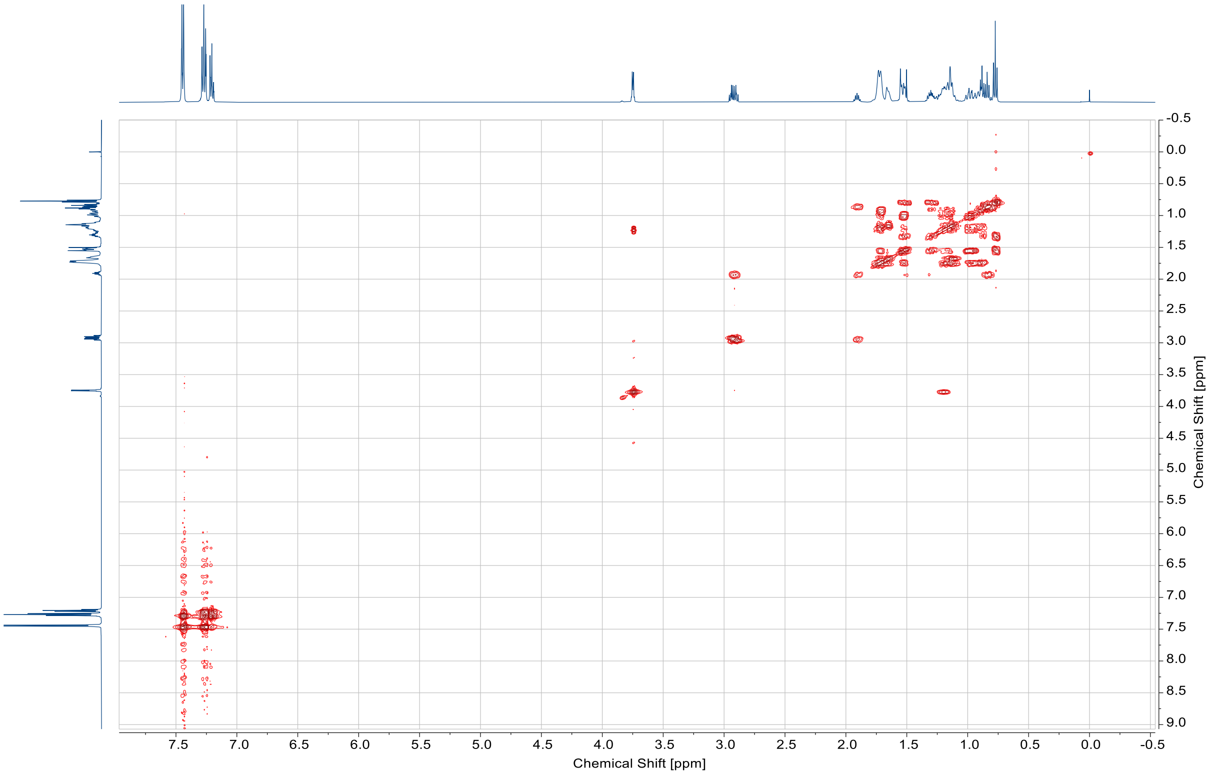

(^{1}H,^{13}C)-HSQC (CDCl$_3$):

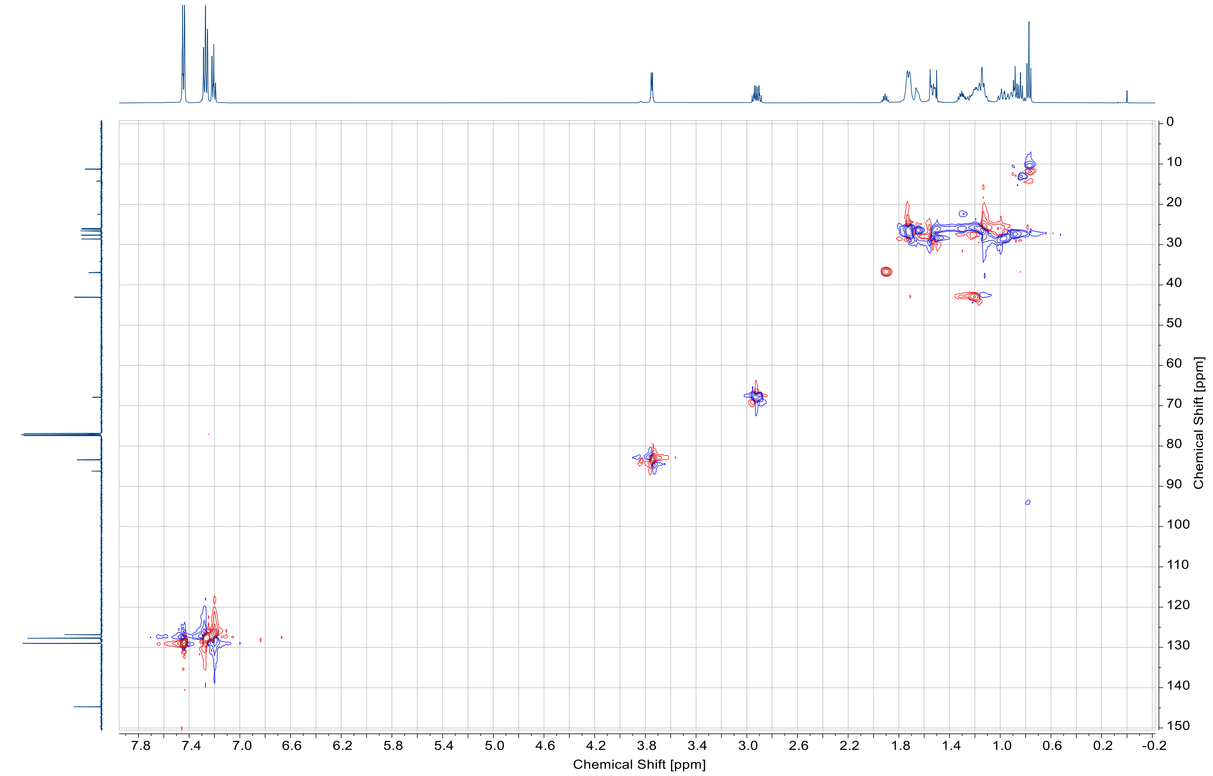

(^{1}H,^{13}C)-HMBC ($CDCl_3$):

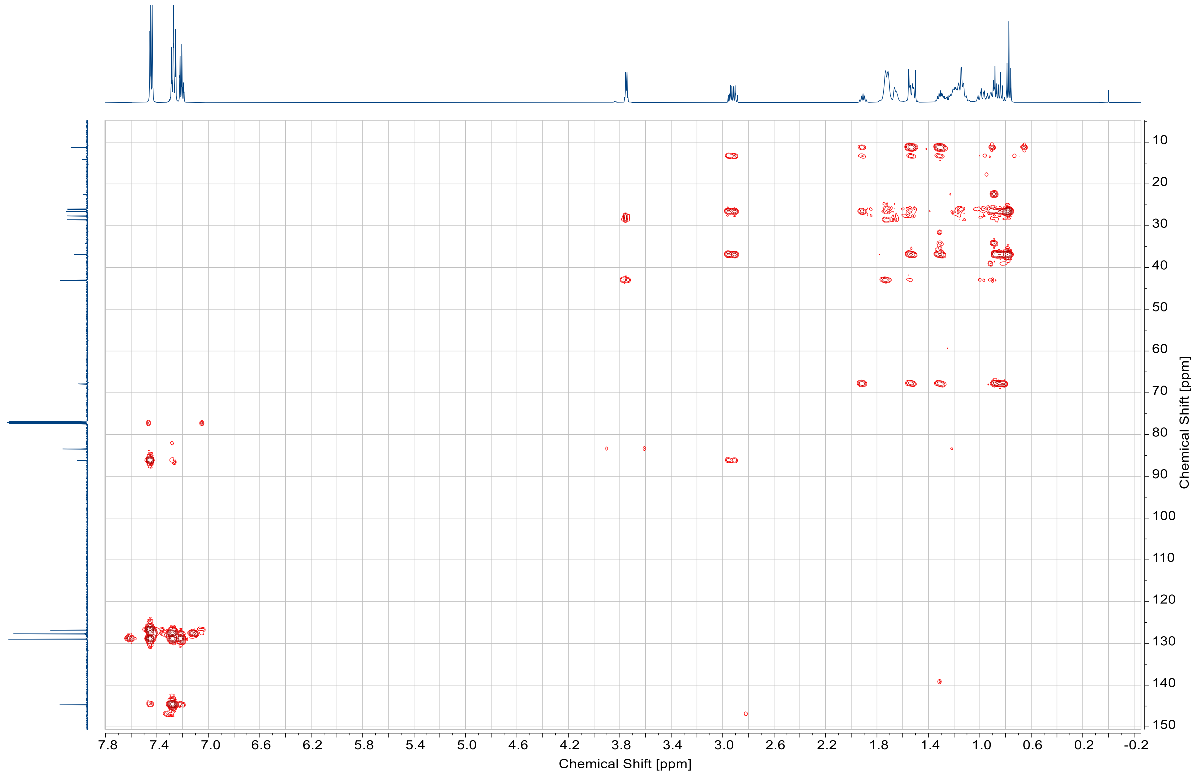

(4*R*,5*R*)-2-[(1*R*,3*R*)-1-Azido-3-methyl-4-(trityloxy)butyl]-4,5-dicyclohexyl-1,3,2-dioxaborolane [6]

^{1}H-NMR (500 MHz, $CDCl_3$):

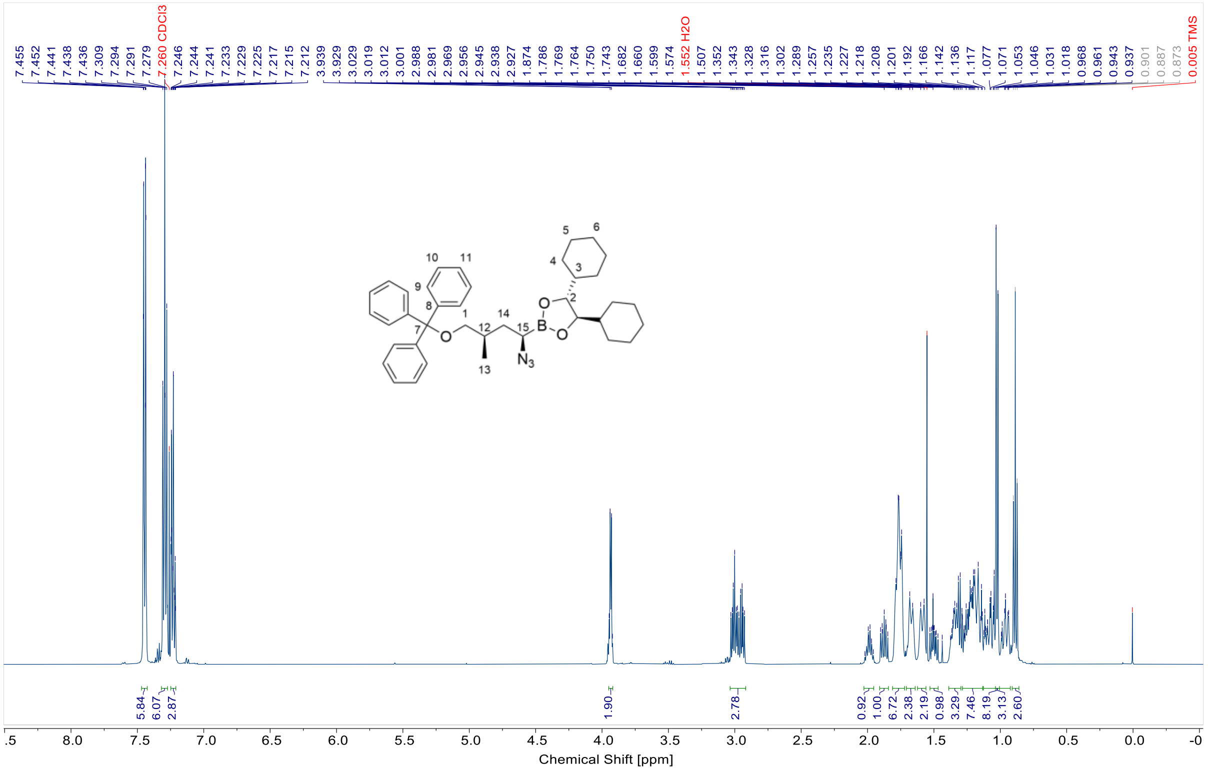

^{13}C-NMR (101 MHz, $CDCl_3$):

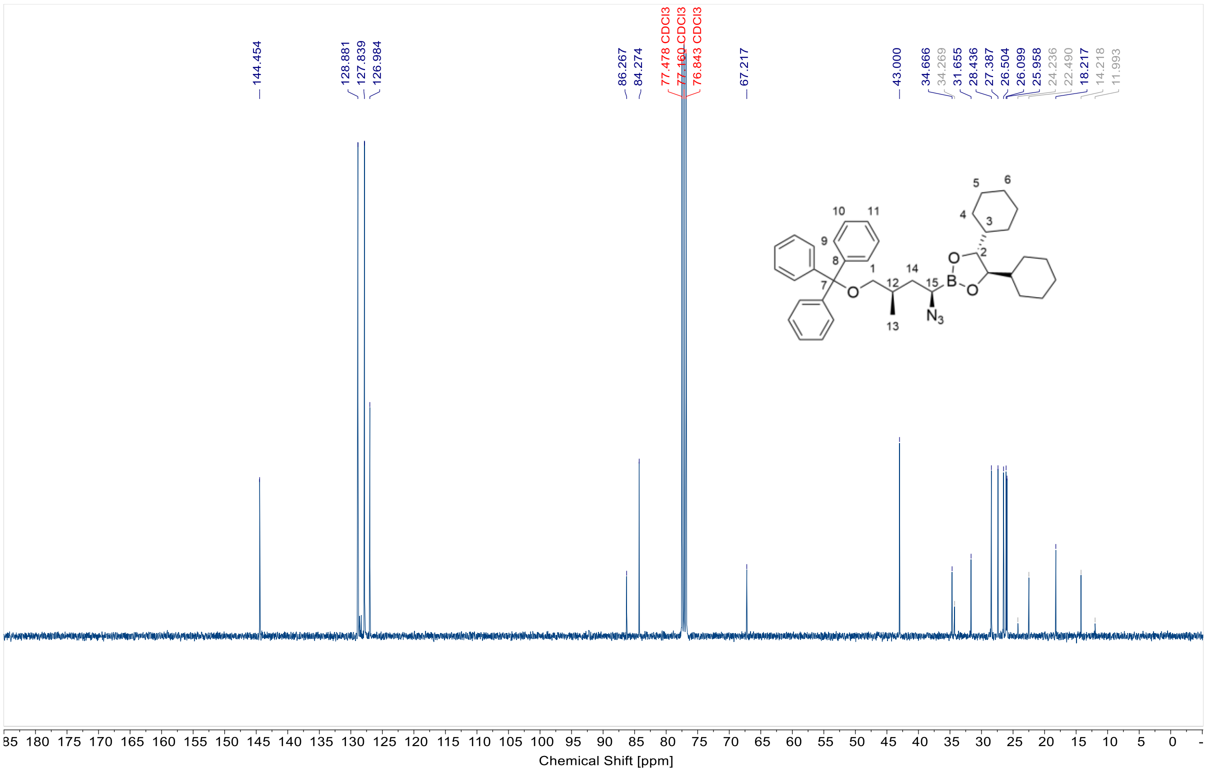

(^{1}H,^{1}H)-COSY ($CDCl_3$):

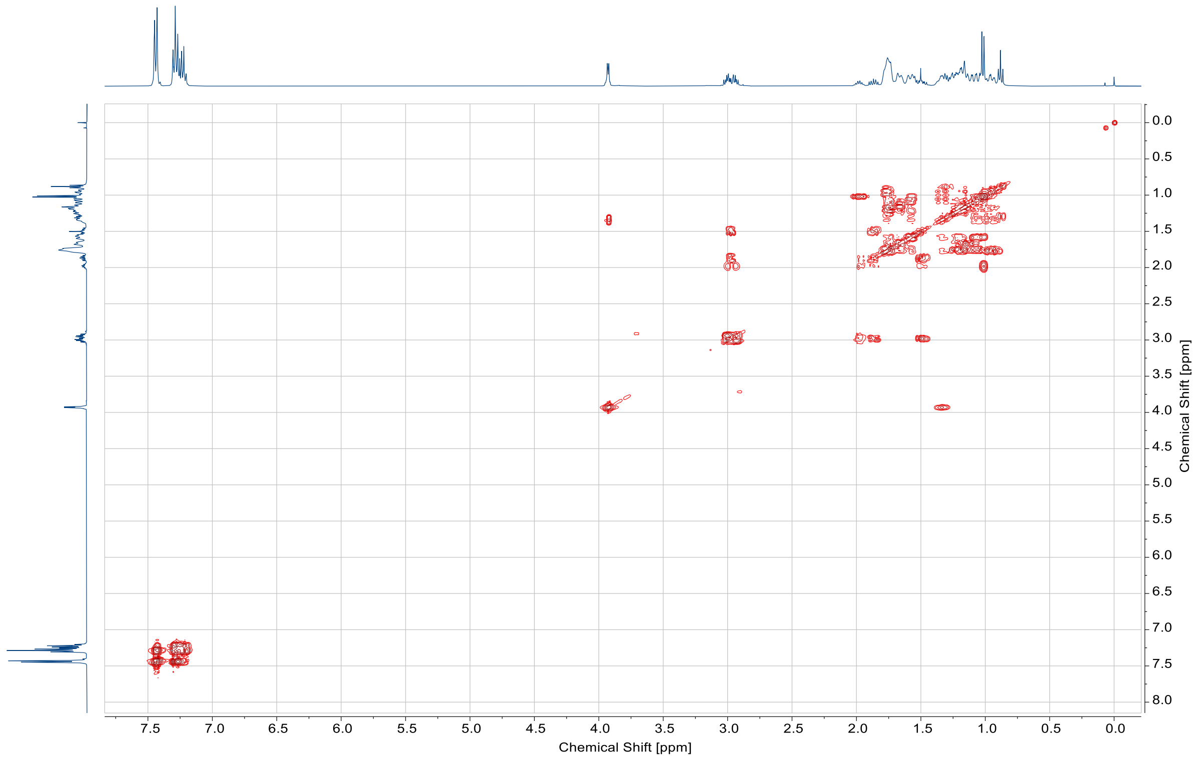

(^{1}H,^{13}C)-HSQC (CDCl$_3$):

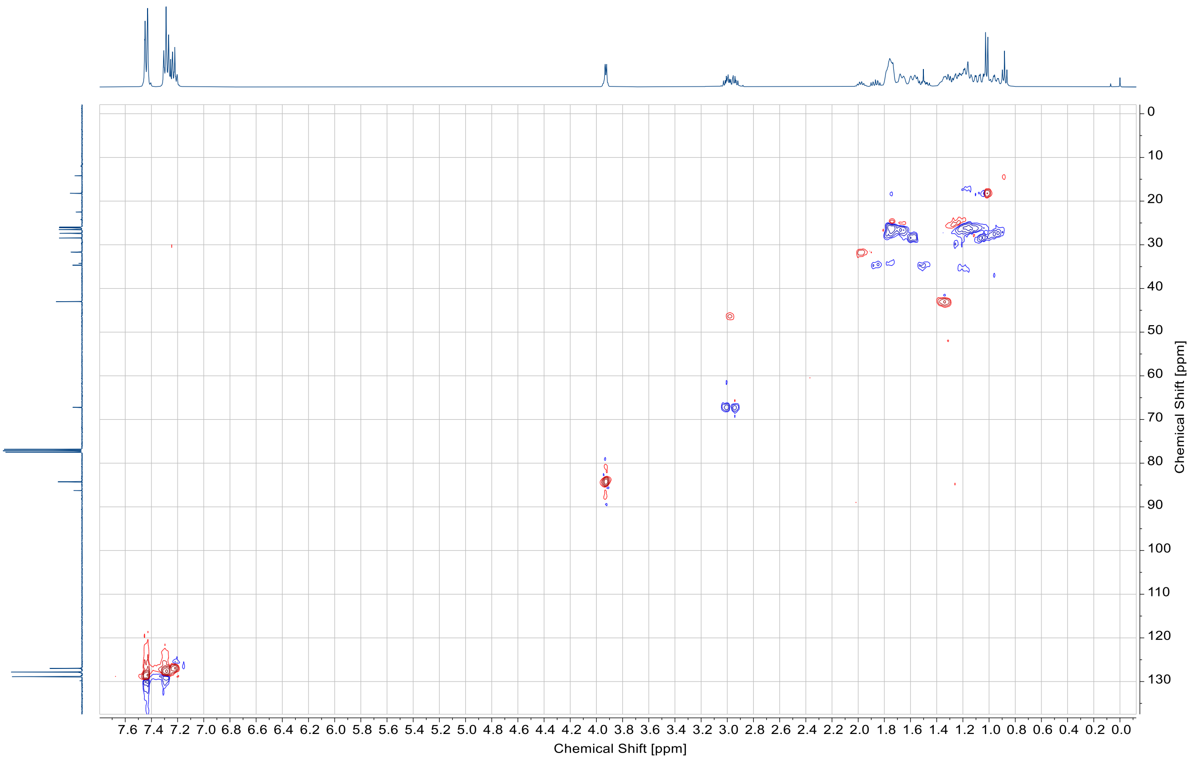

(^{1}H,^{13}C)-HMBC (CDCl$_3$):

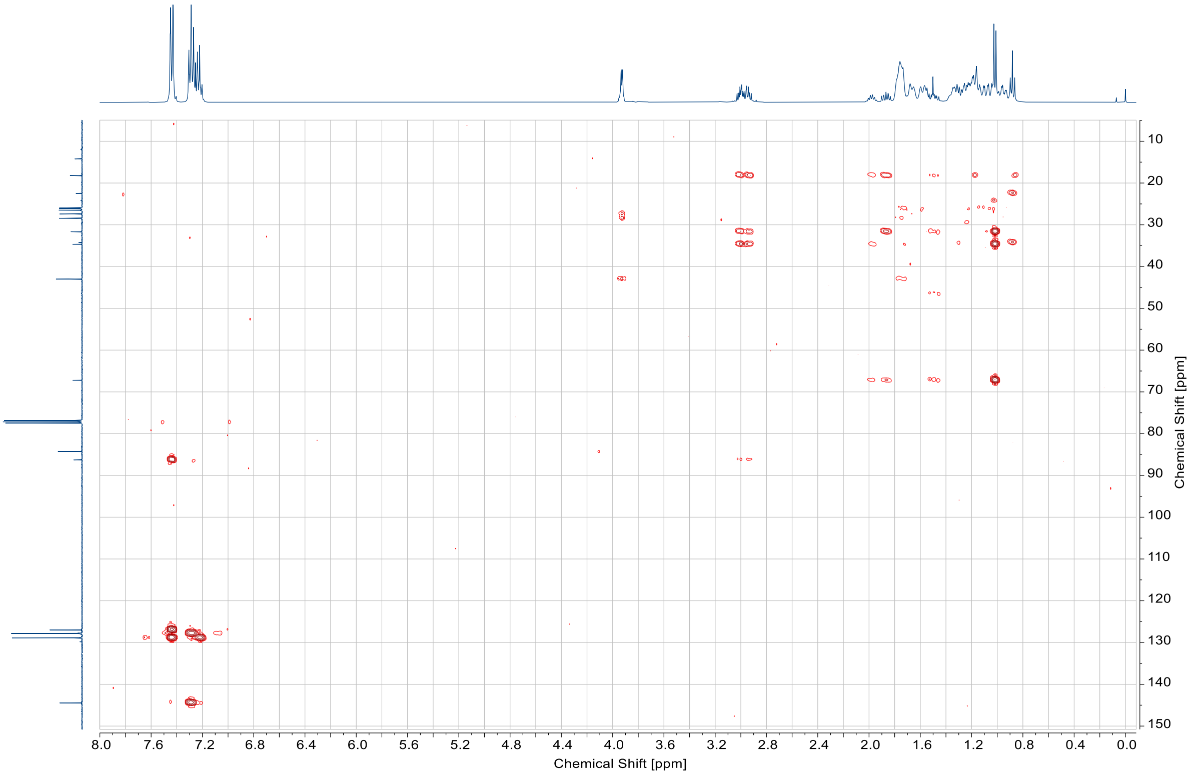

(4*R*,5*R*)-2-[(1*R*,3*R*)-1-Azido-3-((trityloxy)methyl)pentyl]-4,5-dicyclohexyl-1,3,2-dioxaborolane [7]

^{1}H-NMR (500 MHz, $CDCl_3$):

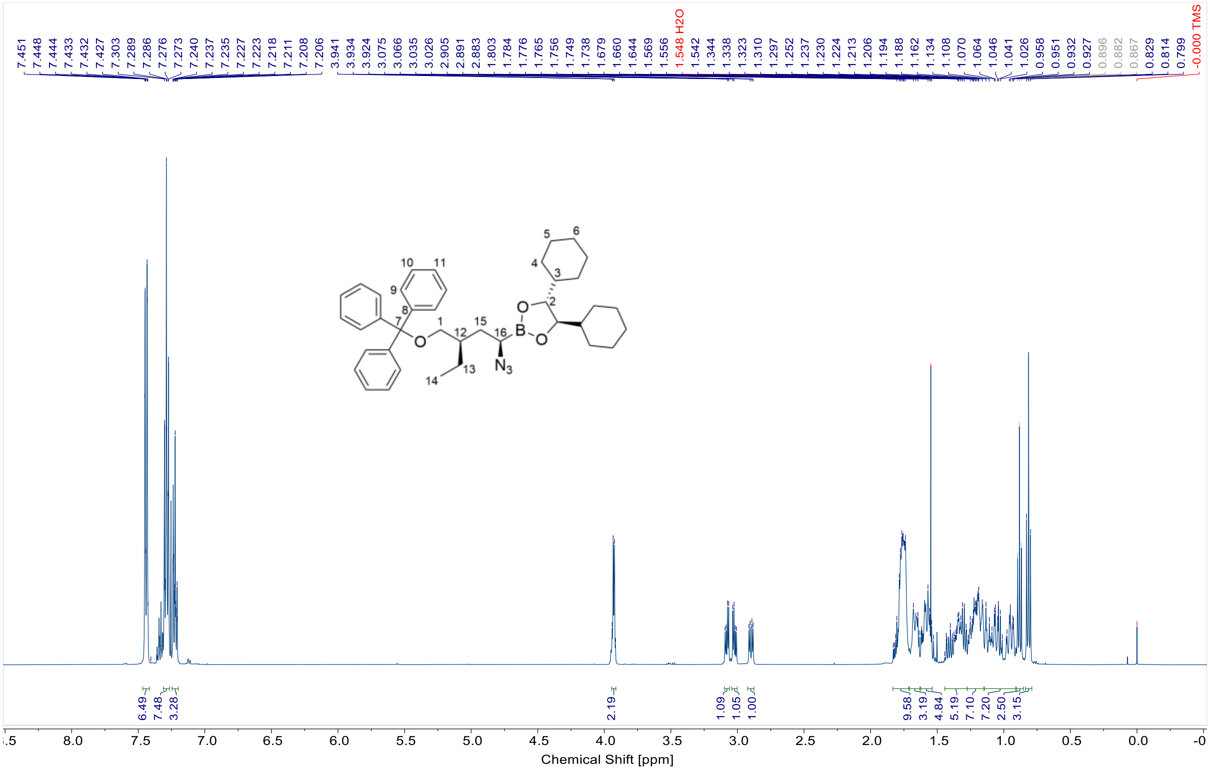

^{13}C-NMR (126 MHz, $CDCl_3$):

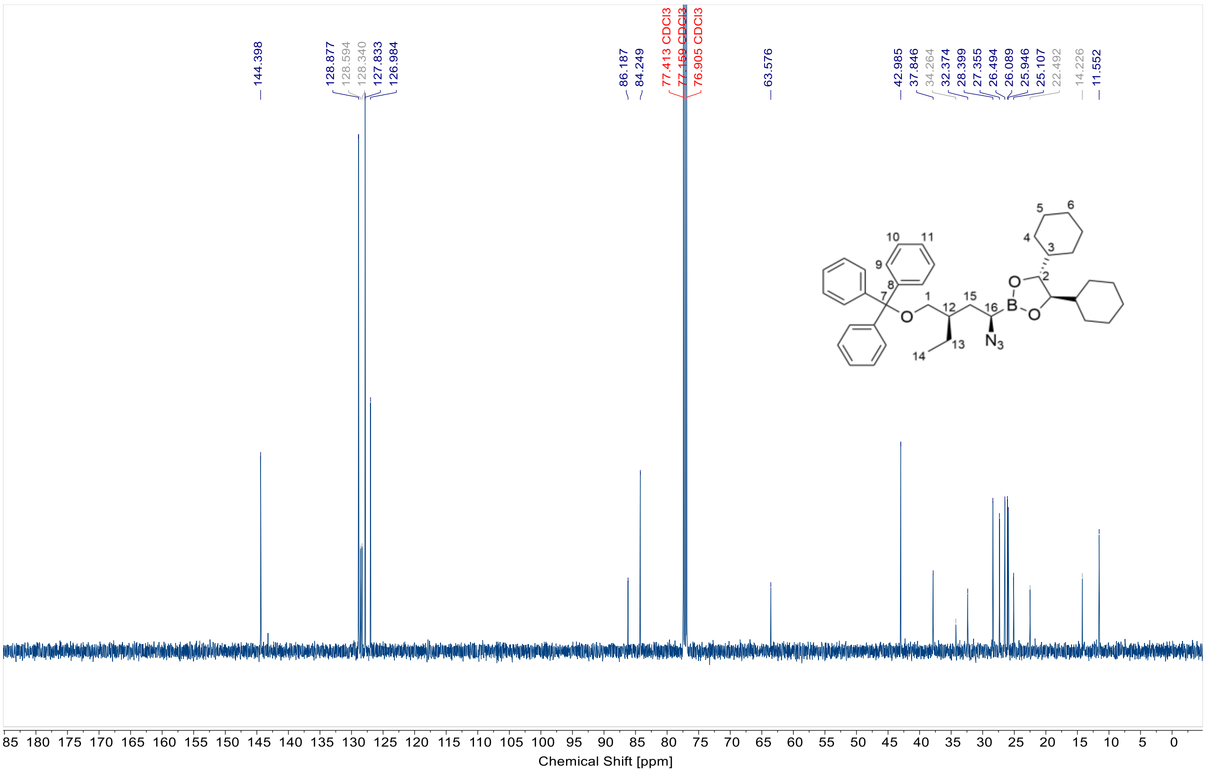

(^{1}H,^{1}H)-COSY ($CDCl_3$):

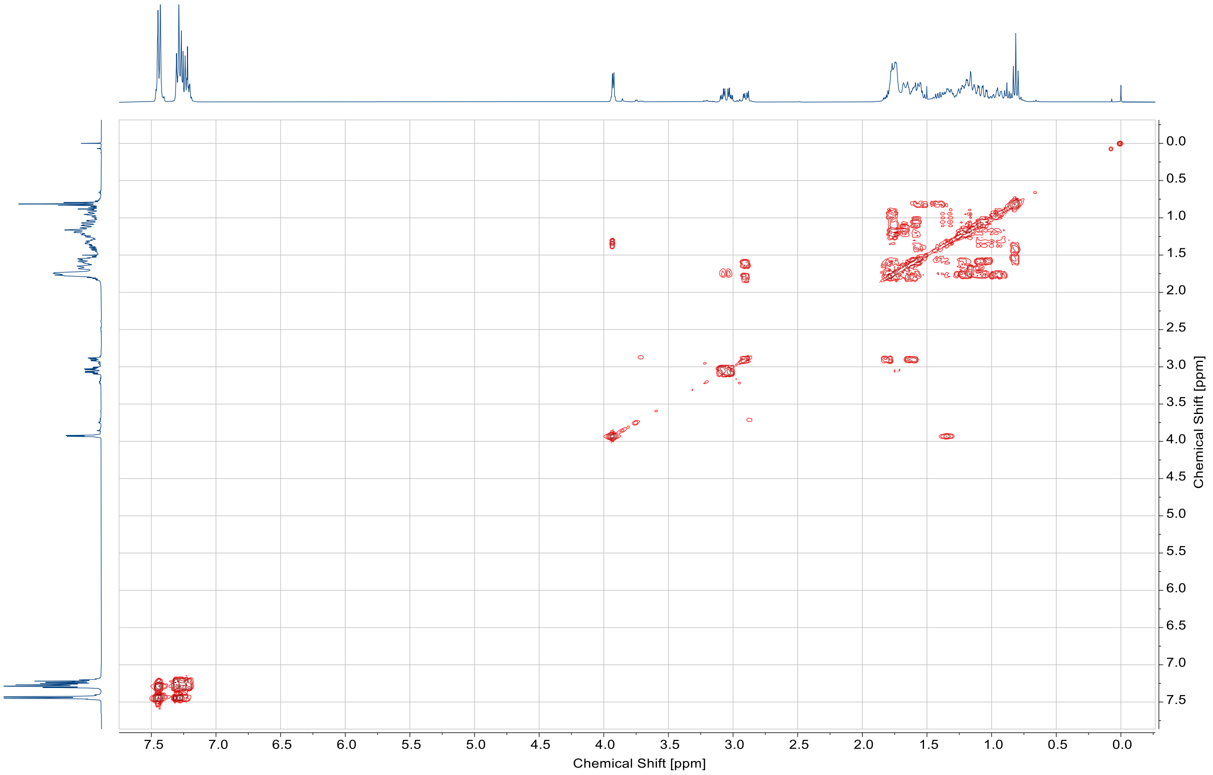

(^{1}H,^{13}C)-HSQC ($CDCl_3$):

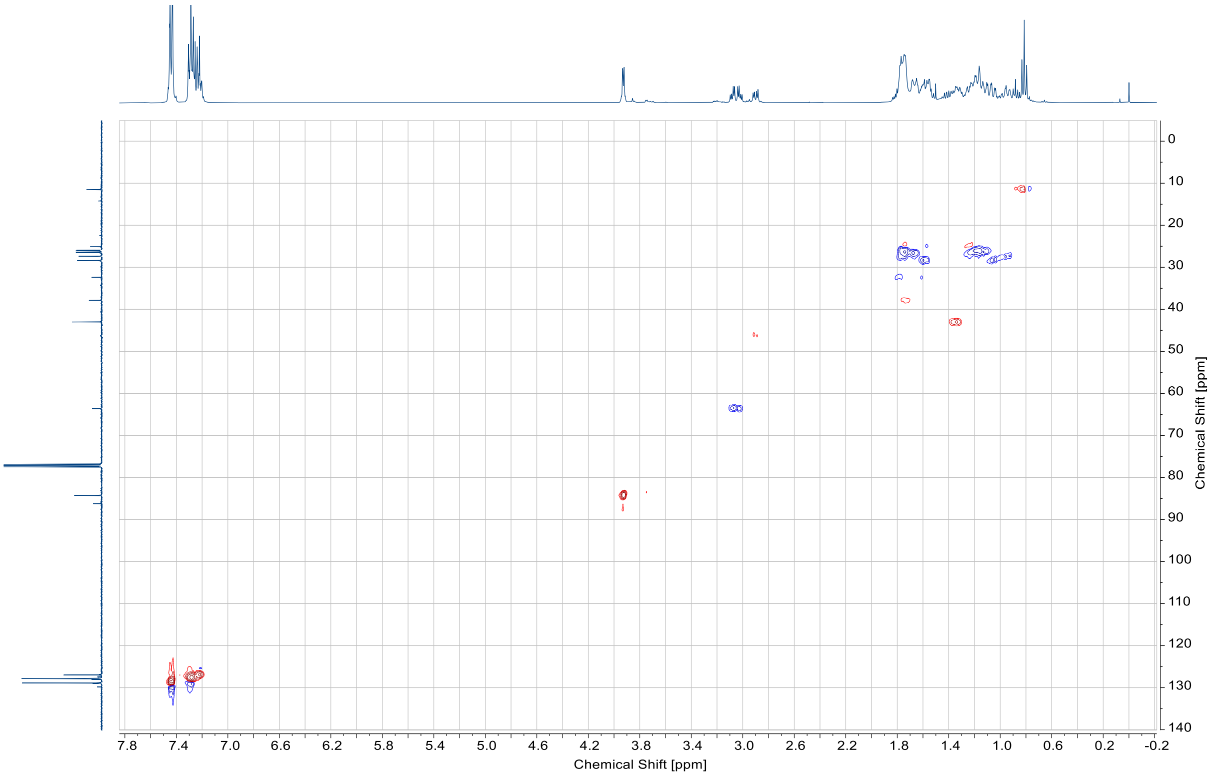

(^{1}H,^{13}C)-HMBC ($CDCl_3$):

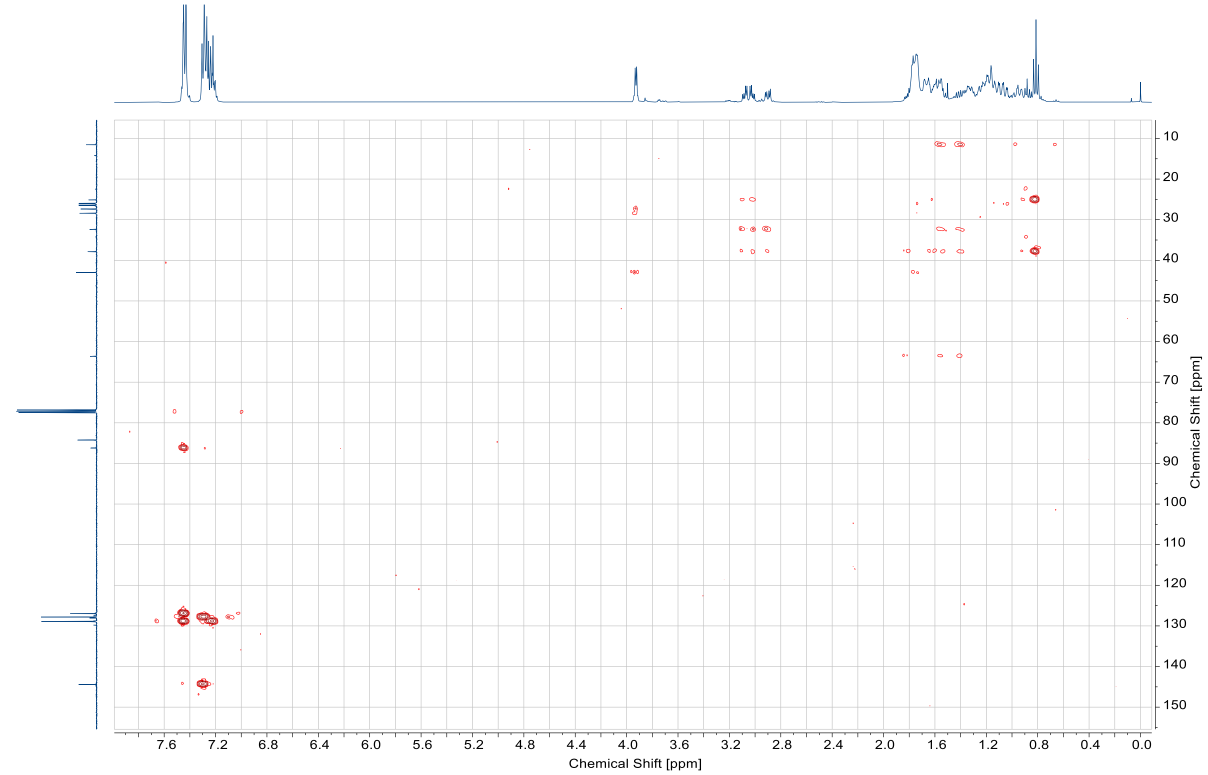

tert-Butyl (2*S*,4*R*)-2-Azido-4-methyl-5-(trityloxy)pentanoate [8]

^{1}H-NMR (400 MHz, $CDCl_3$):

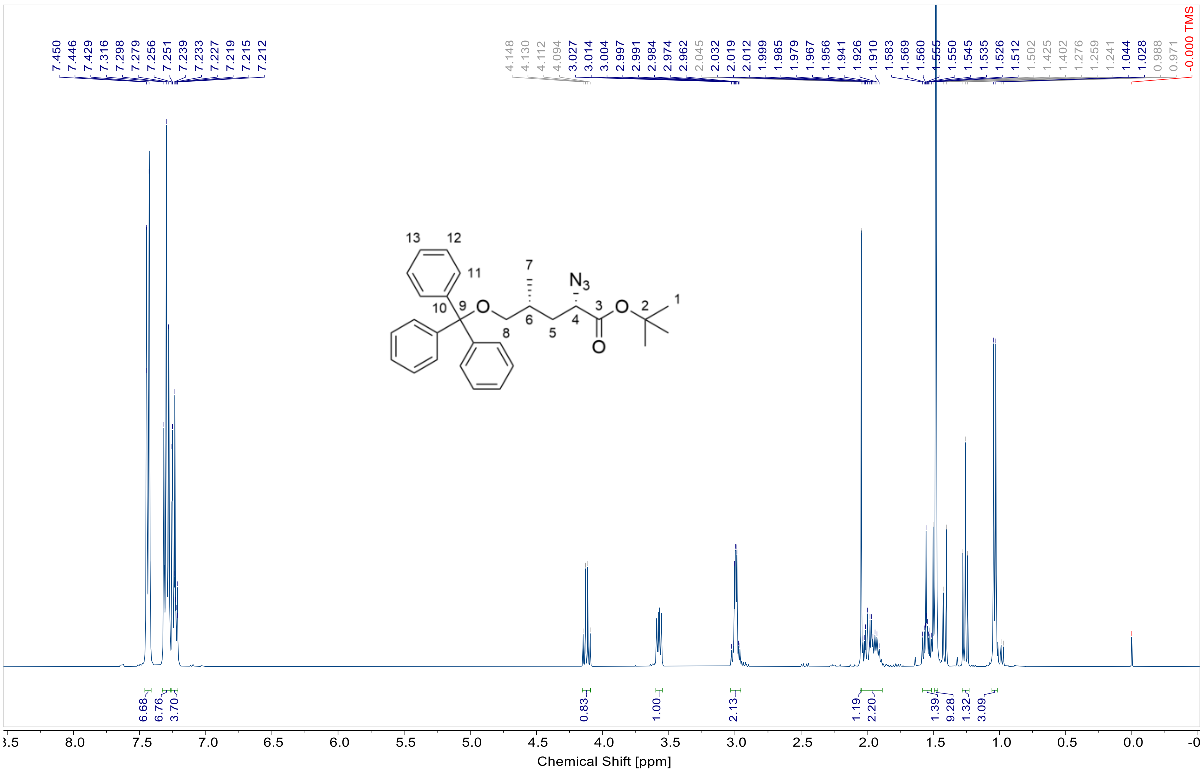

^{13}C-NMR (126 MHz, $CDCl_3$):

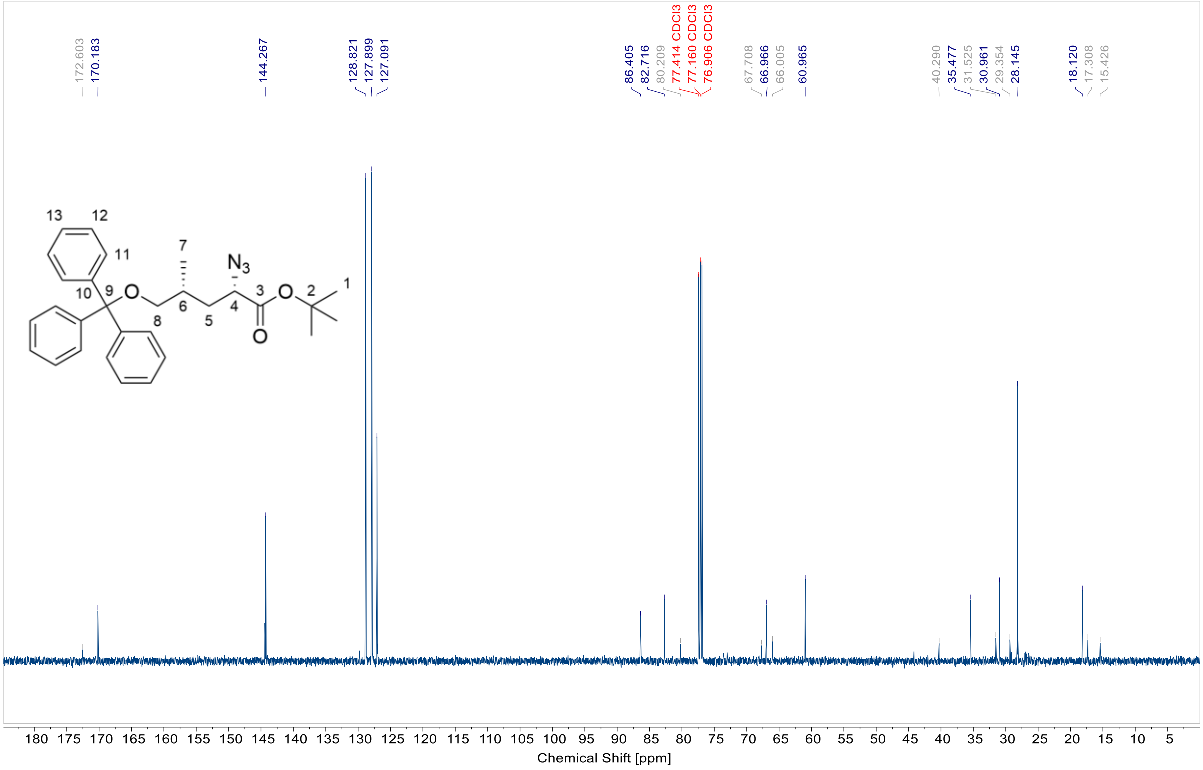

(^{1}H,^{1}H)-COSY ($CDCl_3$):

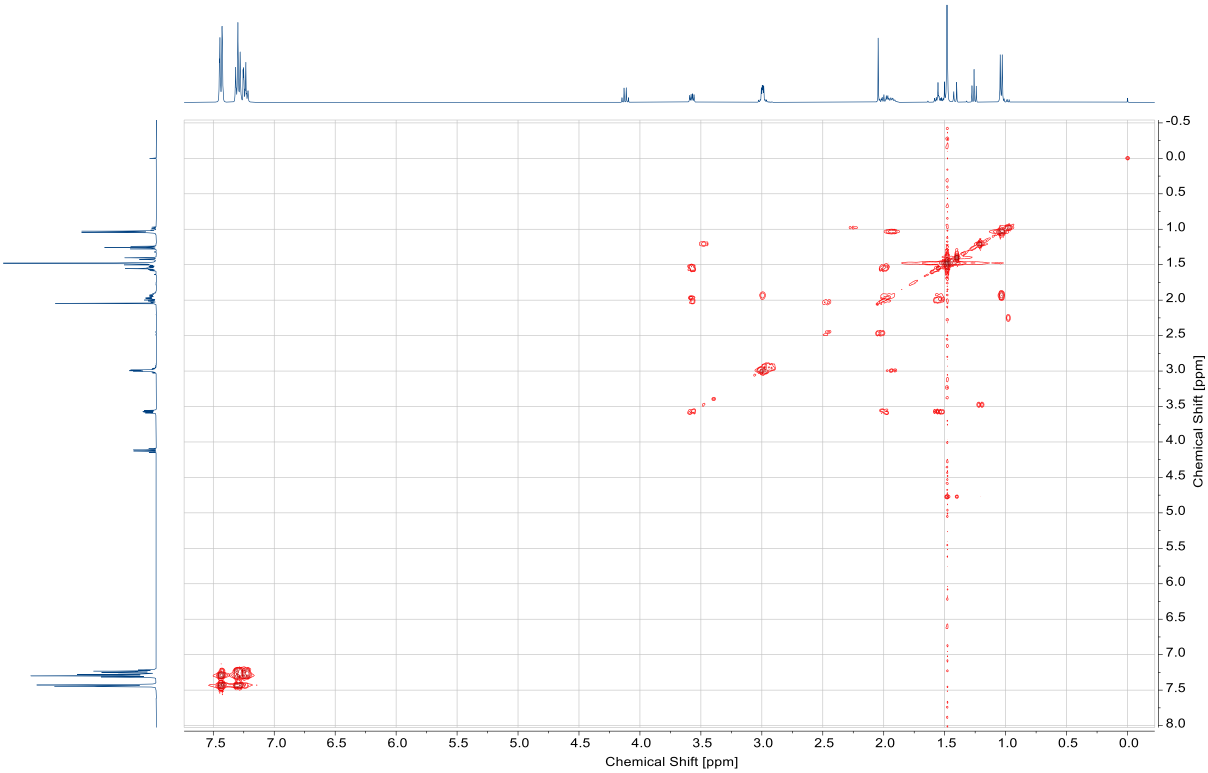

(^{1}H,^{13}C)-HSQC ($CDCl_3$):

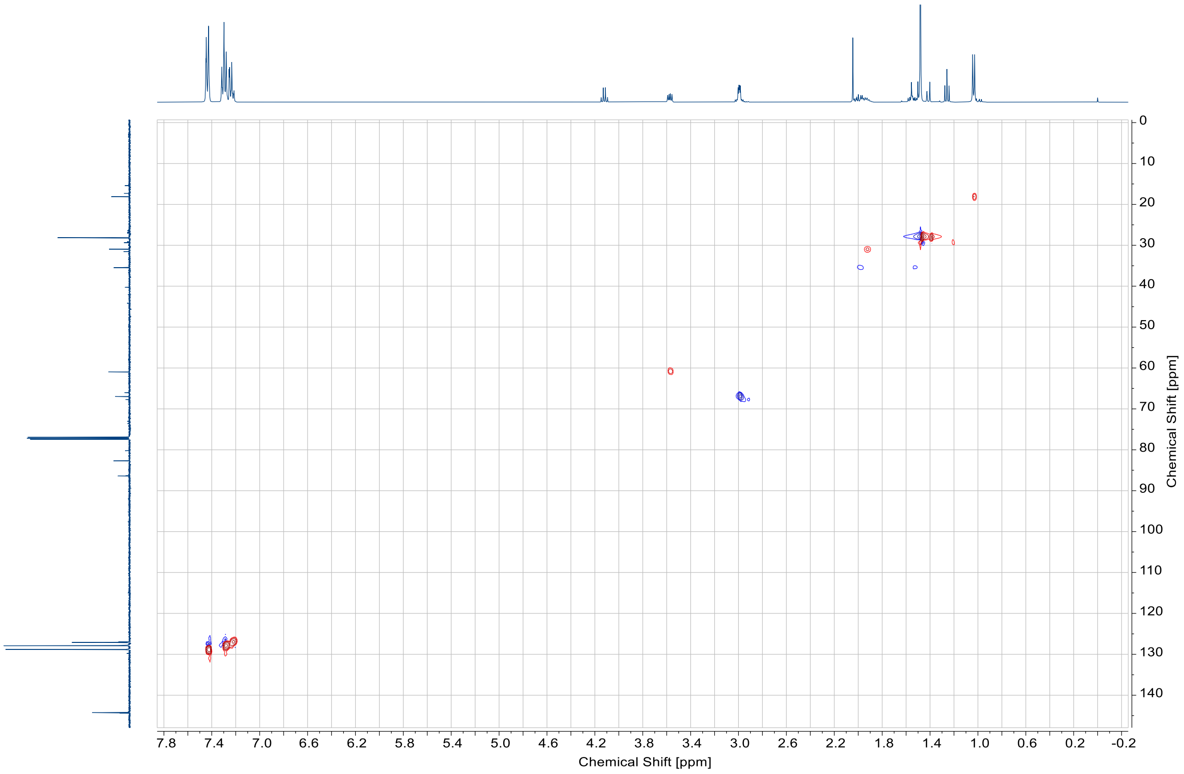

(^{1}H,^{13}C)-HMBC ($CDCl_3$):

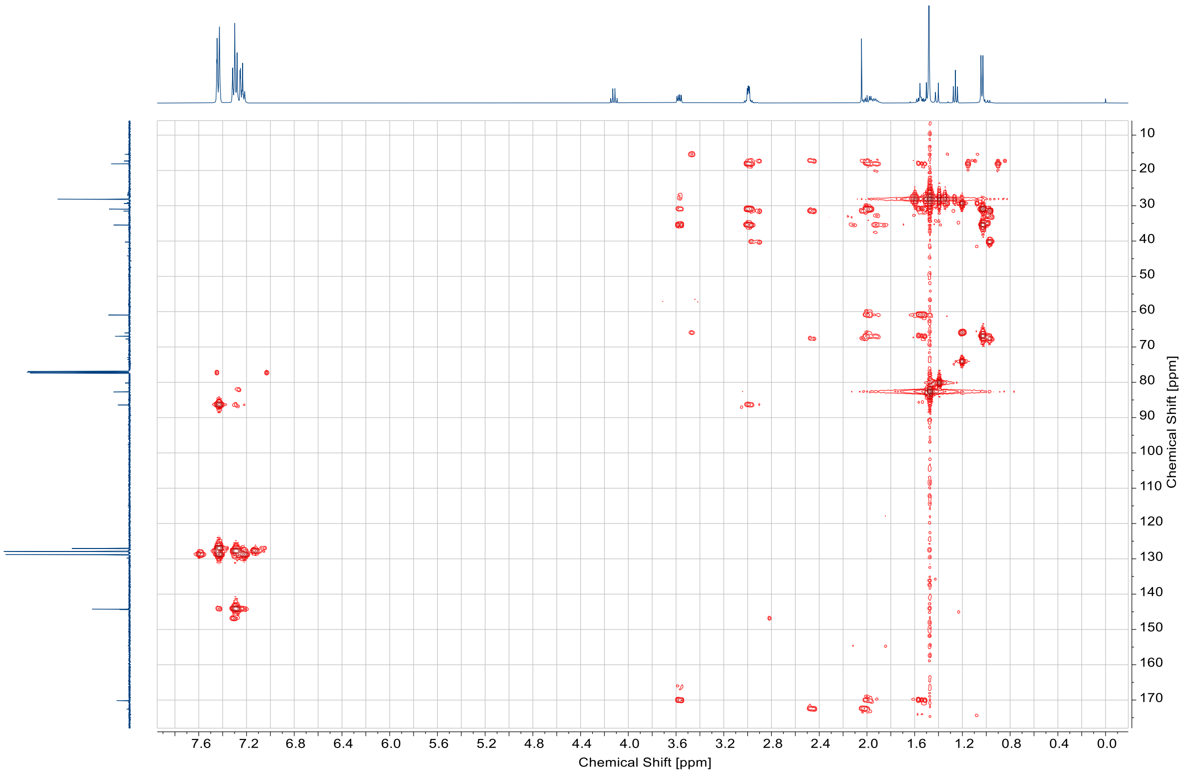

tert-Butyl (2*S*,4*R*)-2-Azido-4-((trityloxy)methyl)hexanoate [9]

^{1}H-NMR (400 MHz, $CDCl_3$):

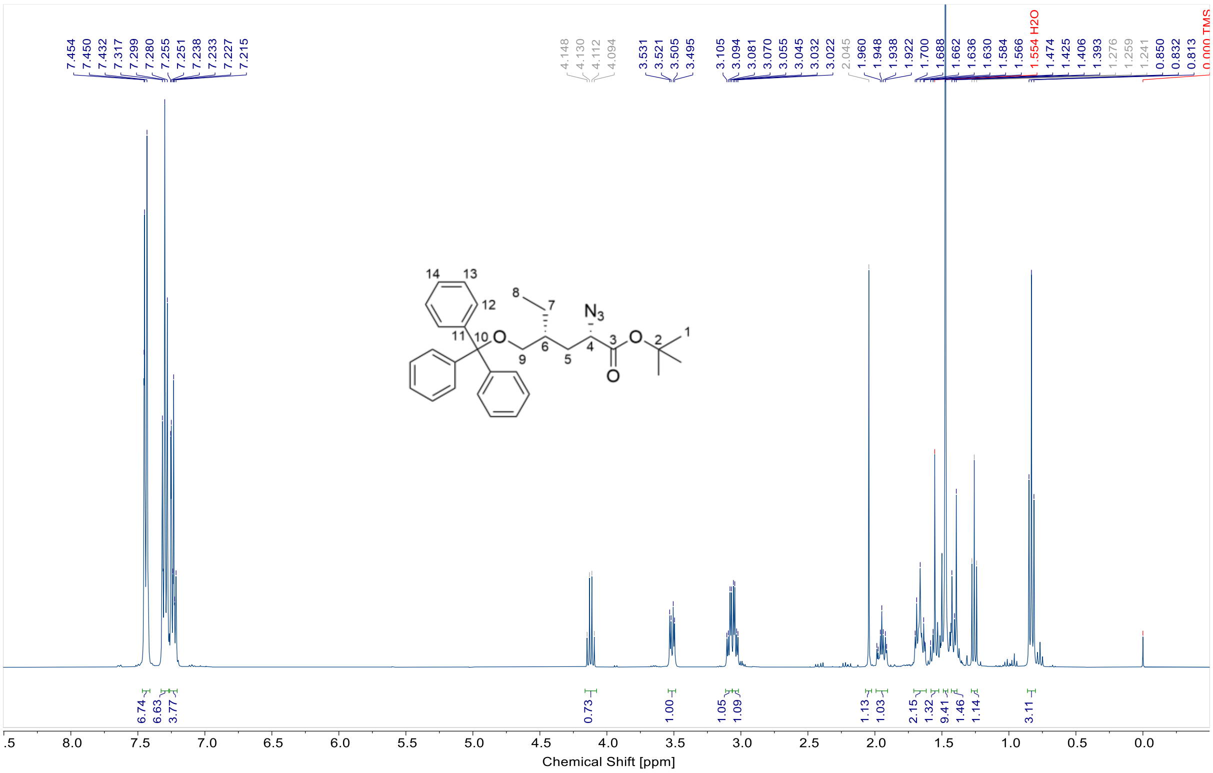

^{13}C-NMR (101 MHz, $CDCl_3$):

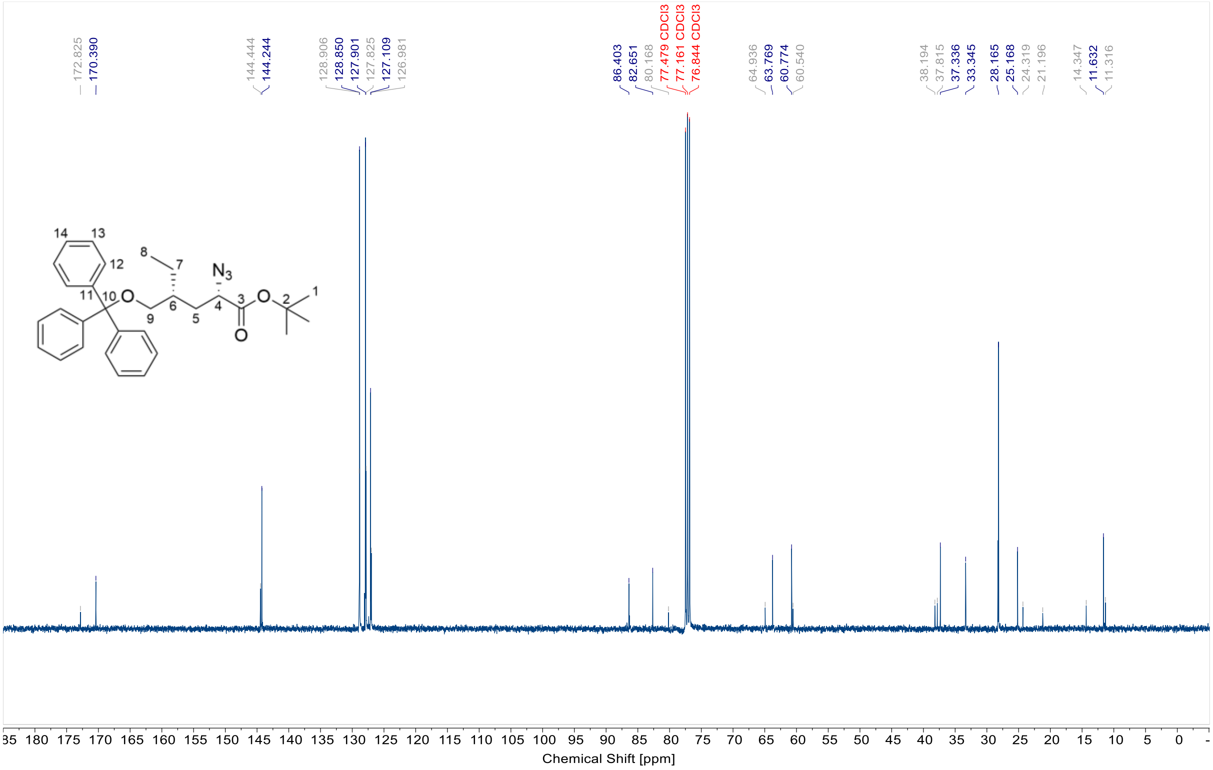

(^{1}H,^{1}H)-COSY (CDCl$_3$):

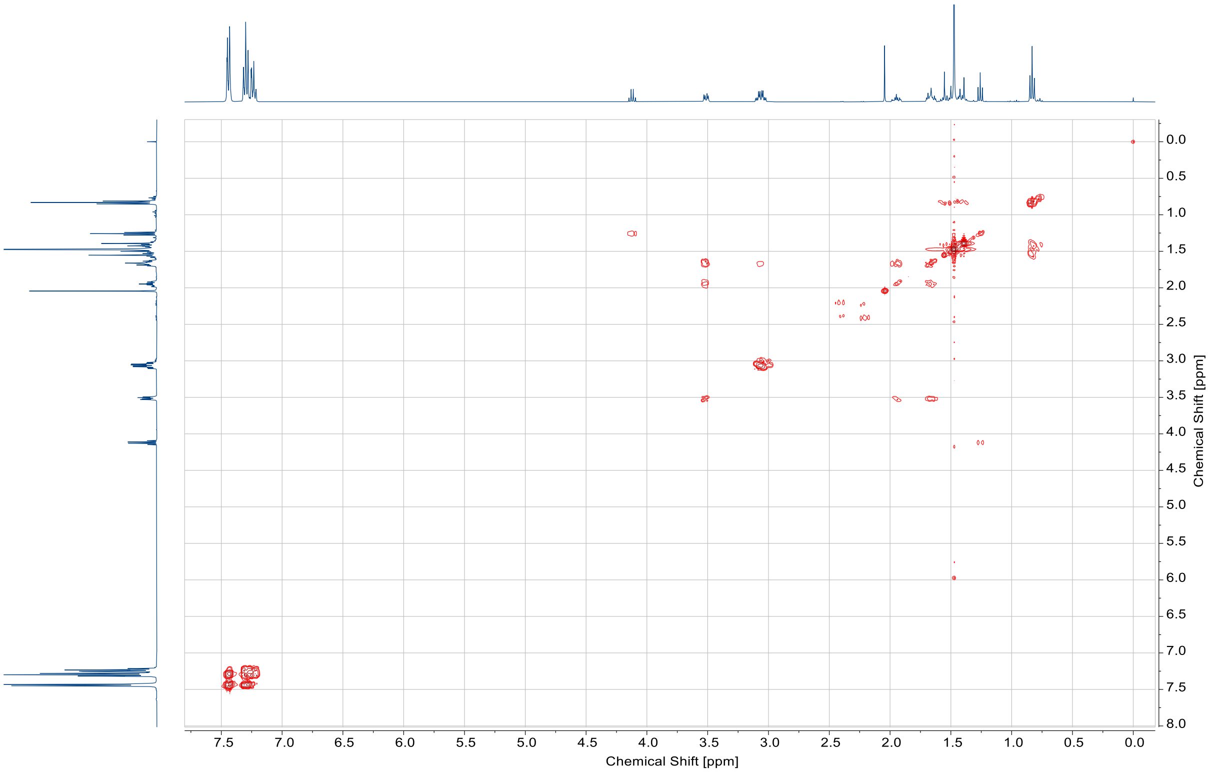

(^{1}H,^{13}C)-HSQC (CDCl$_3$):

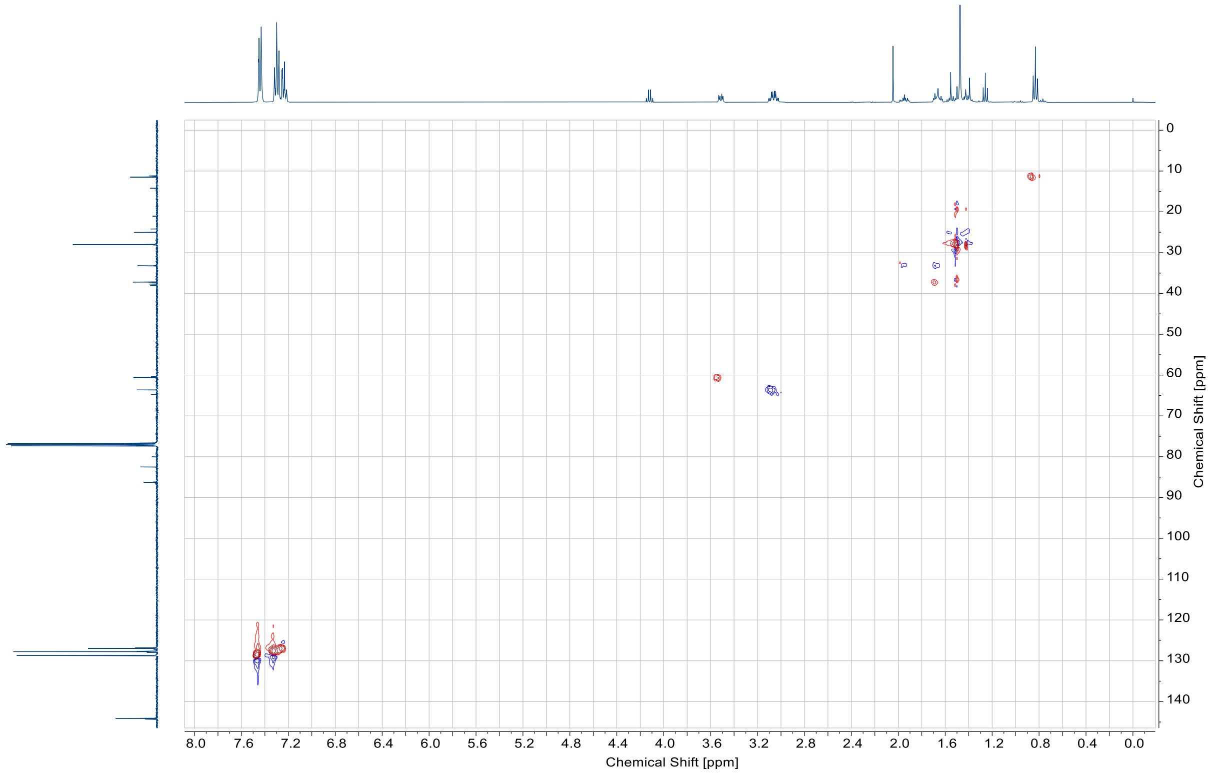

(^{1}H,^{13}C)-HMBC (CDCl$_3$):

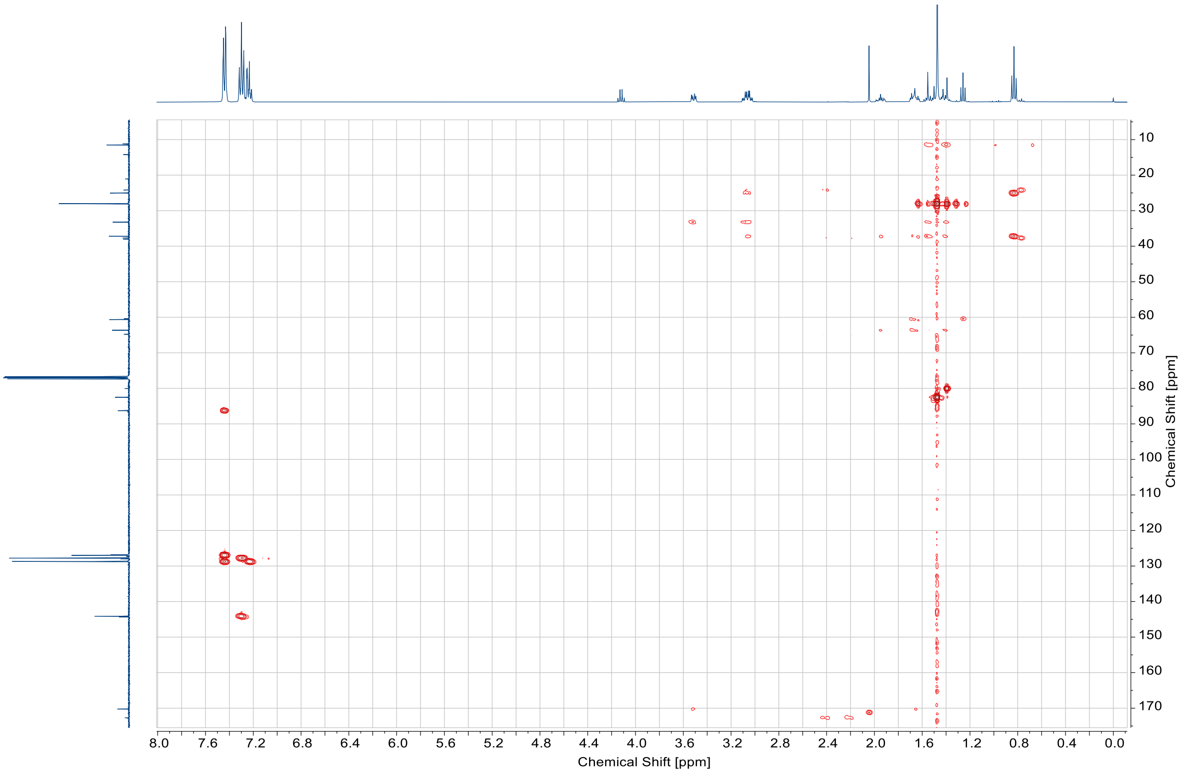

***tert*-Butyl (2*S*,4*R*)-2-Azido-5-hydroxy-4-methylpentanoate [10’]**

^{1}H-NMR (500 MHz, CDCl$_3$):

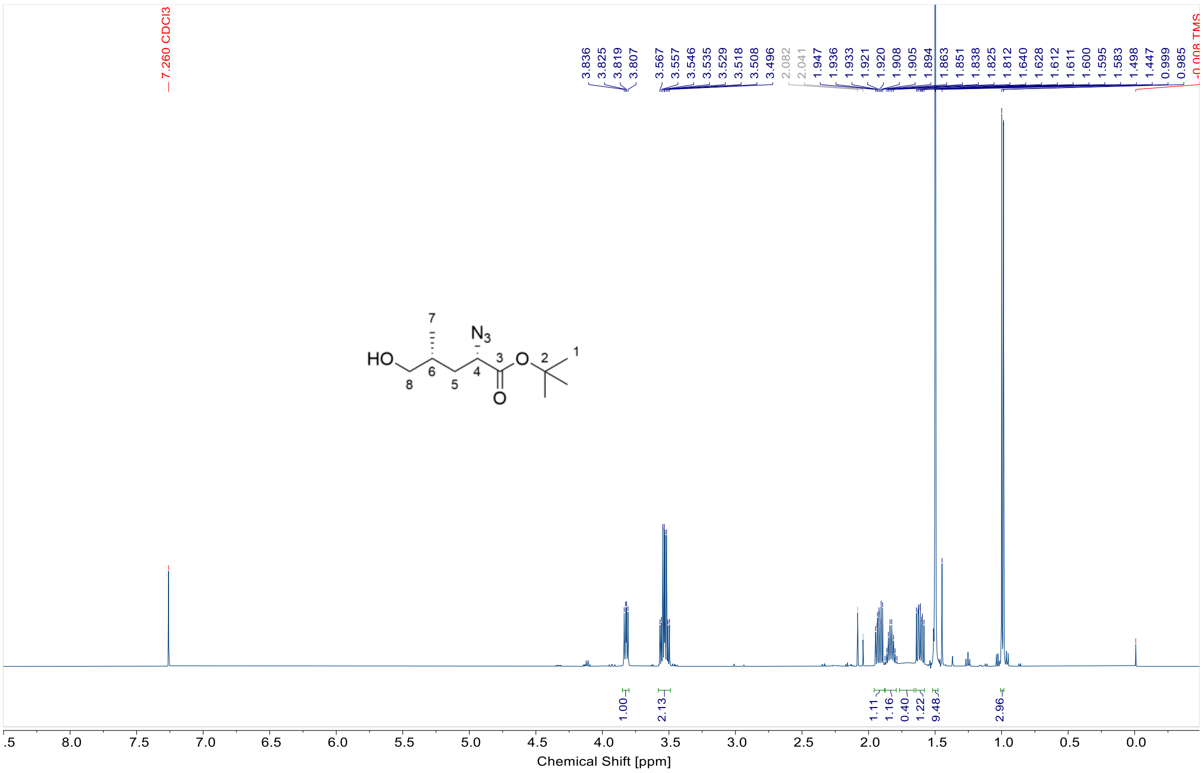

^{13}C-NMR (126 MHz, $CDCl_3$):

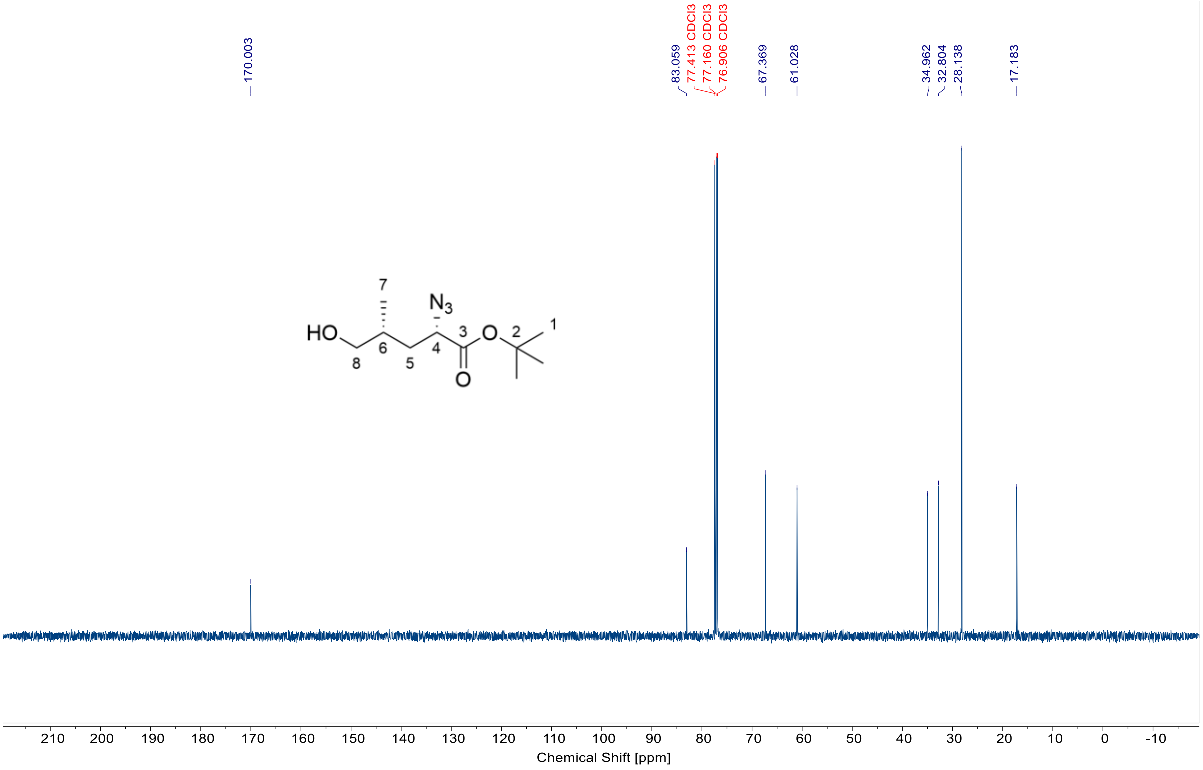

(^{1}H,^{1}H)-COSY ($CDCl_3$):

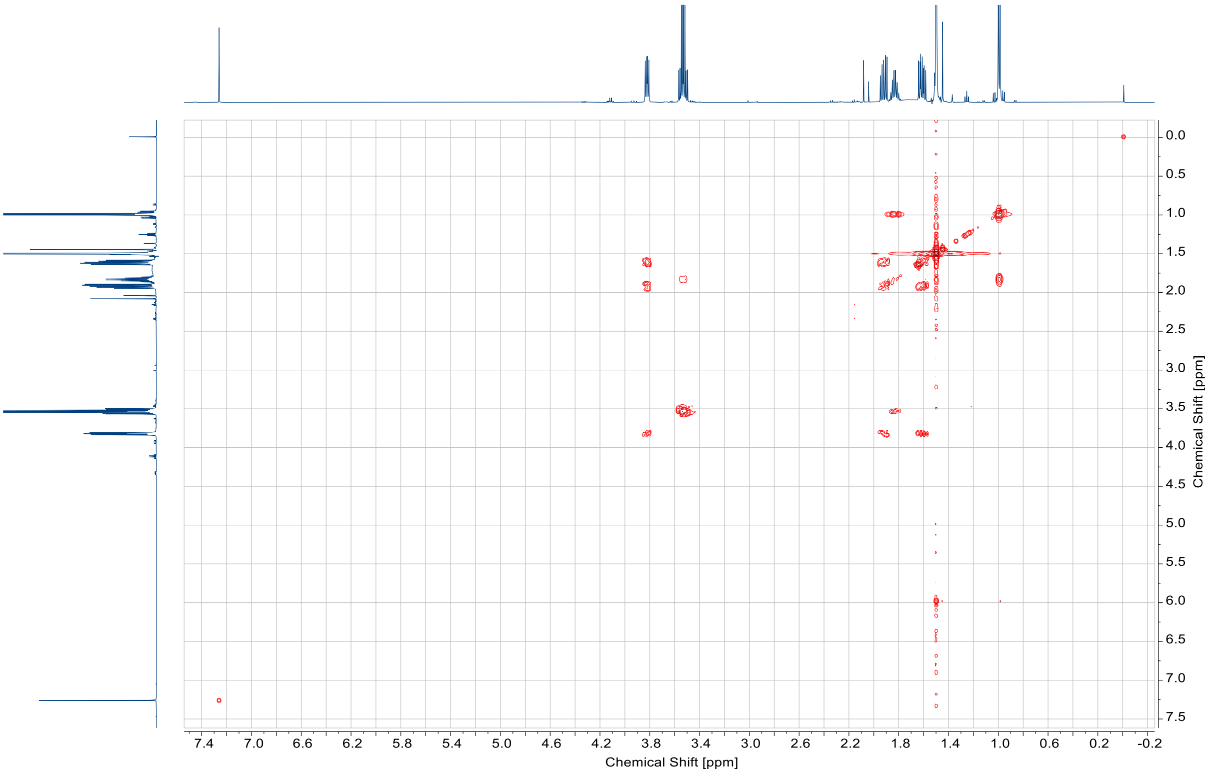

(^{1}H,^{13}C)-HSQC (CDCl$_3$):

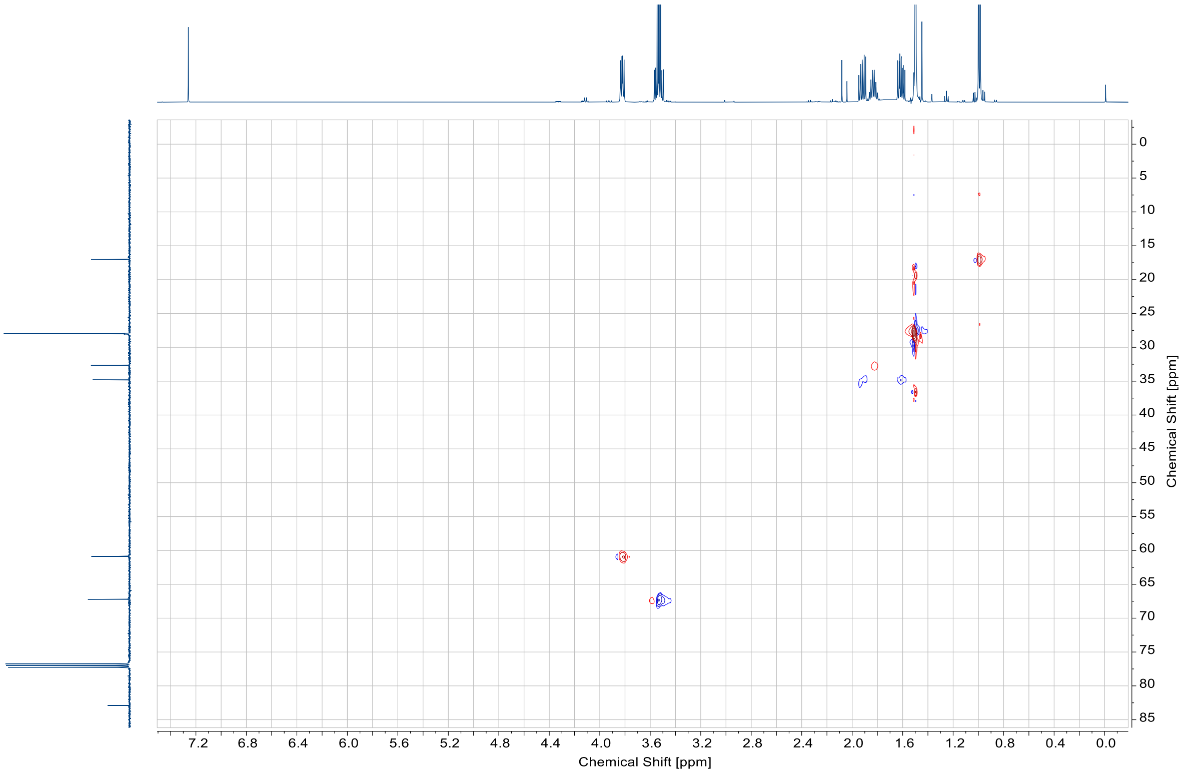

(^{1}H,^{13}C)-HMBC (CDCl$_3$):

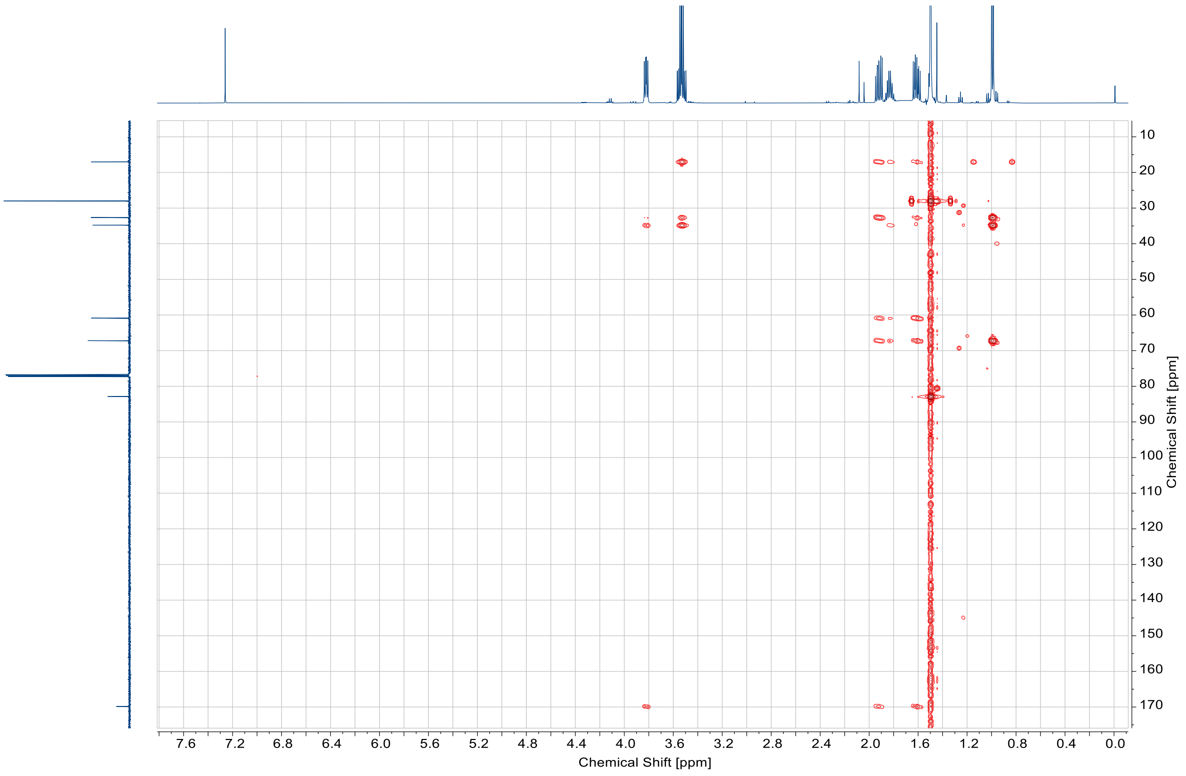

***tert*-Butyl (2*S*,4*R*)-2-Azido-4-(hydroxymethyl)hexanoate [11']**

^{1}H-NMR (500 MHz, $CDCl_3$):

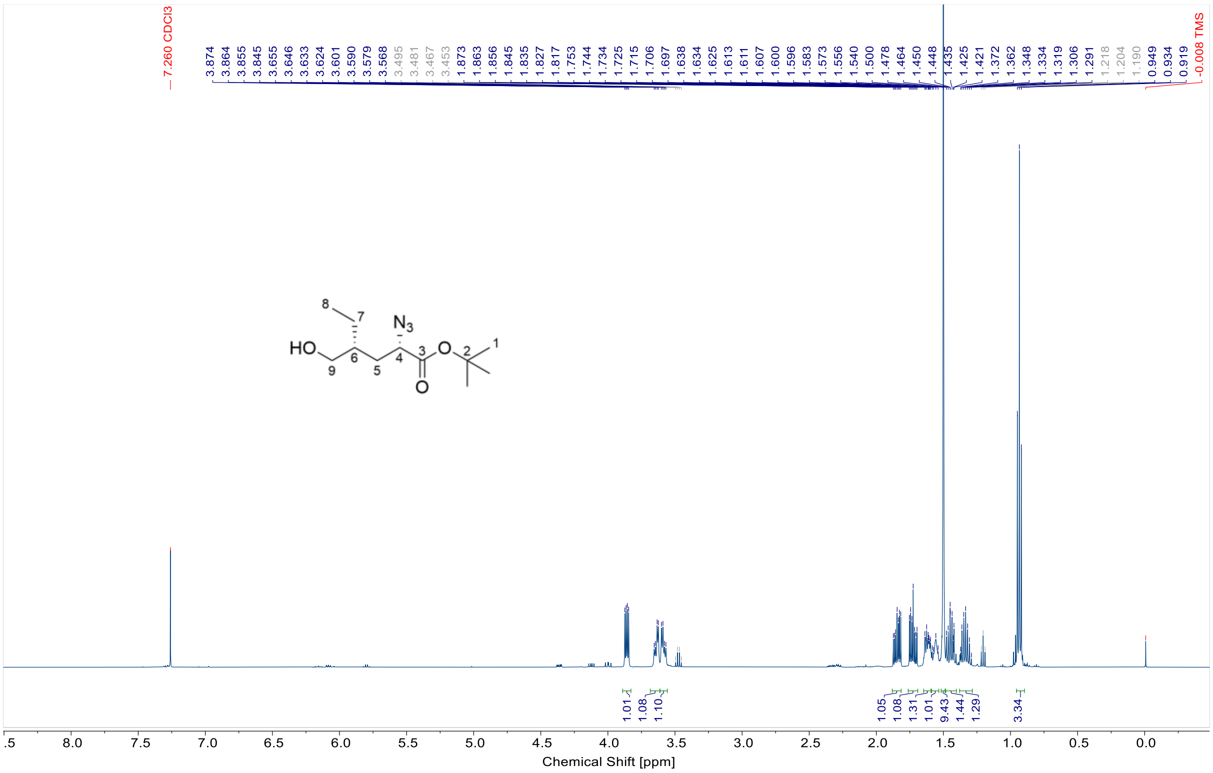

^{13}C-NMR (126 MHz, $CDCl_3$):

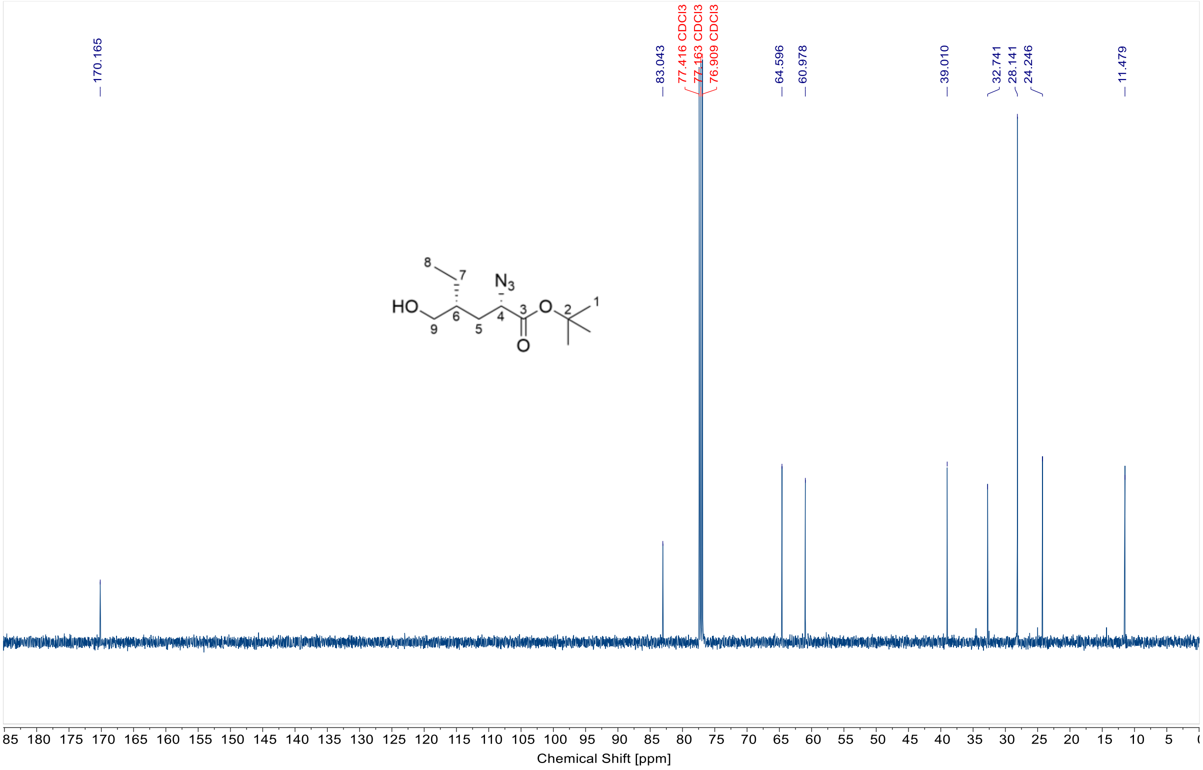

(^{1}H,^{1}H)-COSY ($CDCl_3$):

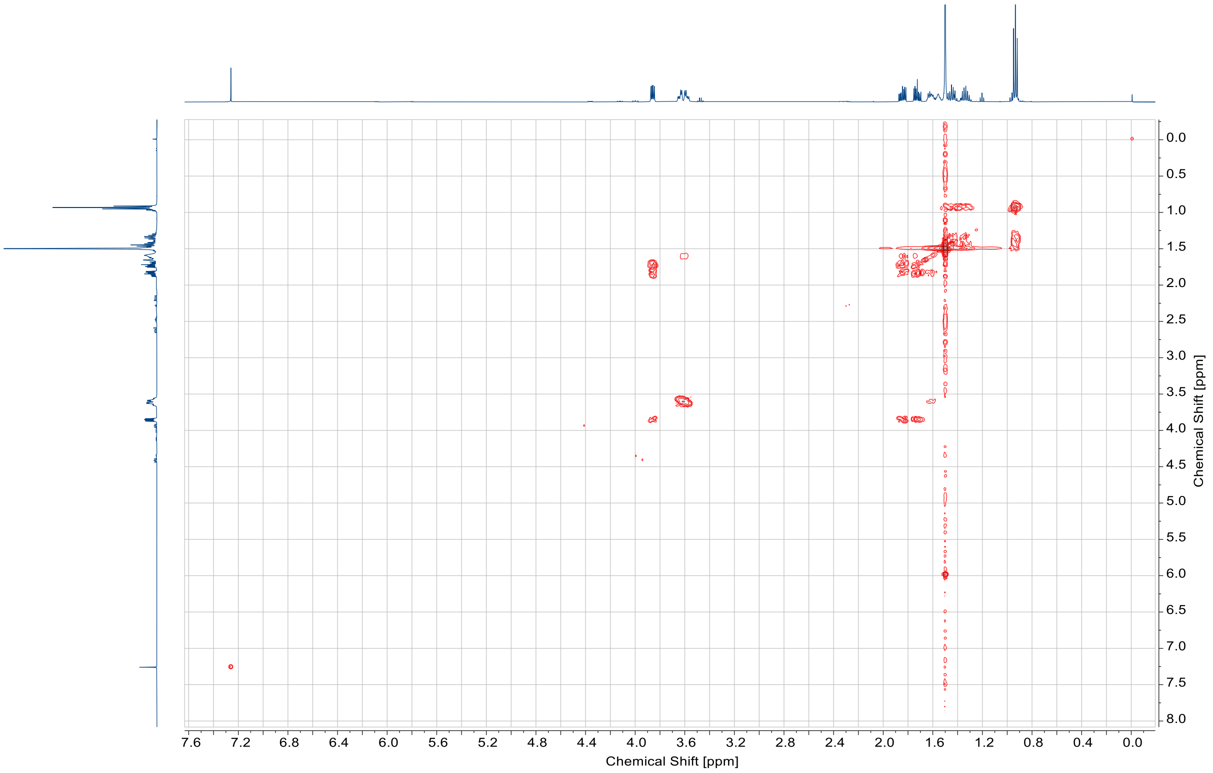

(^{1}H,^{13}C)-HSQC ($CDCl_3$):

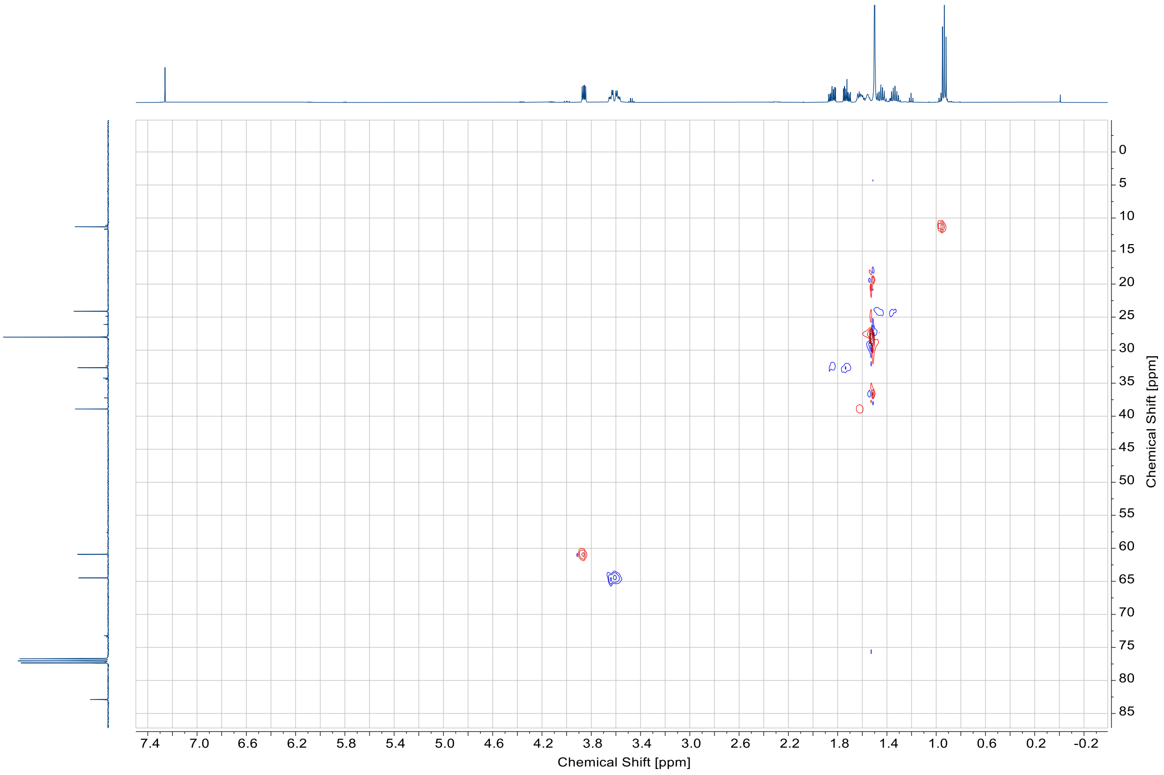

(¹H,¹³C)-HMBC ($CDCl_3$):

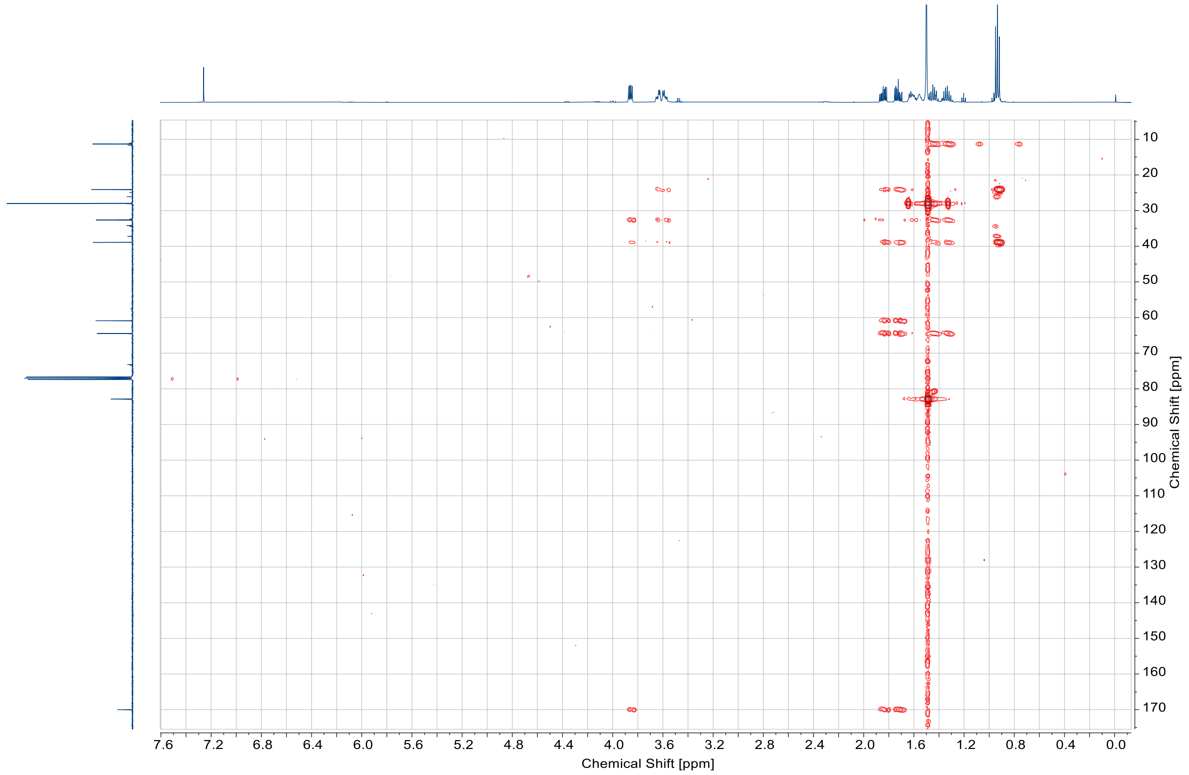

tert-Butyl (2*S*,4*R*)-2-Azido-4-methyl-5-(tosyloxy)pentanoate [10]

¹H-NMR (500 MHz, $CDCl_3$):

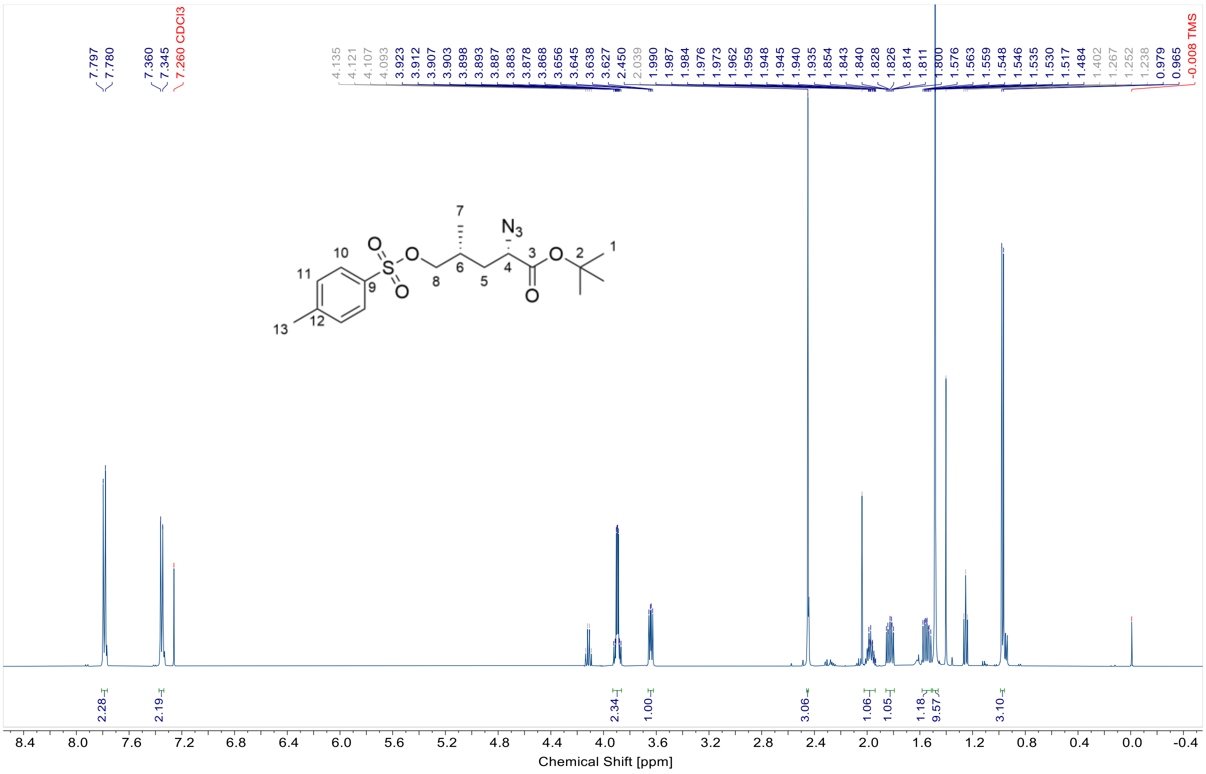

^{13}C-NMR (126 MHz, $CDCl_3$):

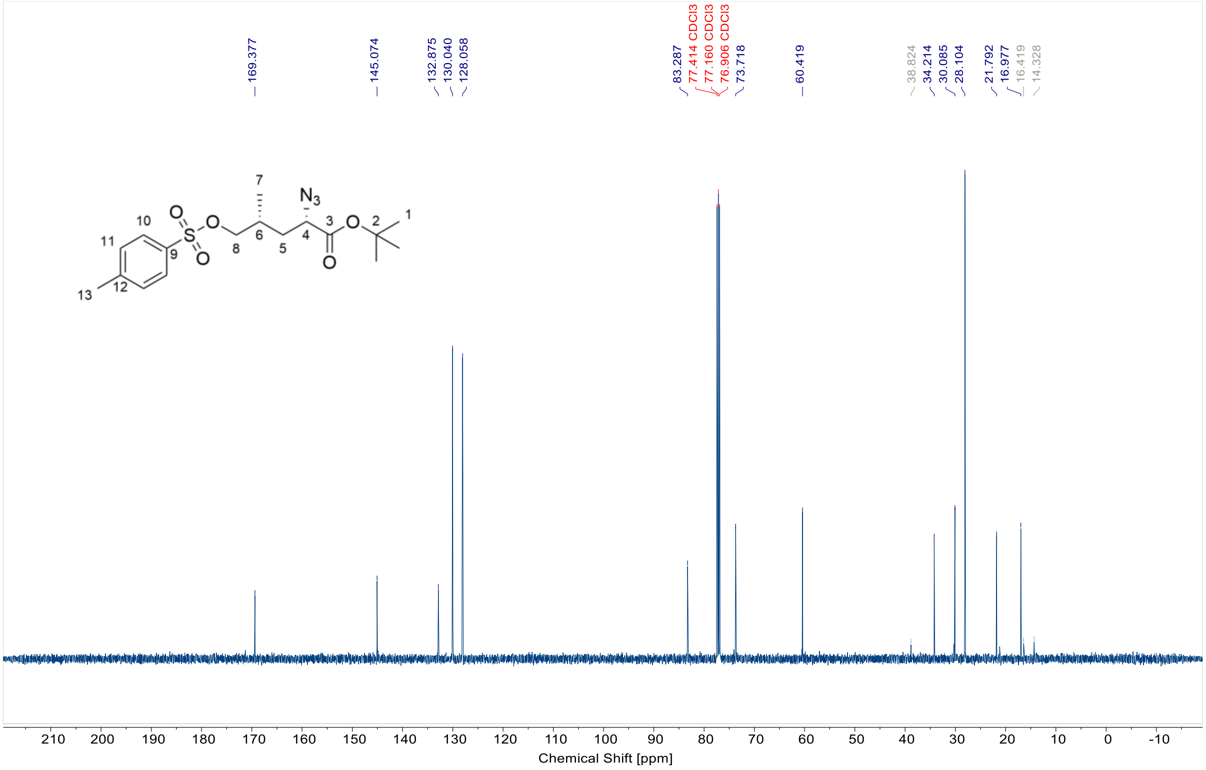

(^{1}H,^{1}H)-COSY ($CDCl_3$):

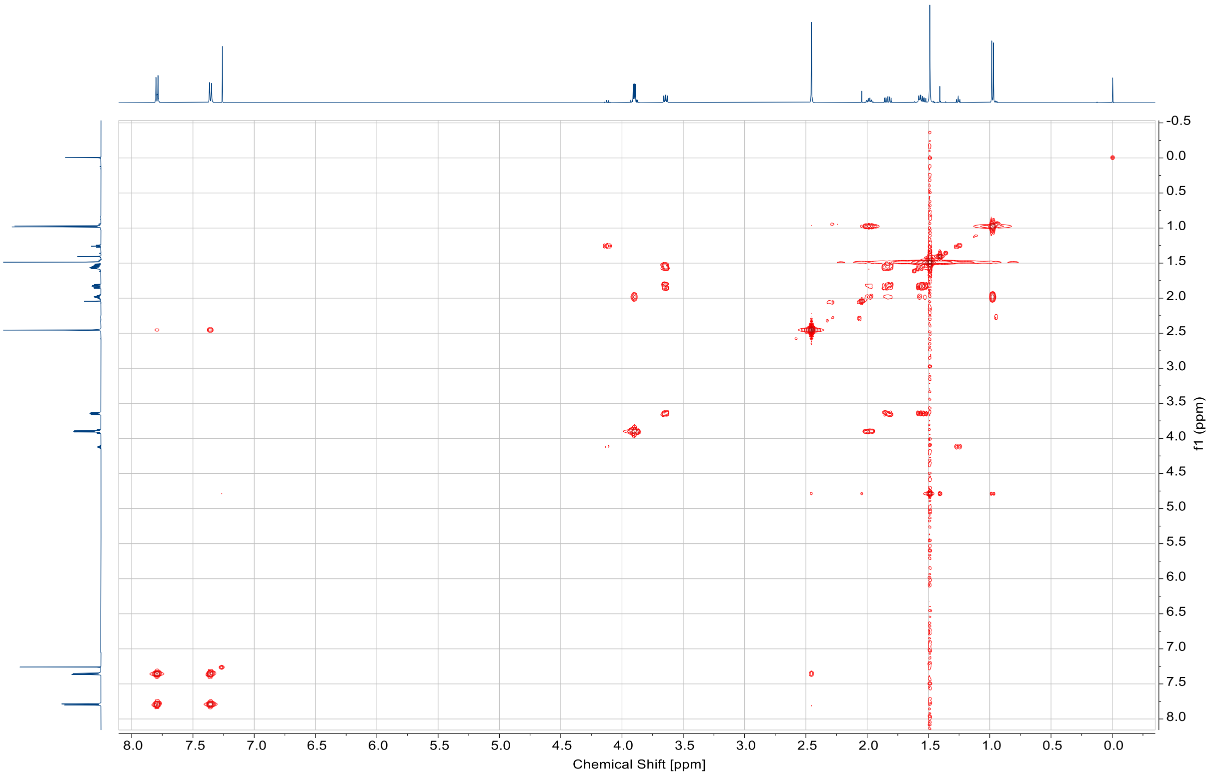

(^{1}H,^{13}C)-HSQC ($CDCl_3$):

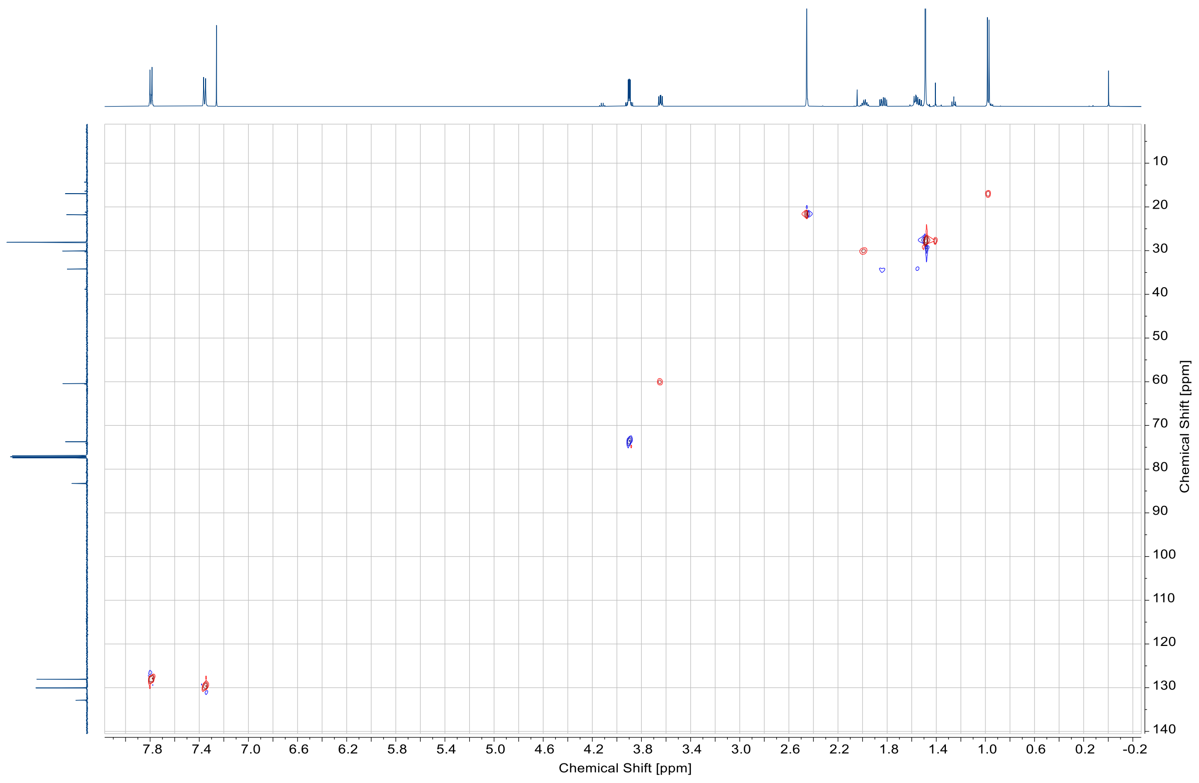

(^{1}H,^{13}C)-HMBC ($CDCl_3$):

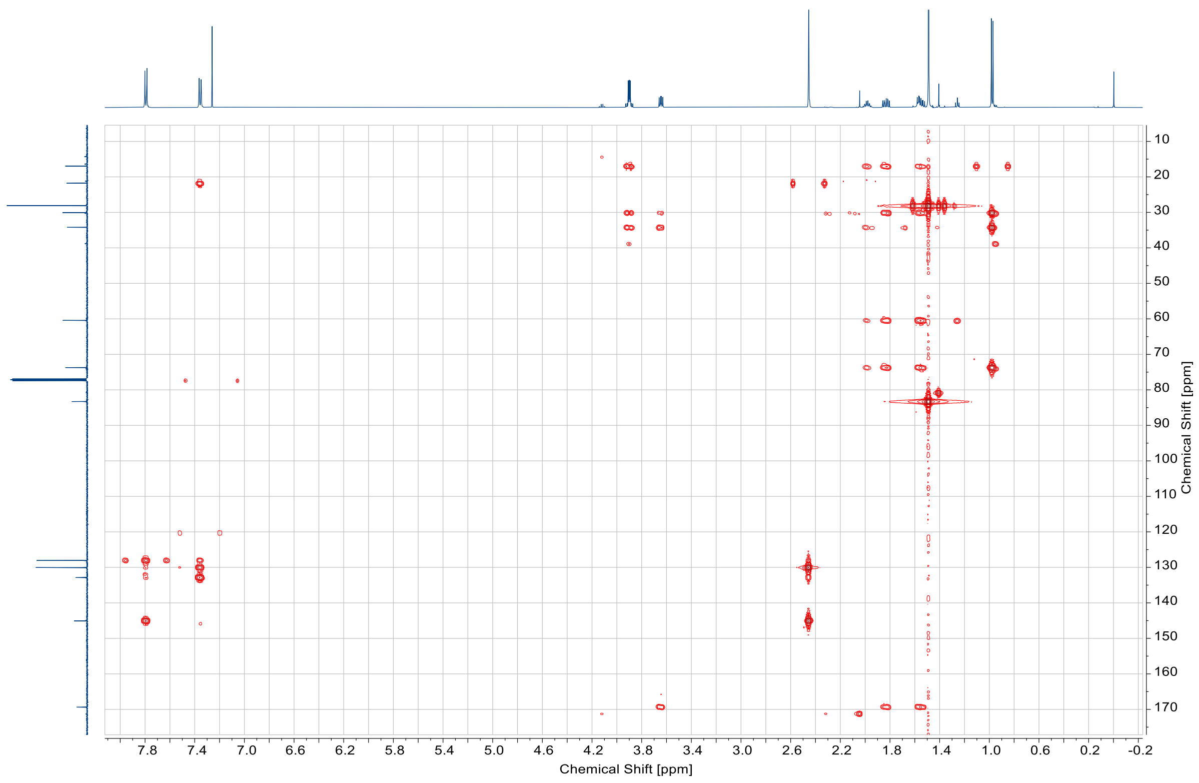

tert-Butyl (2*S*,4*R*)-2-Azido-4-((tosyloxy)methyl)hexanoate [11]

¹H-NMR (400 MHz, $CDCl_3$):

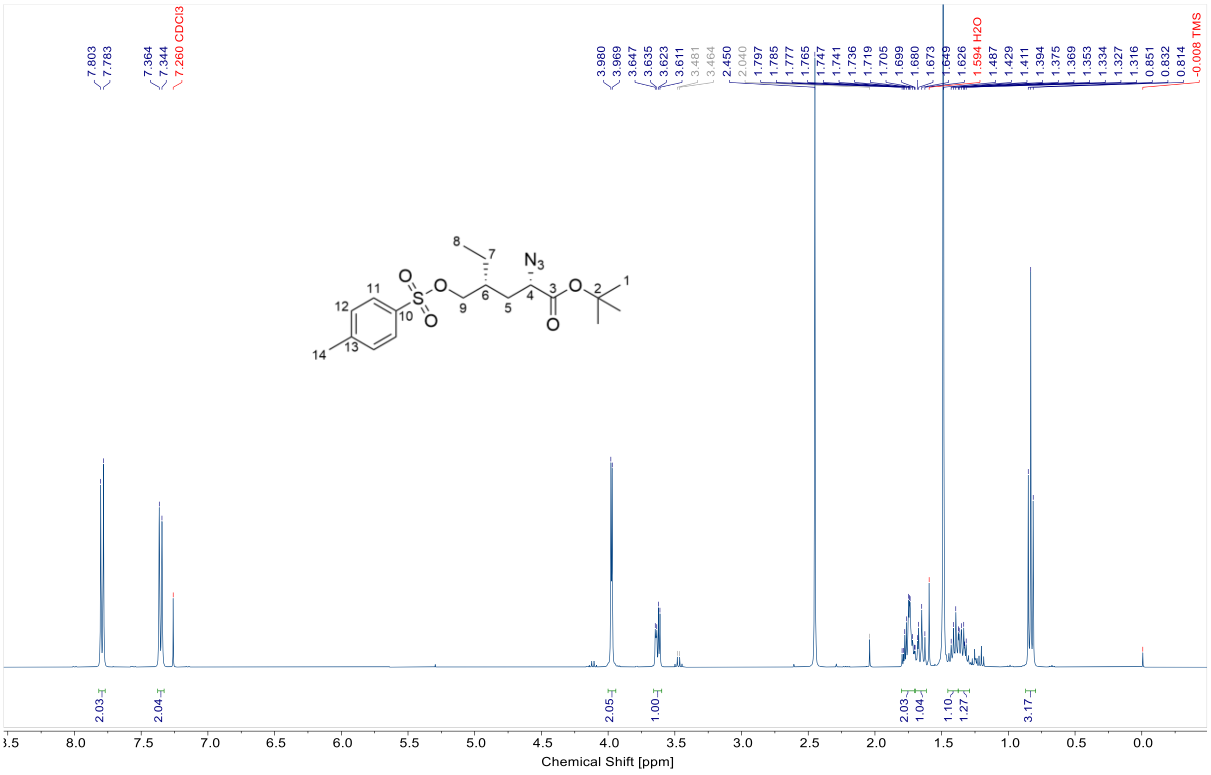

¹³C-NMR (101 MHz, $CDCl_3$):

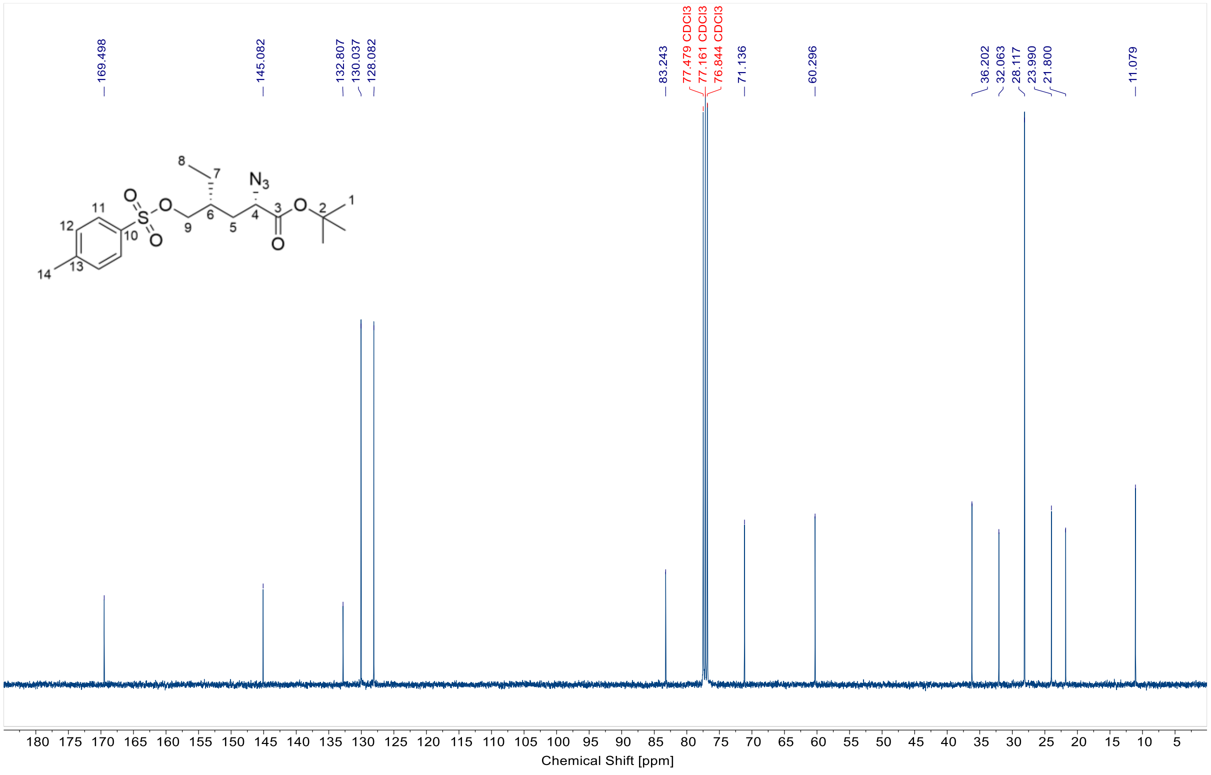

(^{1}H,^{1}H)-COSY ($CDCl_3$):

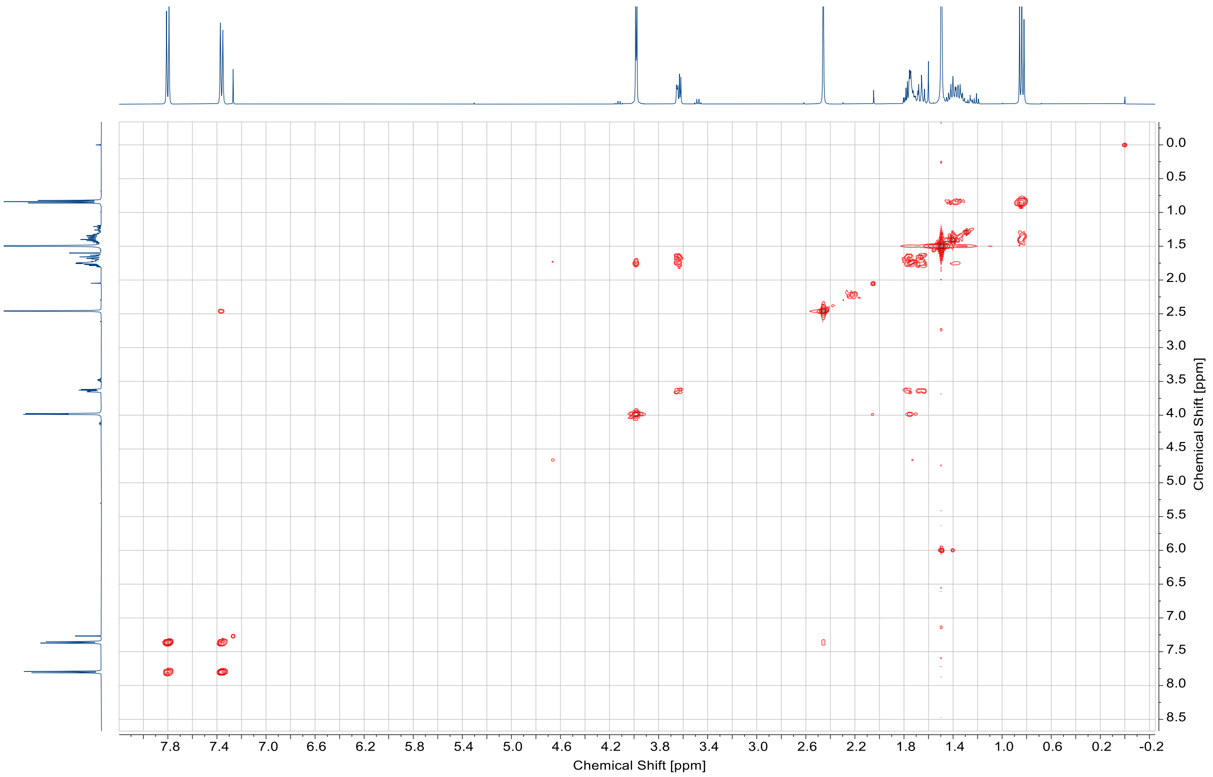

(^{1}H,^{13}C)-HSQC ($CDCl_3$):

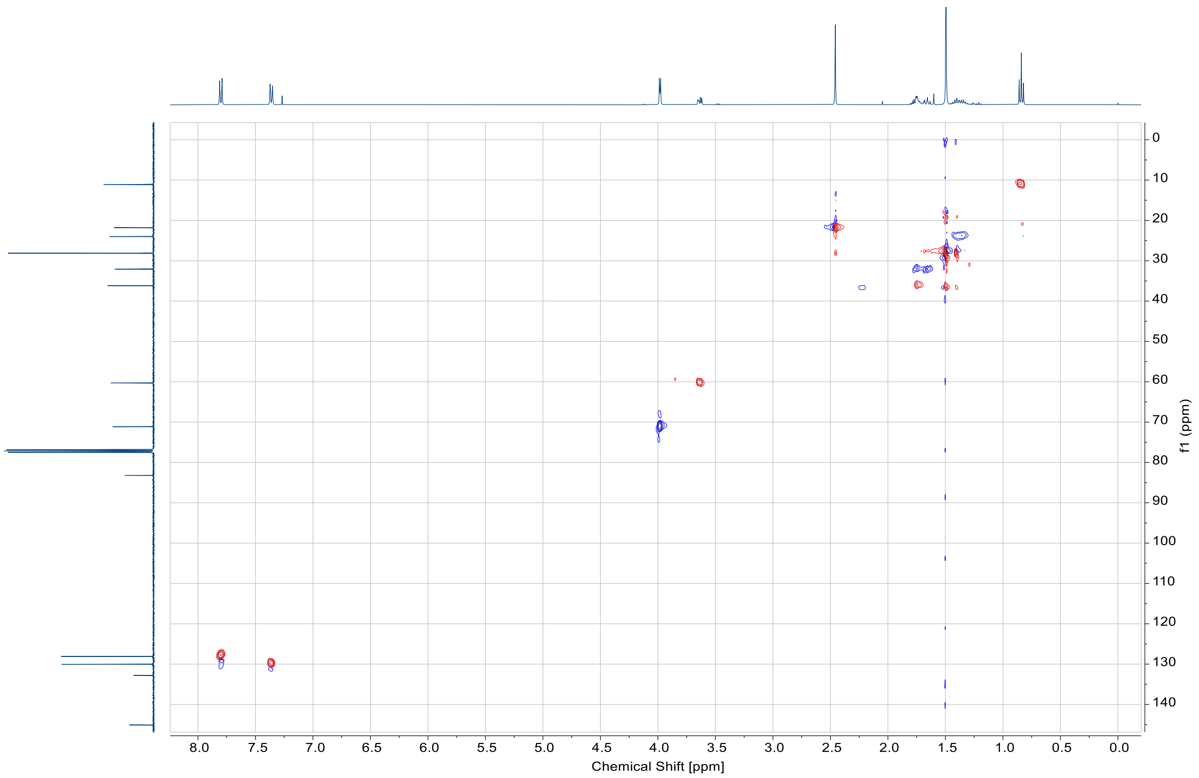

(^{1}H,^{13}C)-HMBC ($CDCl_3$):

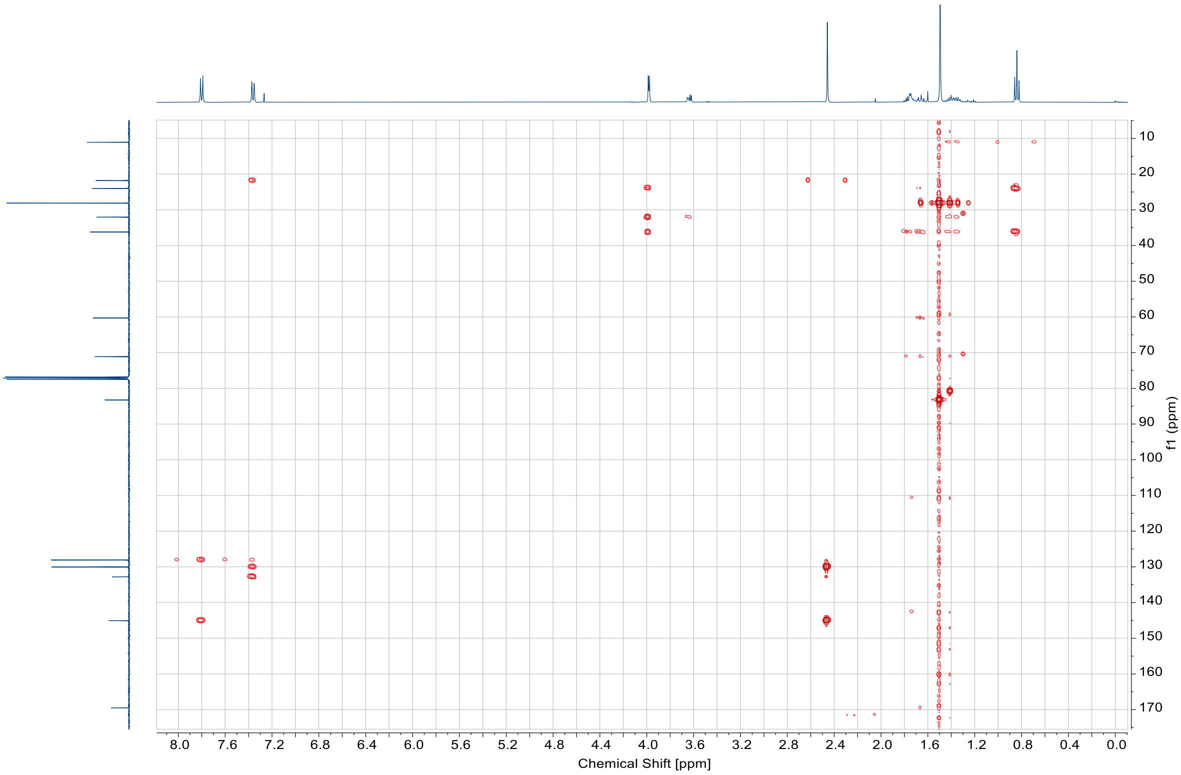

tert-Butyl (2*S*,4*R*)-*N*-Benzyloxycarbonyl-4-Methyl-prolinate [12]

^{1}H-NMR (500 MHz, DMSO-d_6, 373 K):

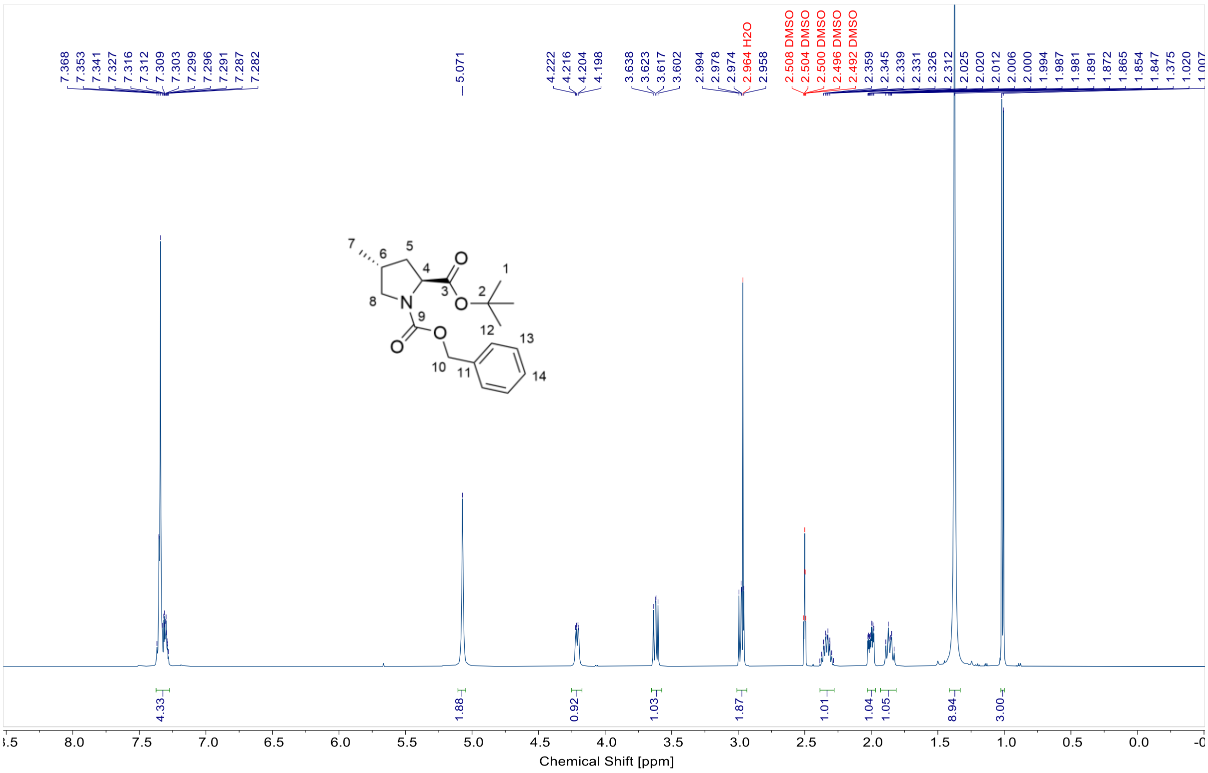

^{13}C-NMR (126 MHz, DMSO-d_6, 373 K):

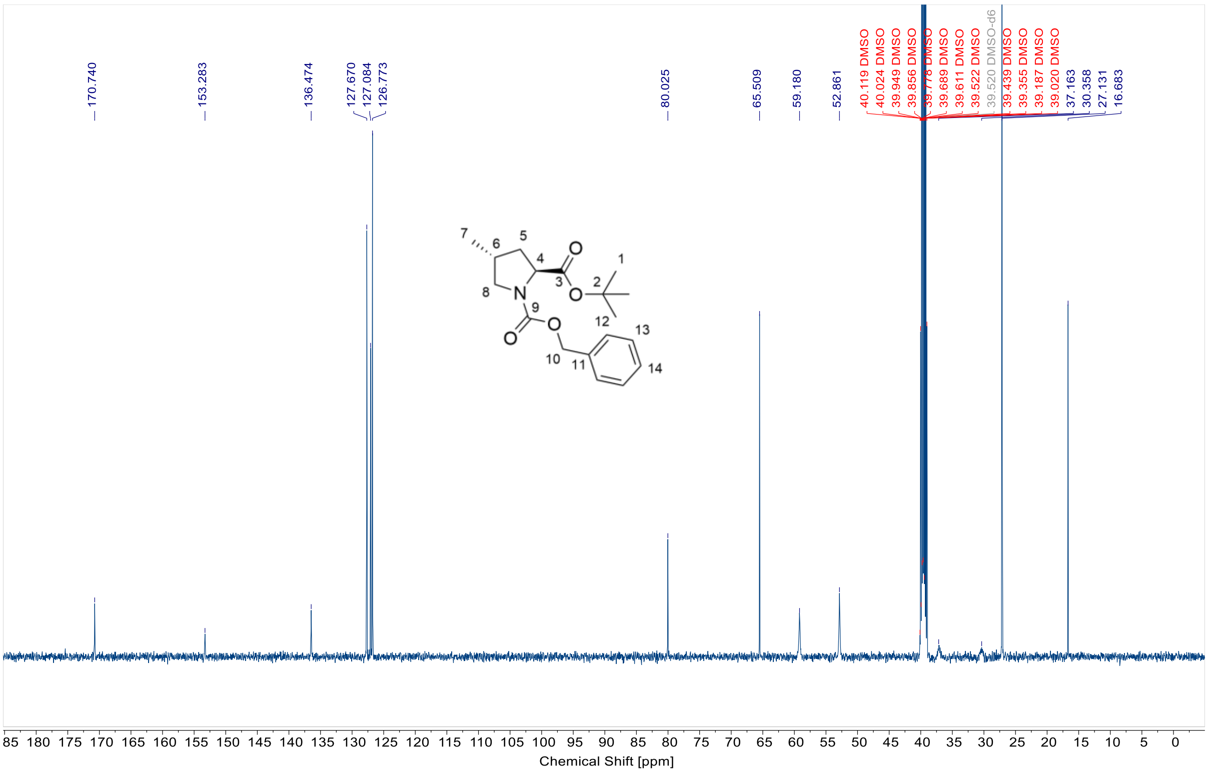

(^{1}H,^{1}H)-COSY (DMSO-d_6, 373 K):

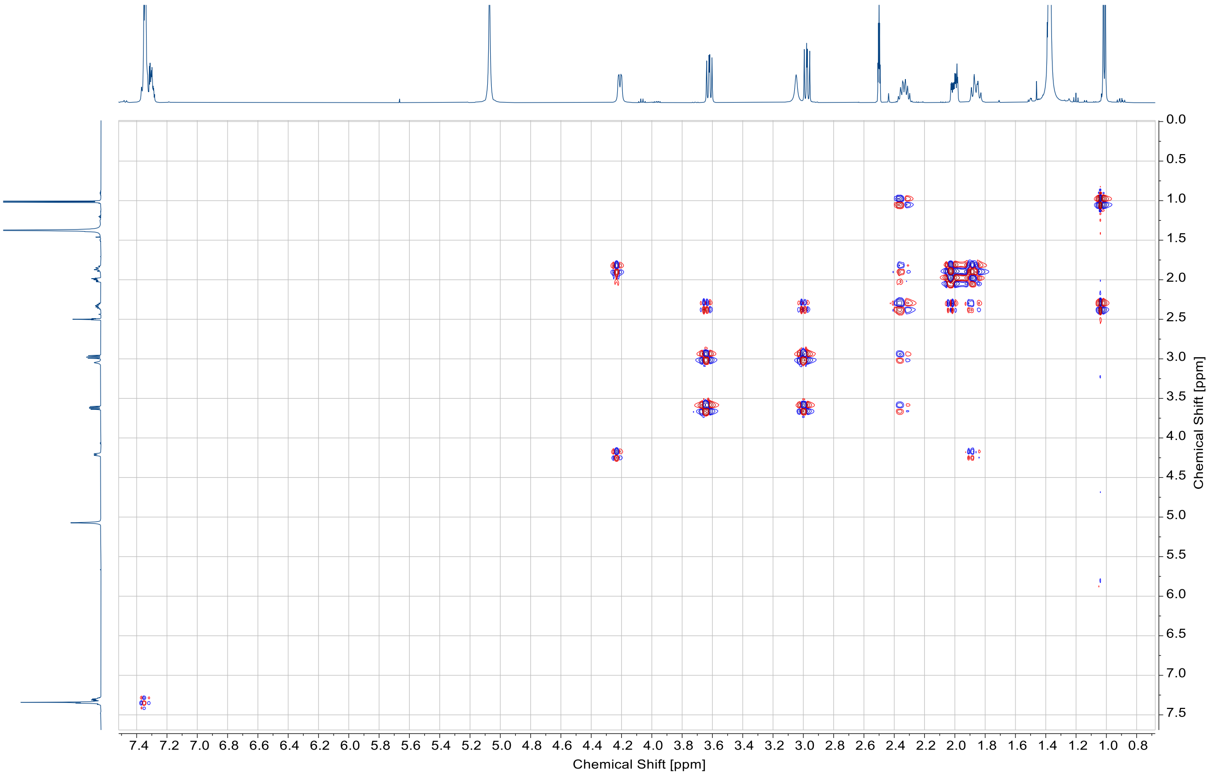

(^{1}H,^{13}C)-HSQC (DMSO-d_6, 373 K):

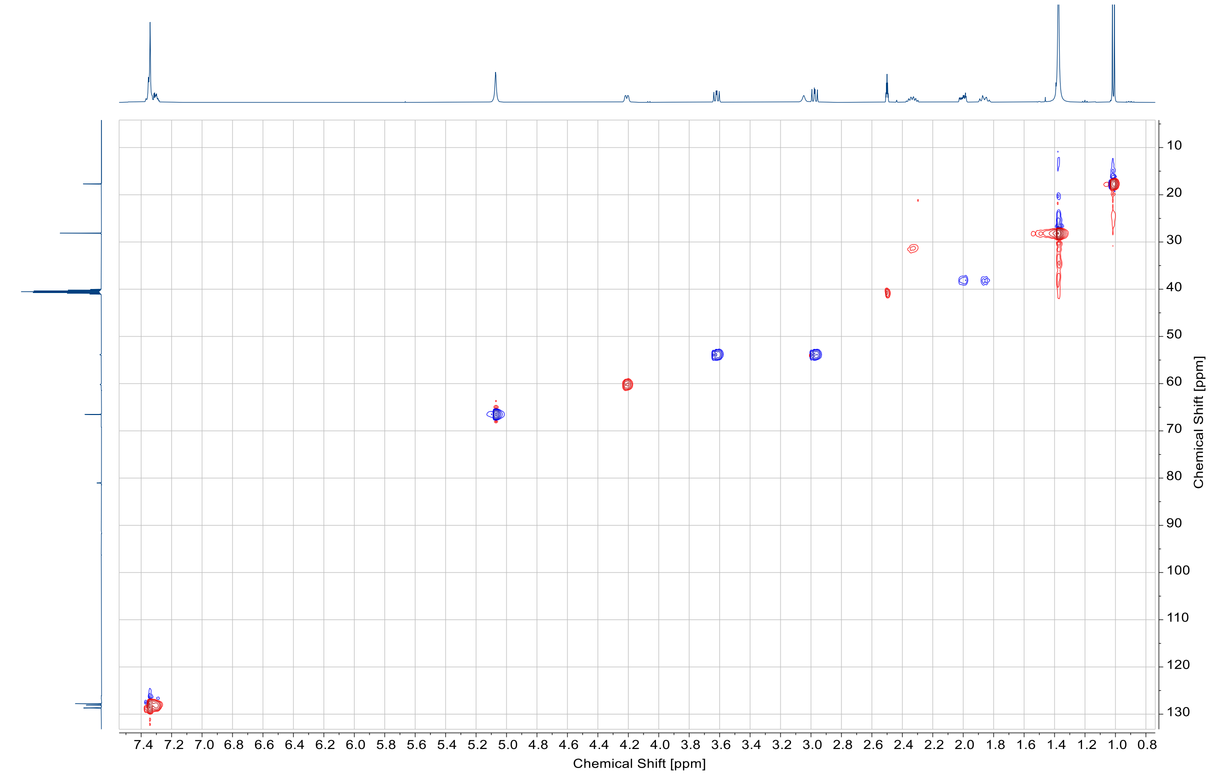

(^{1}H,^{13}C)-HMBC (DMSO-d_6, 373 K):

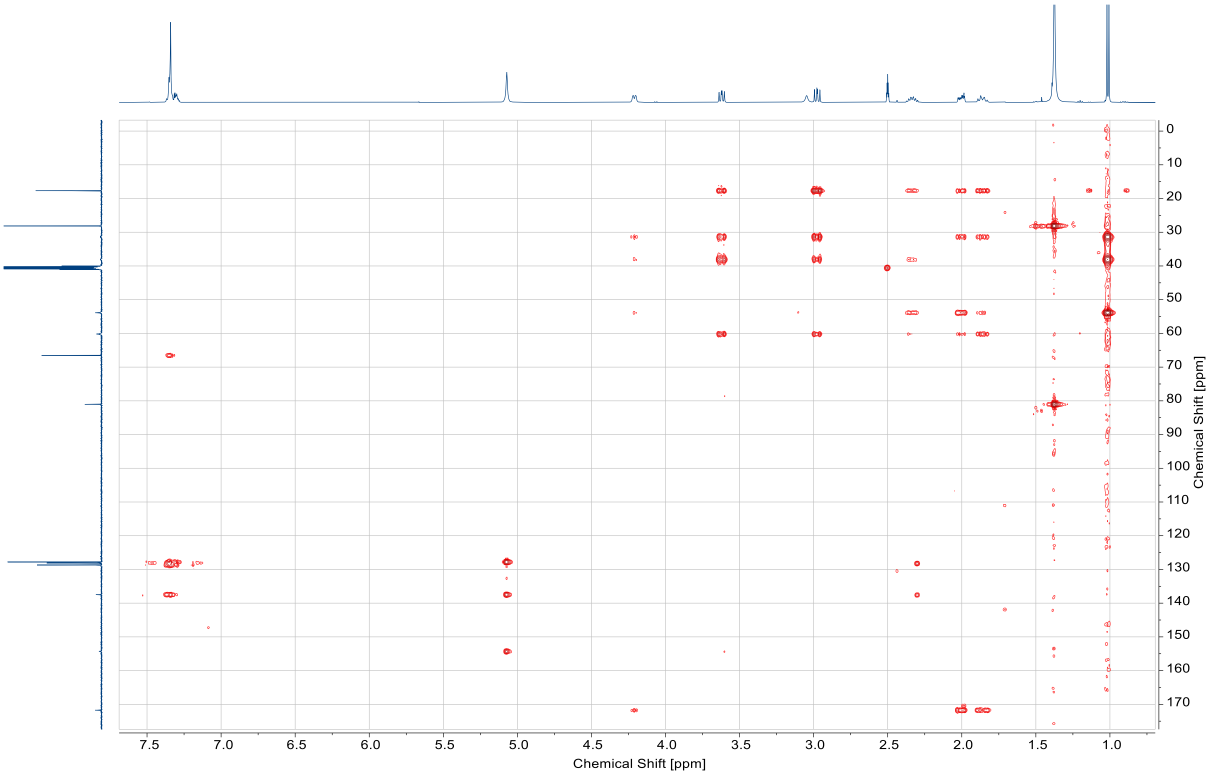

tert-Butyl (2_S_,4_R_)-_N_-benzyloxycarbonyl-4-ethyl-prolinate [13]

^{1}H-NMR (500 MHz, DMSO-d_6, 373 K):

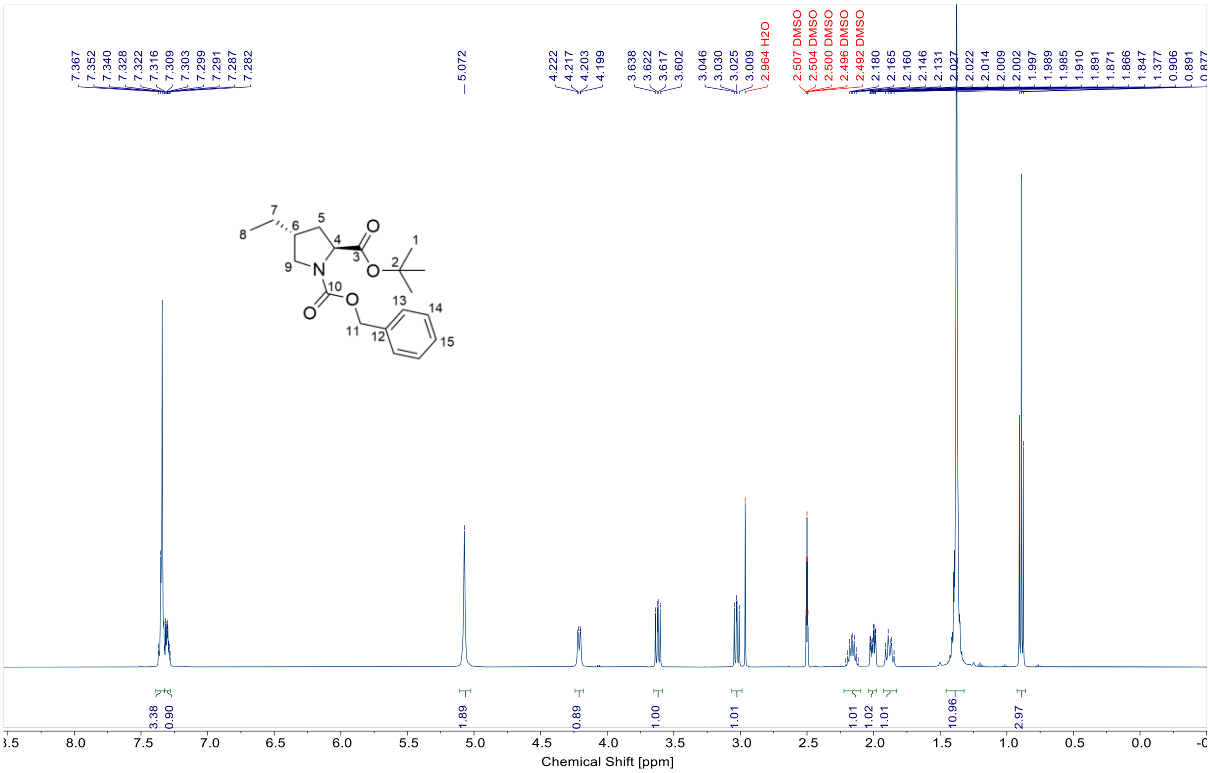

^{13}C-NMR (126 MHz, DMSO-d_6, 373 K):

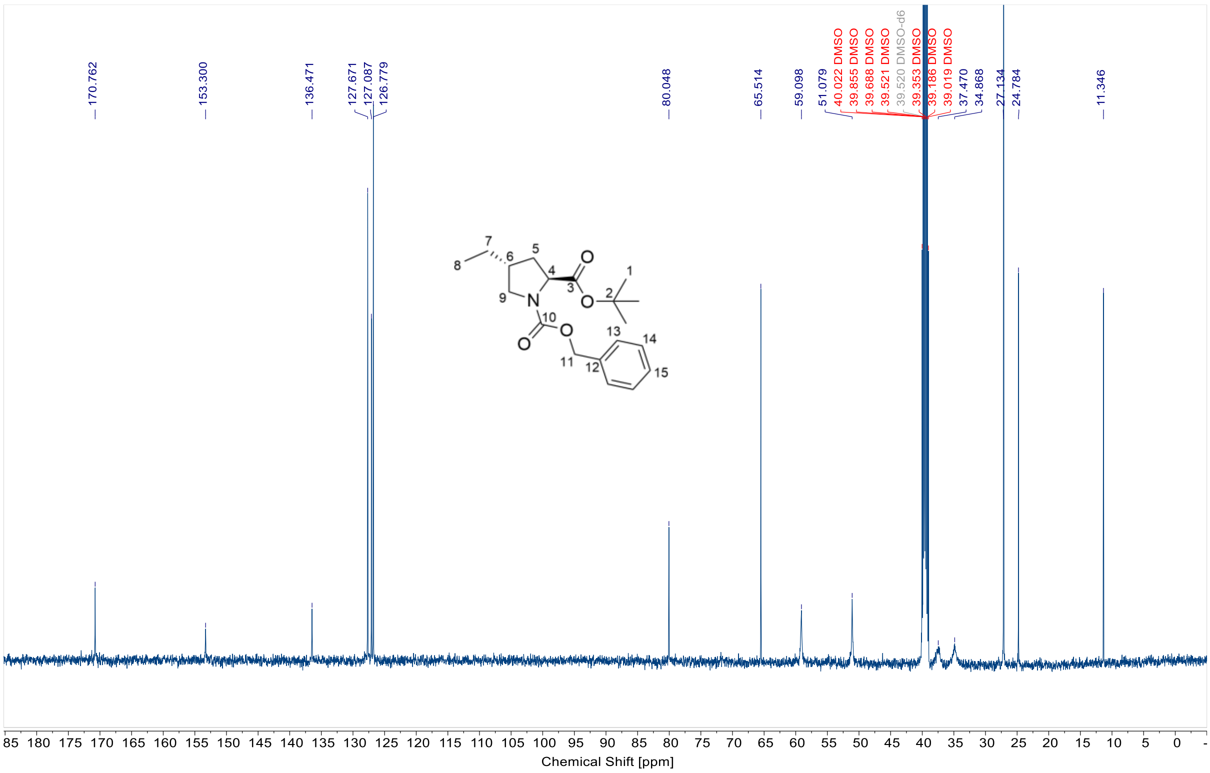

(^{1}H,^{1}H)-COSY (DMSO-d_6, 373 K):

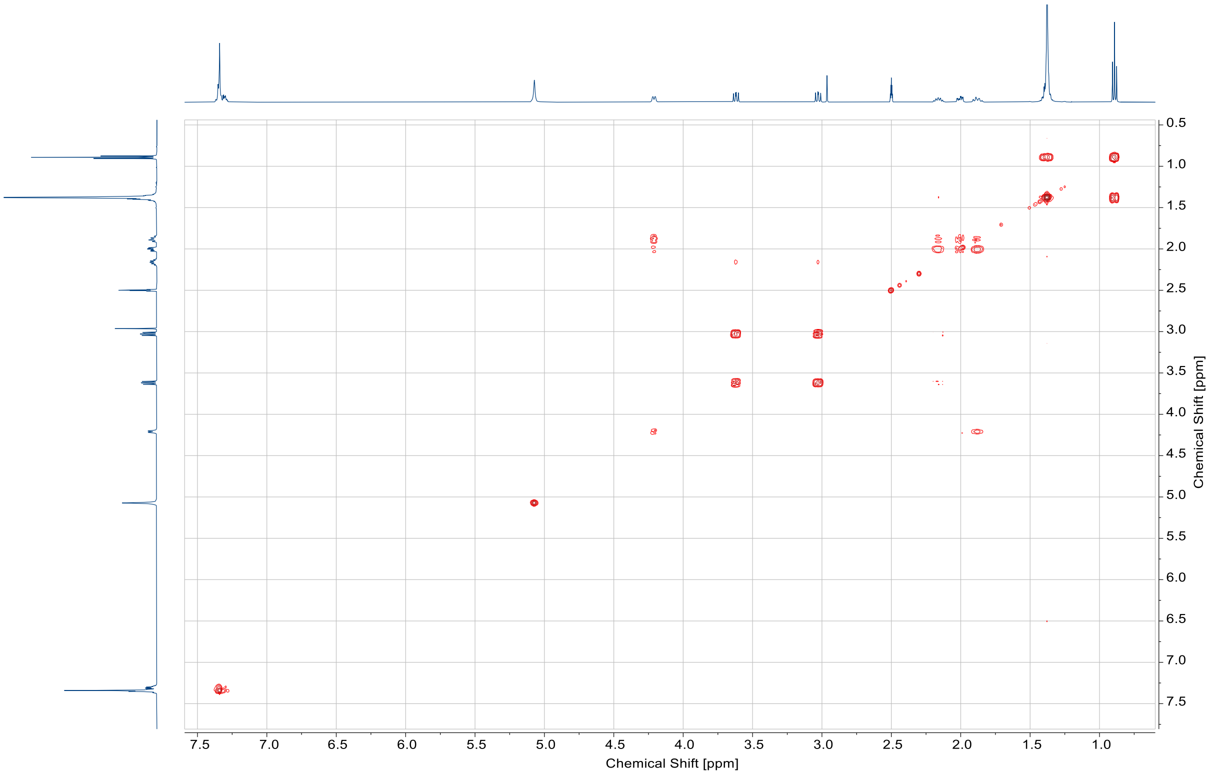

(^{1}H,^{13}C)-HSQC (DMSO-d_6, 373 K):

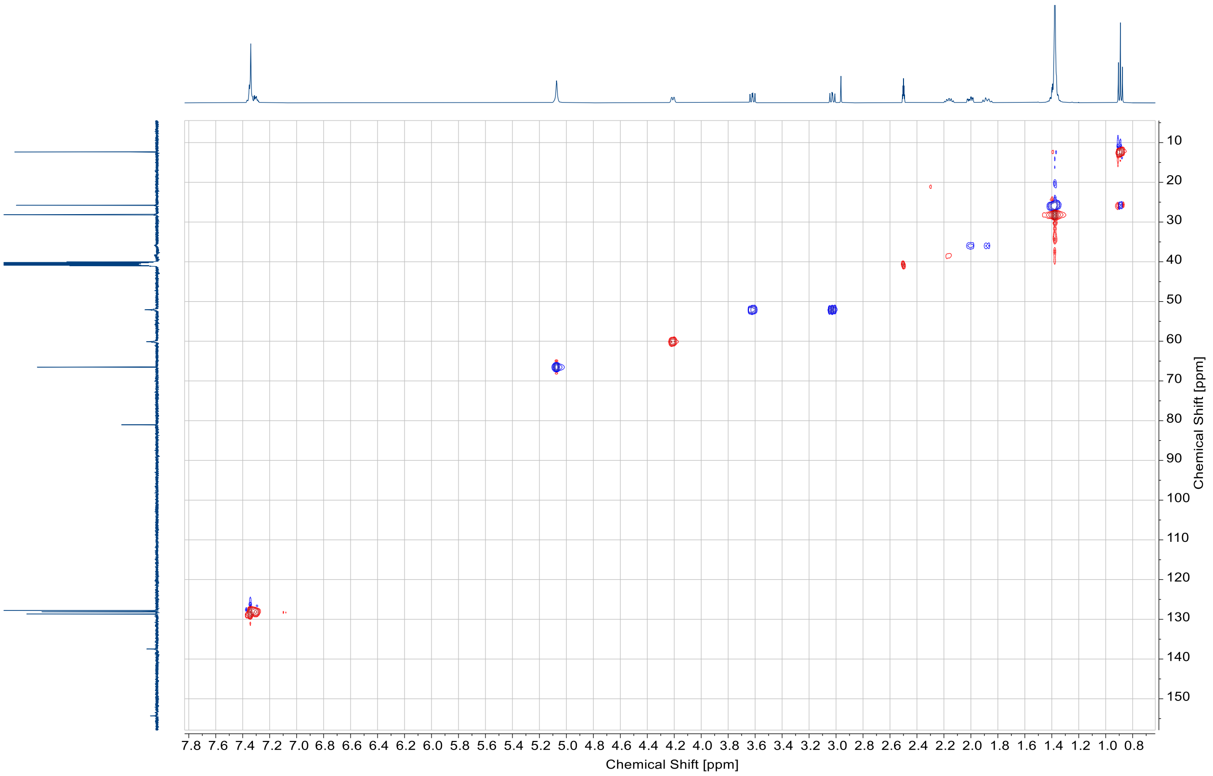

(^{1}H,^{13}C)-HMBC (DMSO-d_6, 373 K):

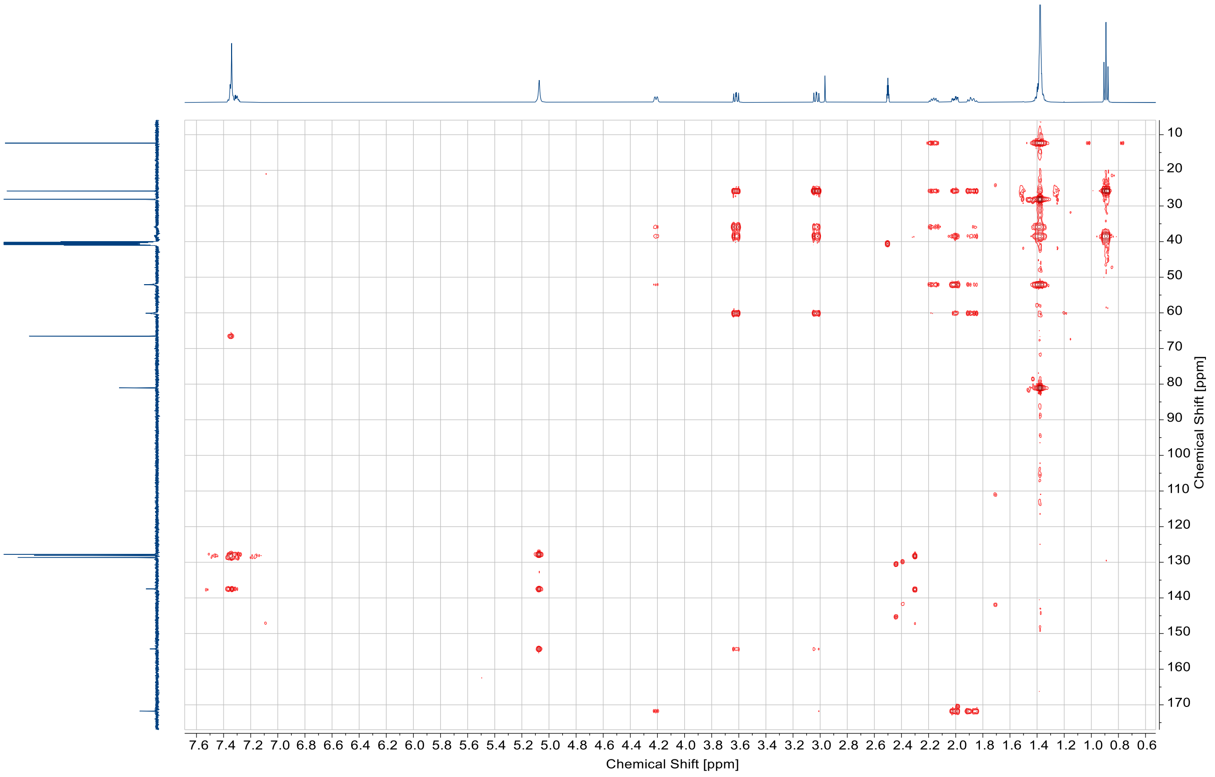

(2*S*,4*R*)-*N*-Benzyloxycarbonyl-4-ethyl-proline [13']

^{1}H-NMR (500 MHz, $CDCl_3$):

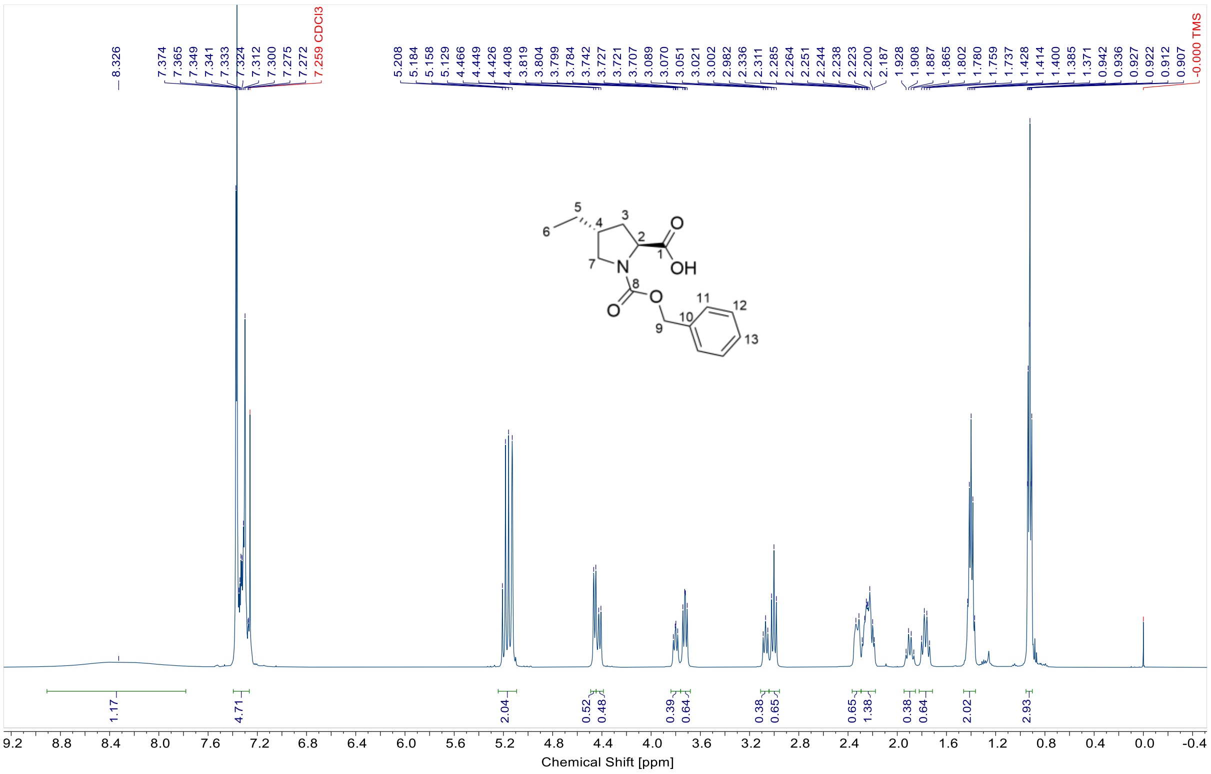

^{13}C-NMR (126 MHz, $CDCl_3$):

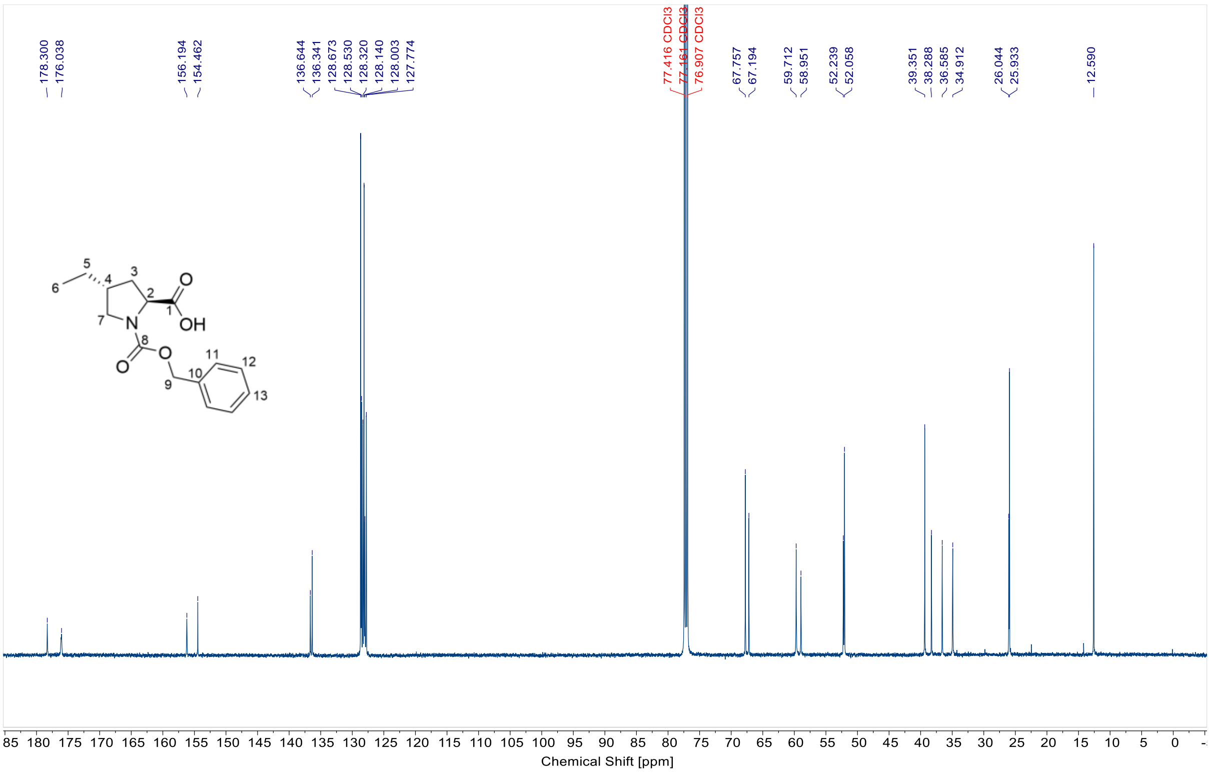

(^{1}H,^{1}H)-COSY ($CDCl_3$):

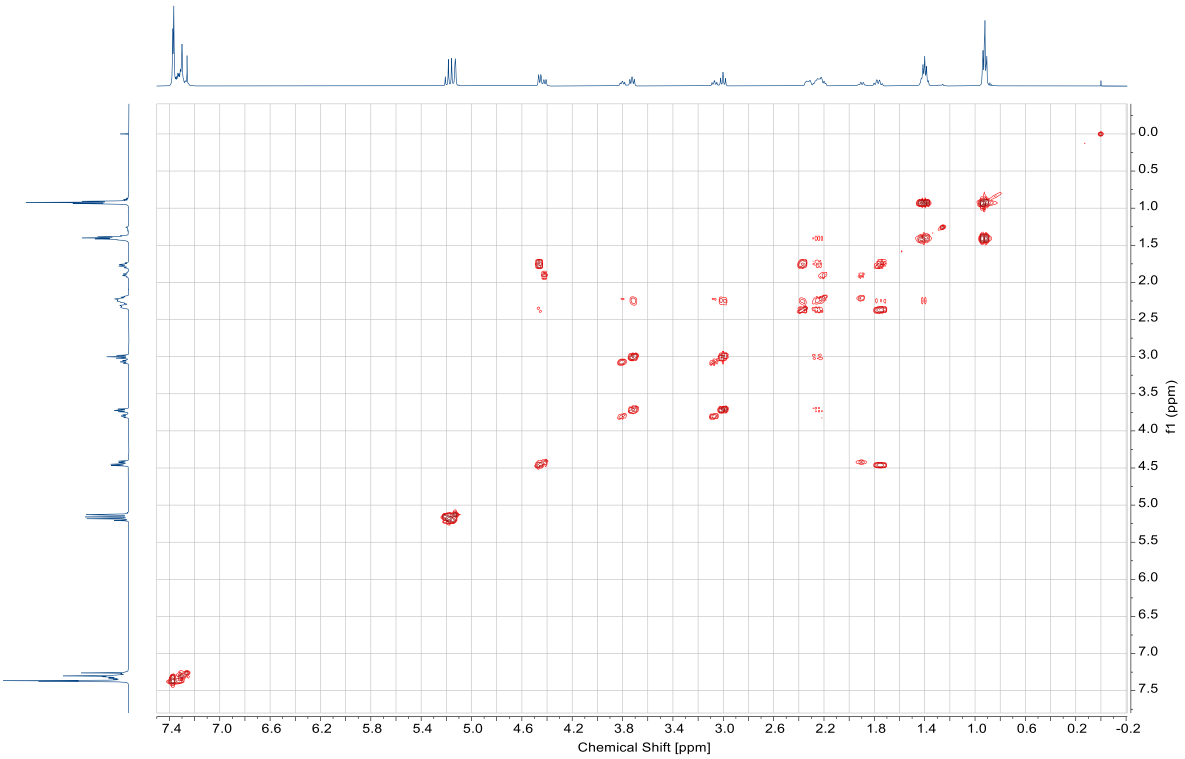

(^{1}H,^{13}C)-HSQC ($CDCl_3$):

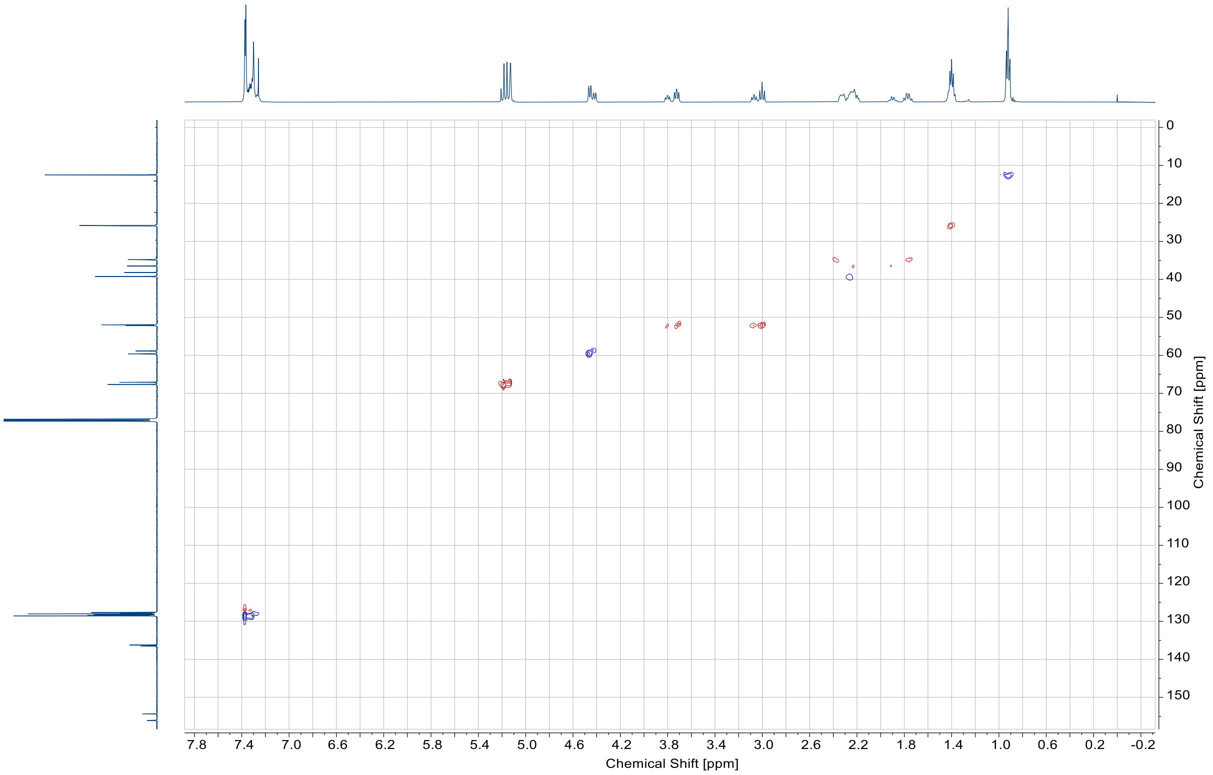

(^{1}H,^{13}C)-HMBC ($CDCl_3$):

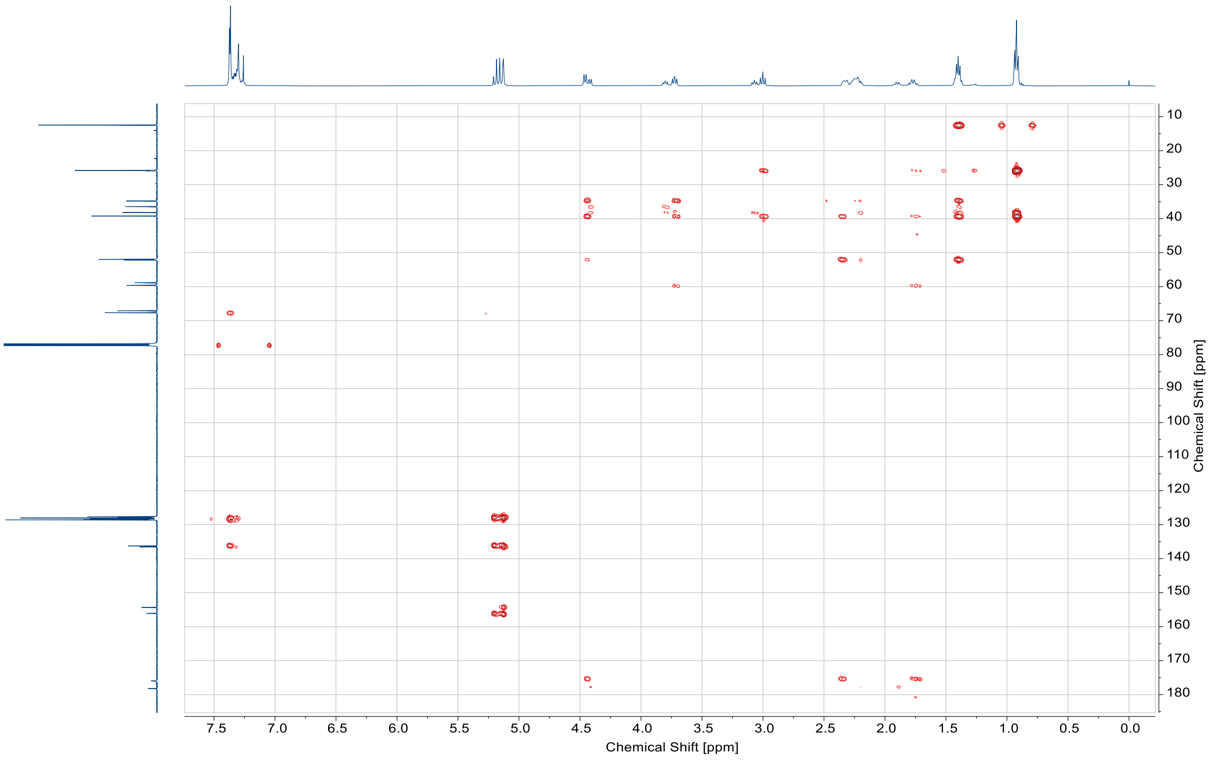

N-Benzyloxycarbonyl-*O*-*tert*-butyldimethylsilyl-L-threonine [S1]

^{1}H-NMR (500 MHz, $CDCl_3$):

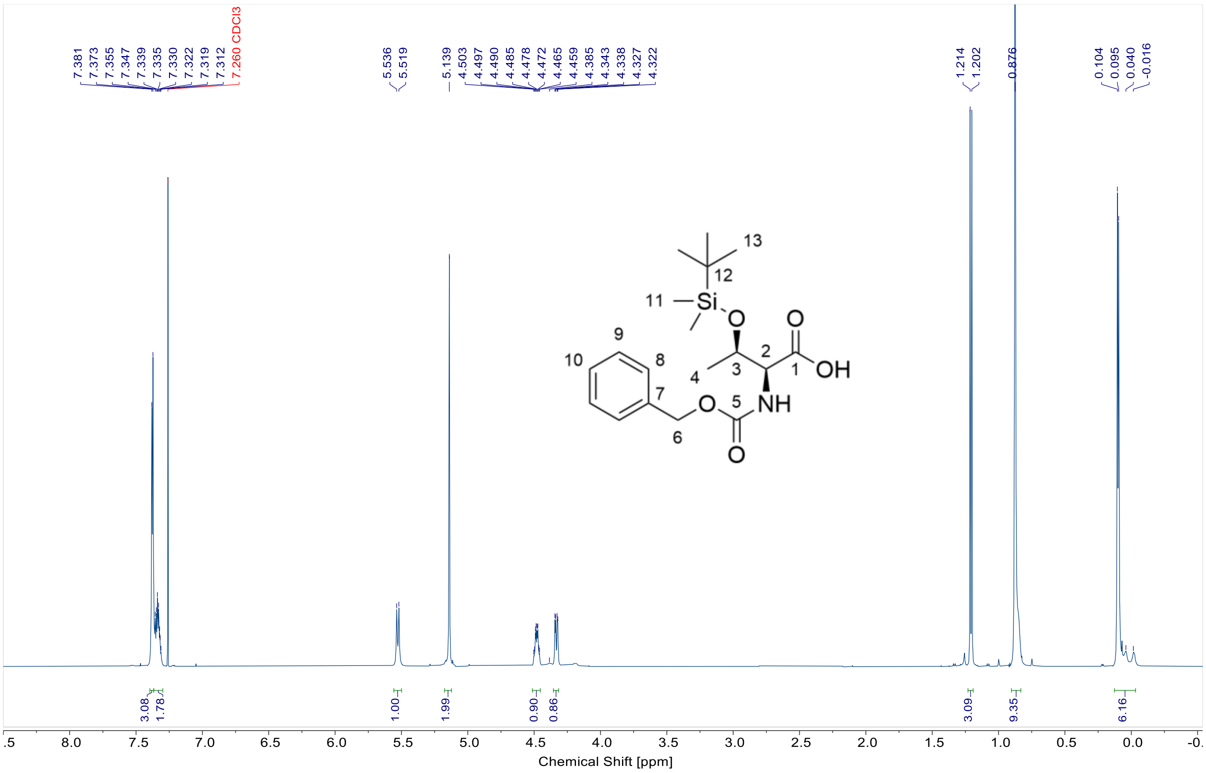

^{13}C-NMR (126 MHz, $CDCl_3$):

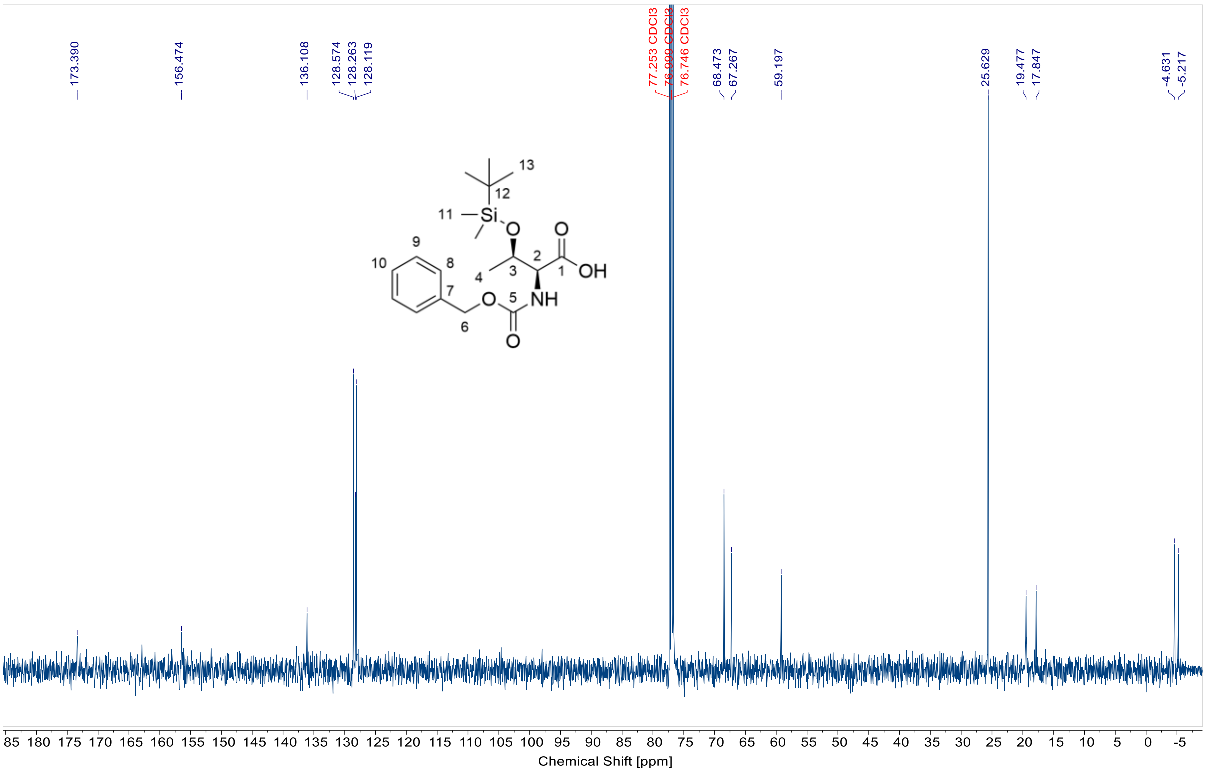

(^{1}H,^{1}H)-COSY (CDCl$_3$):

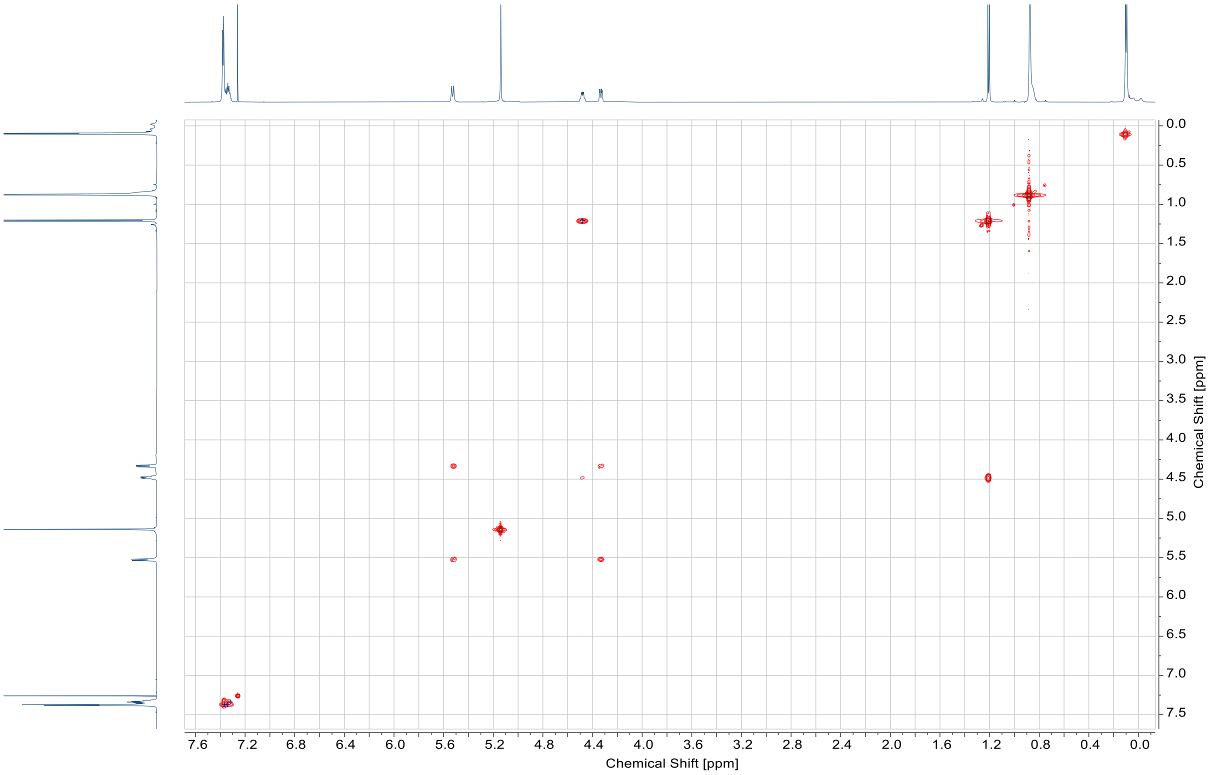

(^{1}H,^{13}C)-HSQC (CDCl$_3$):

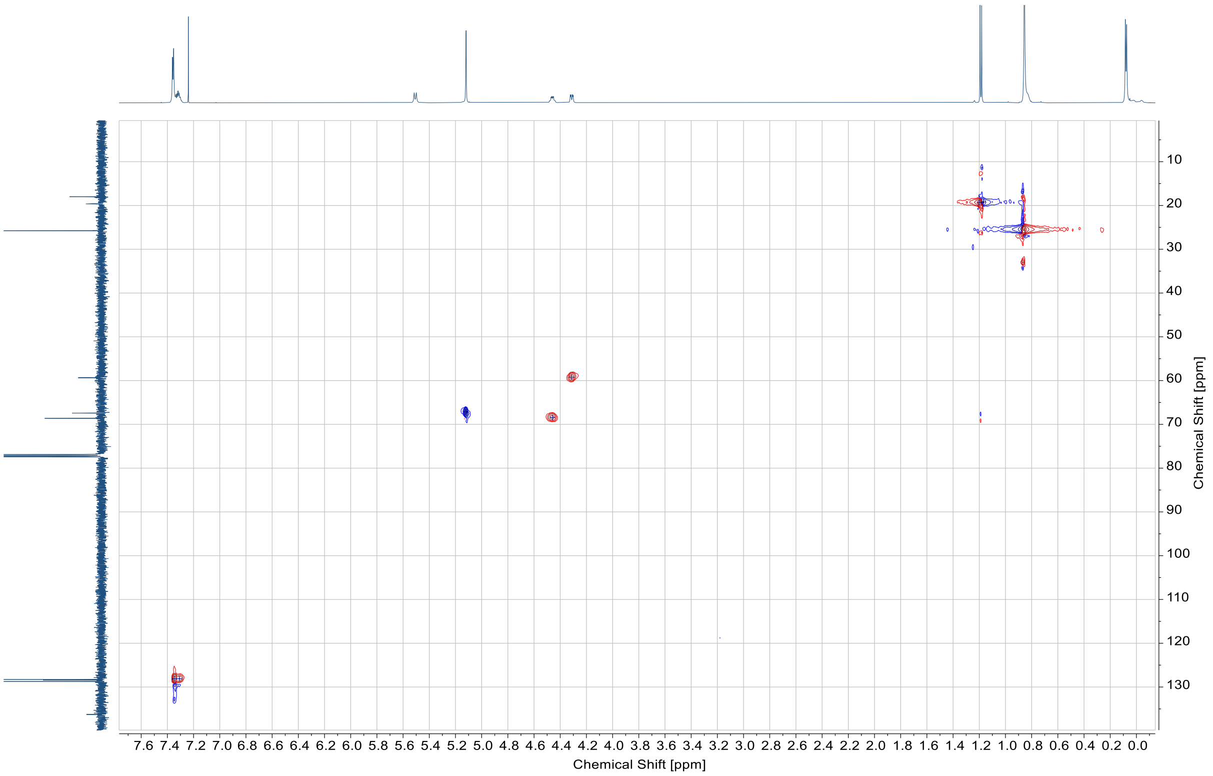

(^{1}H,^{13}C)-HMBC ($CDCl_3$):

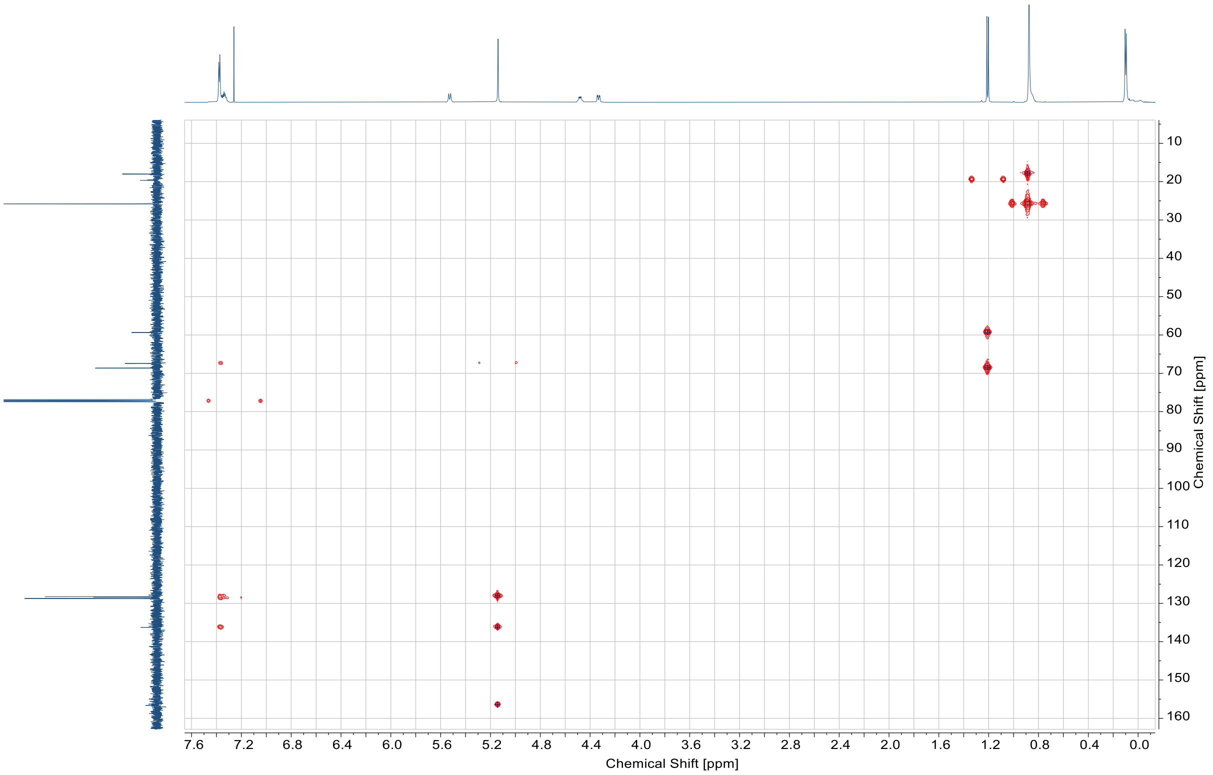

N-Benzyloxycarbonyl-*N*-methyl-*O*-*tert*-butyldimethylsilyl-L-threonine [S2]

^{1}H-NMR (400 MHz, $CDCl_3$):

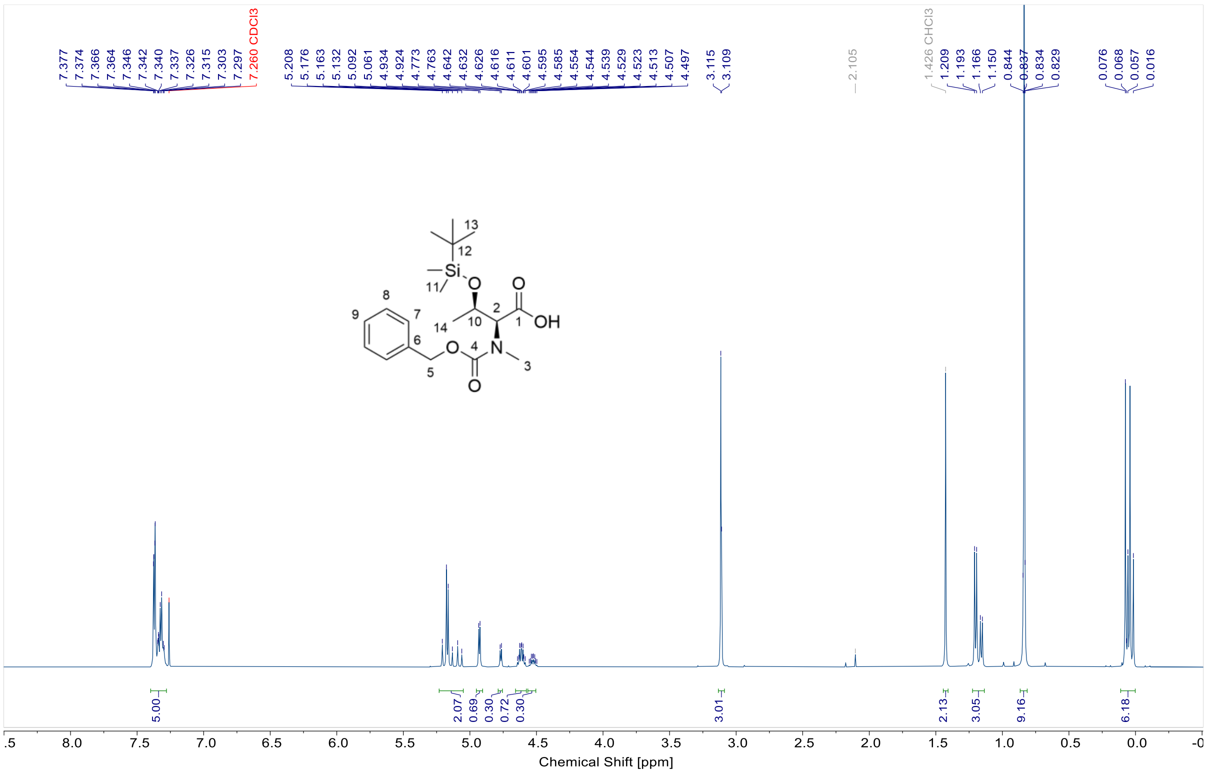

^{13}C-NMR (101 MHz, $CDCl_3$):

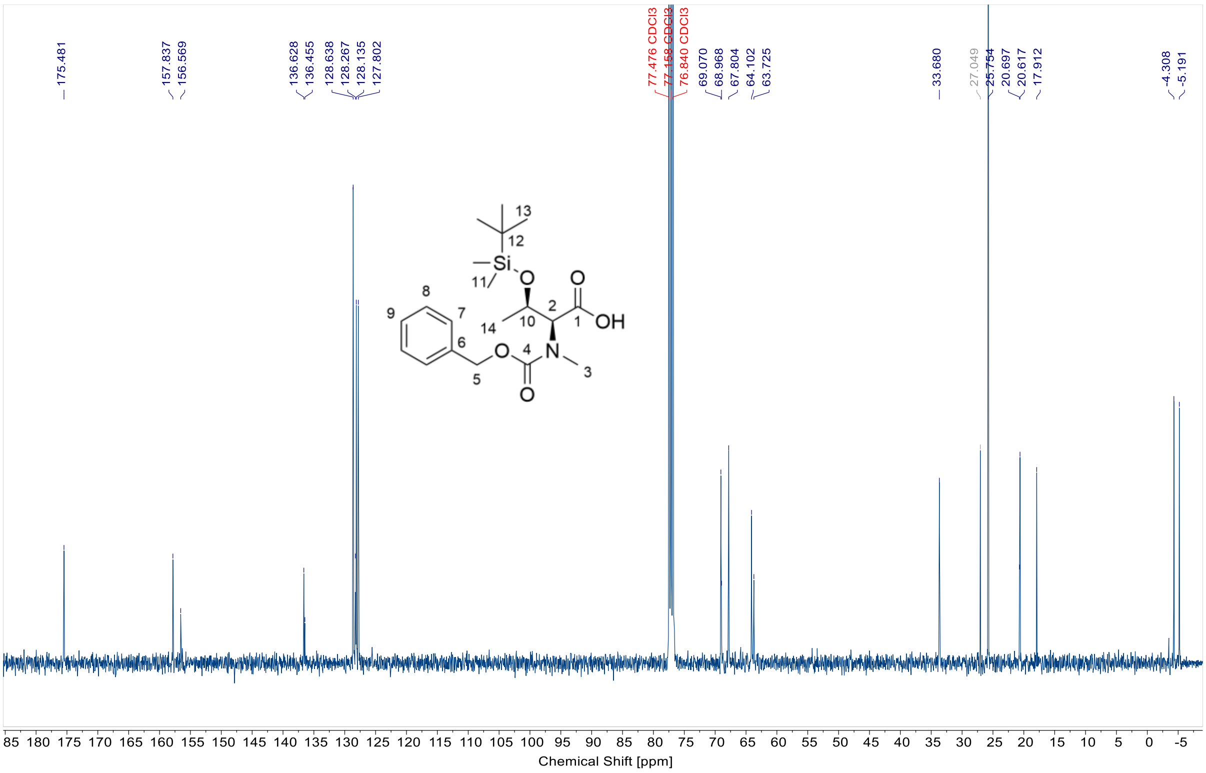

(^{1}H,^{1}H)-COSY ($CDCl_3$):

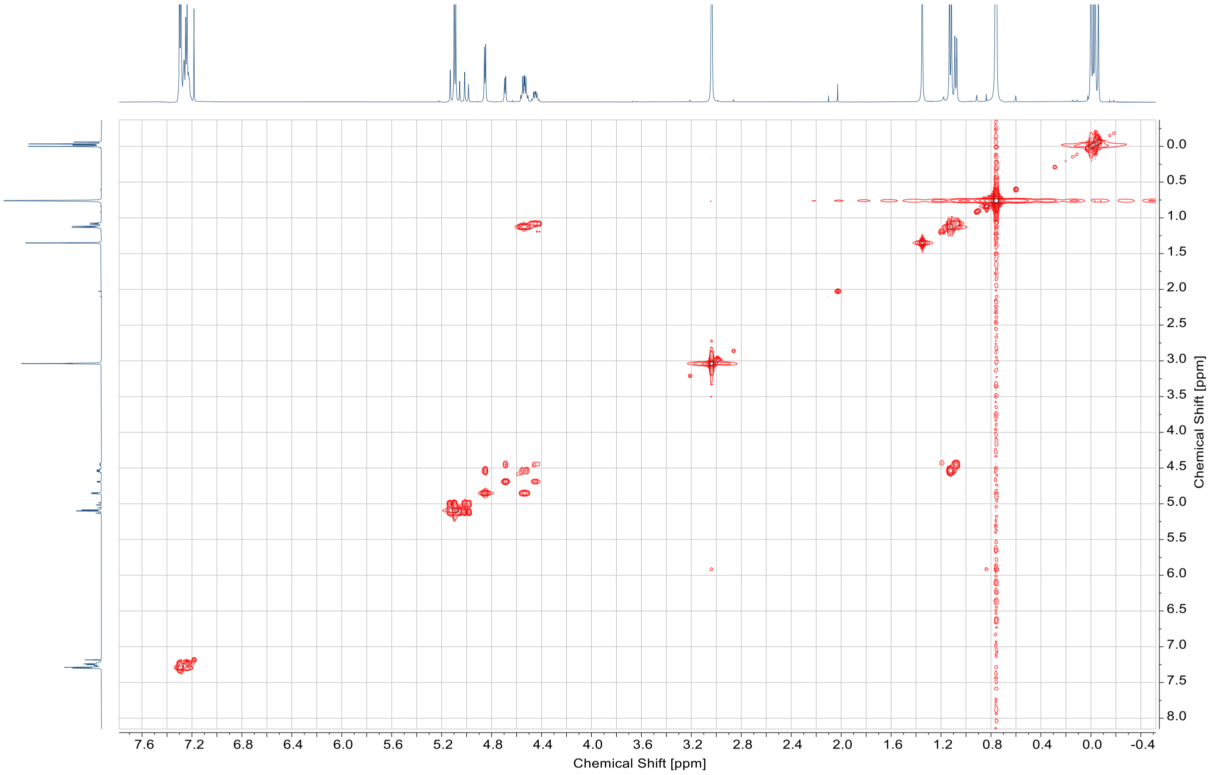

(^{1}H,^{13}C)-HSQC (CDCl$_3$):

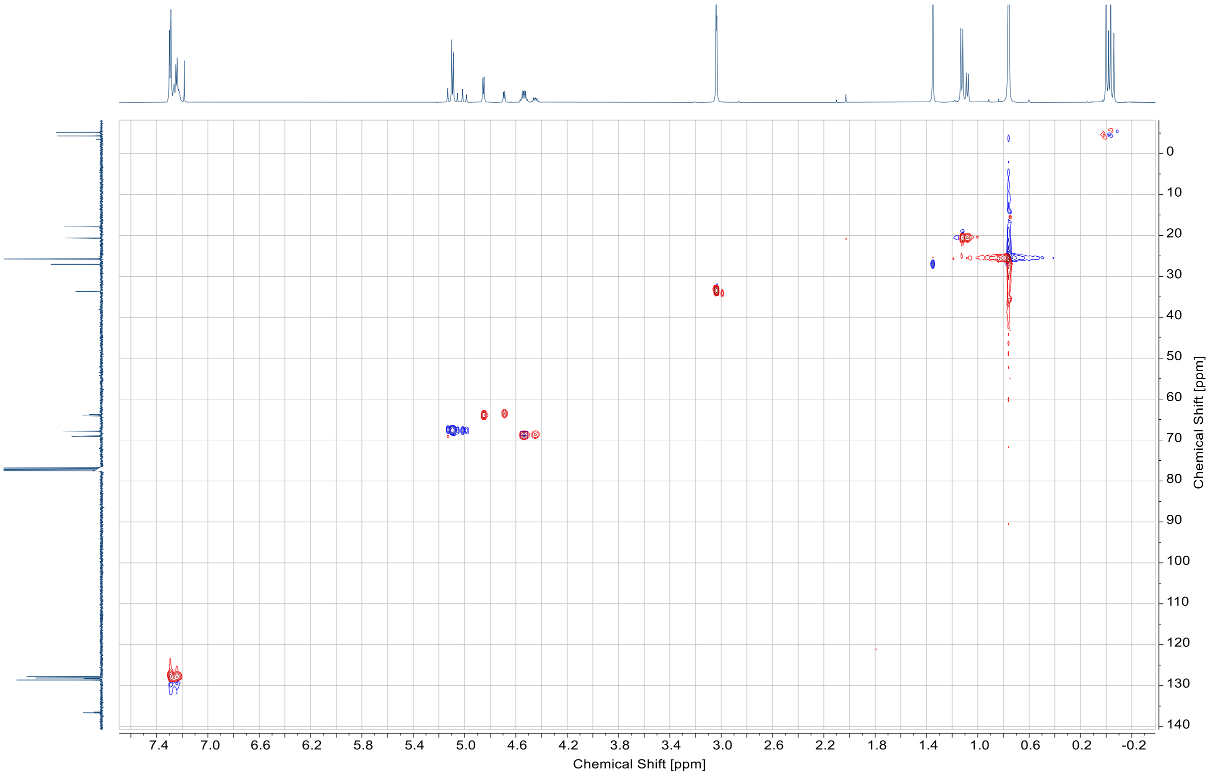

(^{1}H,^{13}C)-HMBC (CDCl$_3$):

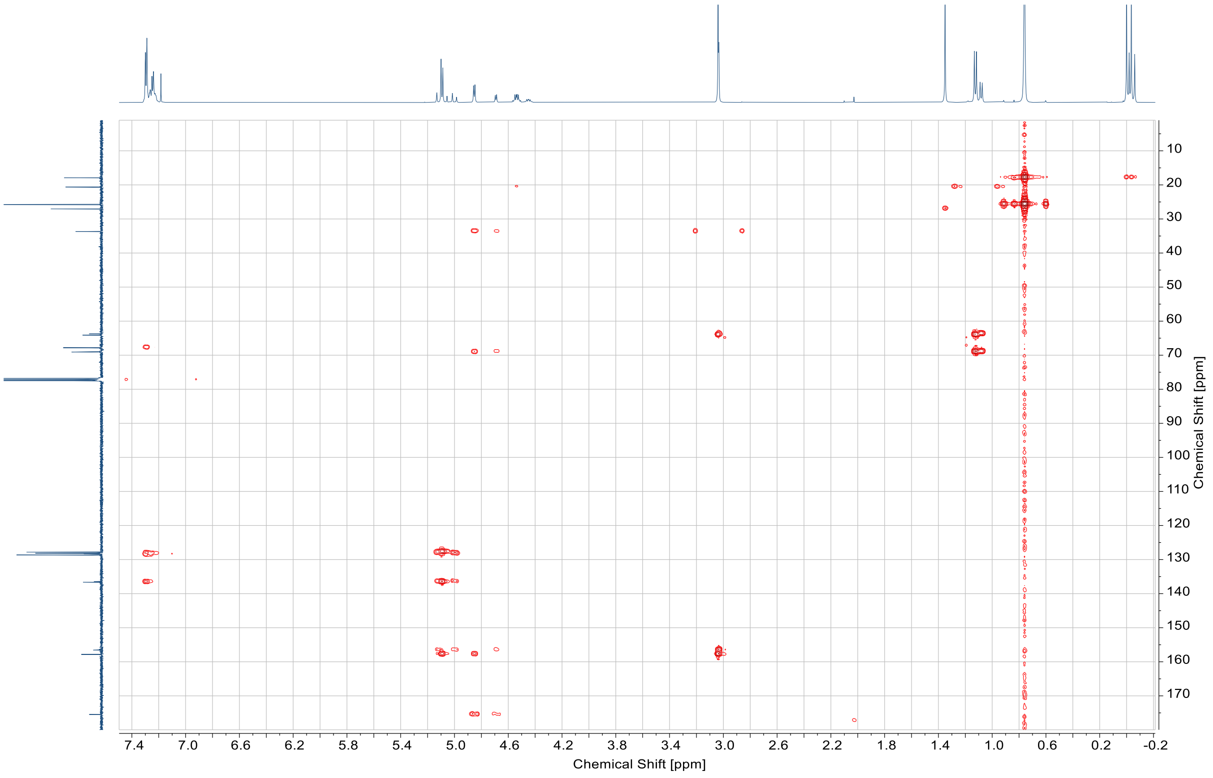

N-Allyloxycarbonyl-L-valine [S3]

^{1}H-NMR (400 MHz, $CDCl_3$):

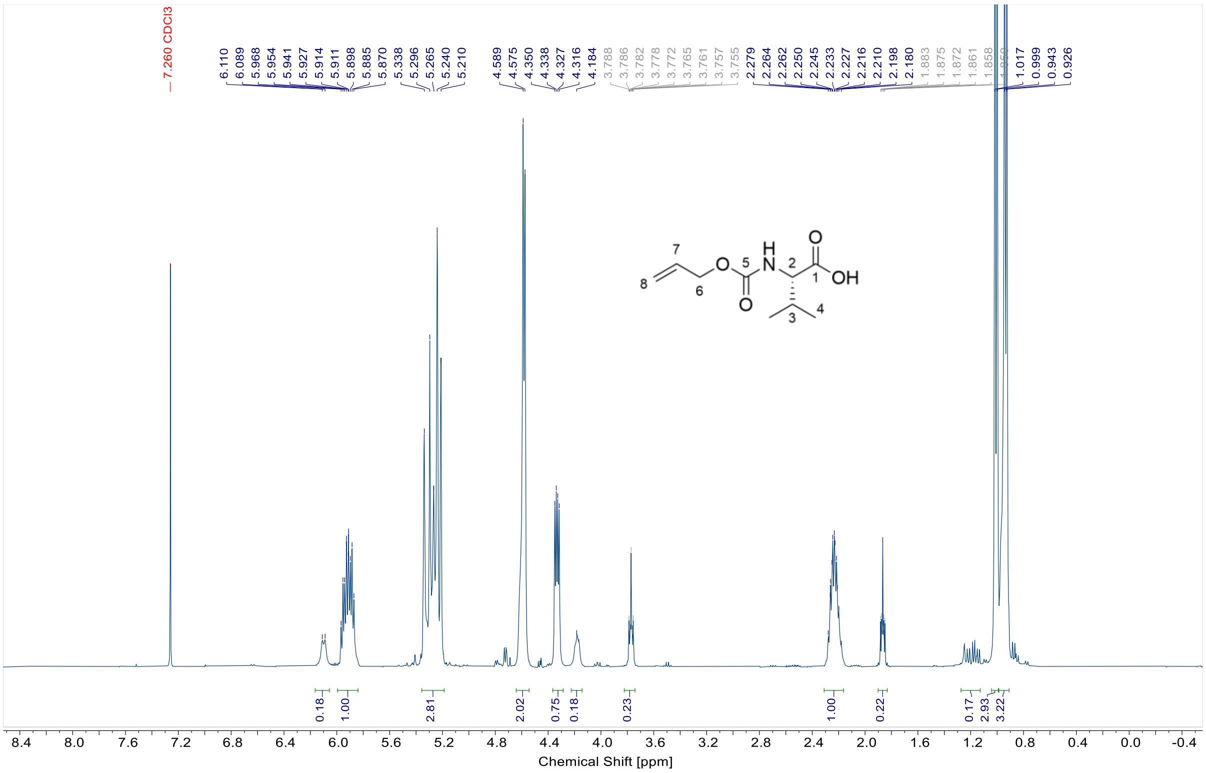

^{13}C-NMR (101 MHz, $CDCl_3$):

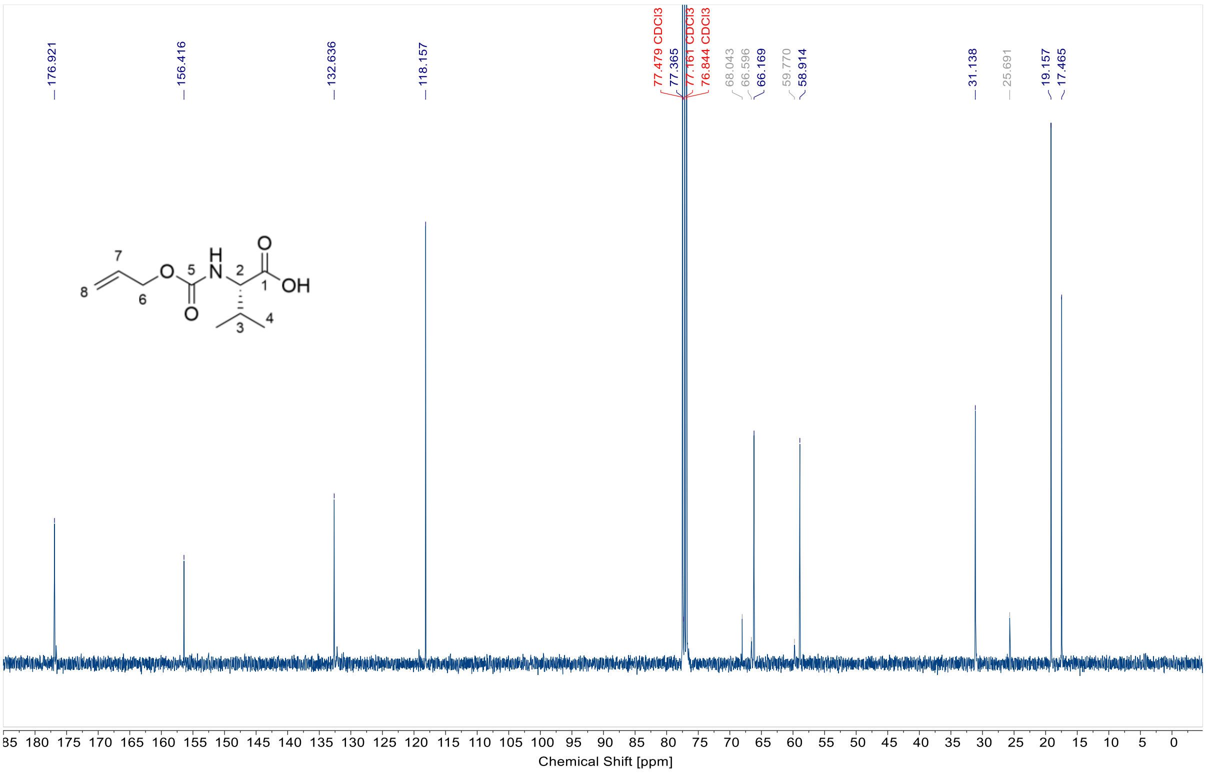

(^{1}H,^{1}H)-COSY (CDCl$_3$):

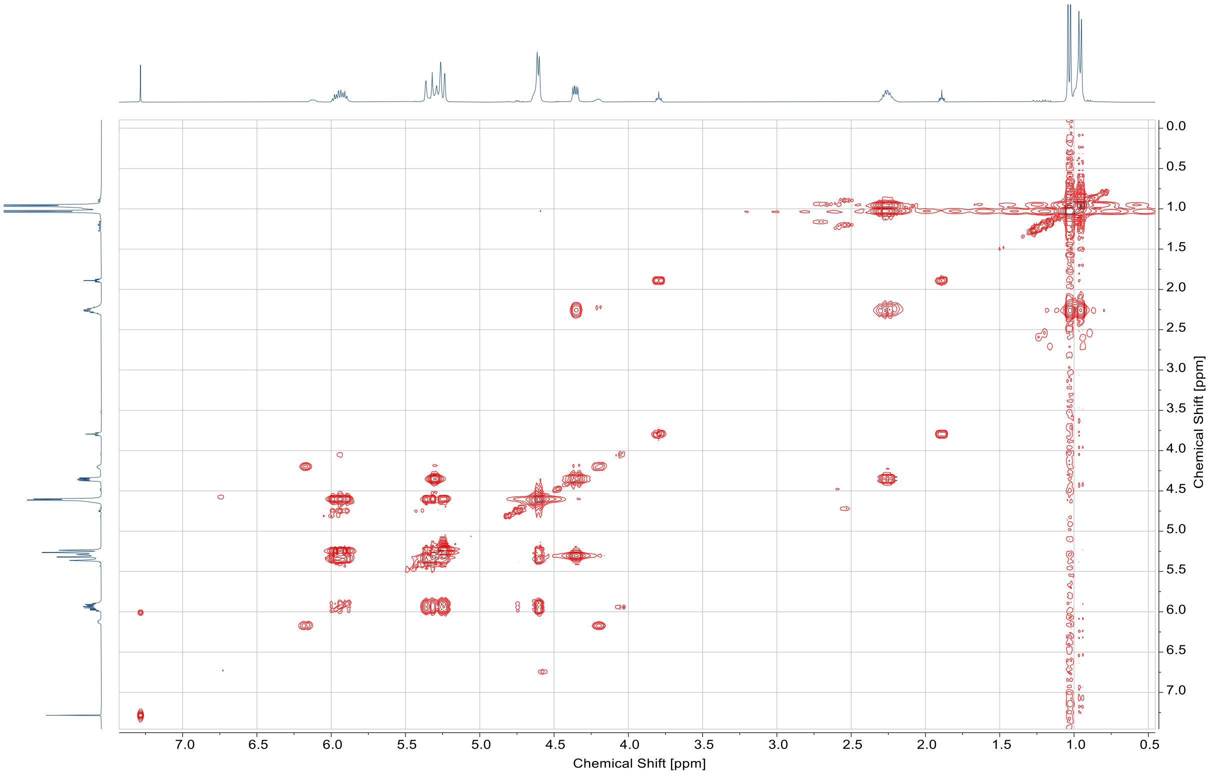

(^{1}H,^{13}C)-HSQC (CDCl$_3$):

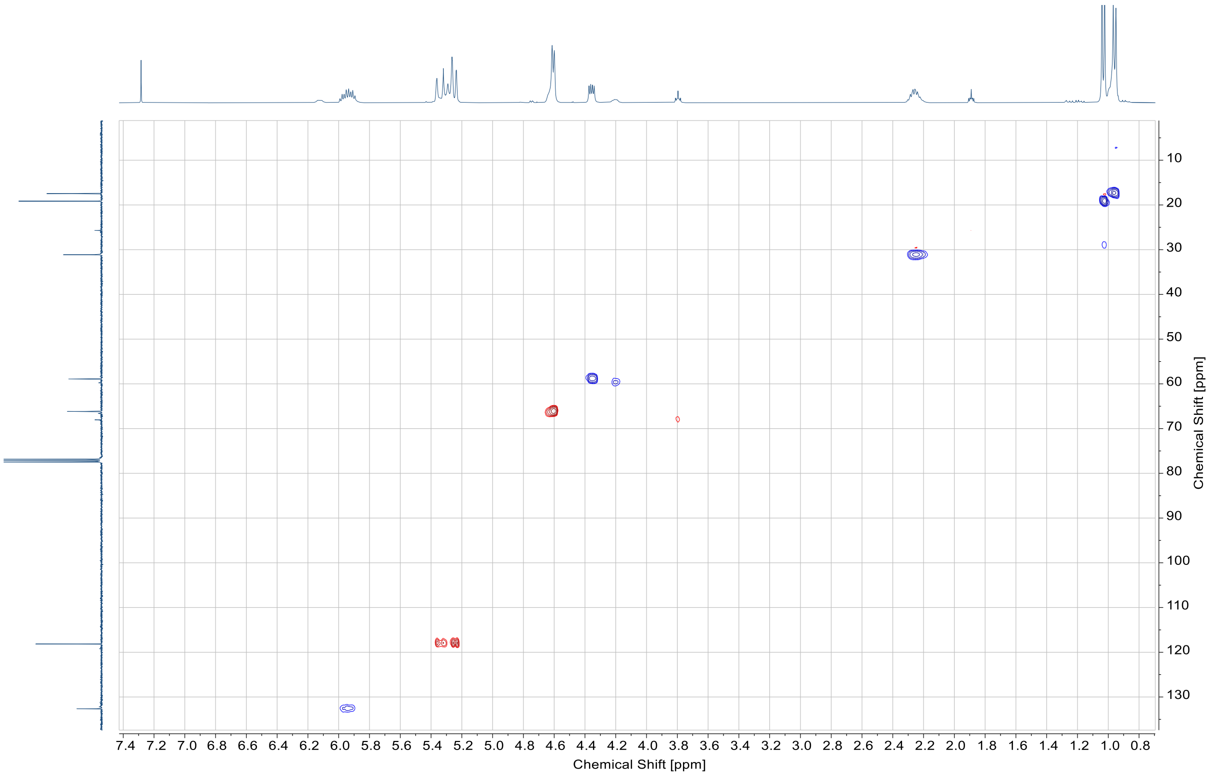

(^{1}H,^{13}C)-HMBC ($CDCl_3$):

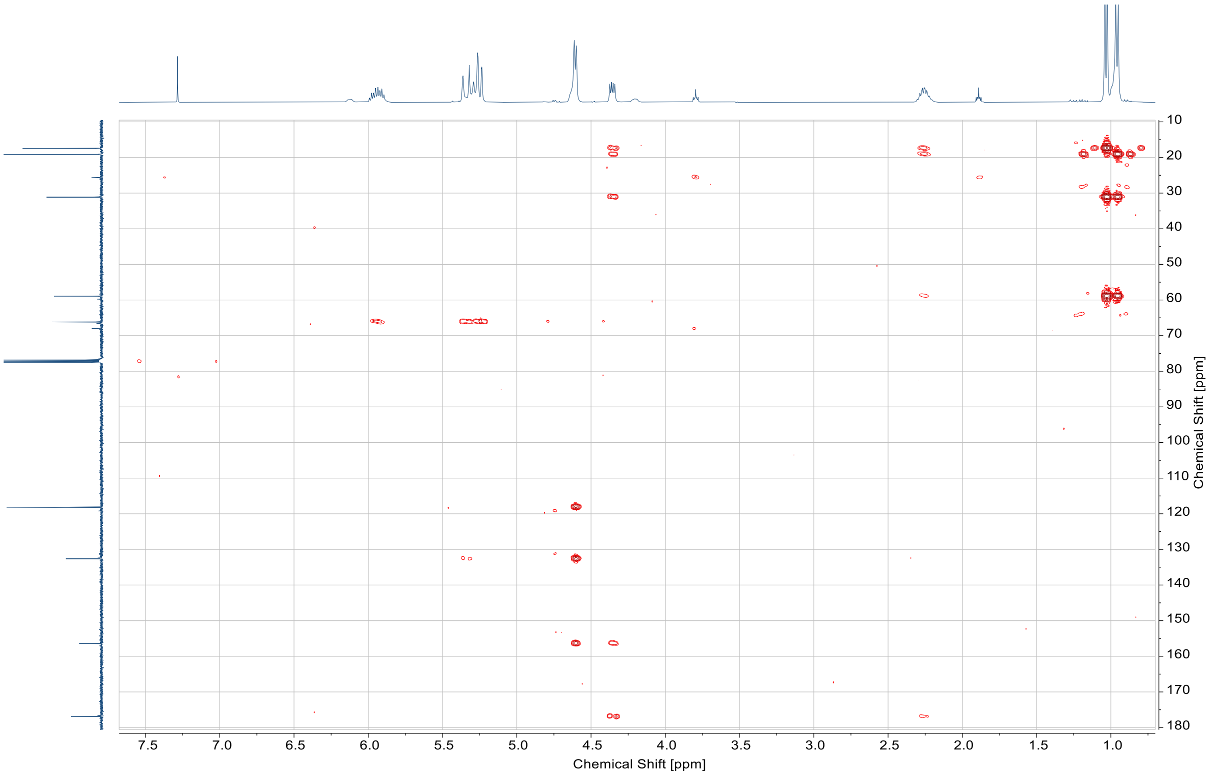

***N*-Allyloxycarbonyl-*N*-methyl-L-valine [S4]**

^{1}H-NMR (400 MHz, $CDCl_3$):

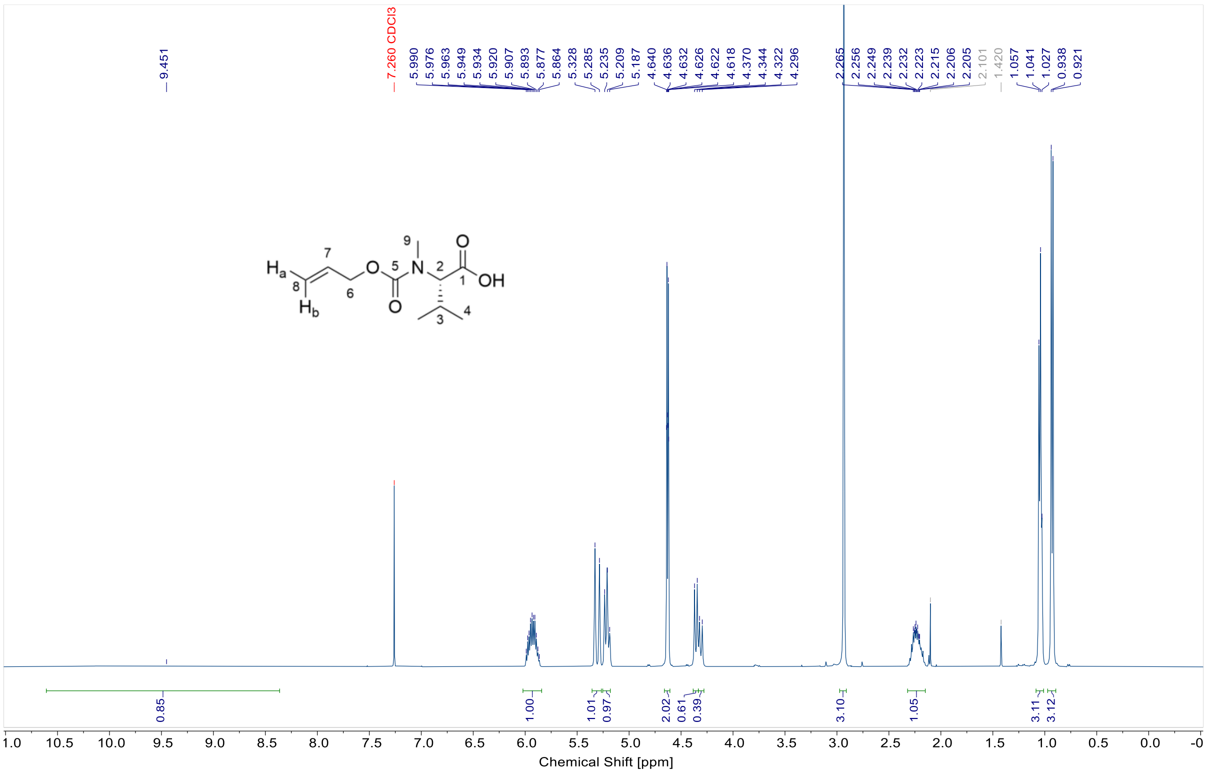

^{13}C-NMR (101 MHz, $CDCl_3$):

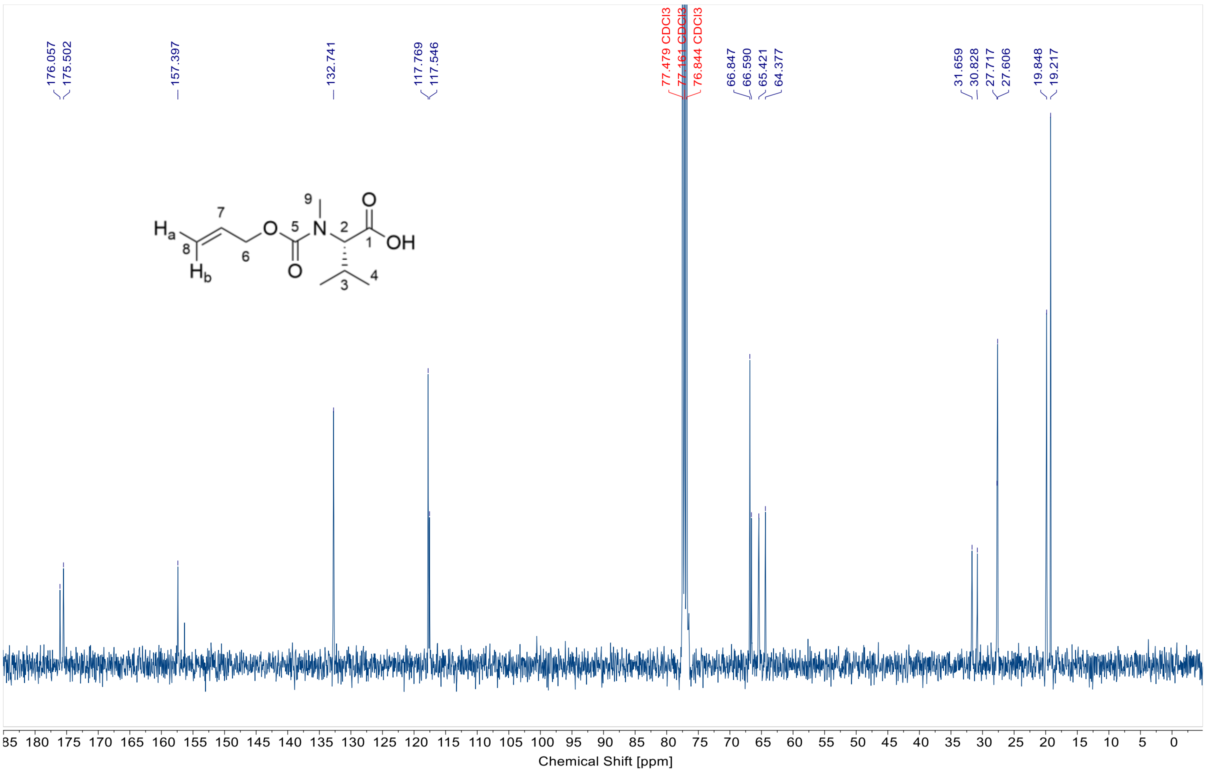

(^{1}H,^{1}H)-COSY ($CDCl_3$):

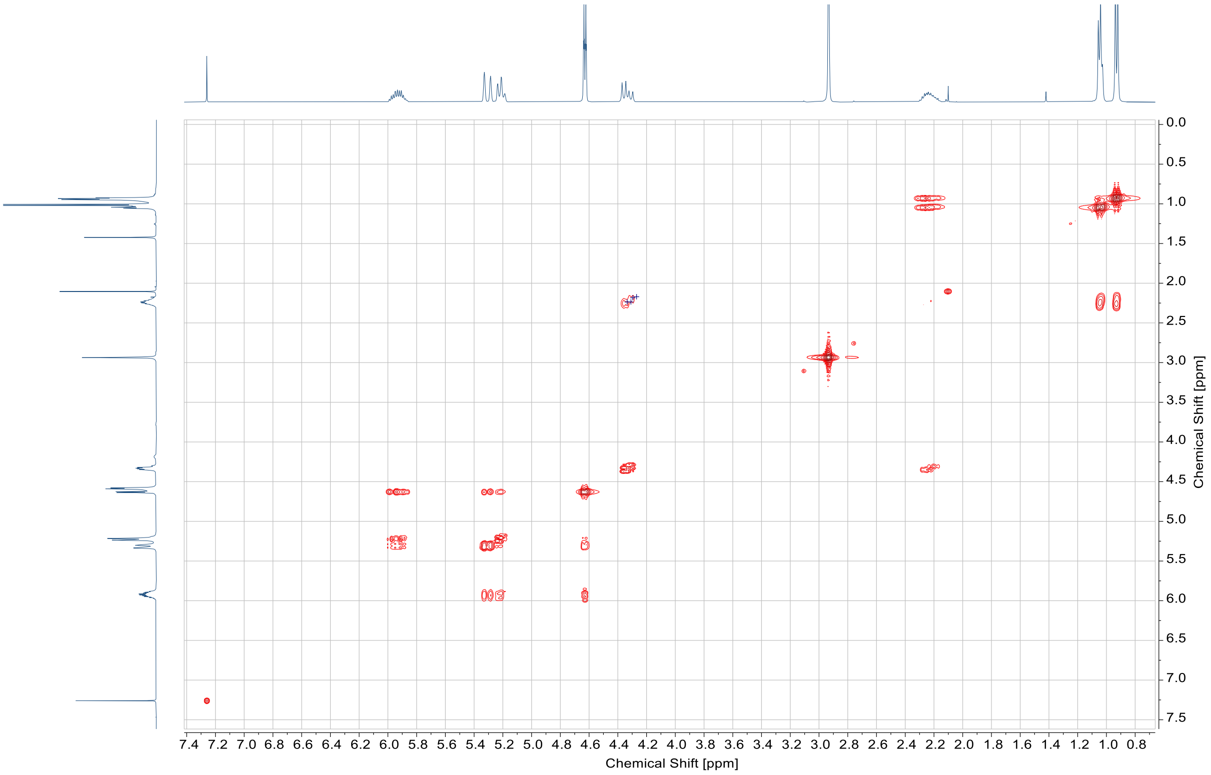

(^{1}H,^{13}C)-HSQC ($CDCl_3$):

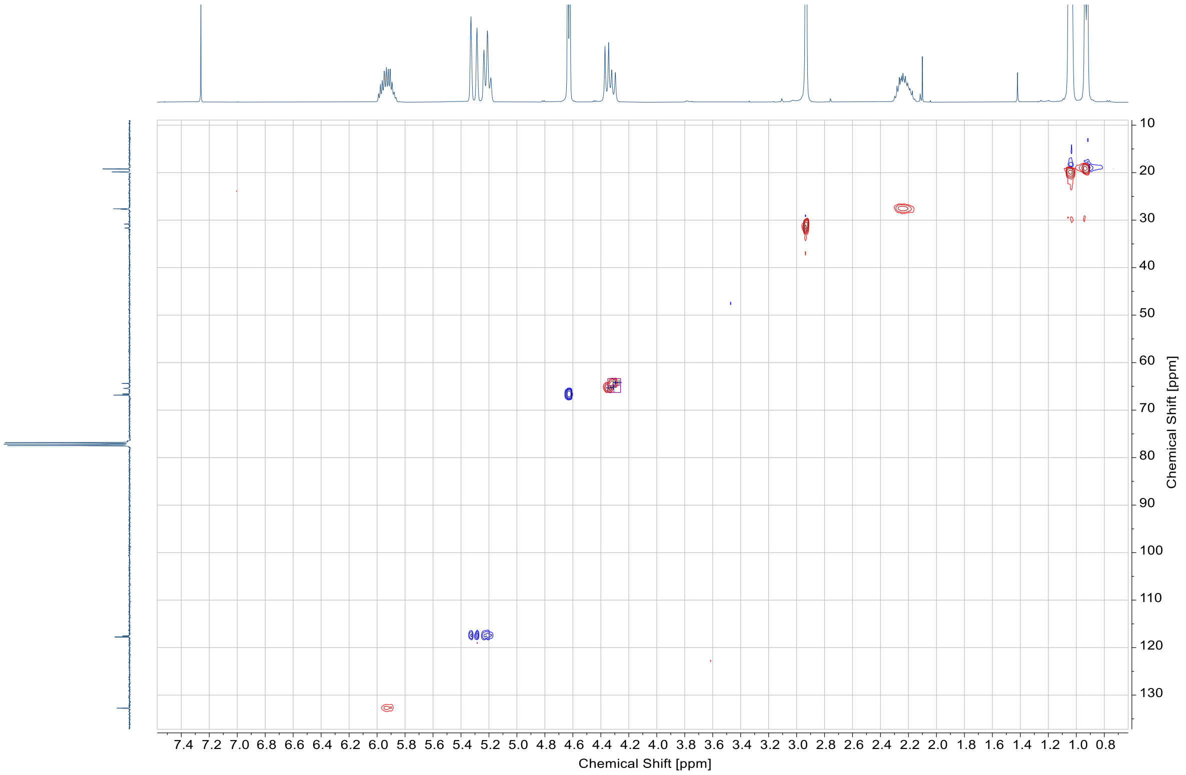

(^{1}H,^{13}C)-HMBC ($CDCl_3$):

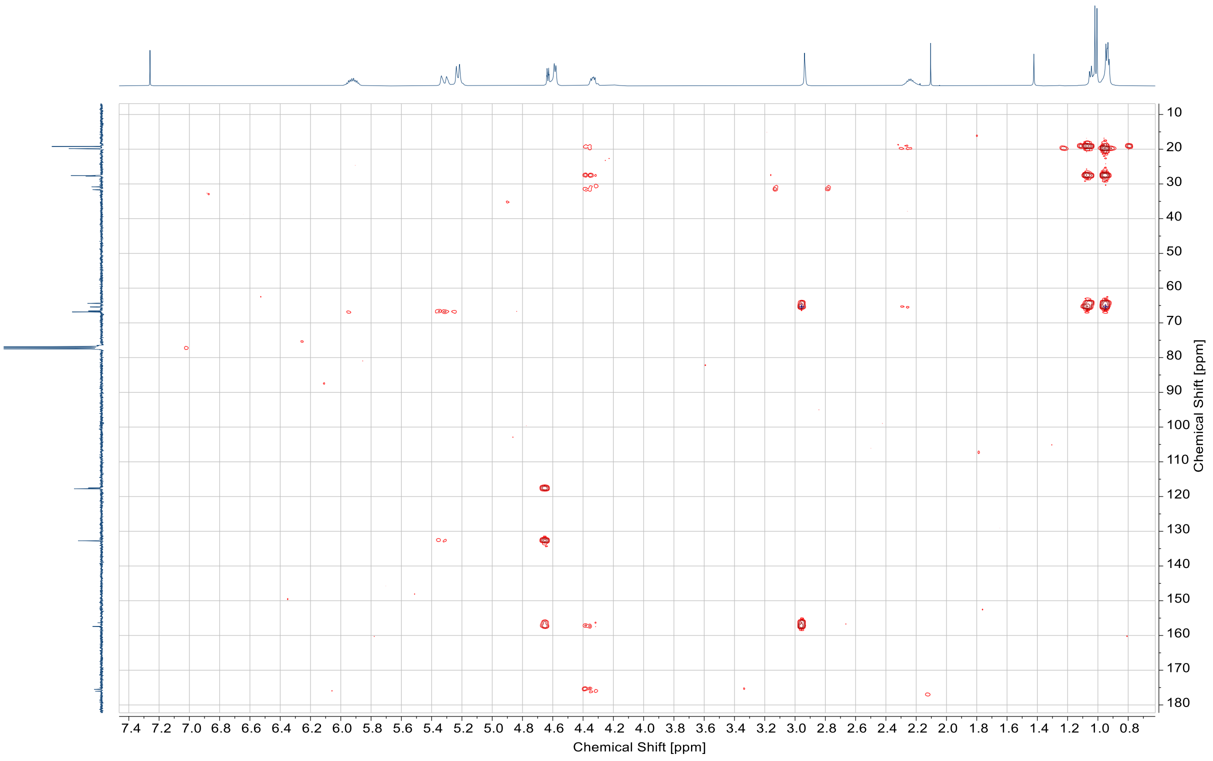

N-*tert*-Butyloxycarbonyl-*N*-methyl-D-leucine [S5]

^{1}H-NMR (400 MHz, $CDCl_3$):

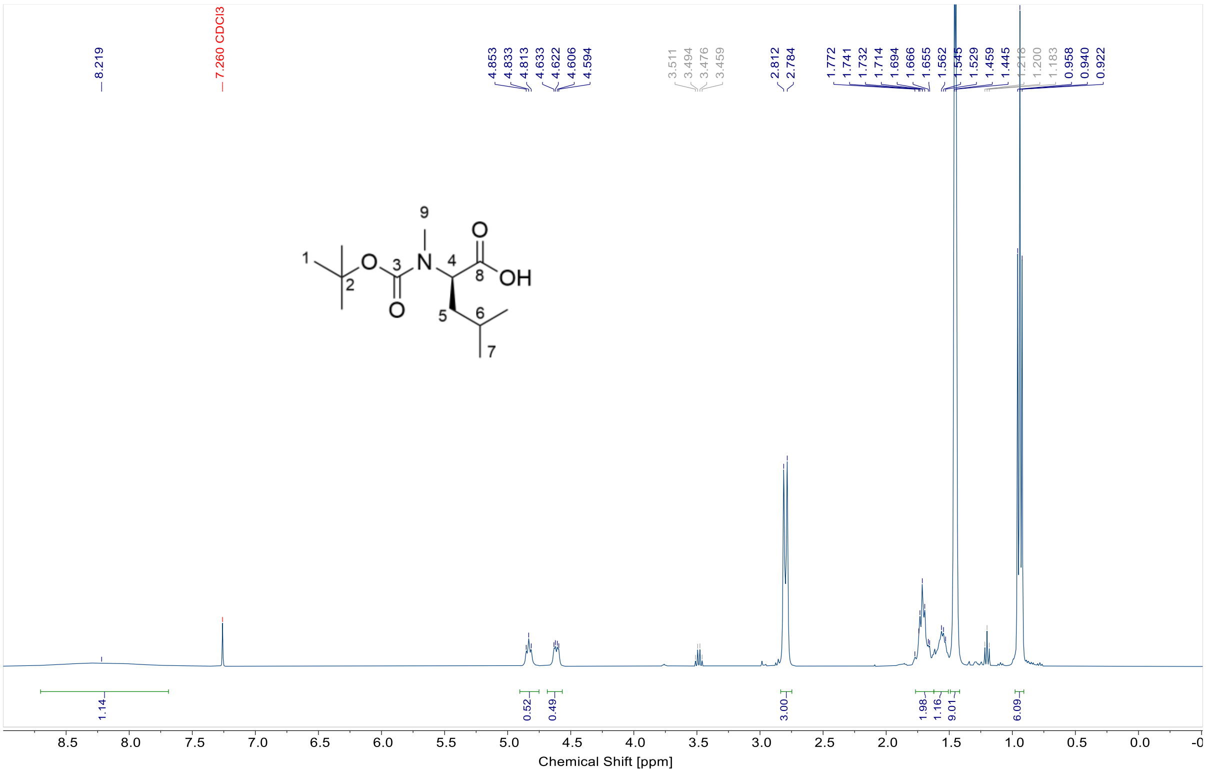

^{13}C-NMR (101 MHz, $CDCl_3$):

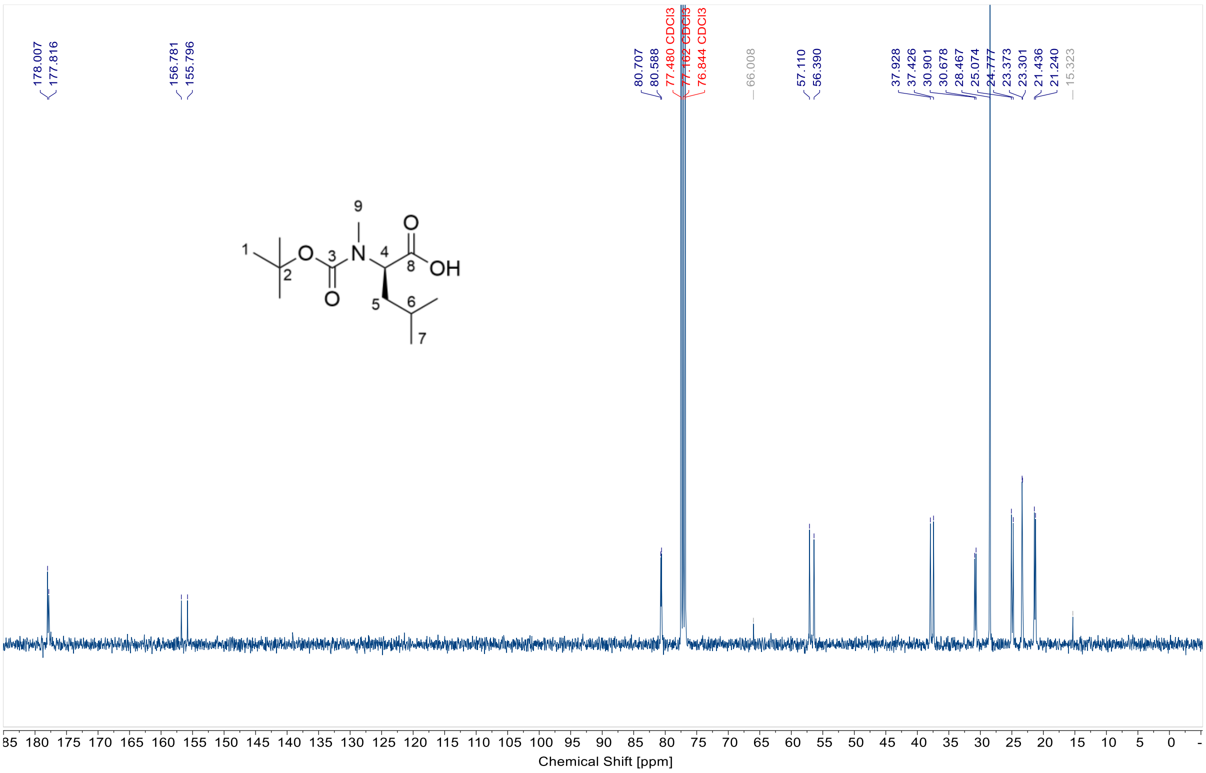

(^{1}H,^{1}H)-COSY ($CDCl_3$):

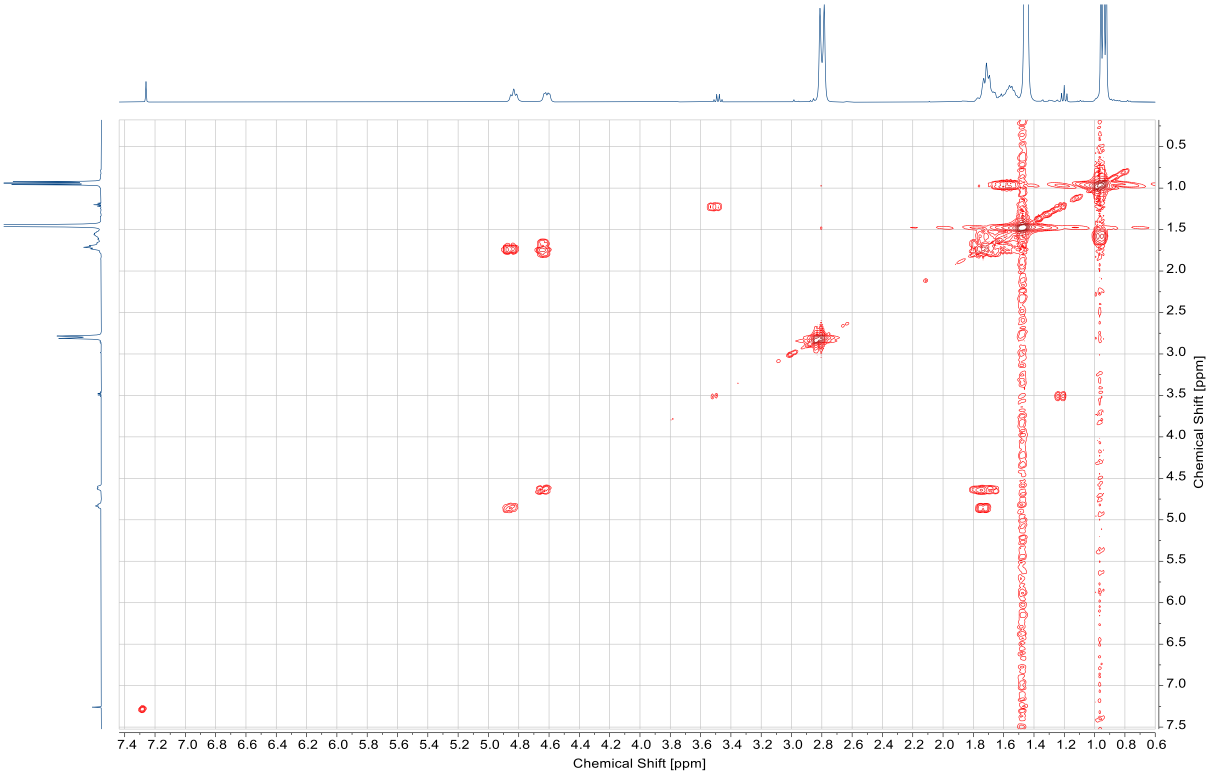

(^{1}H,^{13}C)-HSQC ($CDCl_3$):

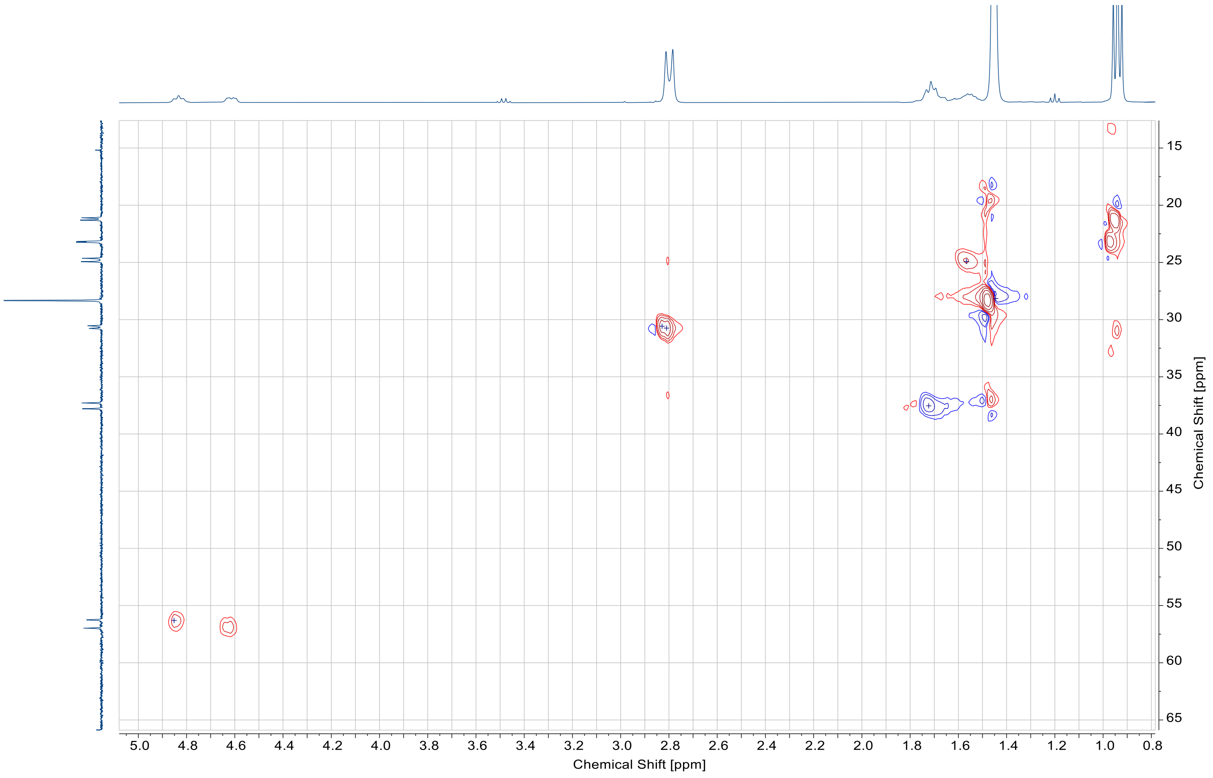

(^{1}H,^{13}C)-HMBC ($CDCl_3$):

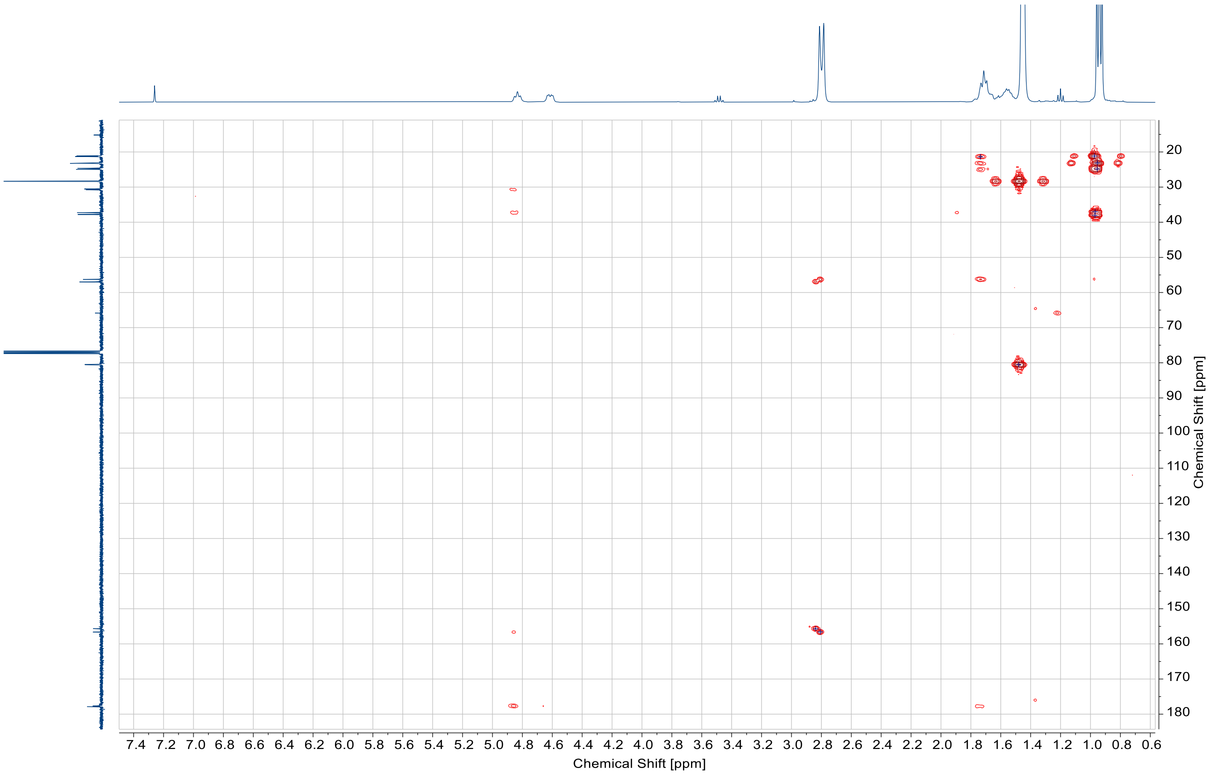

Methyl *N*-*tert*-butyloxycarbonyl-*N*-methyl-D-leucinate [19]

^{1}H-NMR (500 MHz, $CDCl_3$):

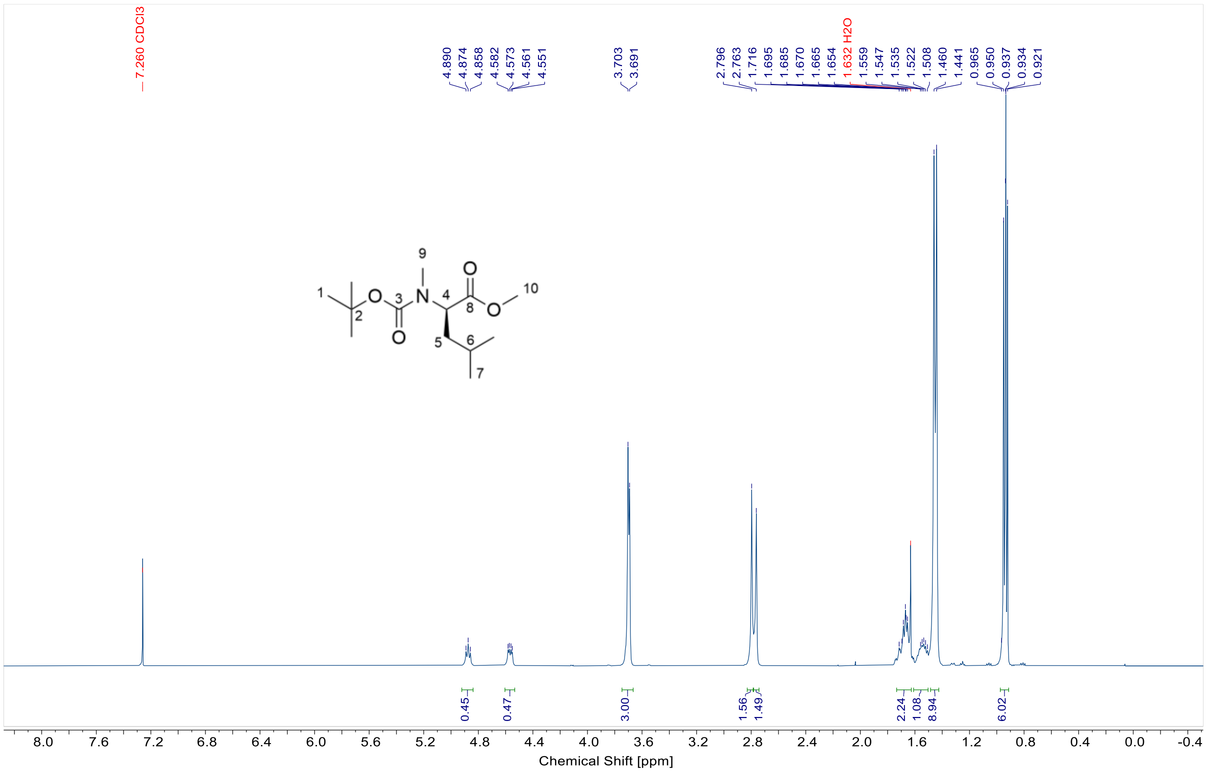

^{13}C-NMR (126 MHz, $CDCl_3$):

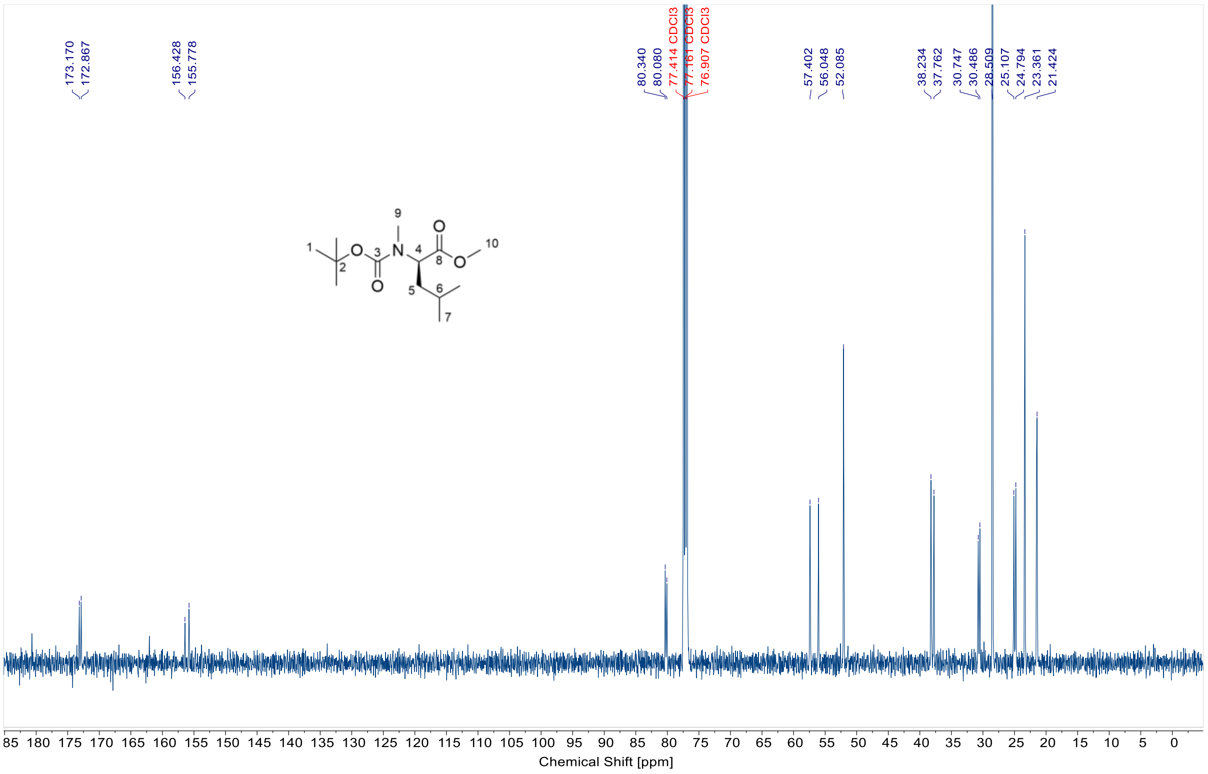

(^{1}H,^{1}H)-COSY ($CDCl_3$)

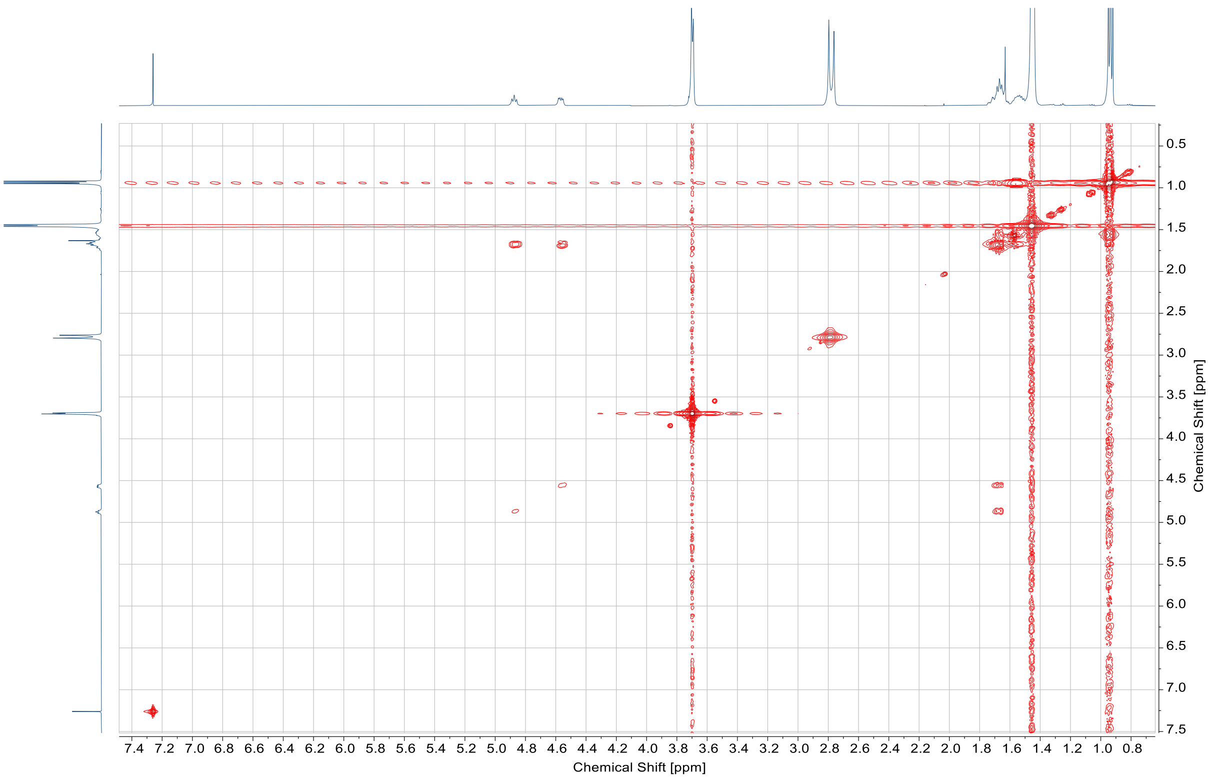

(^{1}H,^{13}C)-HSQC ($CDCl_3$):

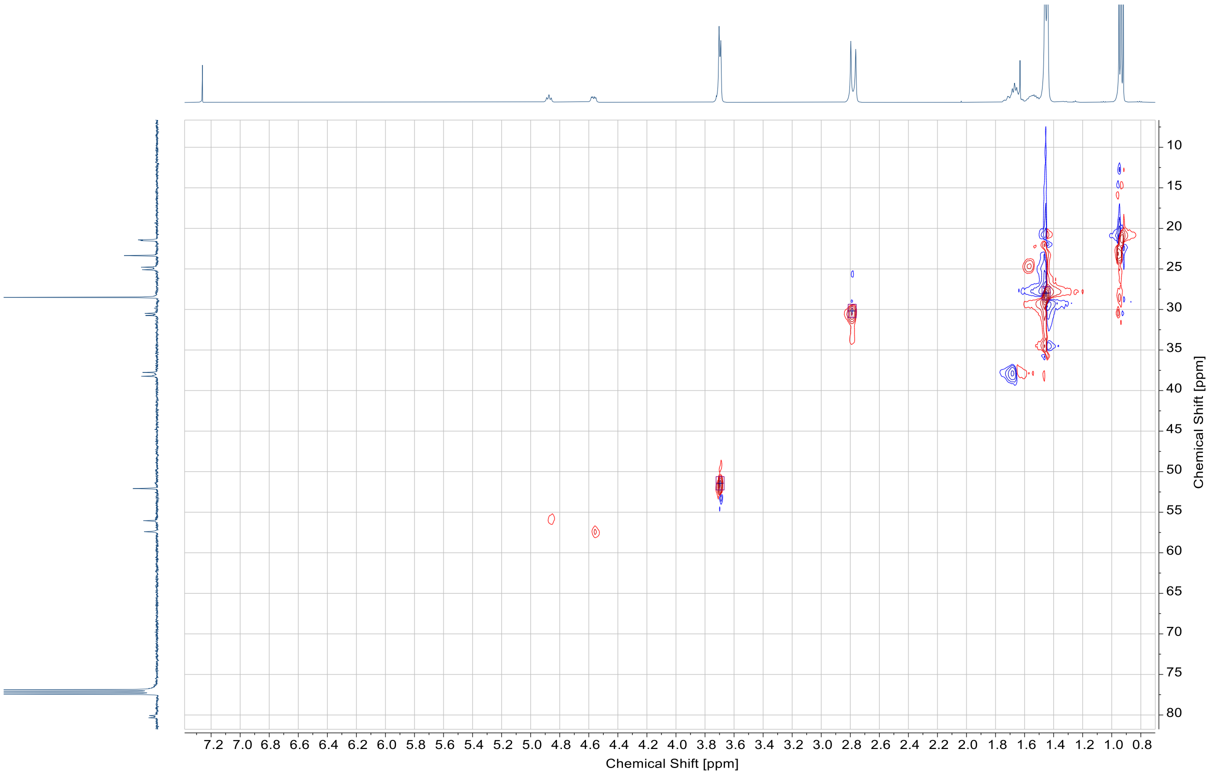

(^{1}H,^{13}C)-HMBC ($CDCl_3$)

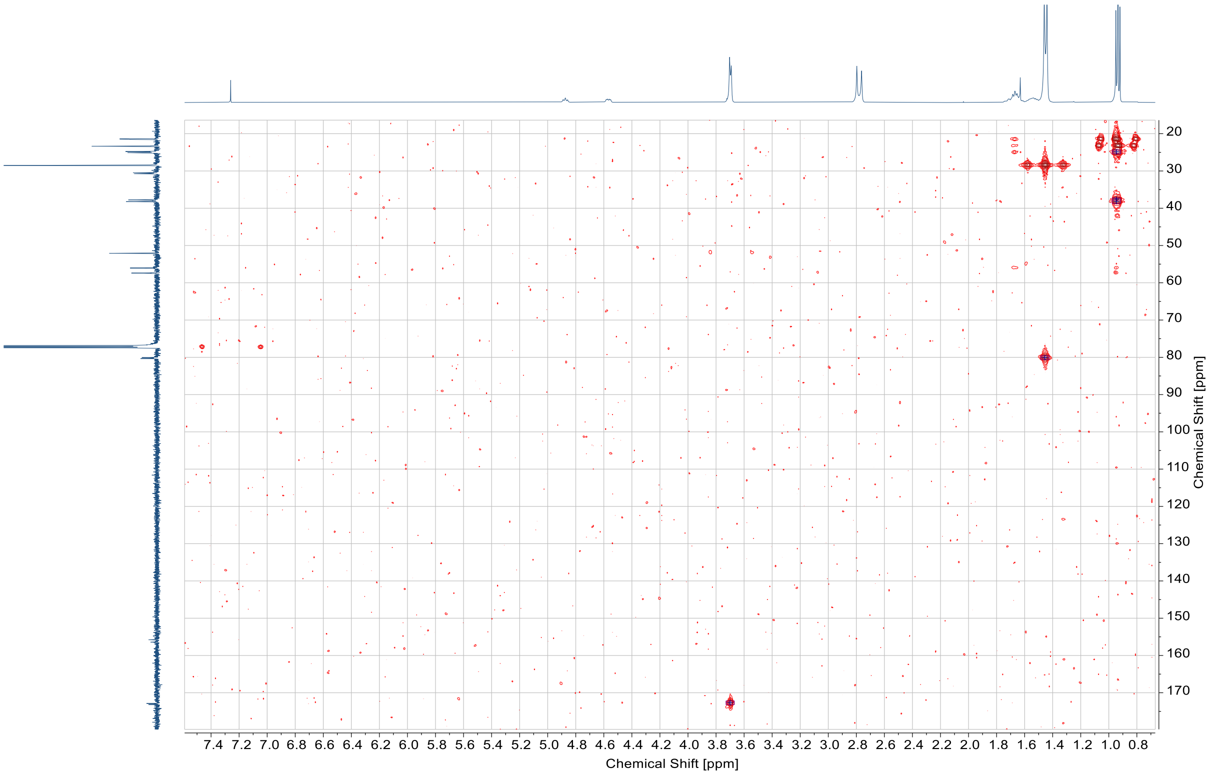

Cbz-L-Leu-L-MePro-O*t*-Bu [14]

^{1}H-NMR (500 MHz, DMSO-d_6, 373 K):

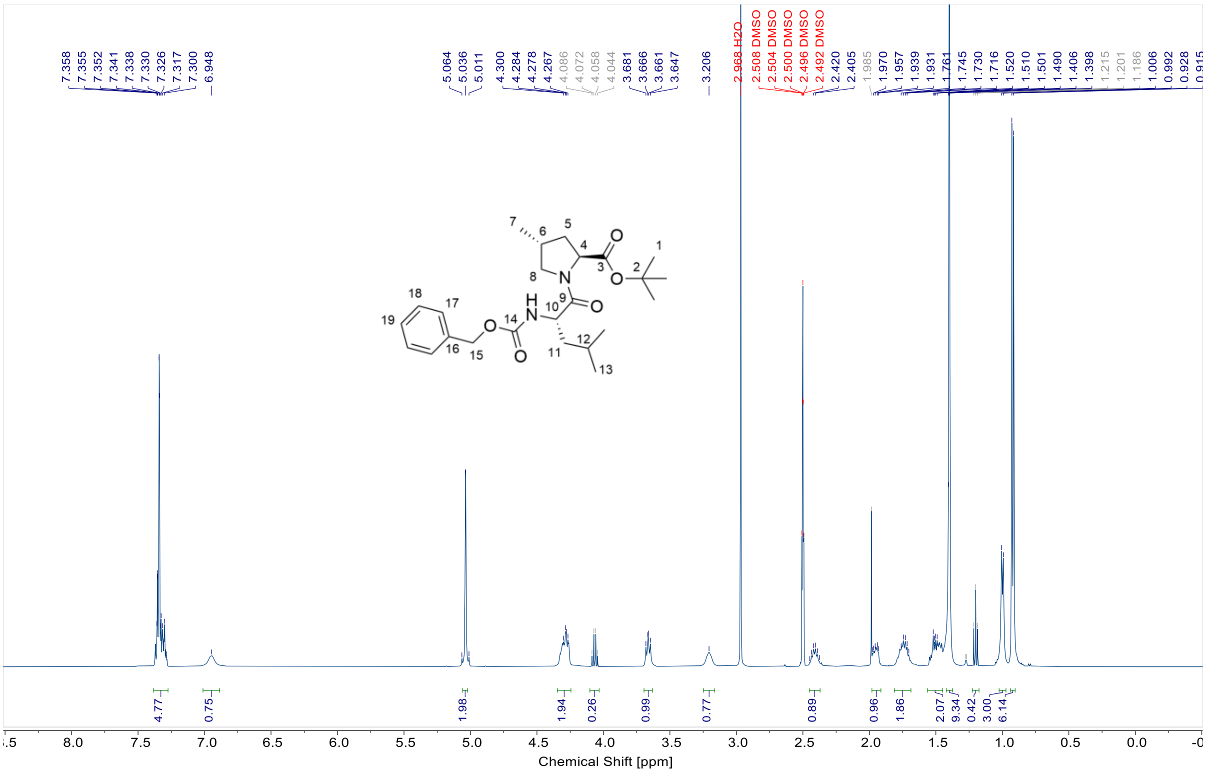

^{13}C-NMR (126 MHz, DMSO-d_6, 373 K):

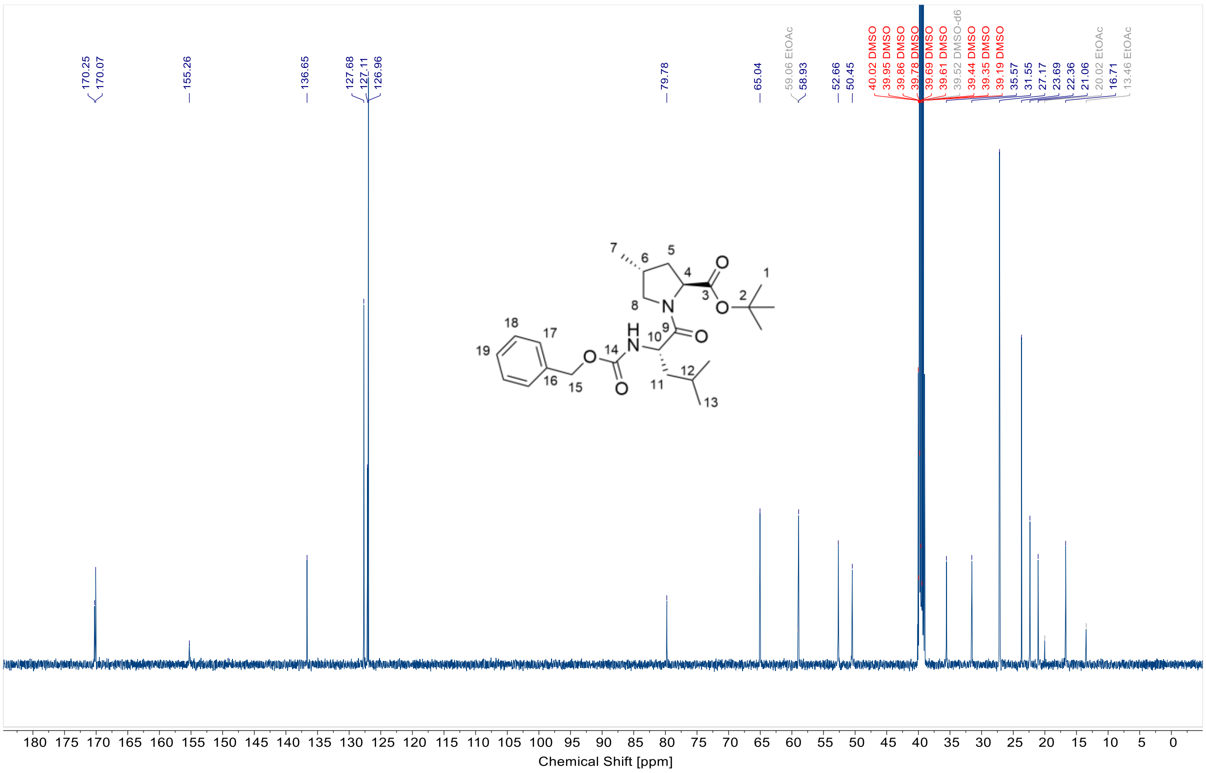

(^{1}H,^{1}H)-COSY (DMSO-d_6, 373 K):

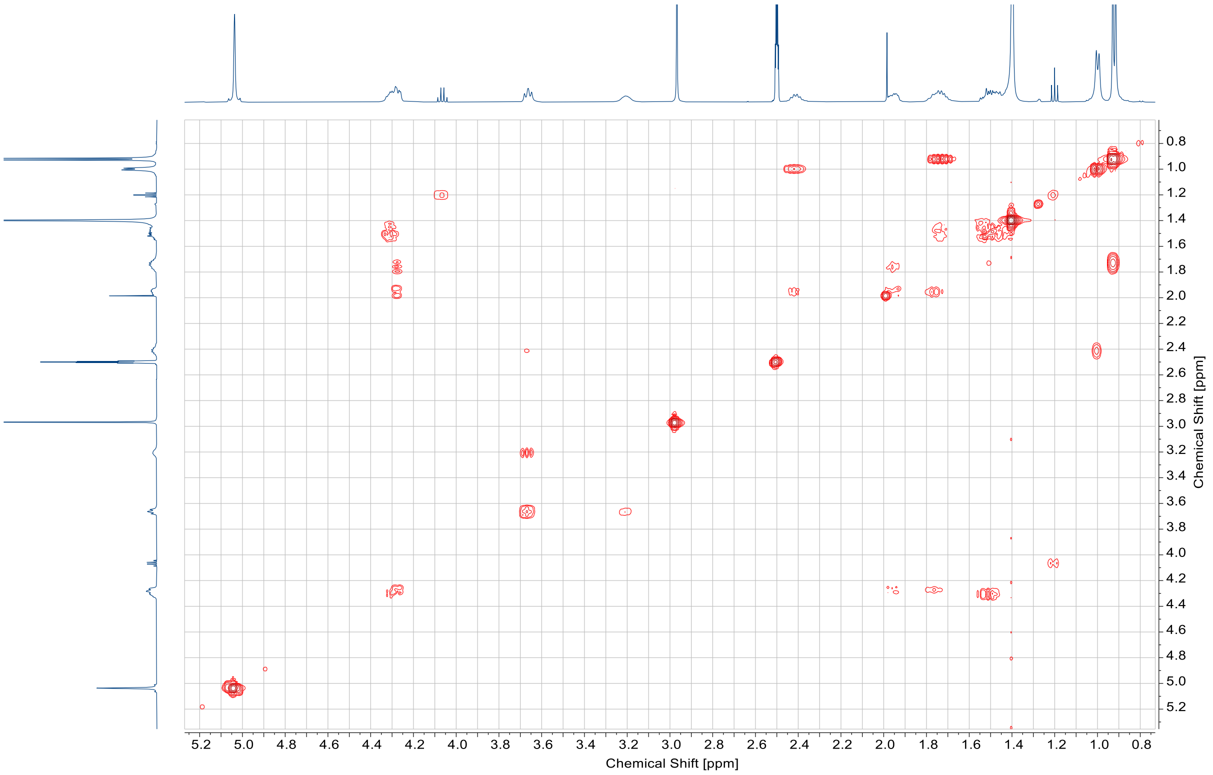

(^{1}H,^{13}C)-HSQC (DMSO-d_6, 373 K):

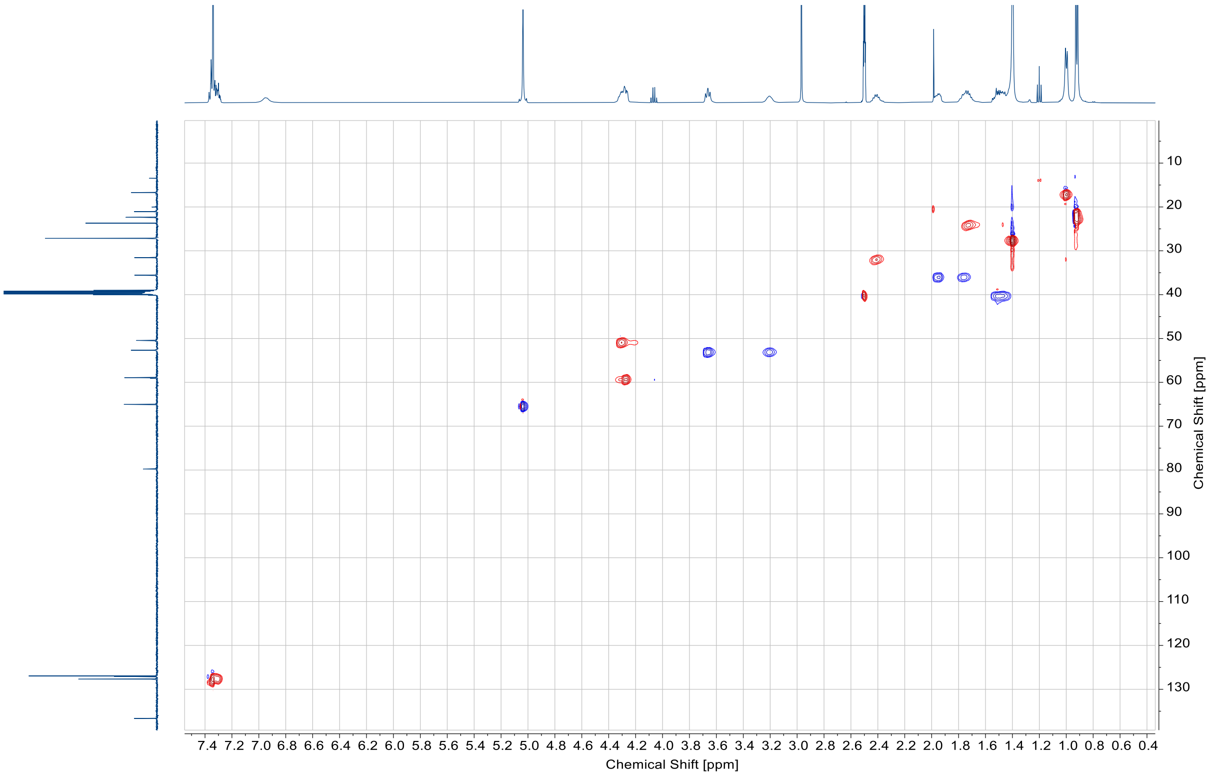

(^{1}H,^{13}C)-HMBC (DMSO-d_6, 373 K):

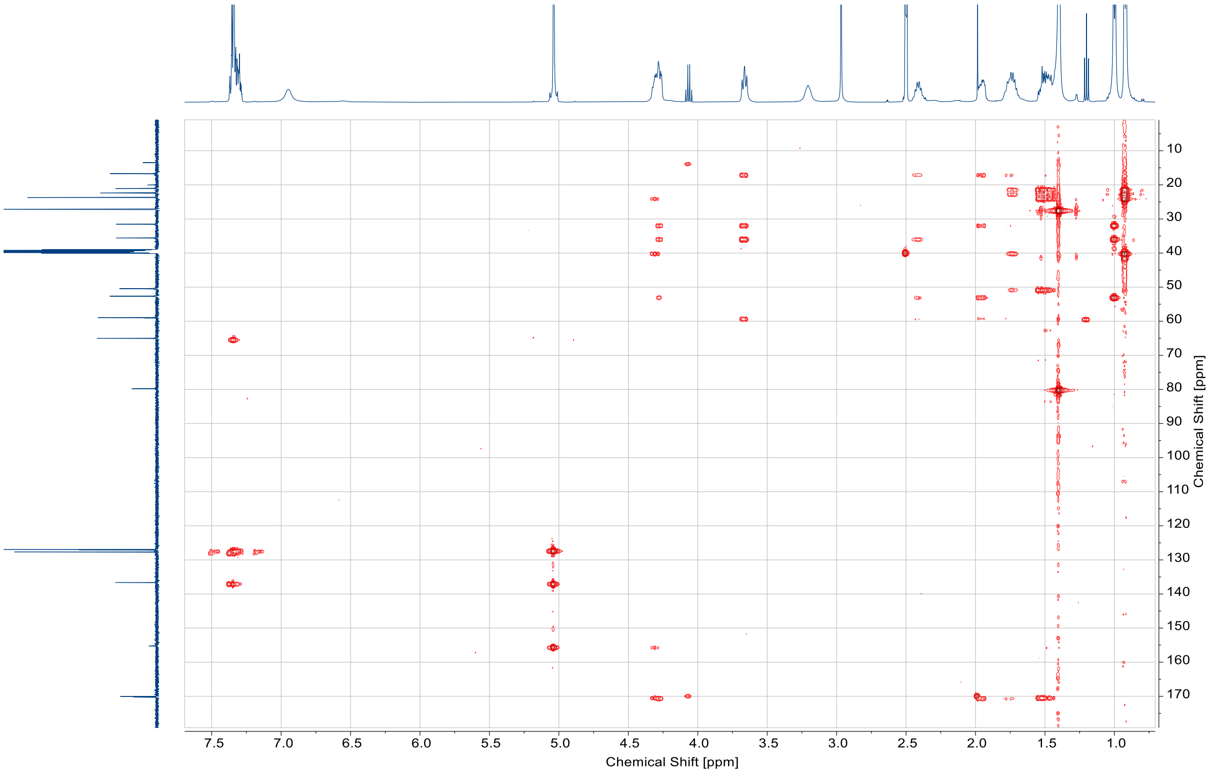

Cbz-*O*-TBS-*N*-Me-L-Thr-L-Leu-L-MePro-O*t*-Bu [15]

^{1}H-NMR (500 MHz, DMSO-d_6, 373 K):

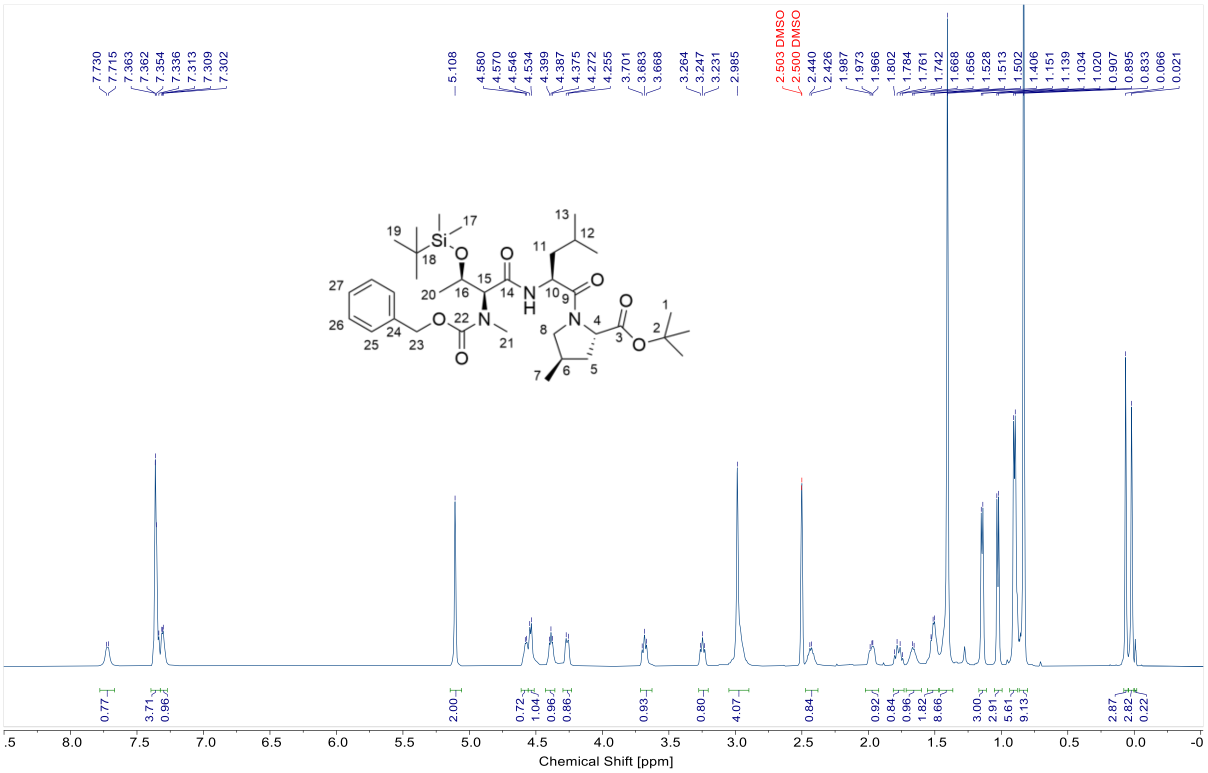

^{13}C-NMR (126 MHz, DMSO-d_6, 373 K):

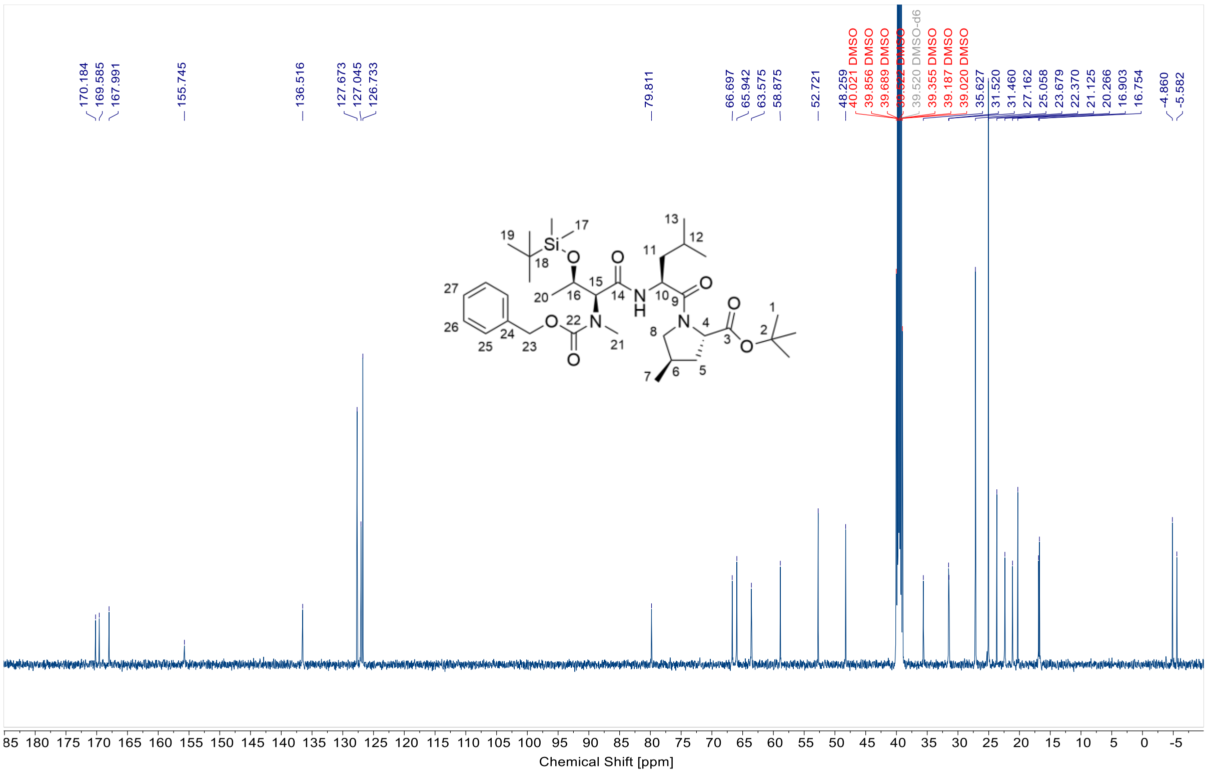

(^{1}H,^{1}H)-COSY (DMSO-d_6, 373 K):

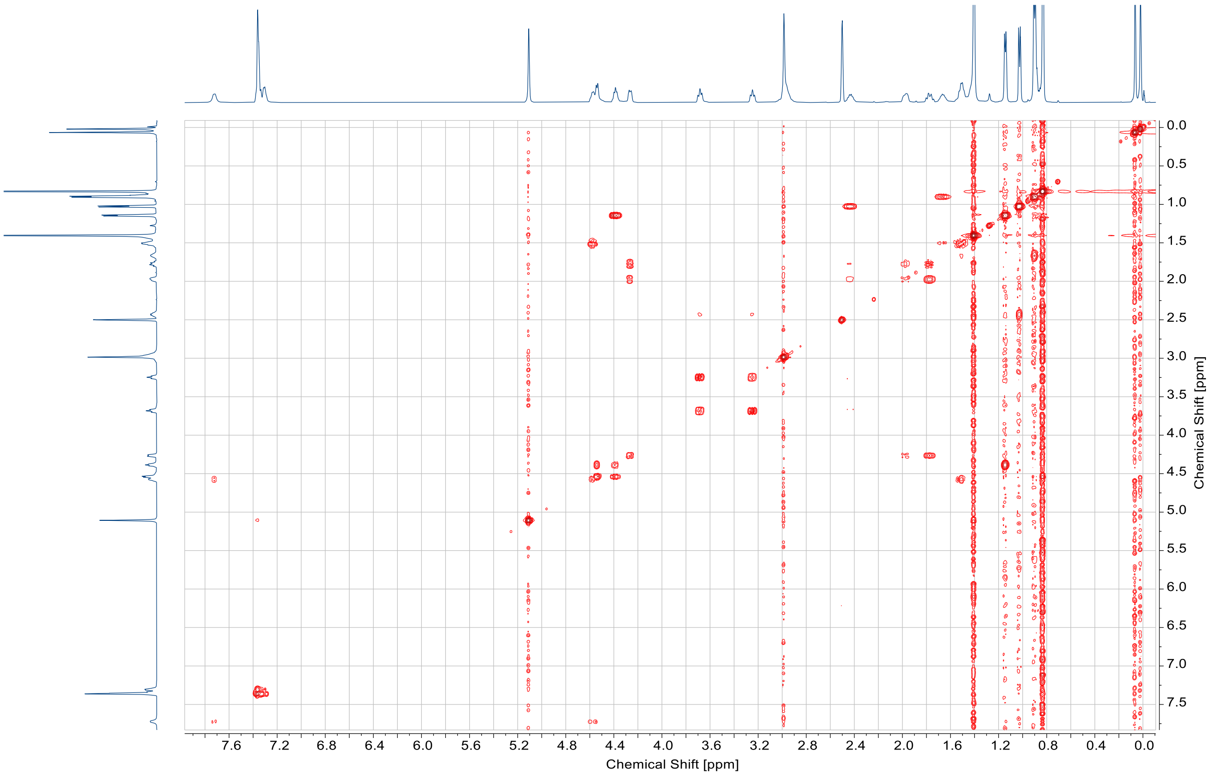

(^{1}H,^{13}C)-HSQC (DMSO-d_6, 373 K):

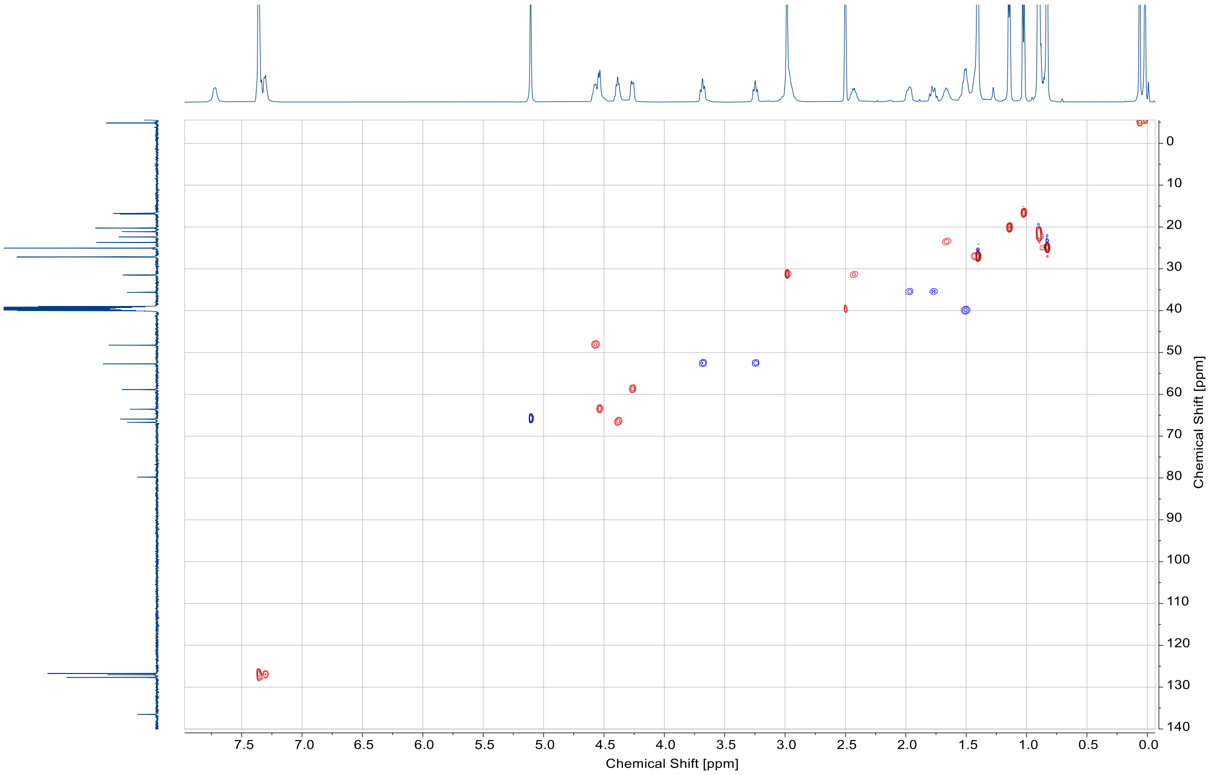

(^{1}H,^{13}C)-HMBC (DMSO-d_6, 373 K)

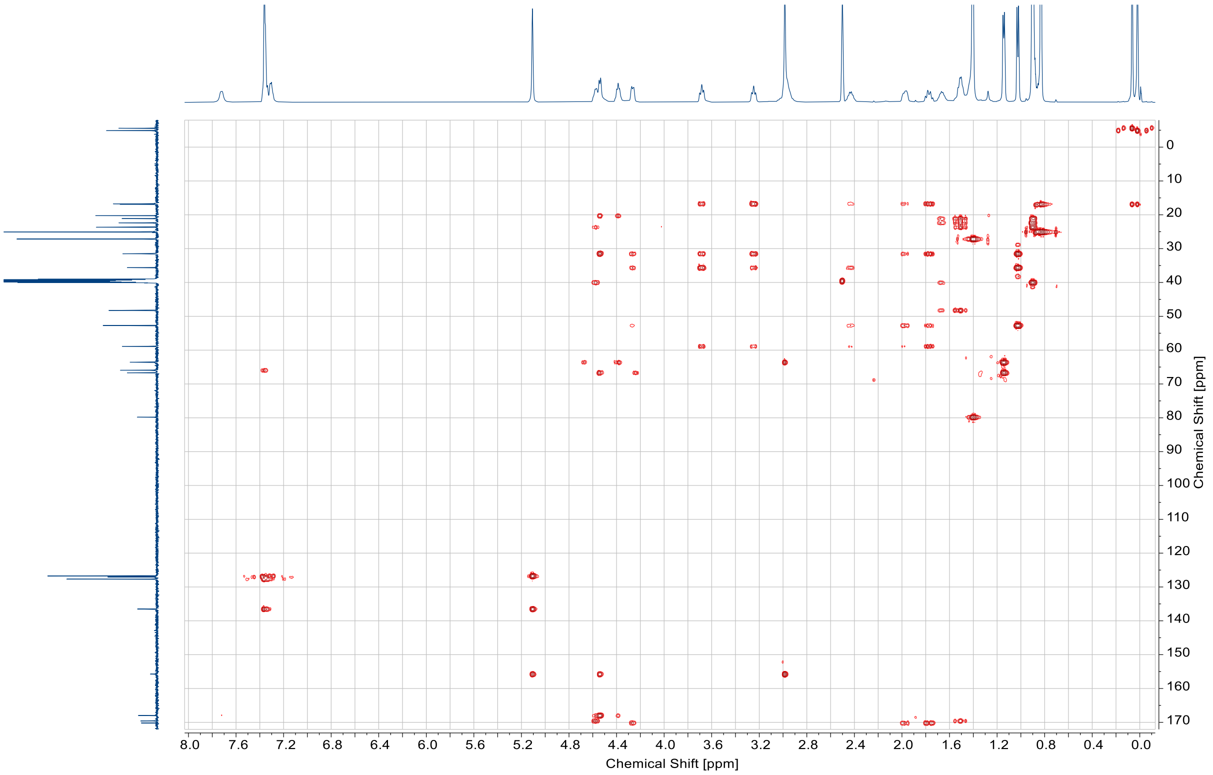

Cbz-*N*-Me-L-Thr-L-Leu-L-MePro-O*t*-Bu [16’]

¹H-NMR (500 MHz, $CDCl_3$):

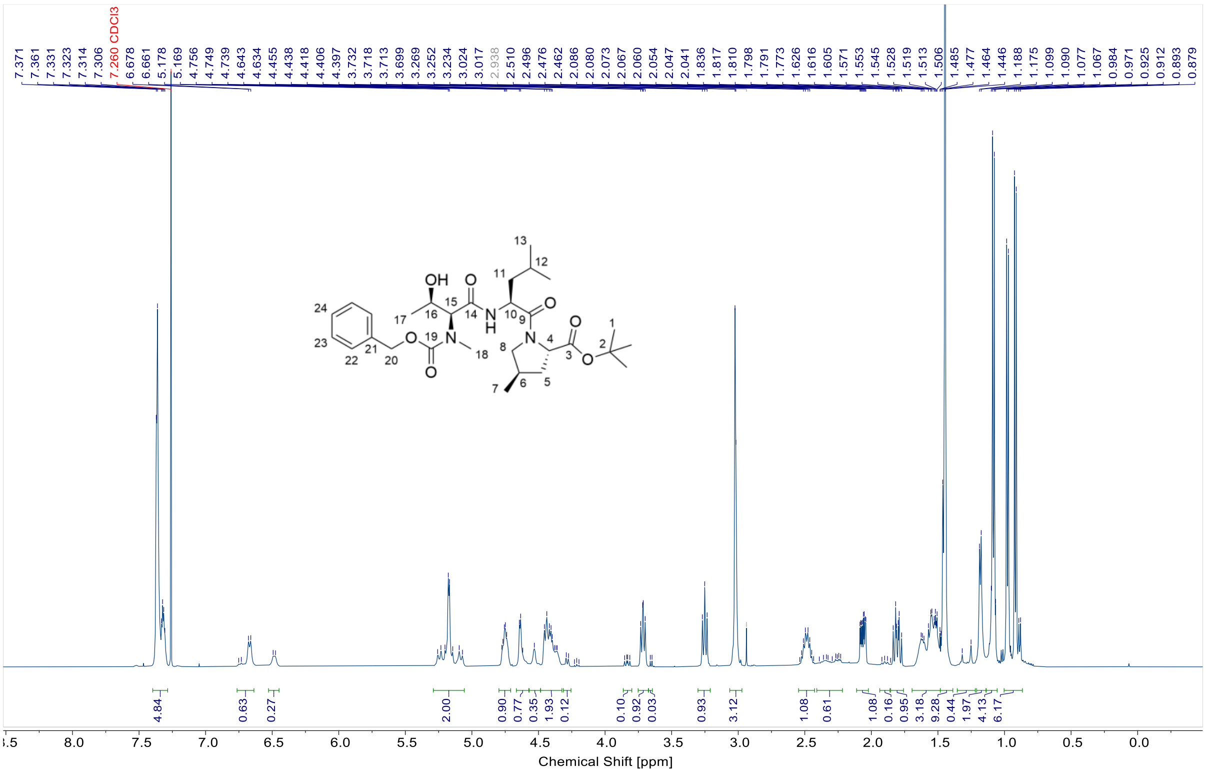

¹³C-NMR (500 MHz, $CDCl_3$):

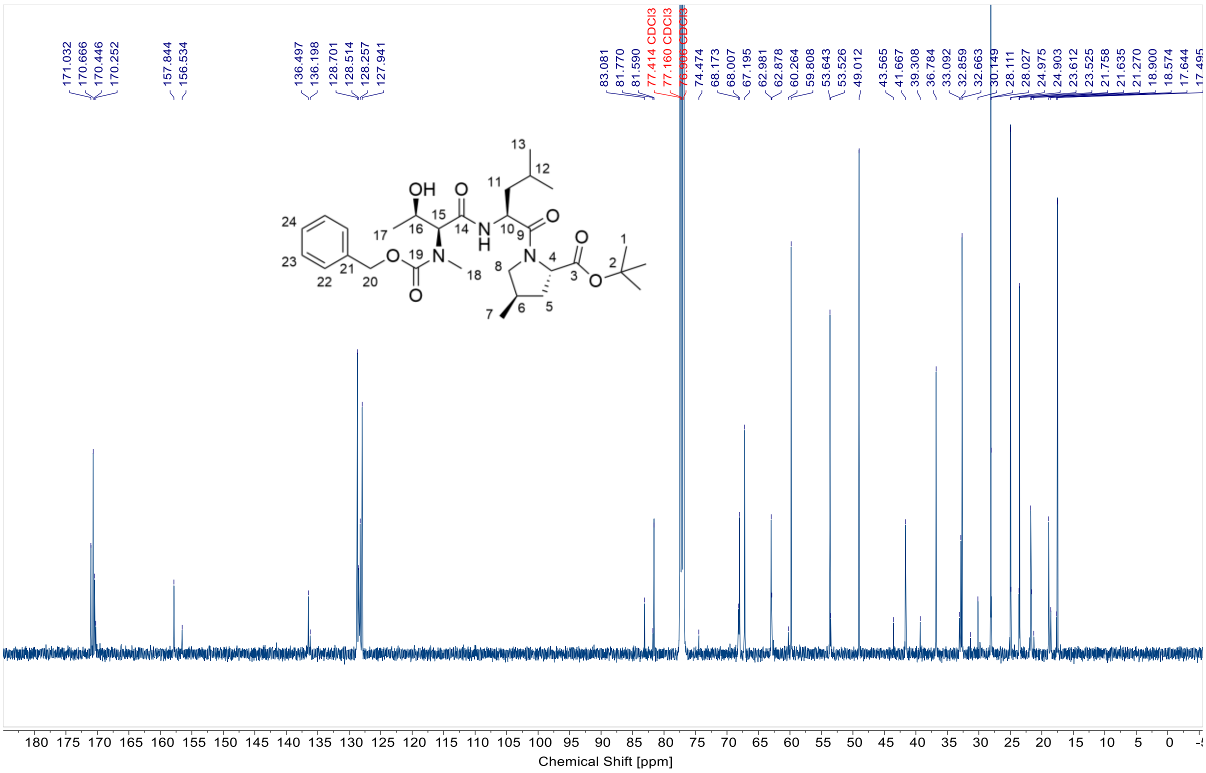

(^{1}H,^{1}H)-COSY (CDCl$_3$):

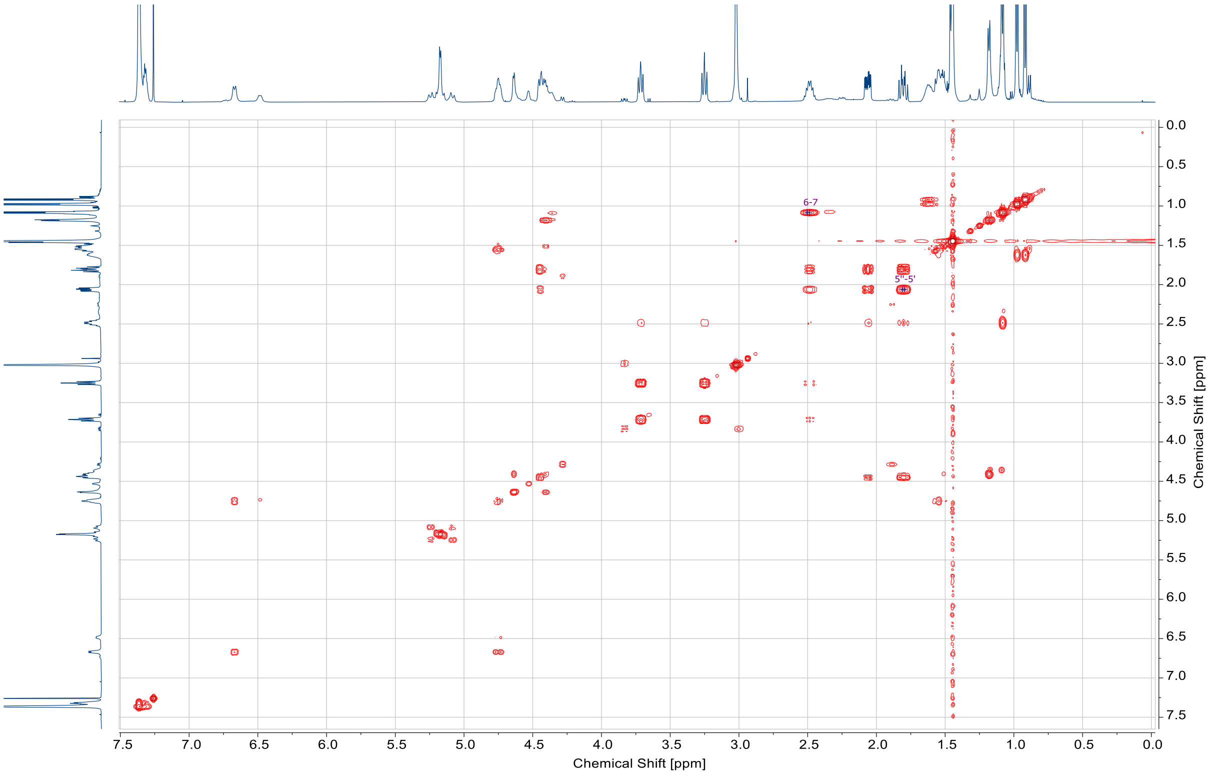

(^{1}H,^{13}C)-HSQC (CDCl$_3$):

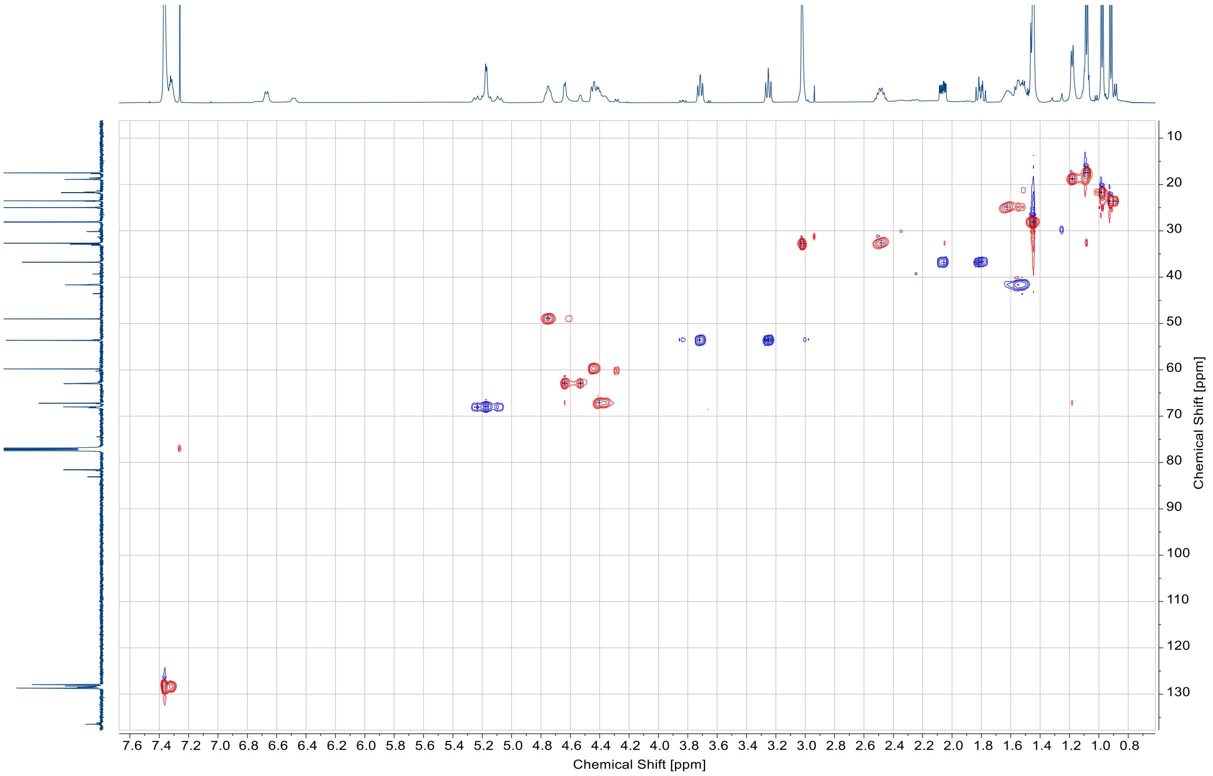

(^{1}H,^{13}C)-HMBC (CDCl$_3$):

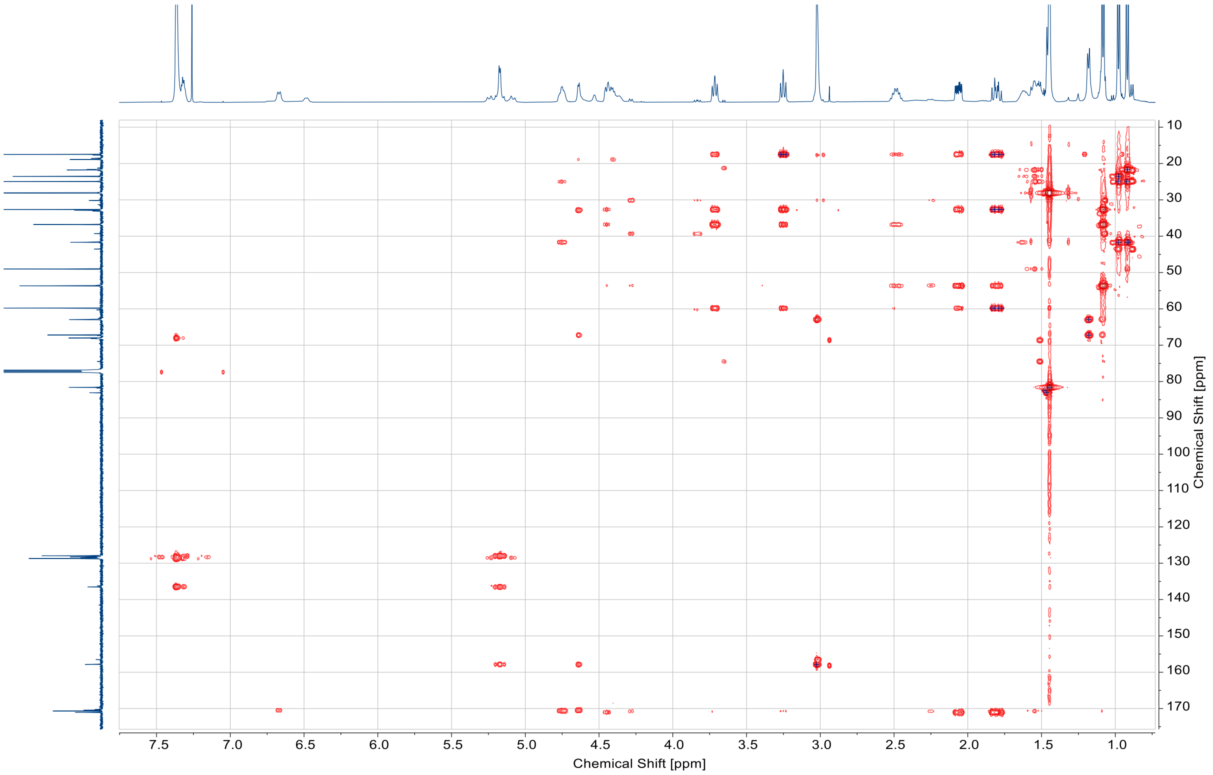

Cbz-*O*-(Cbz-EtPro)-*N*-Me-L-Thr-L-Leu-L-MePro-O*t*-Bu [16]

^{1}H-NMR (500 MHz, CDCl$_3$):

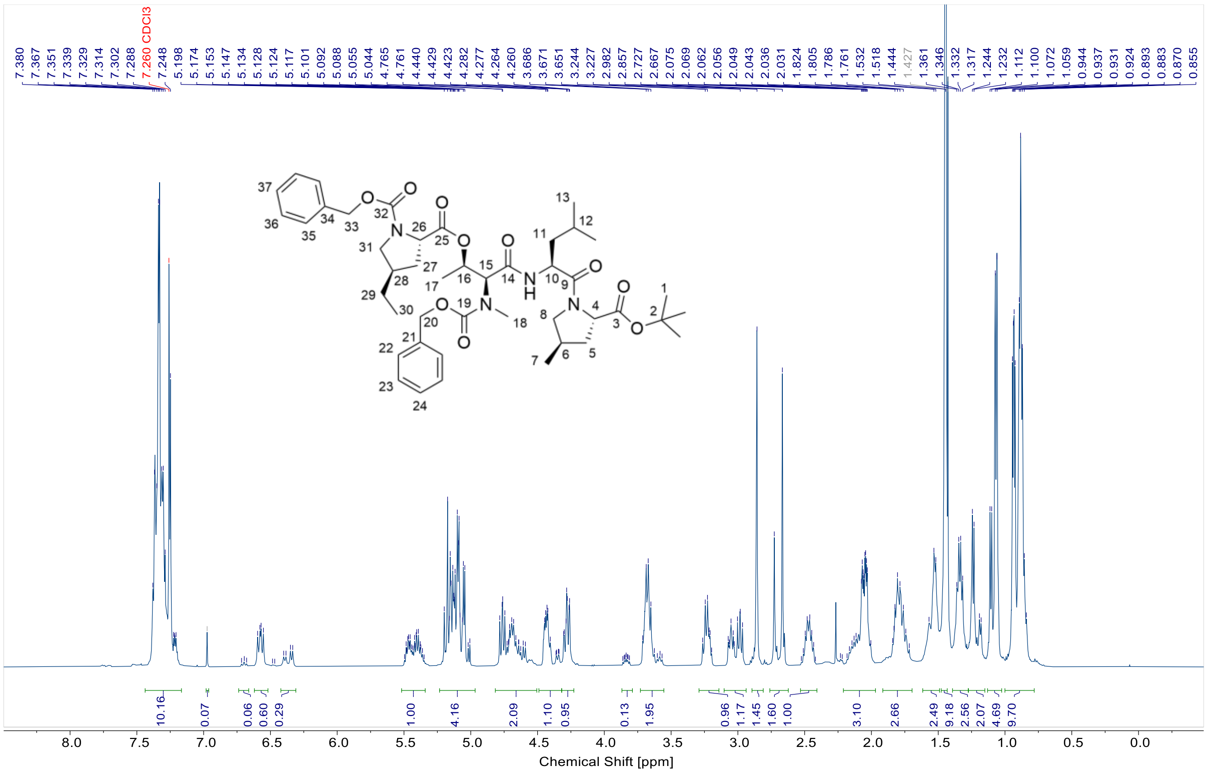

^{13}C-NMR (500 MHz, $CDCl_3$):

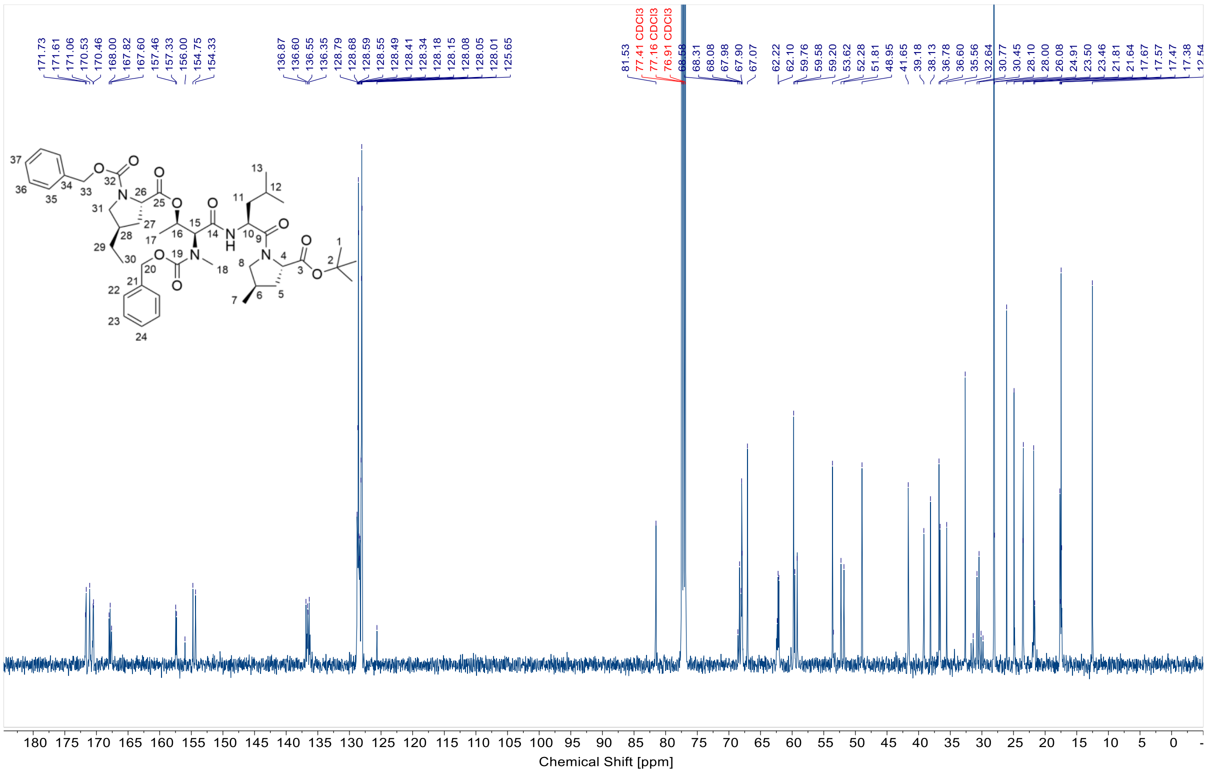

(^{1}H,^{1}H)-COSY ($CDCl_3$):

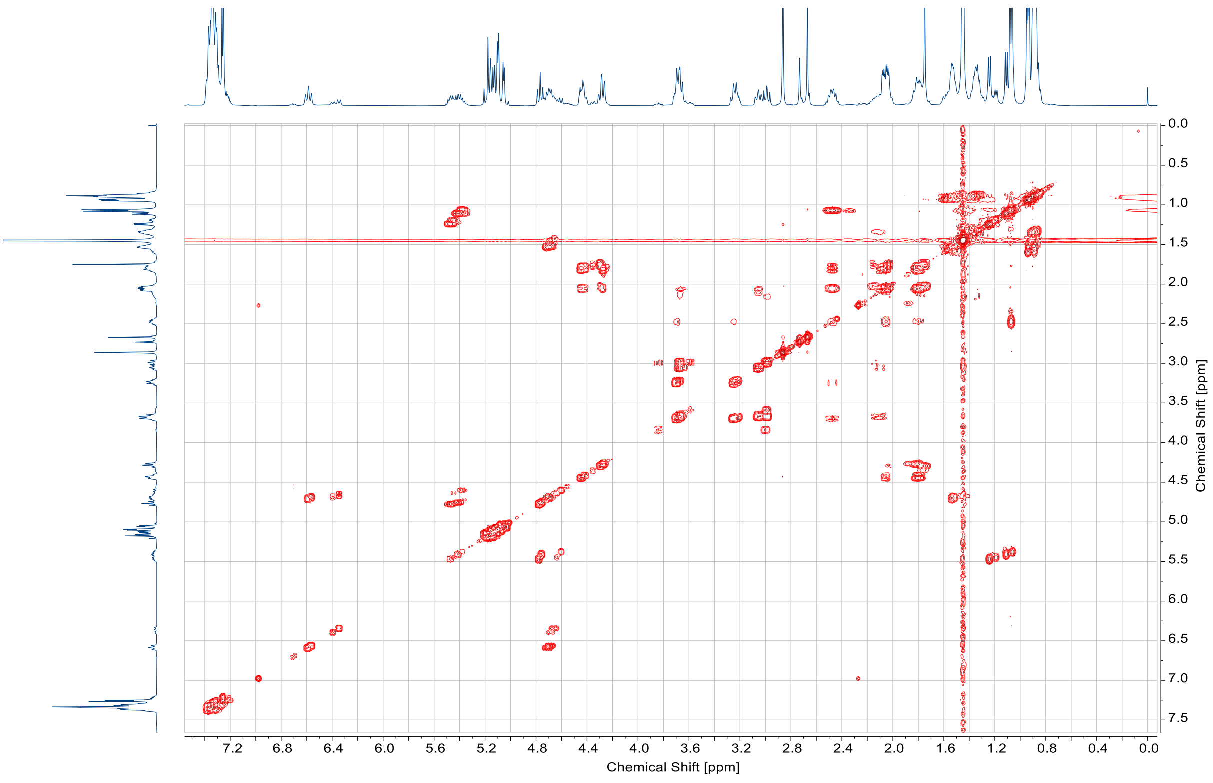

(^{1}H,^{13}C)-HSQC ($CDCl_3$):

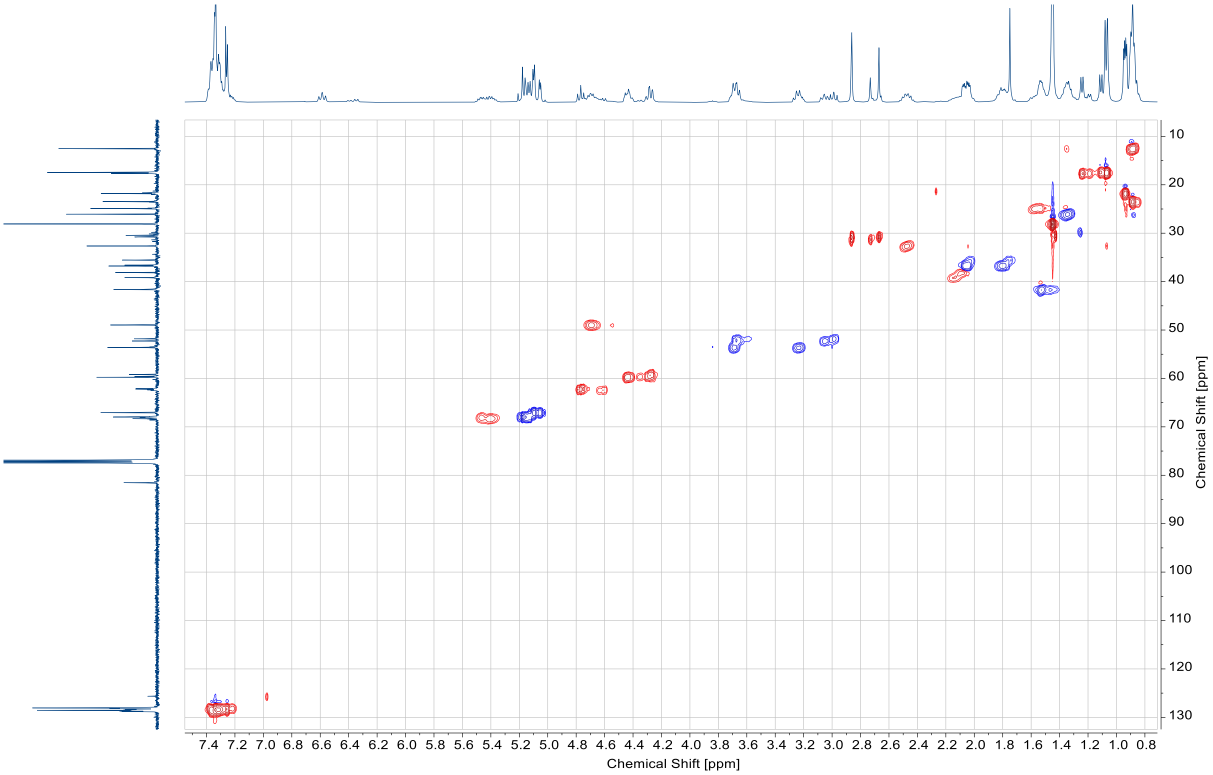

(^{1}H,^{13}C)-HMBC ($CDCl_3$)

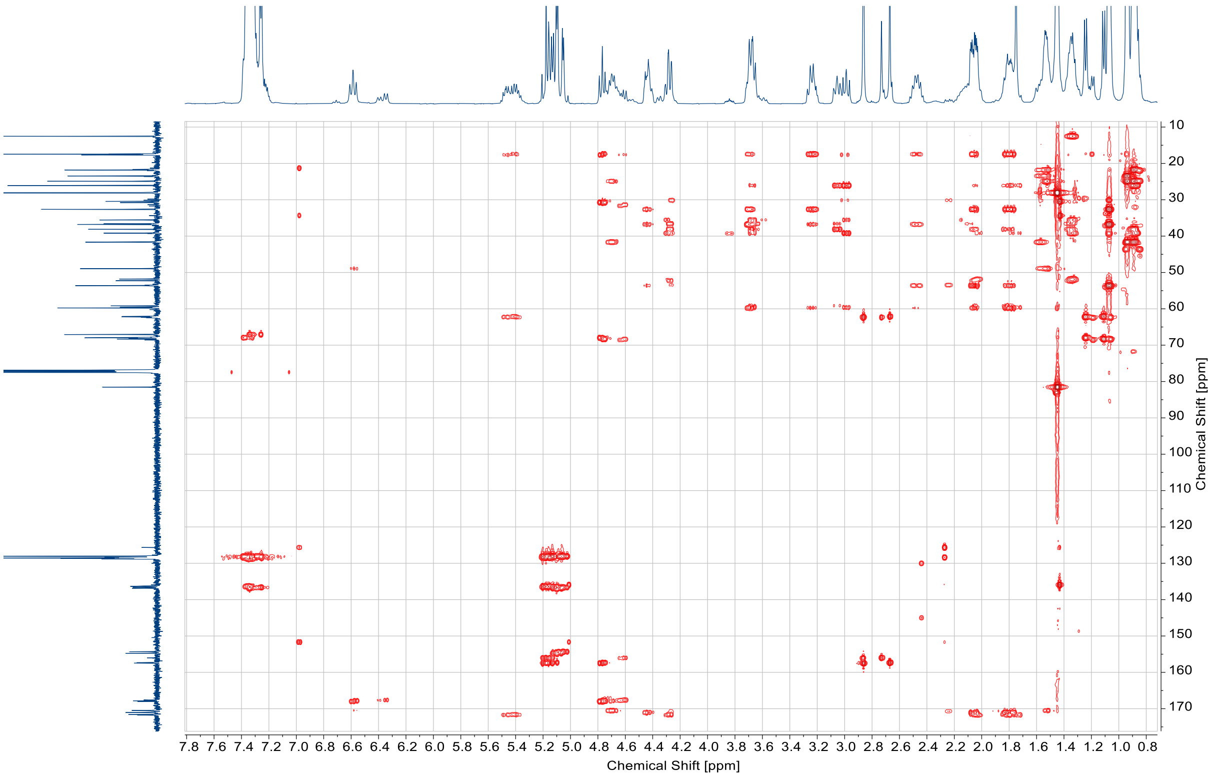

Alloc-*N*-Me-L-Val-L-EtPro-*N*-Me-L-Thr-L-Leu-L-MePro-O*t*-Bu [17]

^{1}H-NMR (500 MHz, DMSO-d_6, 373 K):

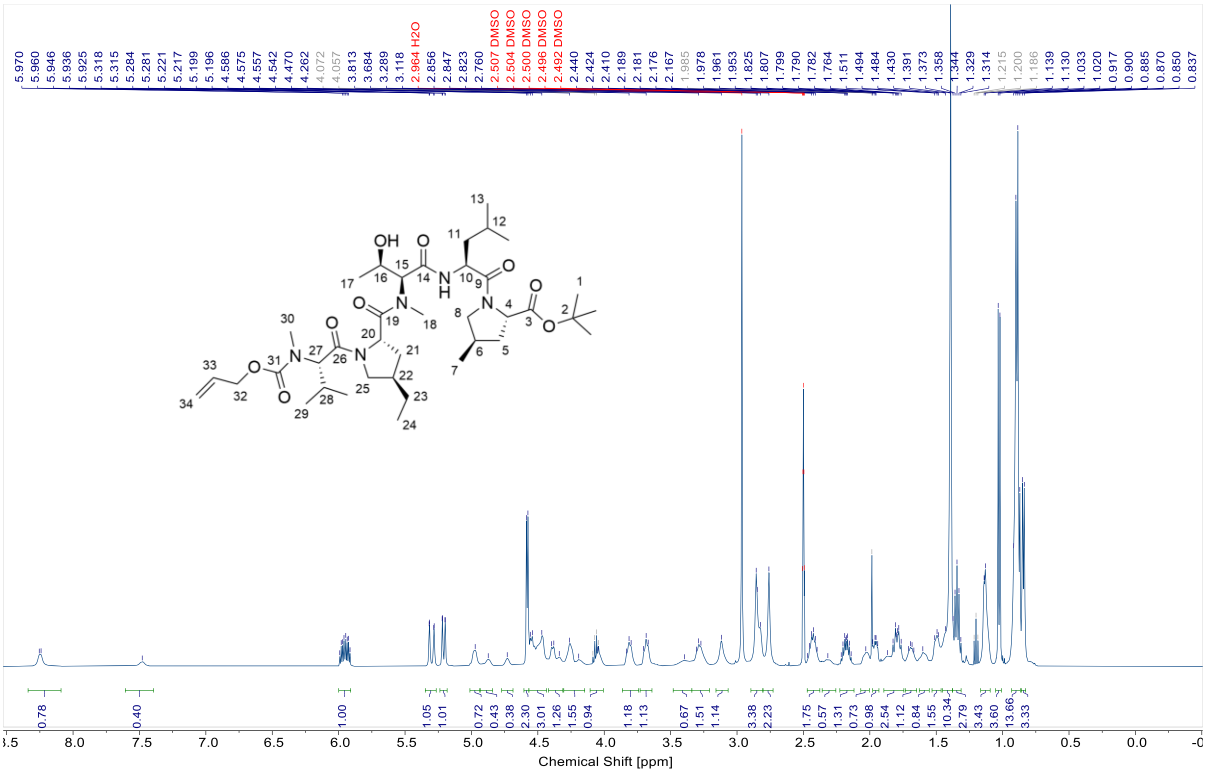

^{13}C-NMR (126 MHz, DMSO-d_6, 373 K):

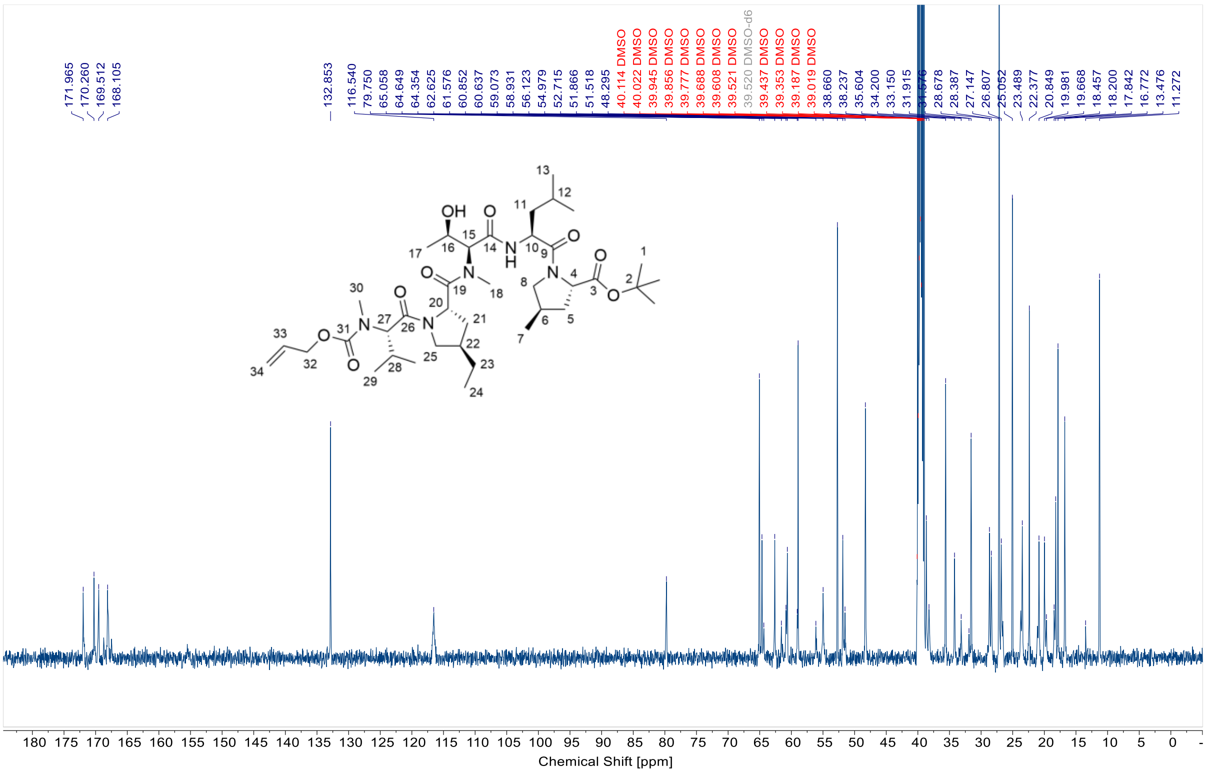

(^{1}H,^{1}H)-COSY (DMSO-d_6, 373 K):

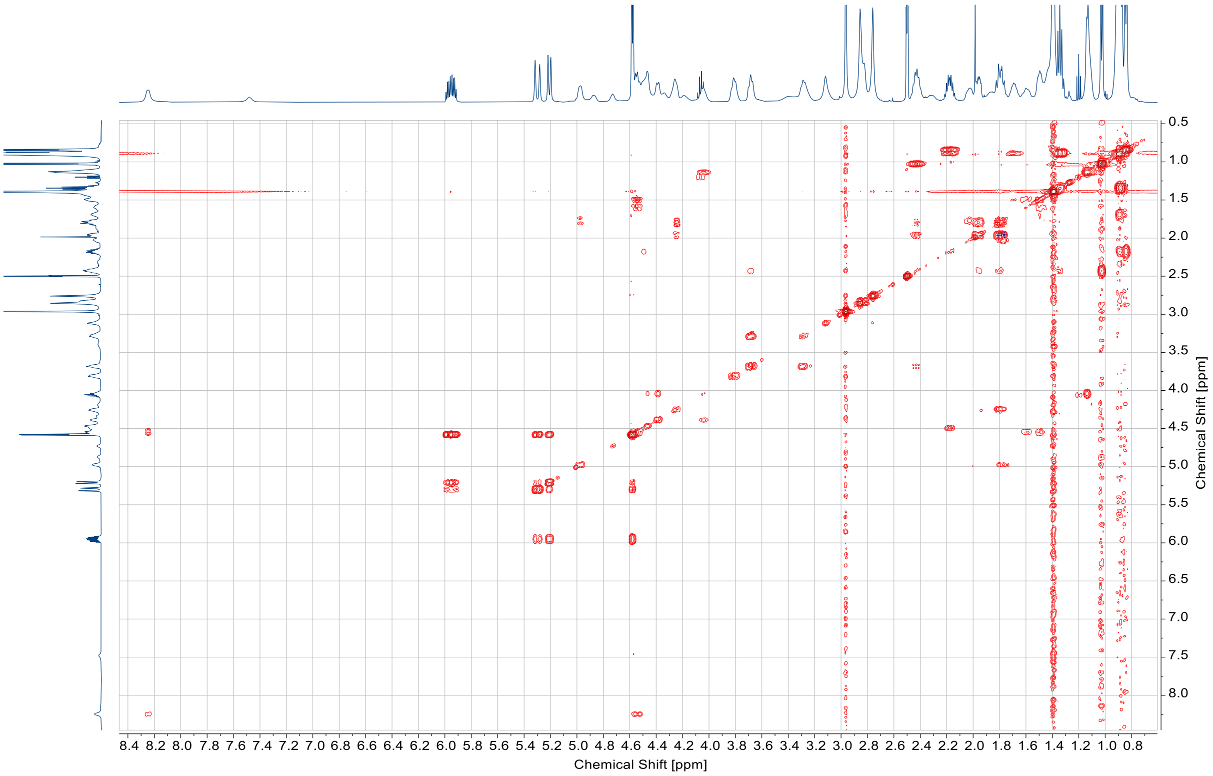

(^{1}H,^{13}C)-HSQC (DMSO-d_6, 373 K):

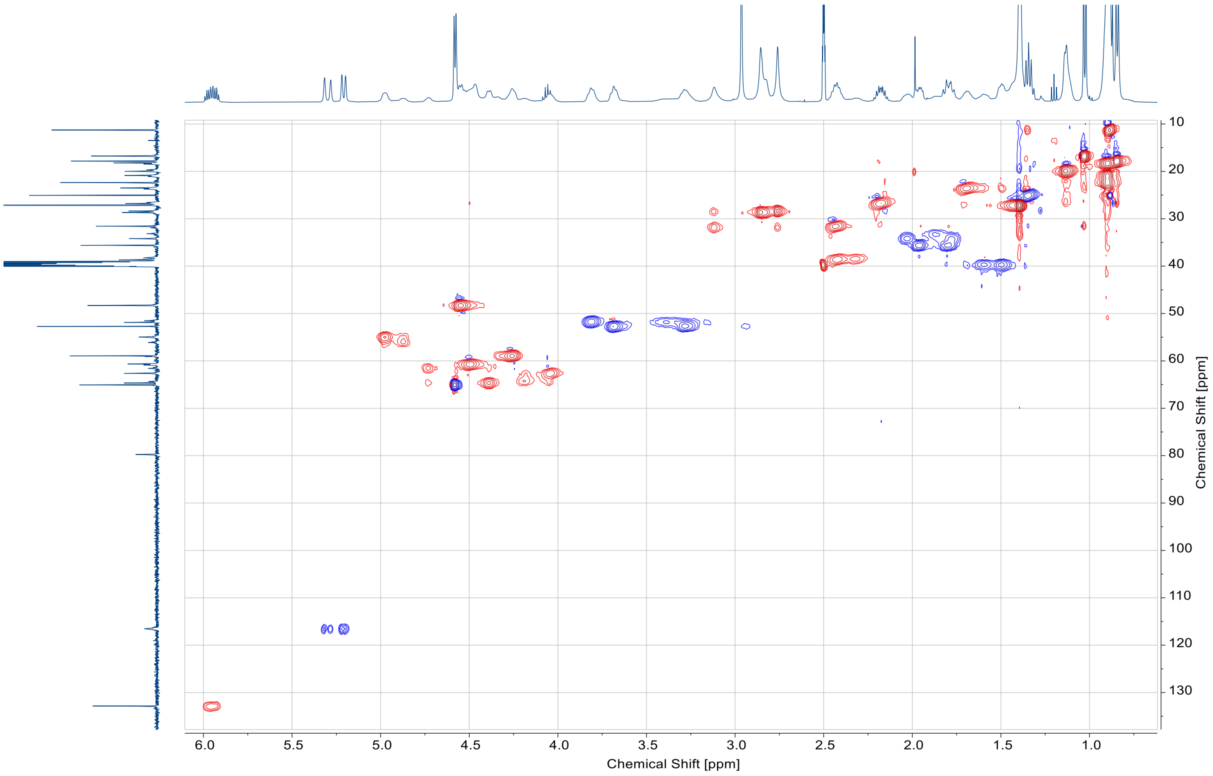

(^{1}H,^{13}C)-**HMBC** (DMSO-*d*$_6$, 373 K):

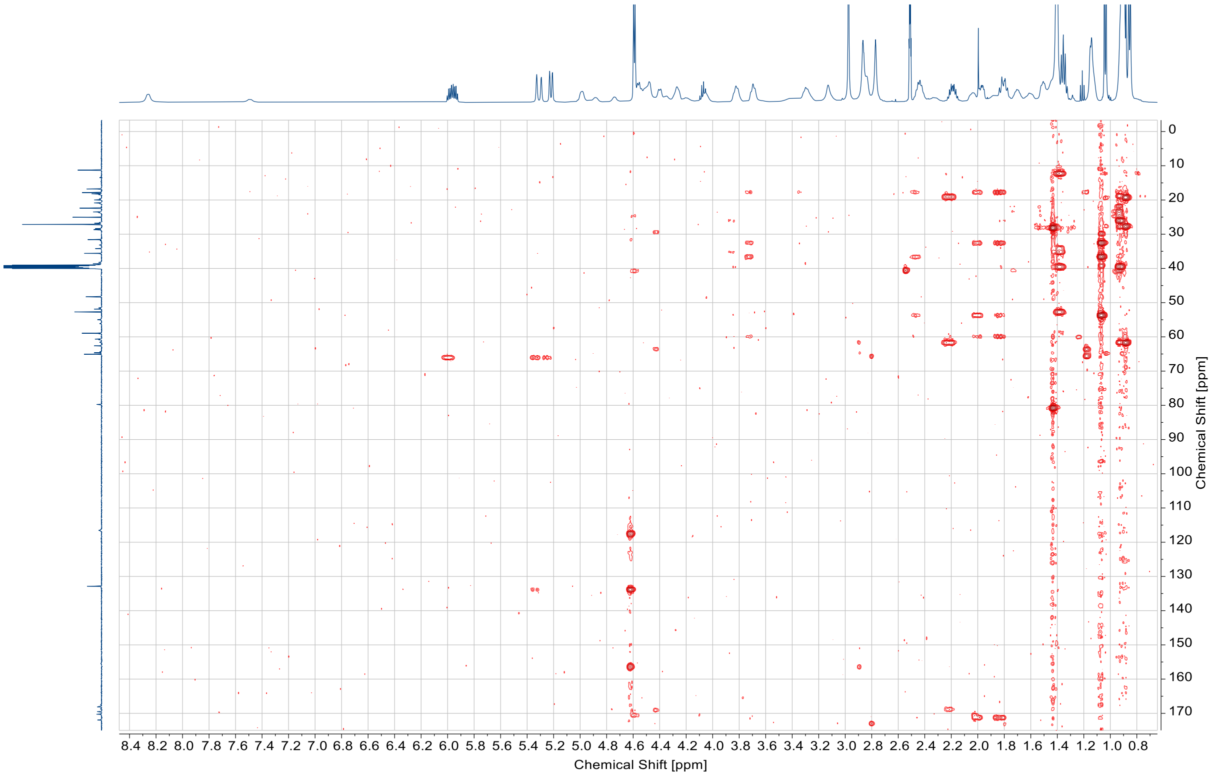

Alloc-*N*-Me-L-Val-L-EtPro-*O*-(Fmoc-Gly)-*N*-Me-L-Thr-L-Leu-L-MePro-O*t*-Bu [18]

^{1}**H-NMR** (500 MHz, $CDCl_3$):

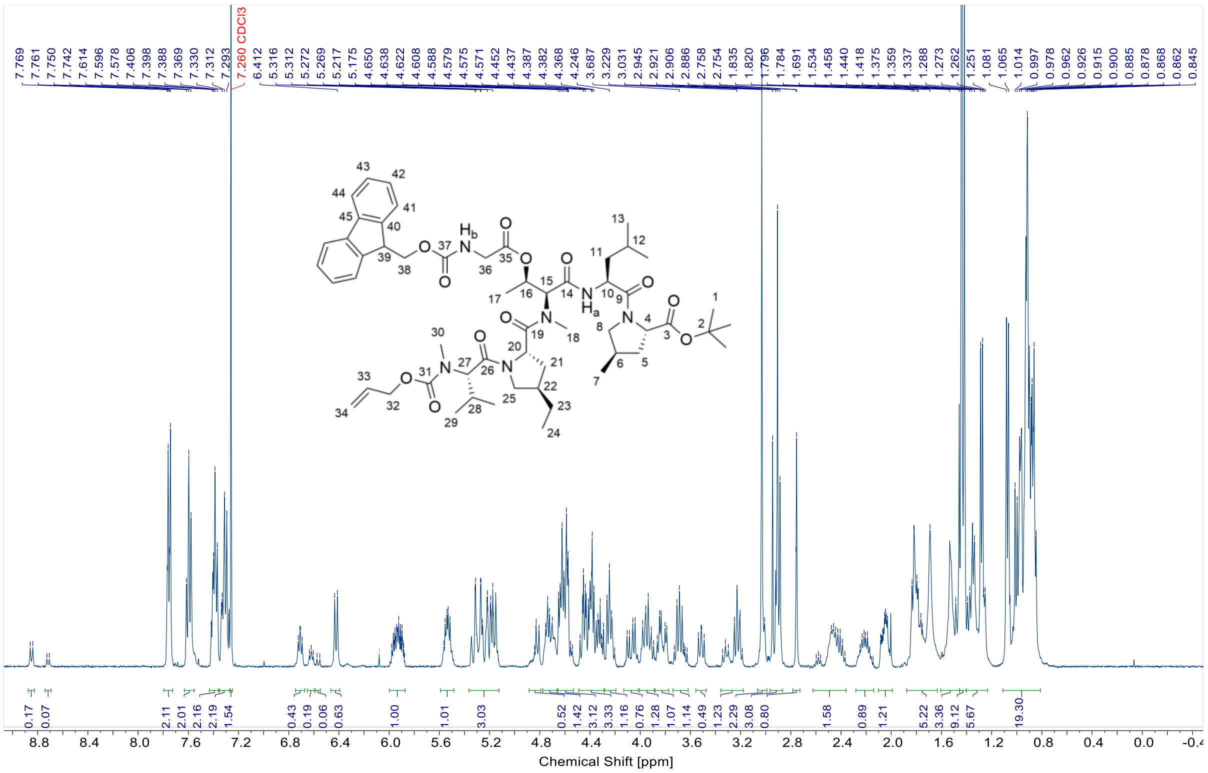

^{13}C-NMR (500 MHz, $CDCl_3$):

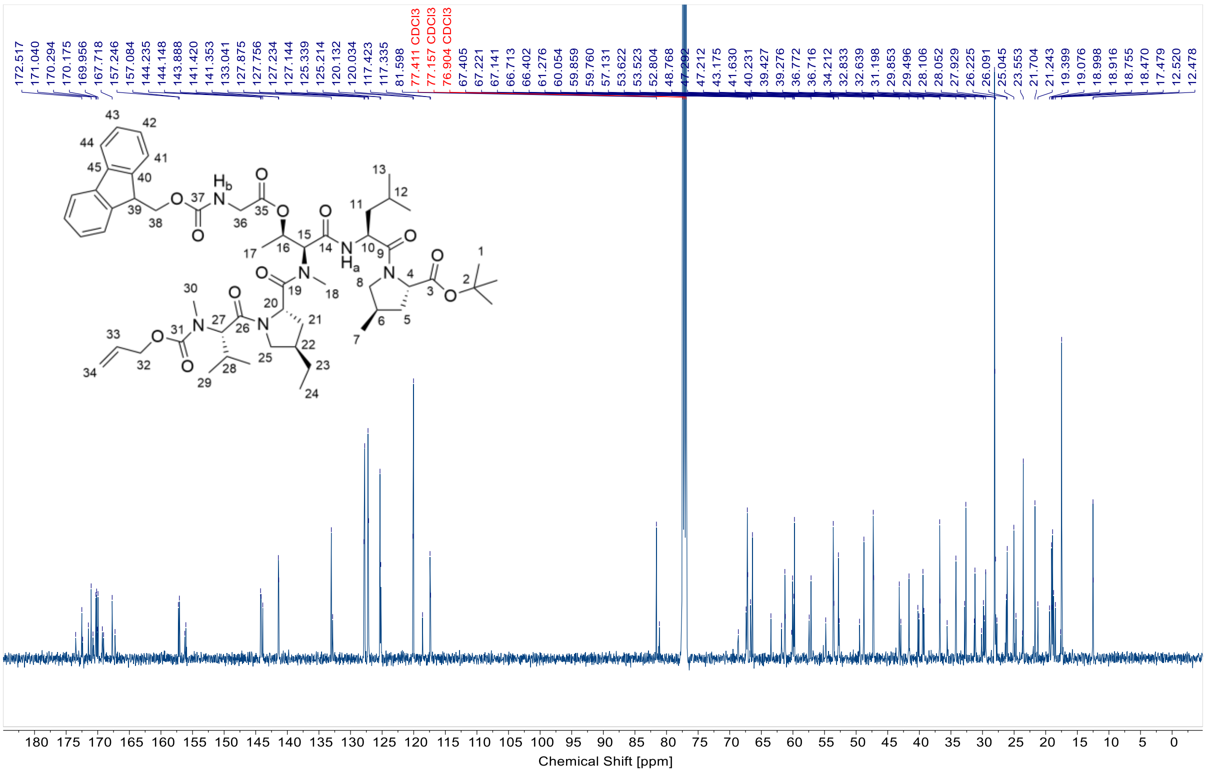

(^{1}H,^{1}H)-COSY ($CDCl_3$):

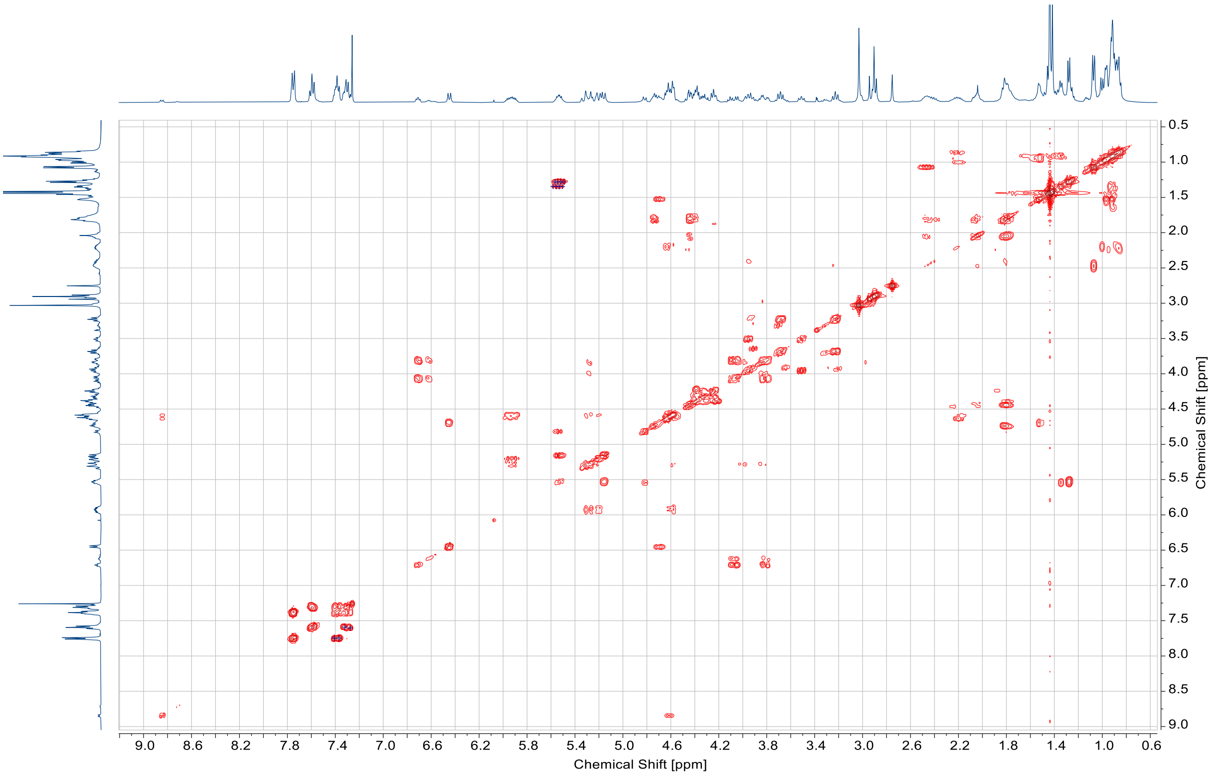

(^{1}H,^{13}C)-HSQC (CDCl$_3$):

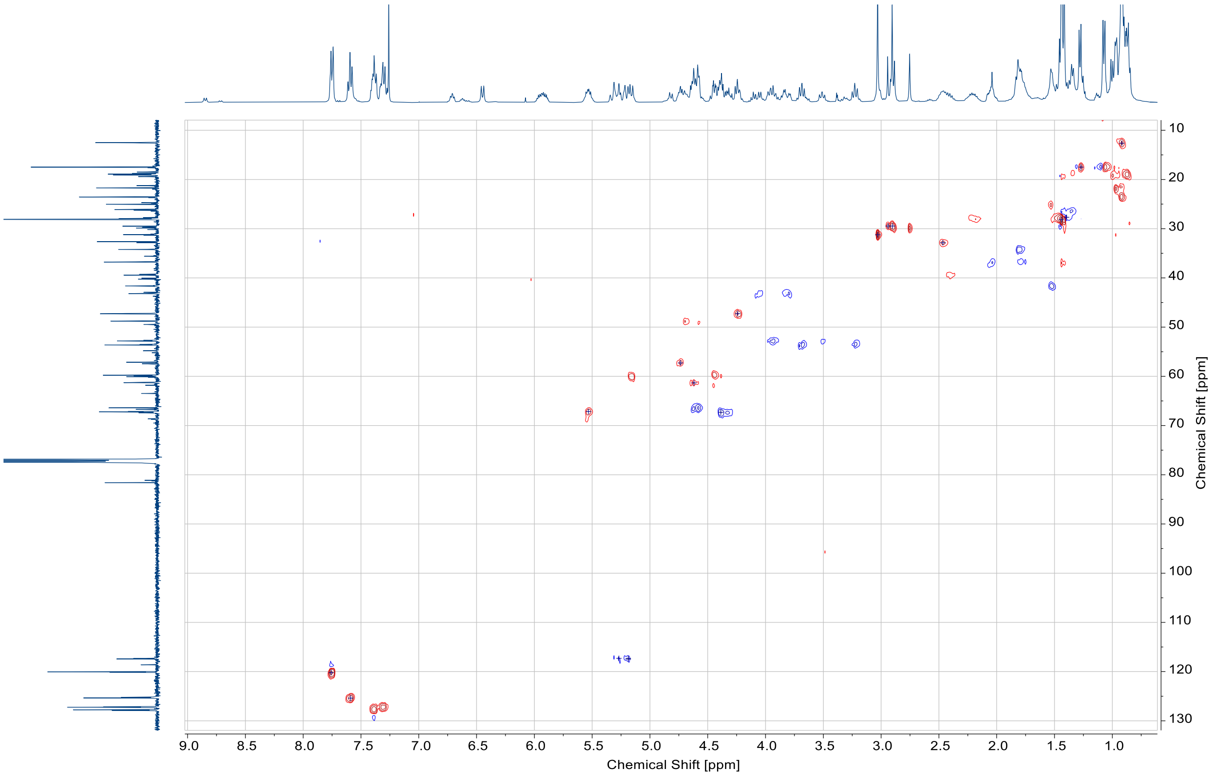

(^{1}H,^{13}C)-HMBC (CDCl$_3$):

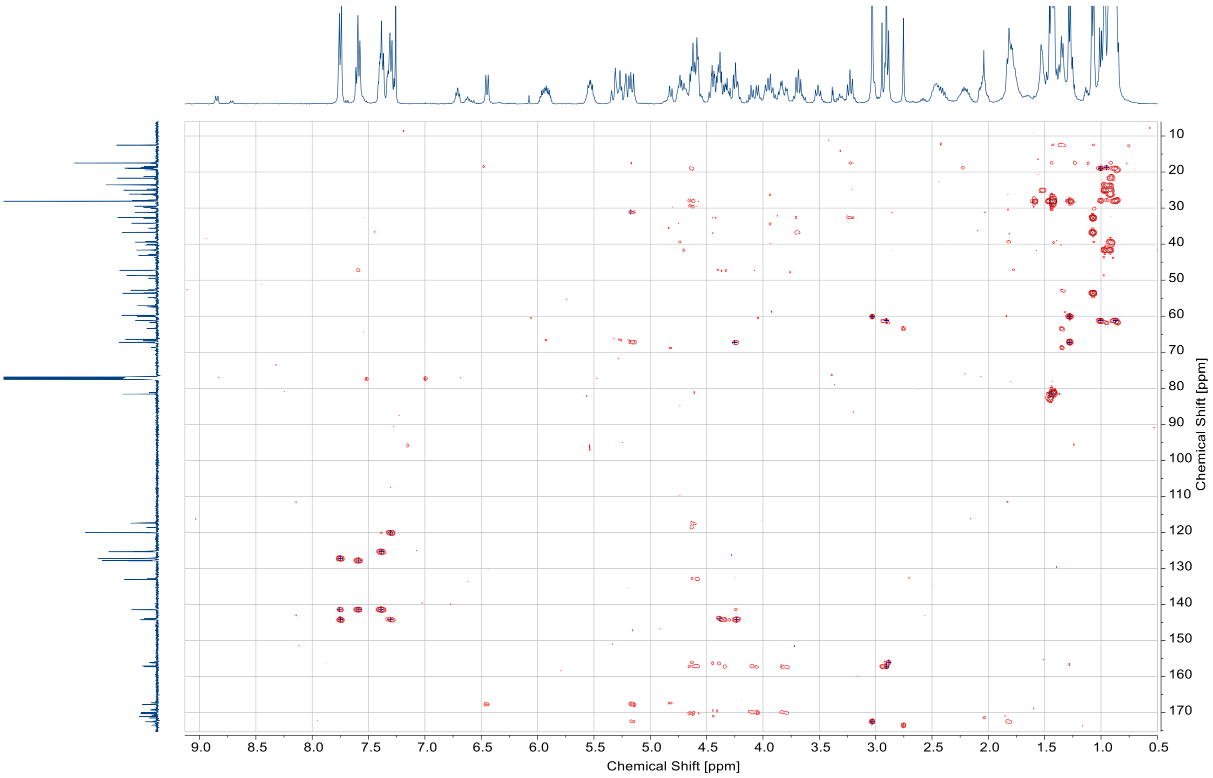

Boc-L-Pro-*N*-Me-D-Leu-OMe [20]

^{1}H-NMR (500 MHz, DMSO-d_6, 373 K):

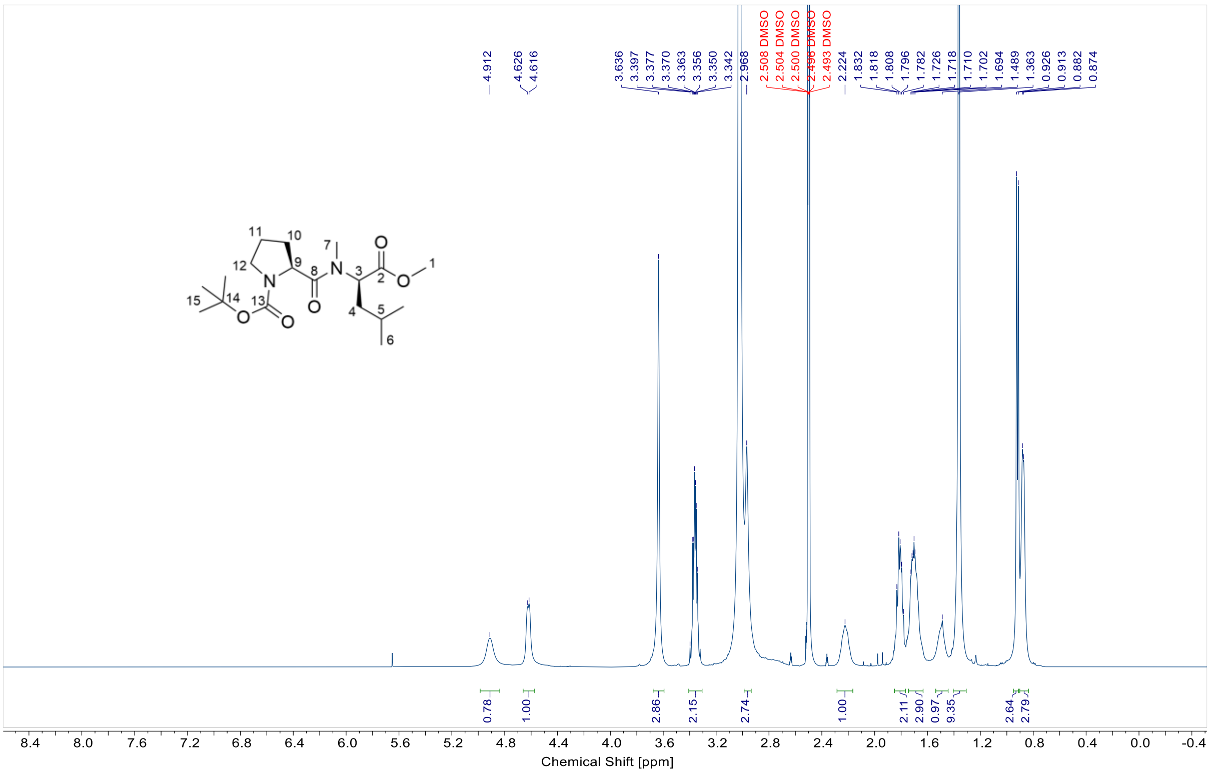

^{13}C-NMR (126 MHz, DMSO-d_6, 373 K):

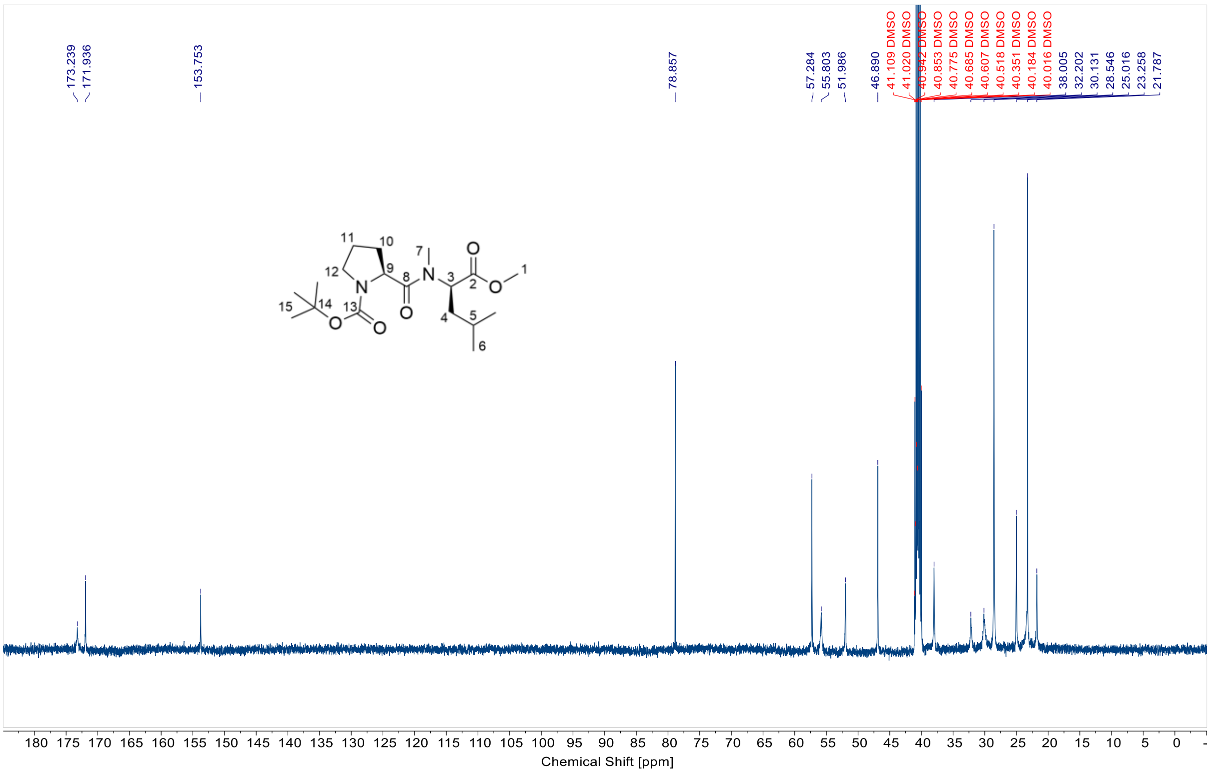

(^{1}H,^{1}H)-COSY (DMSO-d_6, 373 K):

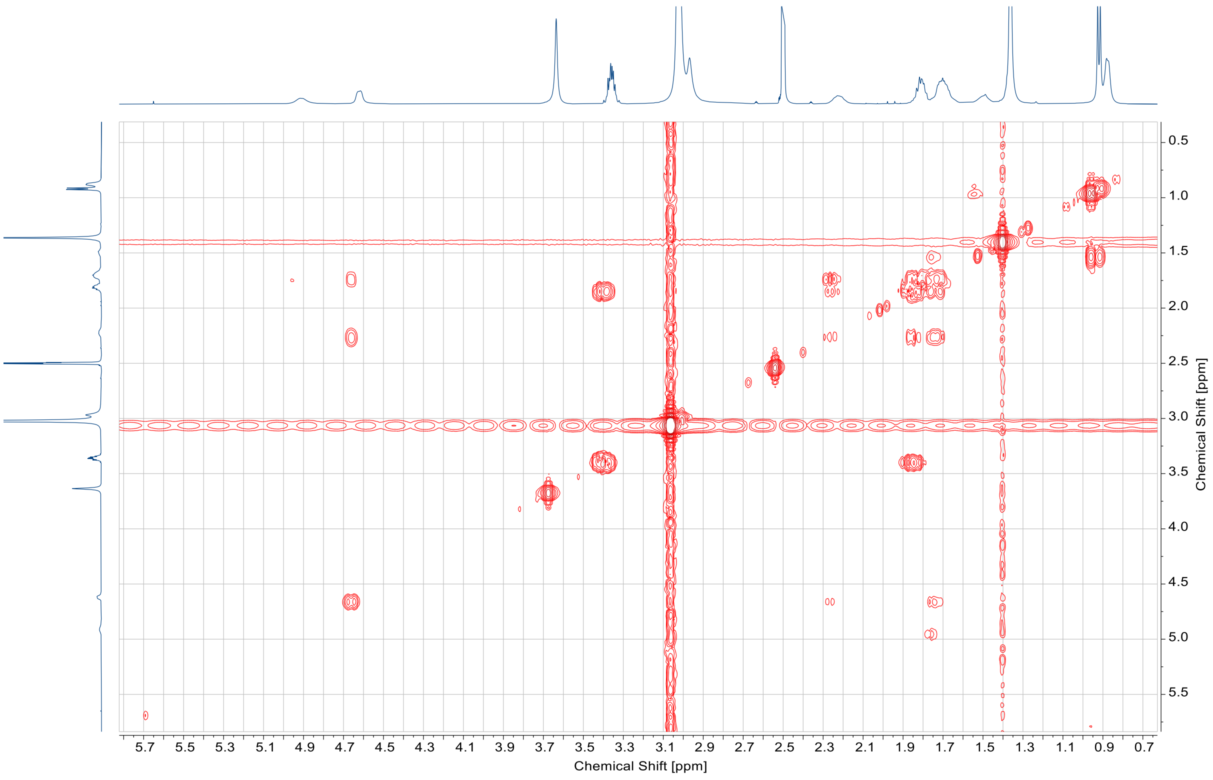

(^{1}H,^{13}C)-HSQC (DMSO-d_6, 373 K):

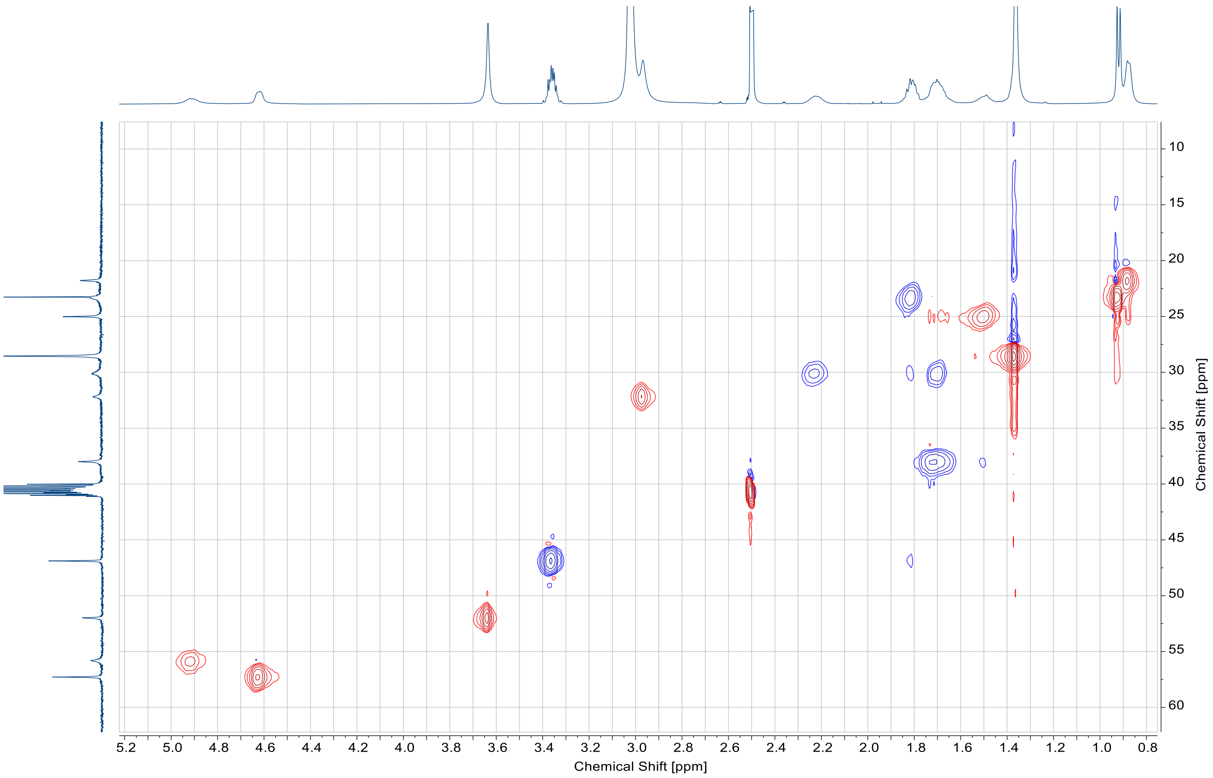

(^{1}H,^{13}C)-HMBC (DMSO-d_6, 373 K):

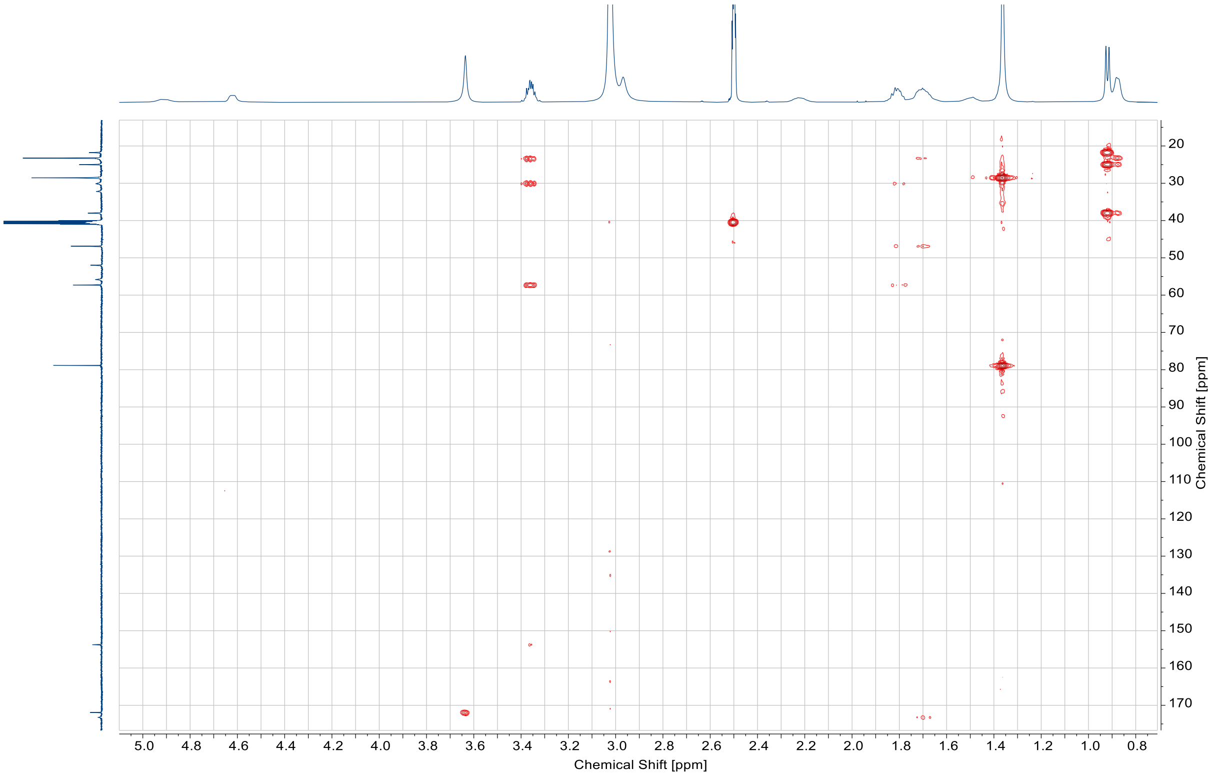

Boc-*N*-Me-L-Val-L-Pro-*N*-Me-D-Leu-OMe [21]

^{1}H-NMR (500 MHz, DMSO-d_6, 373 K):

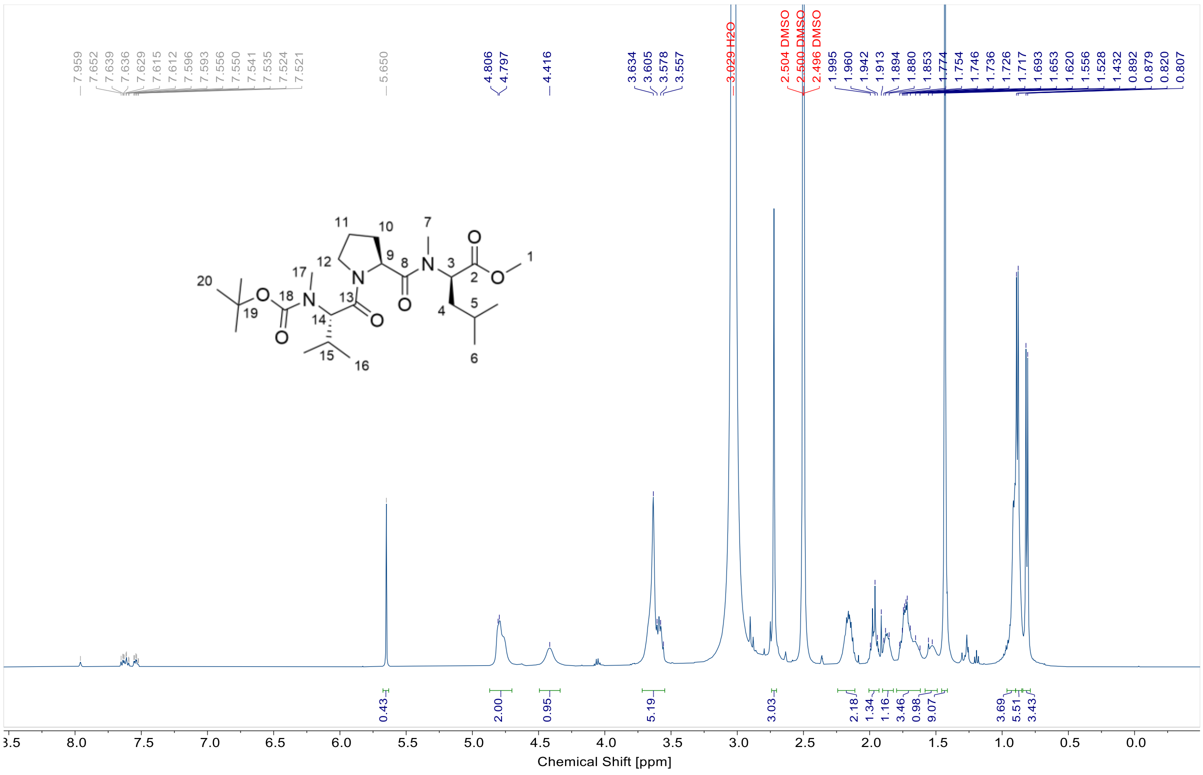

^{13}C-NMR (126 MHz, DMSO-d_6, 373 K):

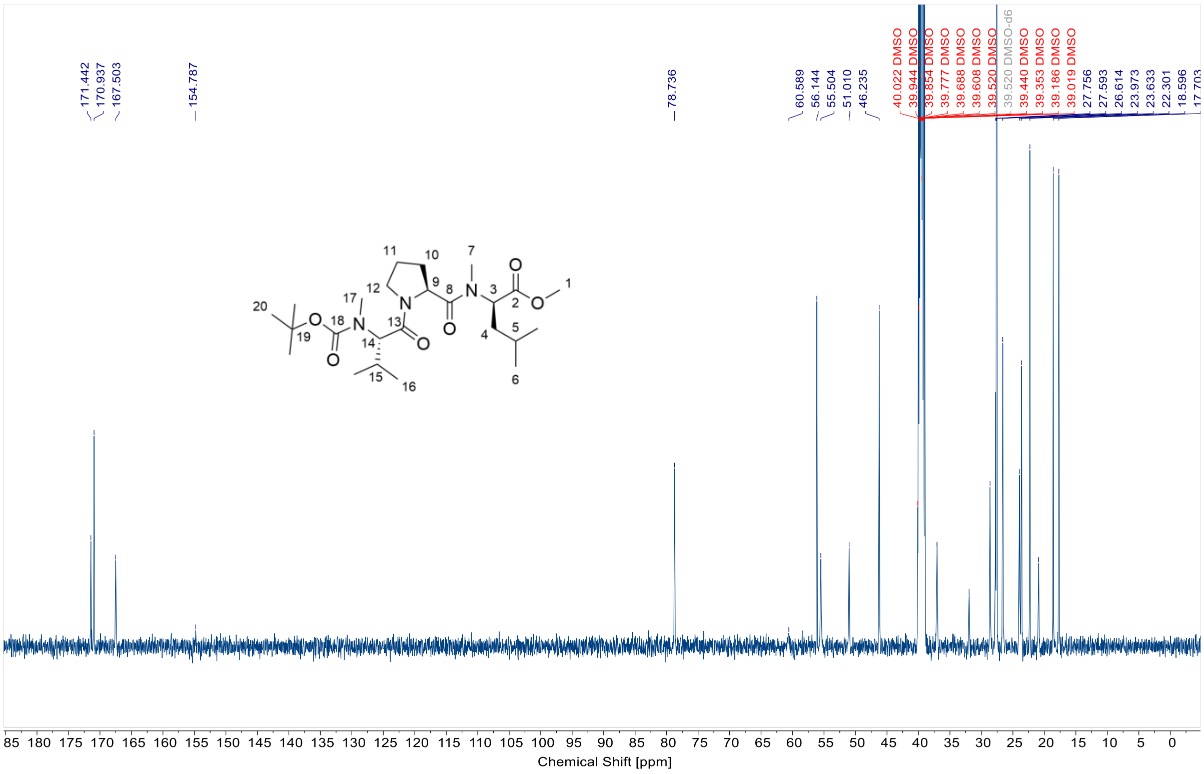

(^{1}H,^{1}H)-COSY (DMSO-d_6, 373 K):

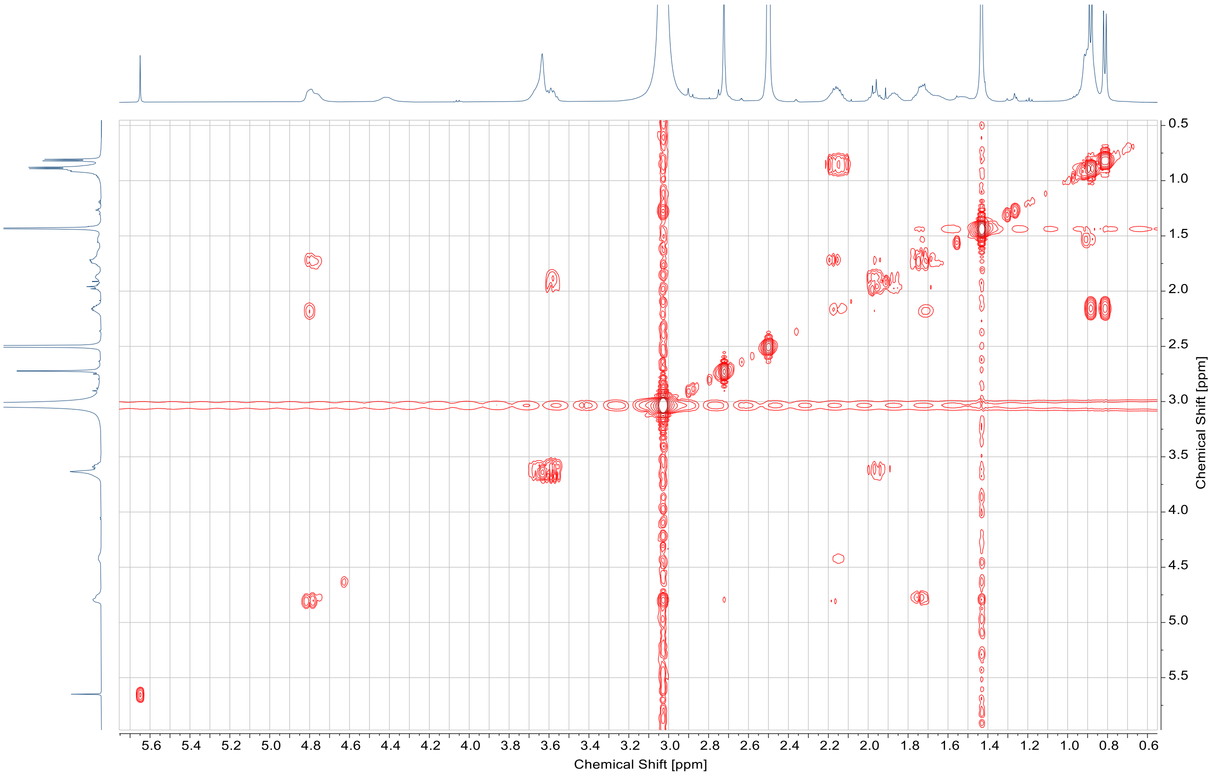

(^{1}H,^{13}C)-HSQC (DMSO-d_6, 373 K):

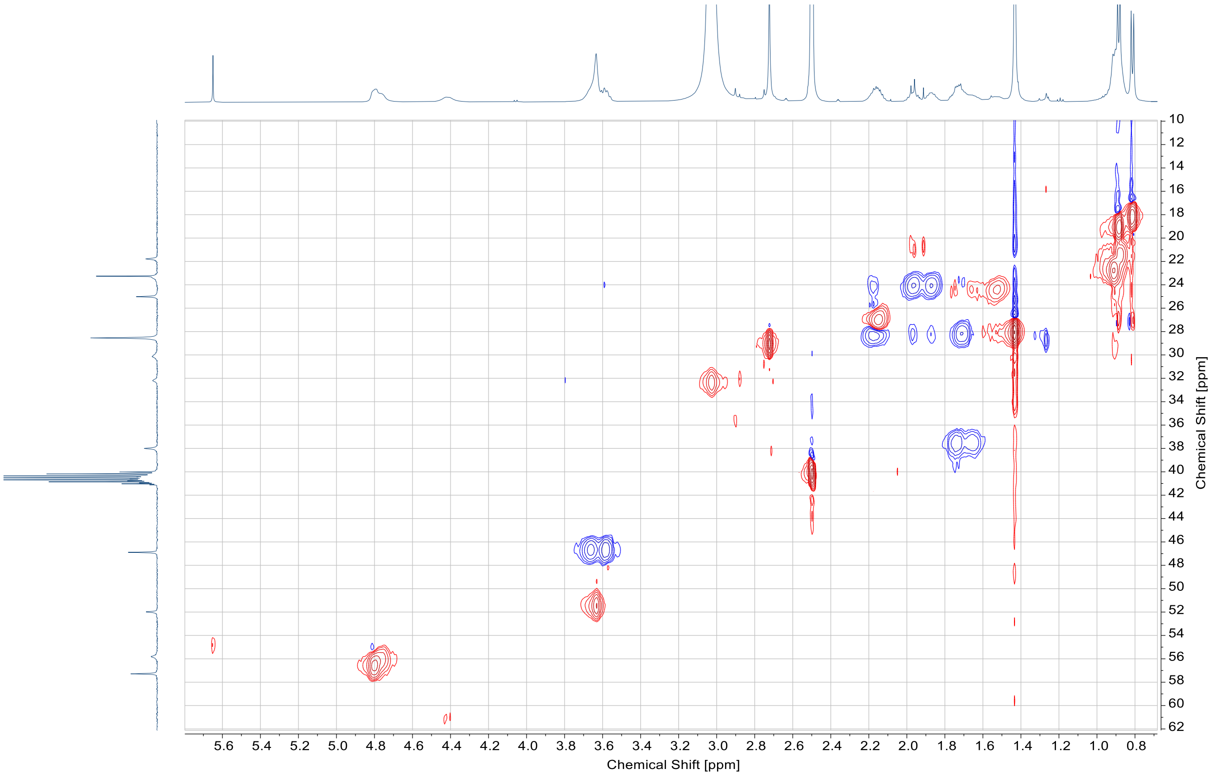

(^{1}H,^{13}C)-HMBC (DMSO-d_6, 373 K):

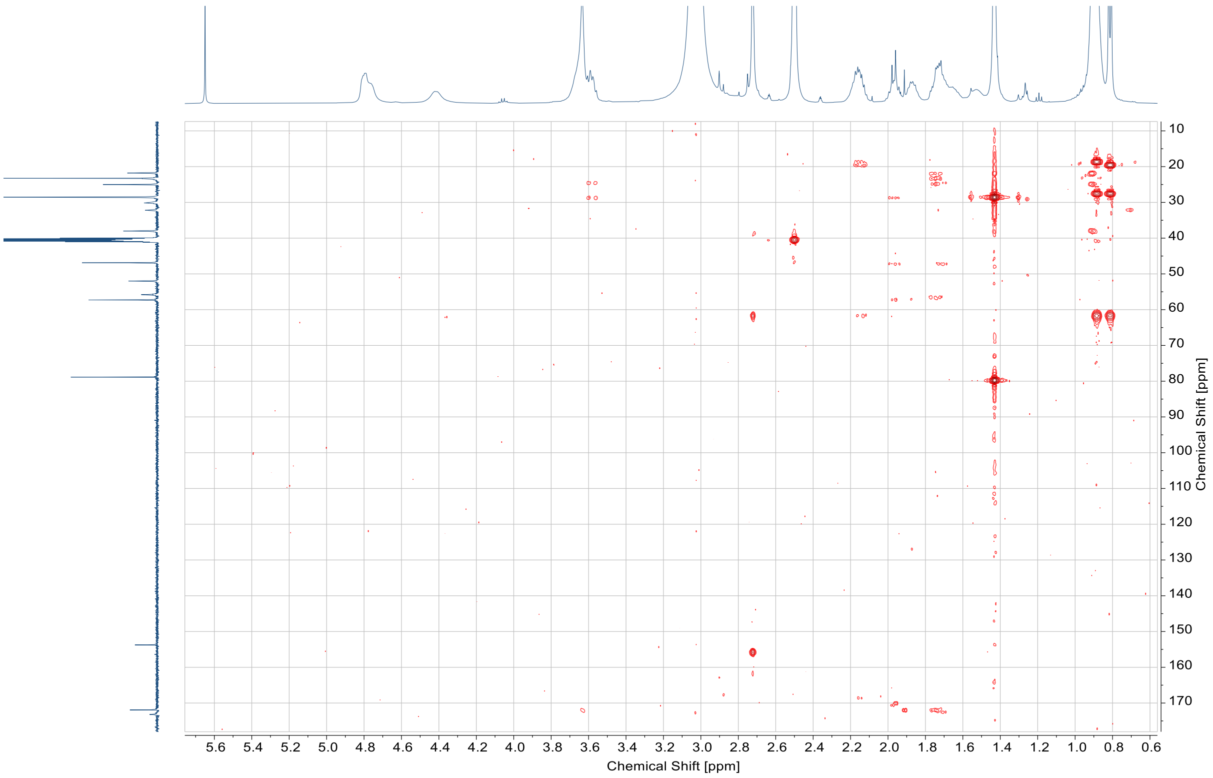

Boc-L-HoLeu-*N*-Me-L-Val-L-Pro-*N*-Me-D-Leu-OMe [22']

^{1}H-NMR (500 MHz, DMSO-d_6, 373 K):

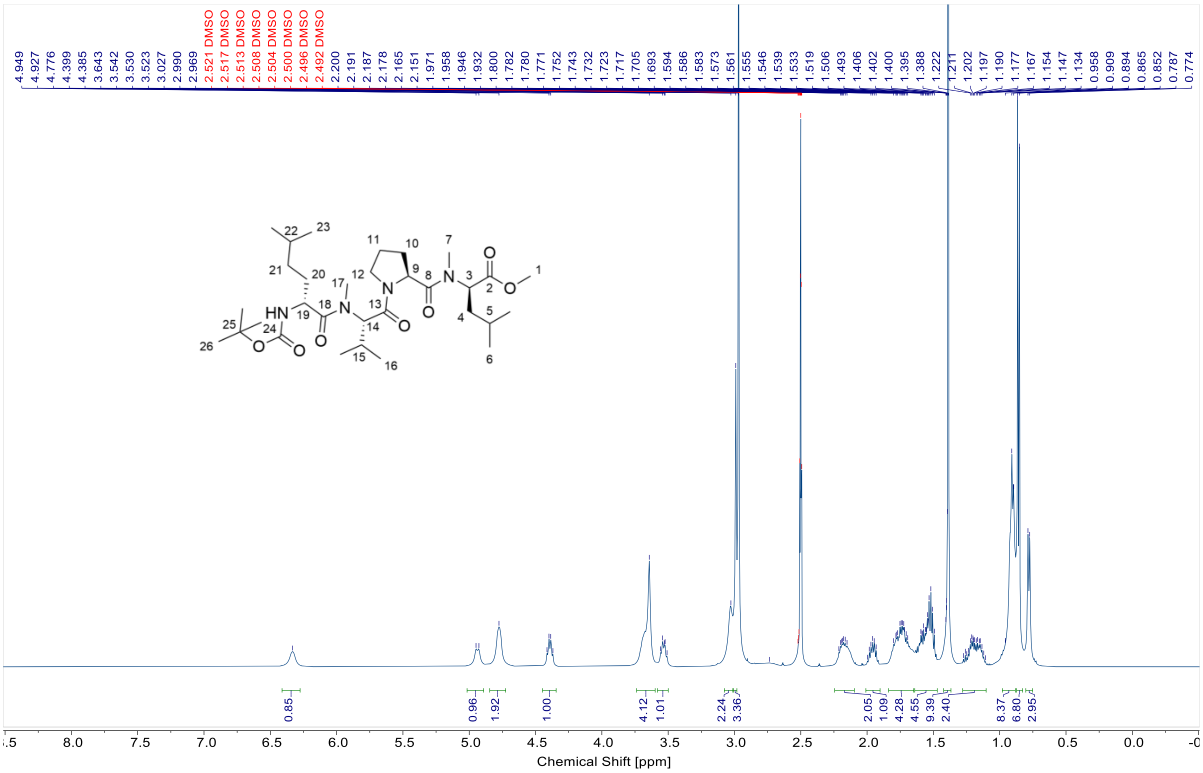

^{13}C-NMR (126 MHz, DMSO-d_6, 373 K):

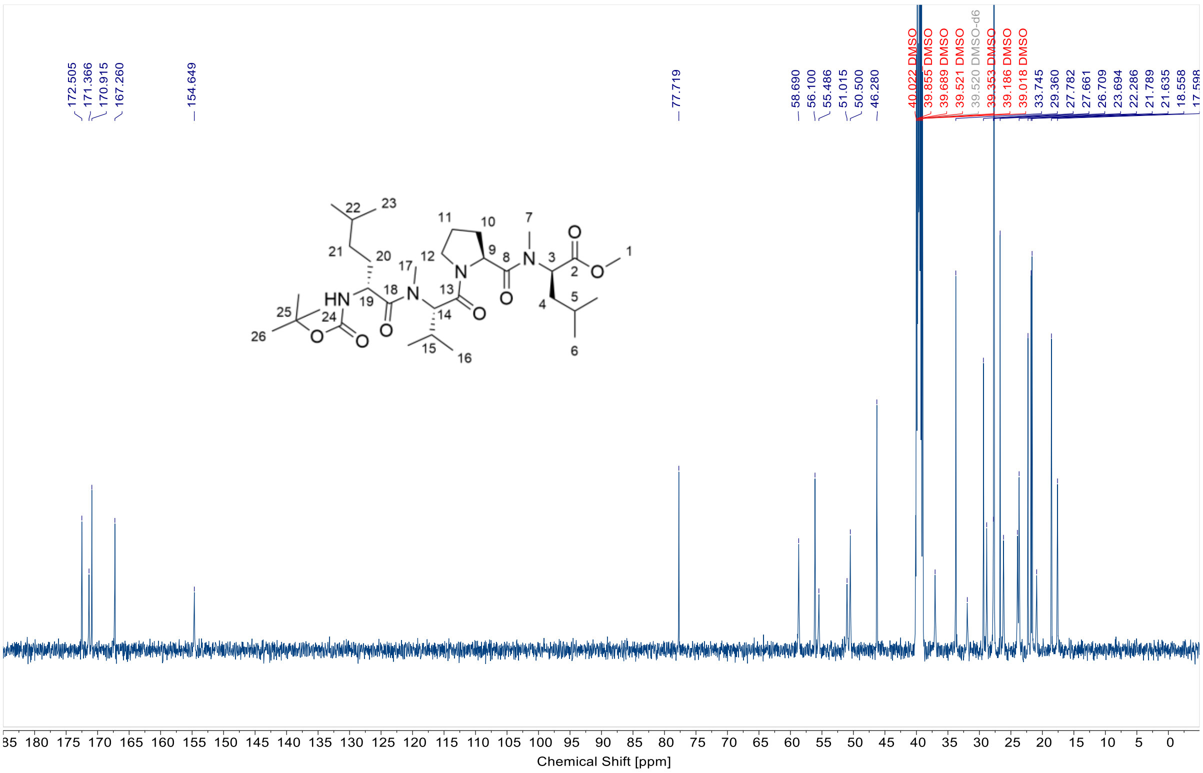

(^{1}H,^{1}H)-COSY (DMSO-d_6, 373 K):

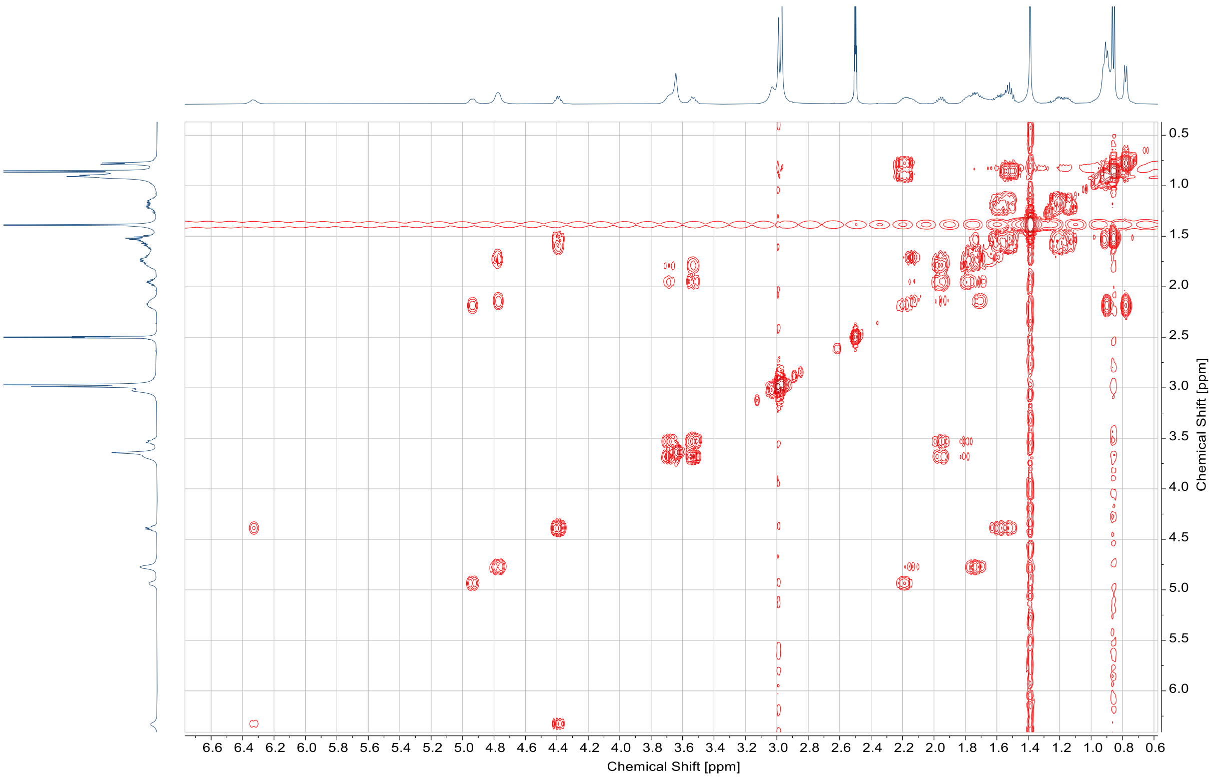

(^{1}H,^{13}C)-HSQC (DMSO-d_6, 373 K):

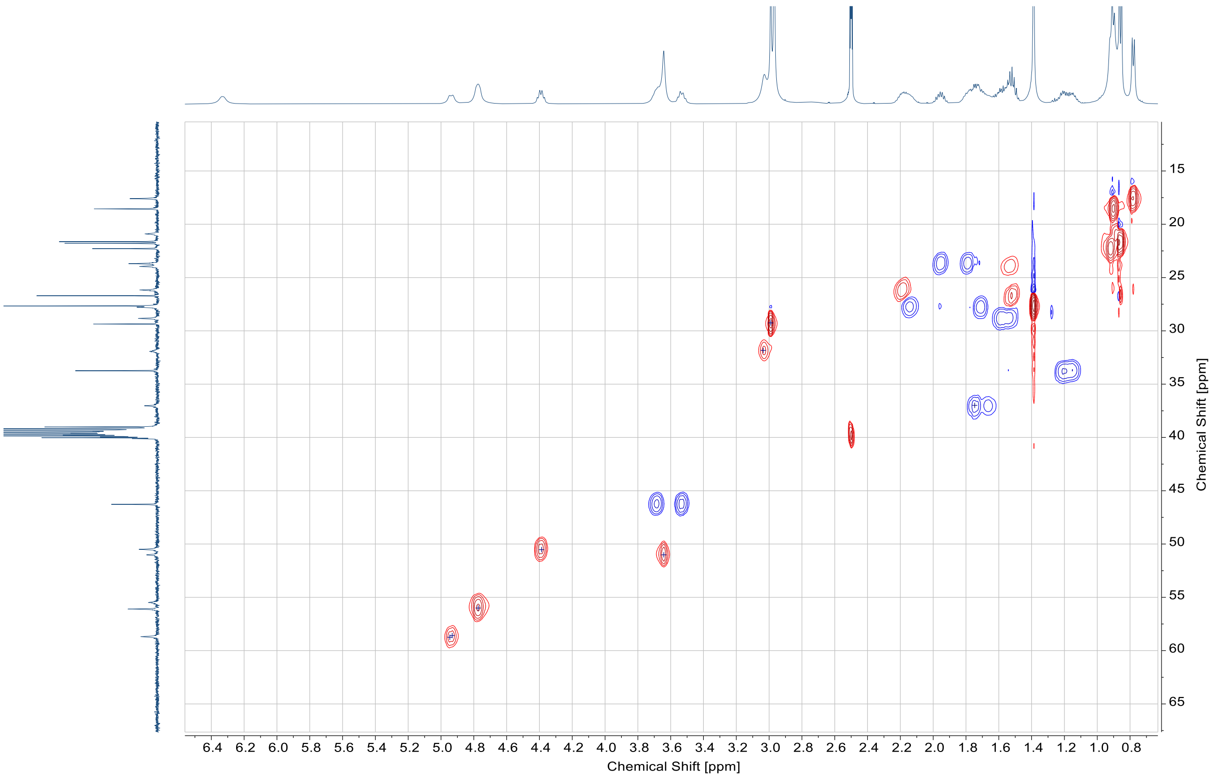

(^{1}H,^{13}C)-HMBC (DMSO-d_6, 373 K):

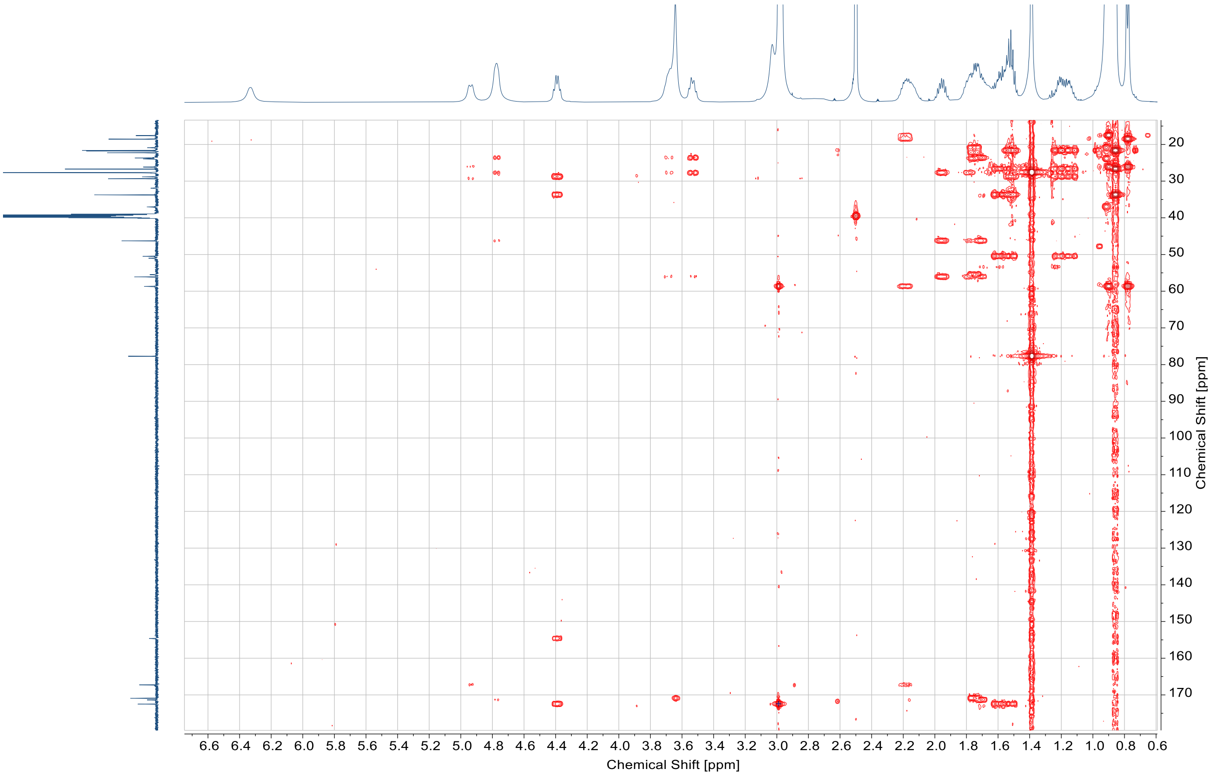

Boc-L-HoLeu-*N*-Me-L-Val-L-Pro-*N*-Me-D-Leu-OH [22]

^{1}H-NMR (400 MHz, DMSO-d_6):

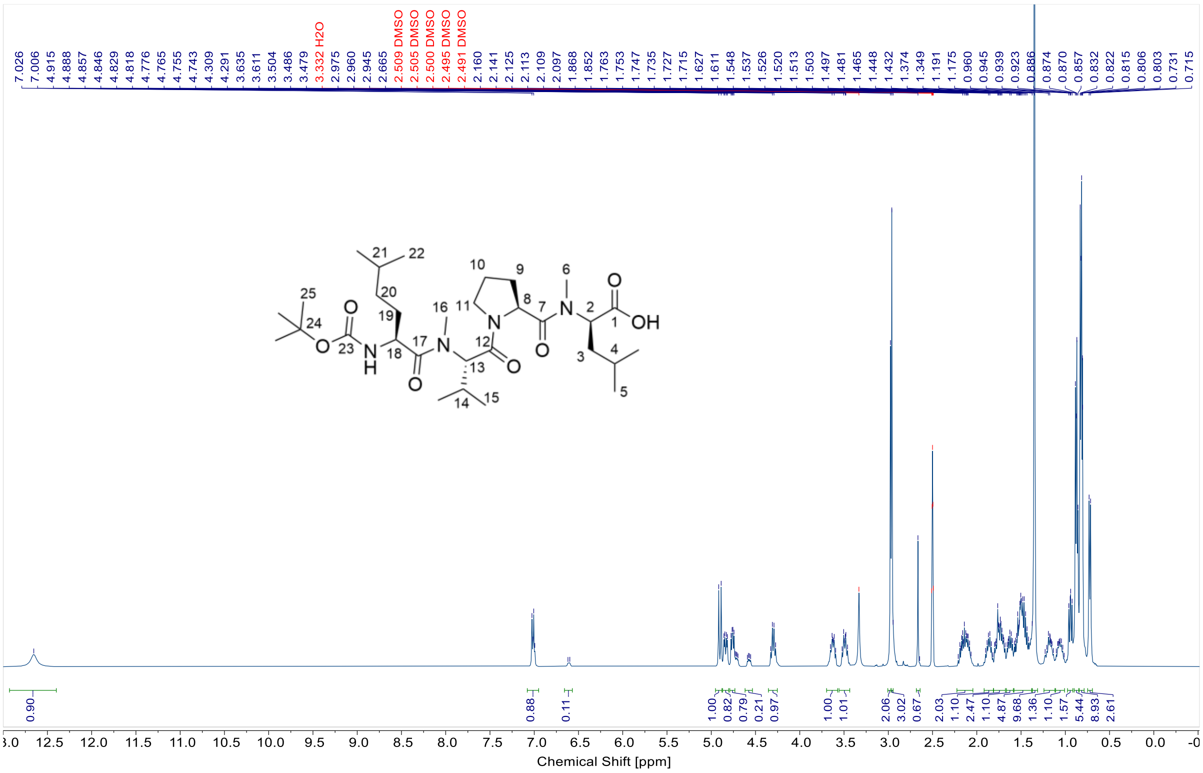

^{13}C-NMR (101 MHz, DMSO-d_6):

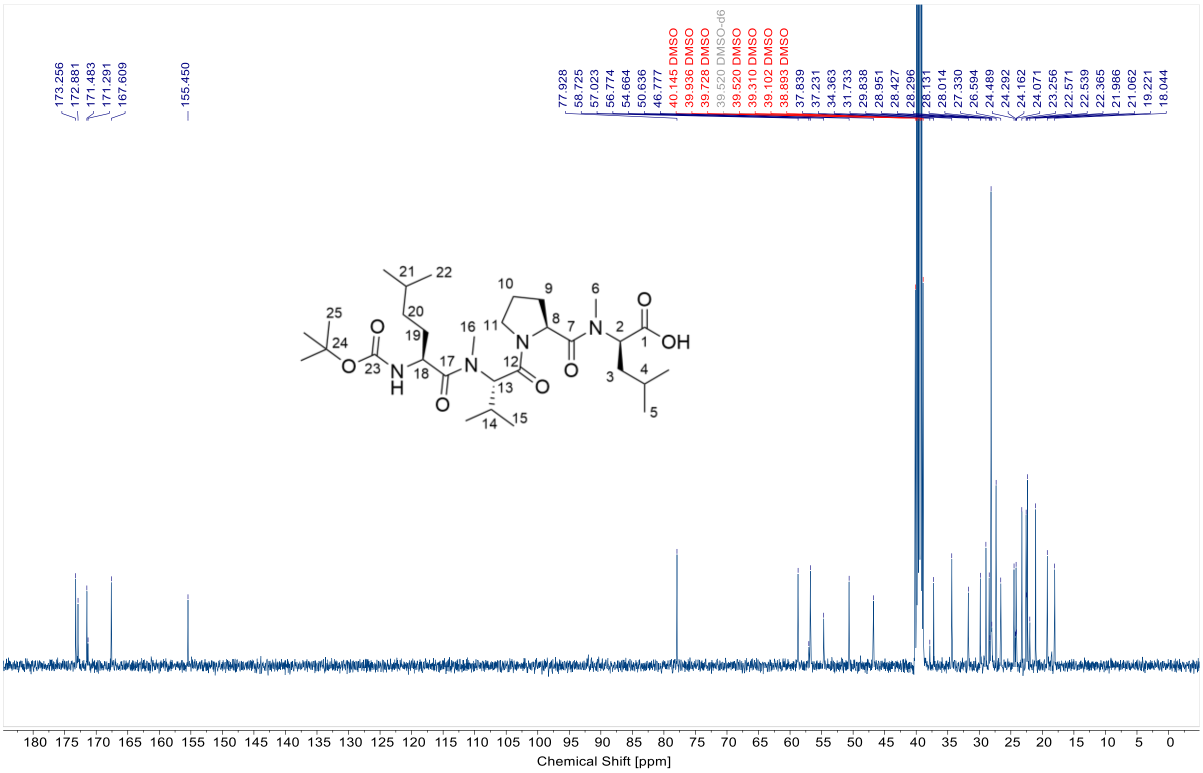

(^{1}H,^{1}H)-COSY (DMSO-d_6):

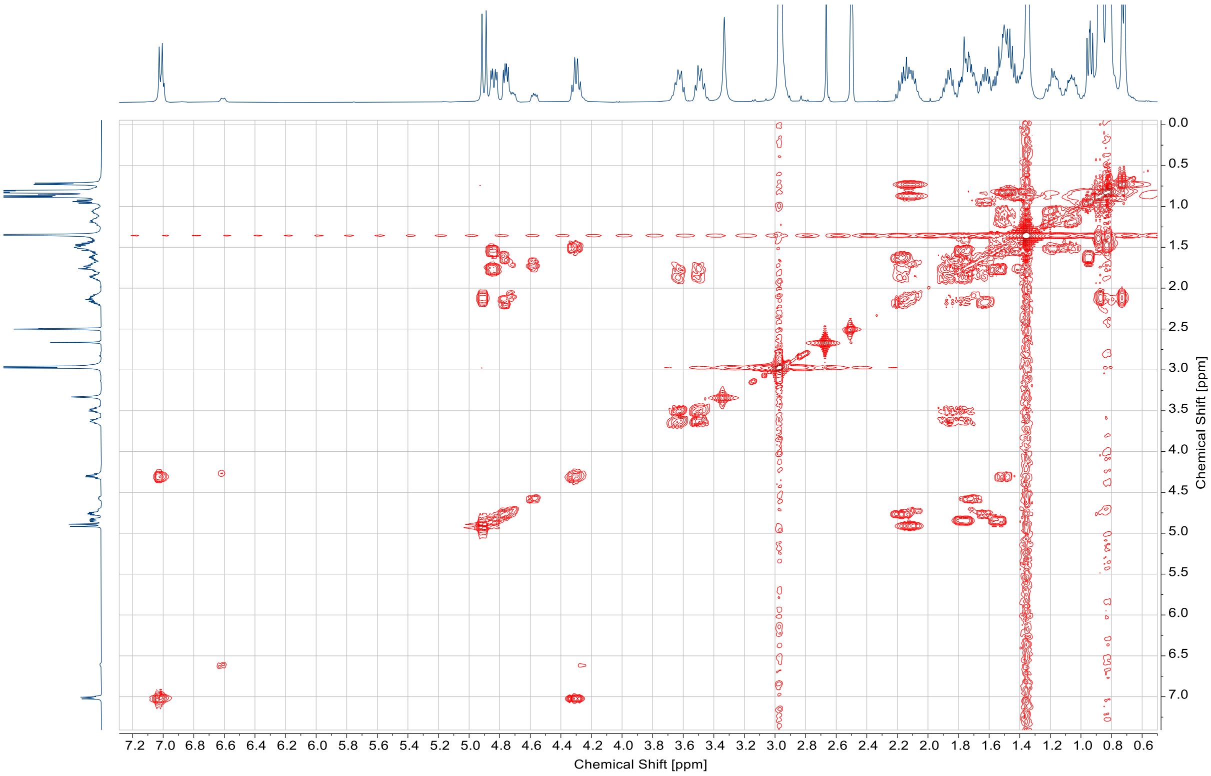

(^{1}H,^{13}C)-HSQC (DMSO-d_6):

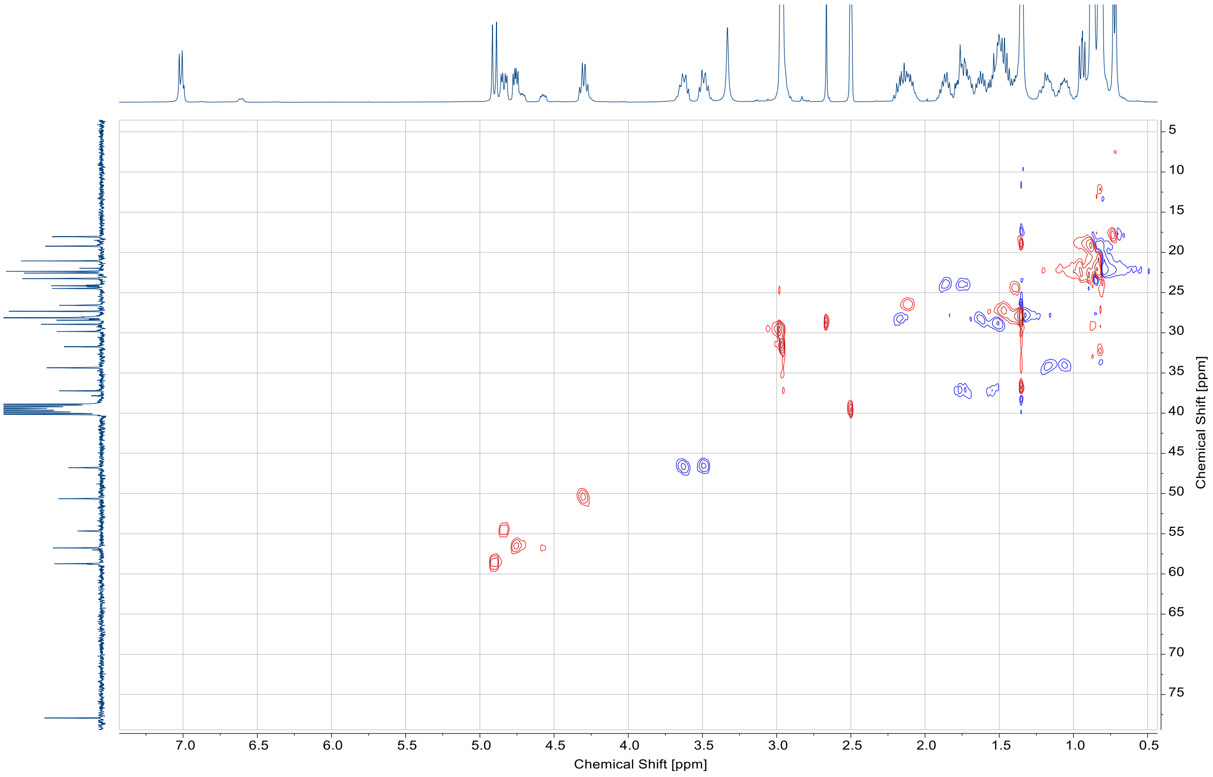

(^{1}H,^{13}C)-HMBC (DMSO-d_6):

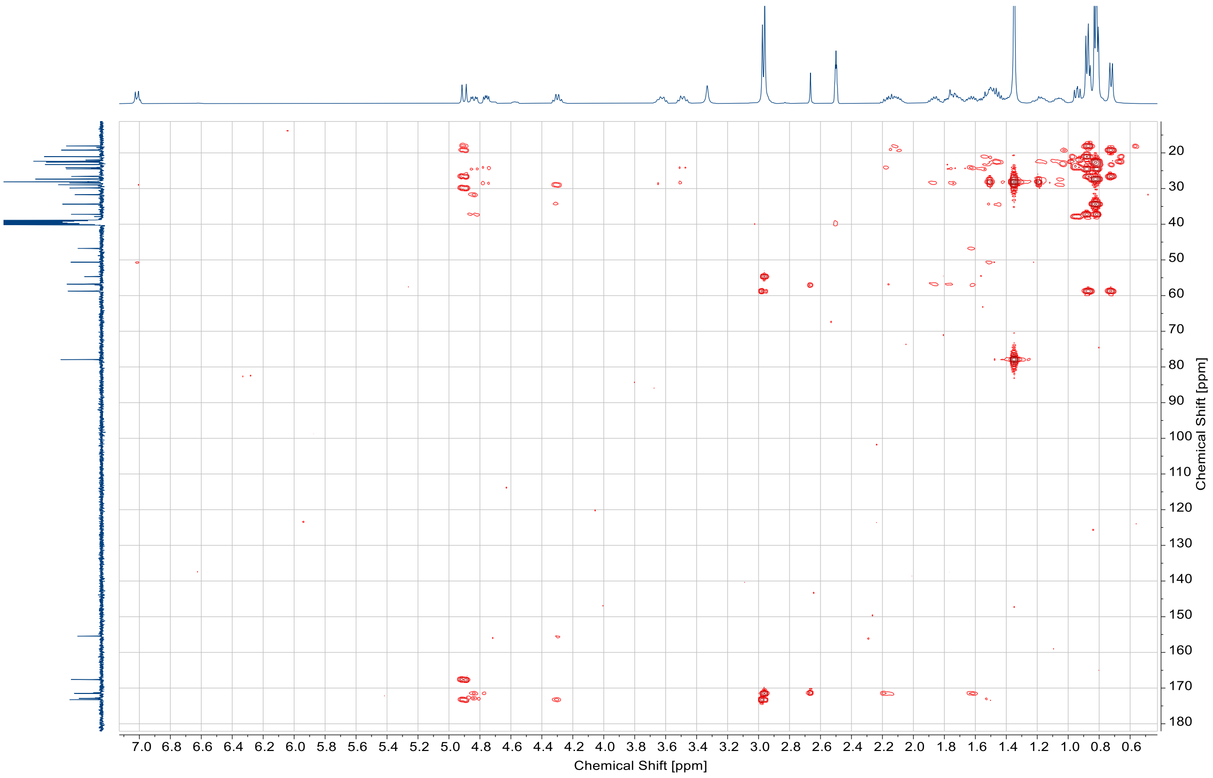

Alloc-*N*-Me-L-Val-L-EtPro-*O*-(Gly-*N*-Me-D-Leu-L-Pro-*N*-Me-L-Val-L-HoLeu-Boc)-*N*-Me-L-Thr-L-Leu-L-MePro-O*t*-Bu [23]

^{1}H-NMR (500 MHz, $CDCl_3$):

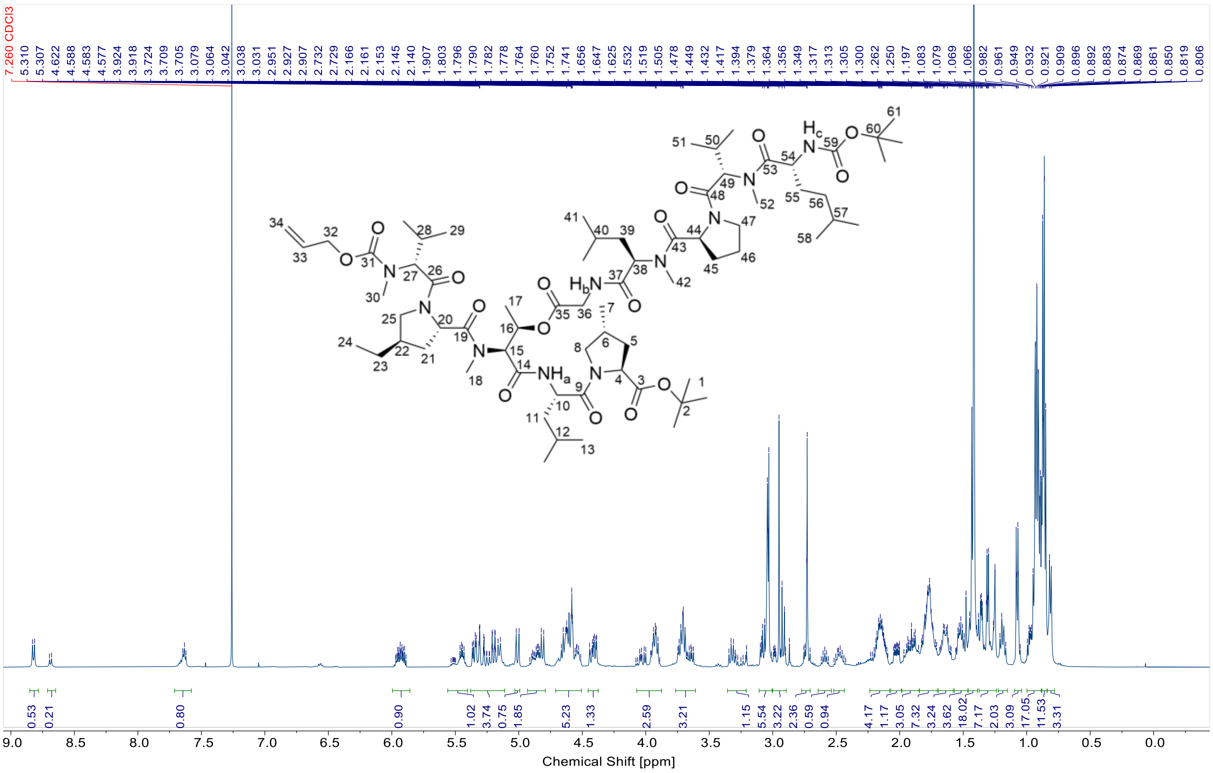

^{13}C-NMR (126 MHz, $CDCl_3$):

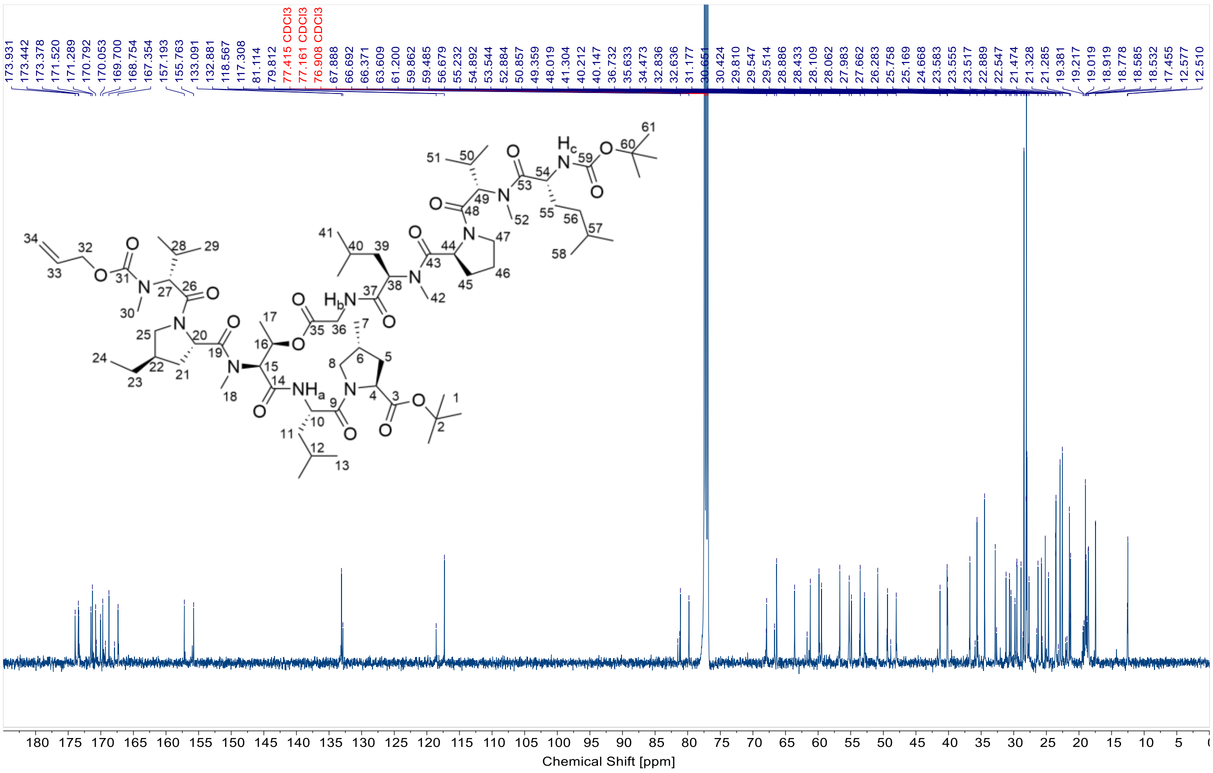

(^{1}H,^{1}H)-COSY ($CDCl_3$):

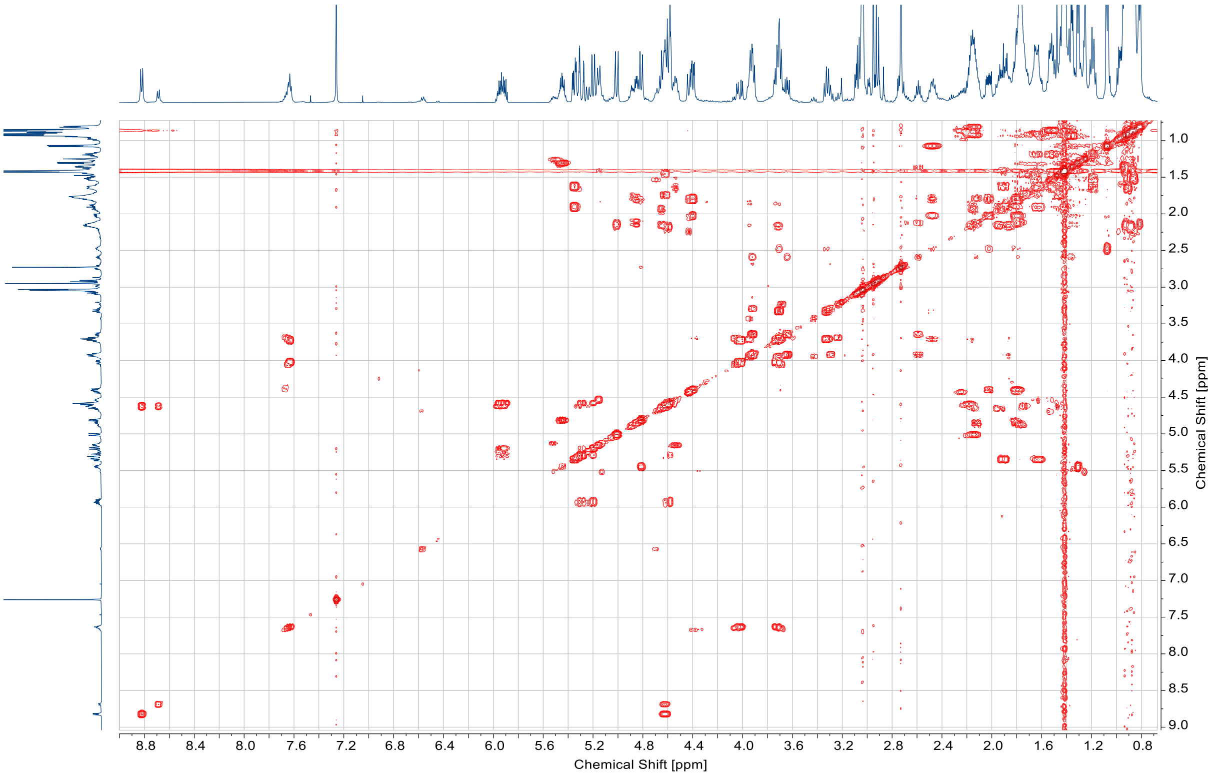

(^{1}H,^{13}C)-HSQC ($CDCl_3$):

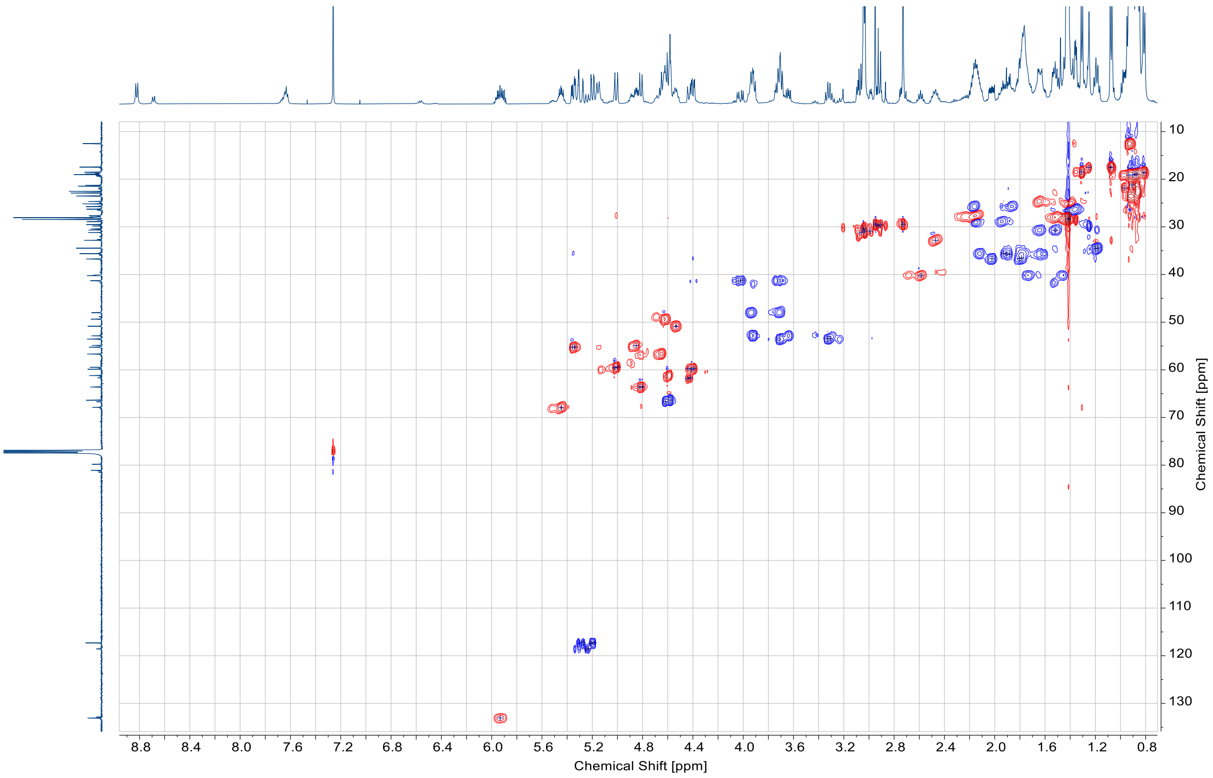

(^{1}H,^{13}C)-HMBC ($CDCl_3$):

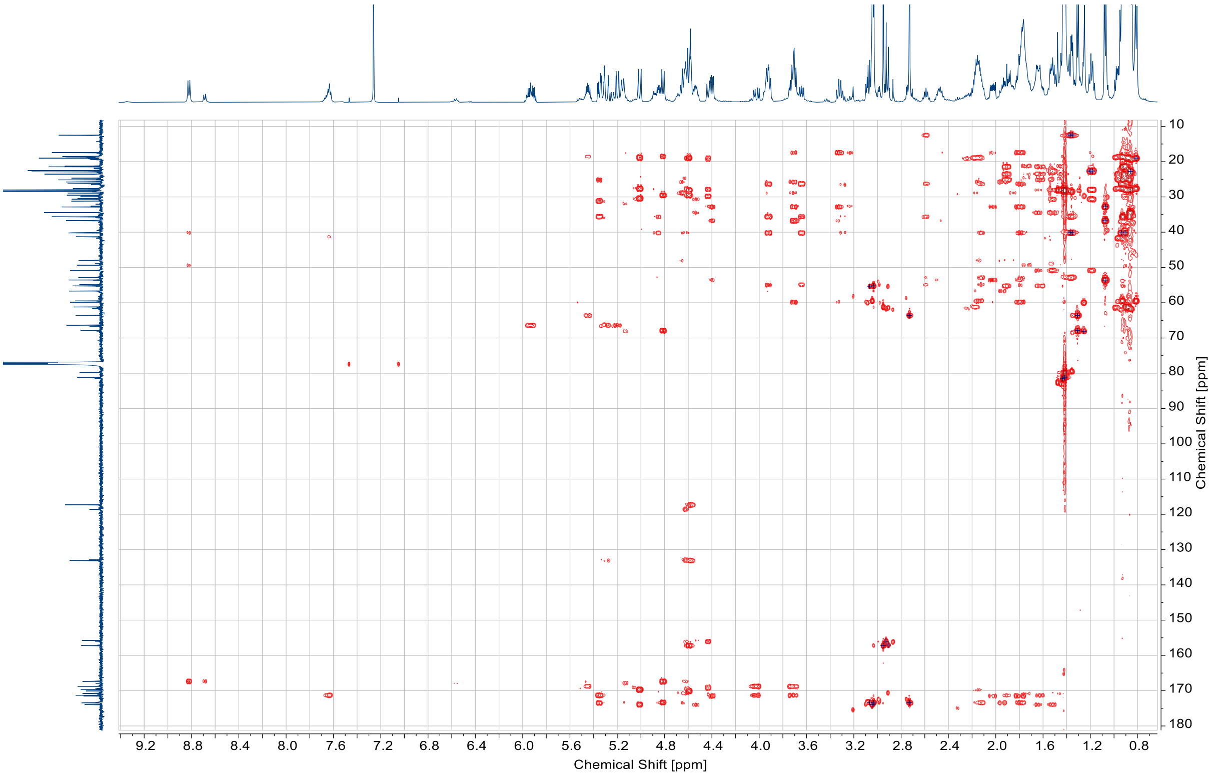

Alloc-*N*-Me-L-Val-L-EtPro-*N*-Me-*cyclo*-*O*-(Gly-*N*-Me-D-Leu-L-Pro-*N*-Me-L-Val-L-HoLeu-L-MePro-L-Leu)-L-Thr [24]

^{1}H-NMR (500 MHz, $CDCl_3$):

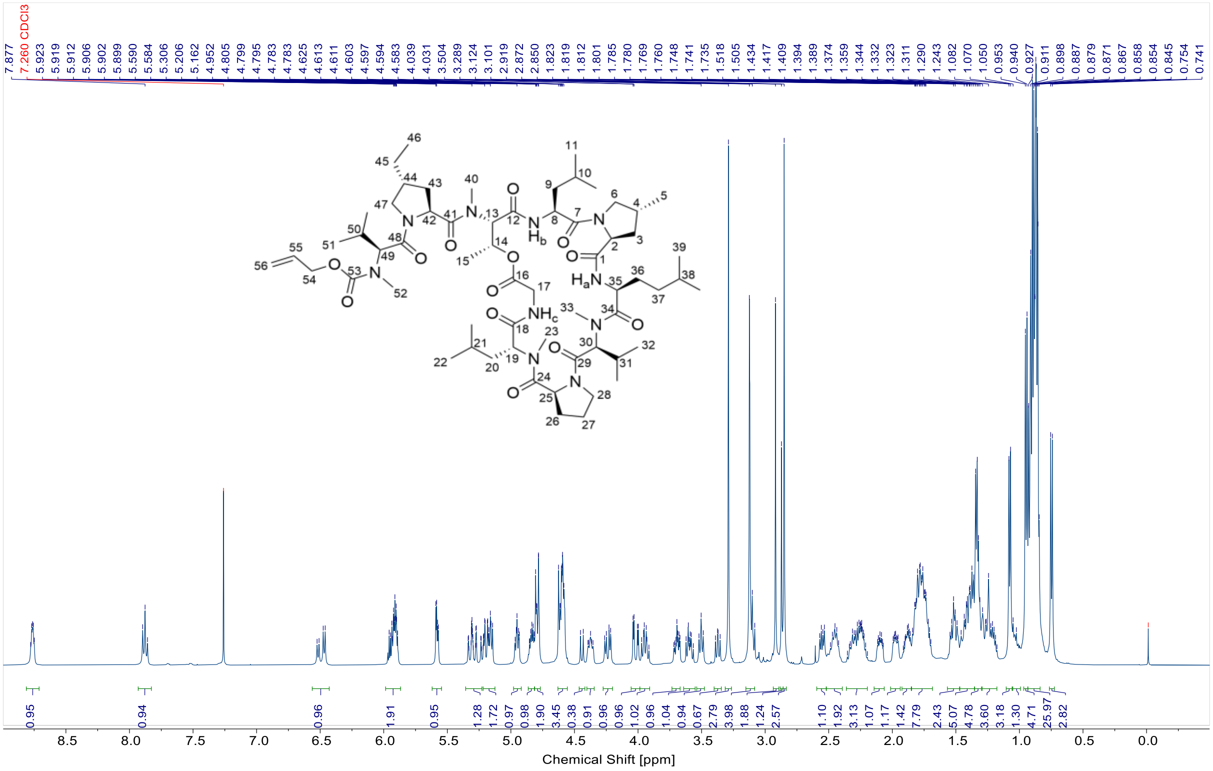

^{13}C-NMR (126 MHz, $CDCl_3$):

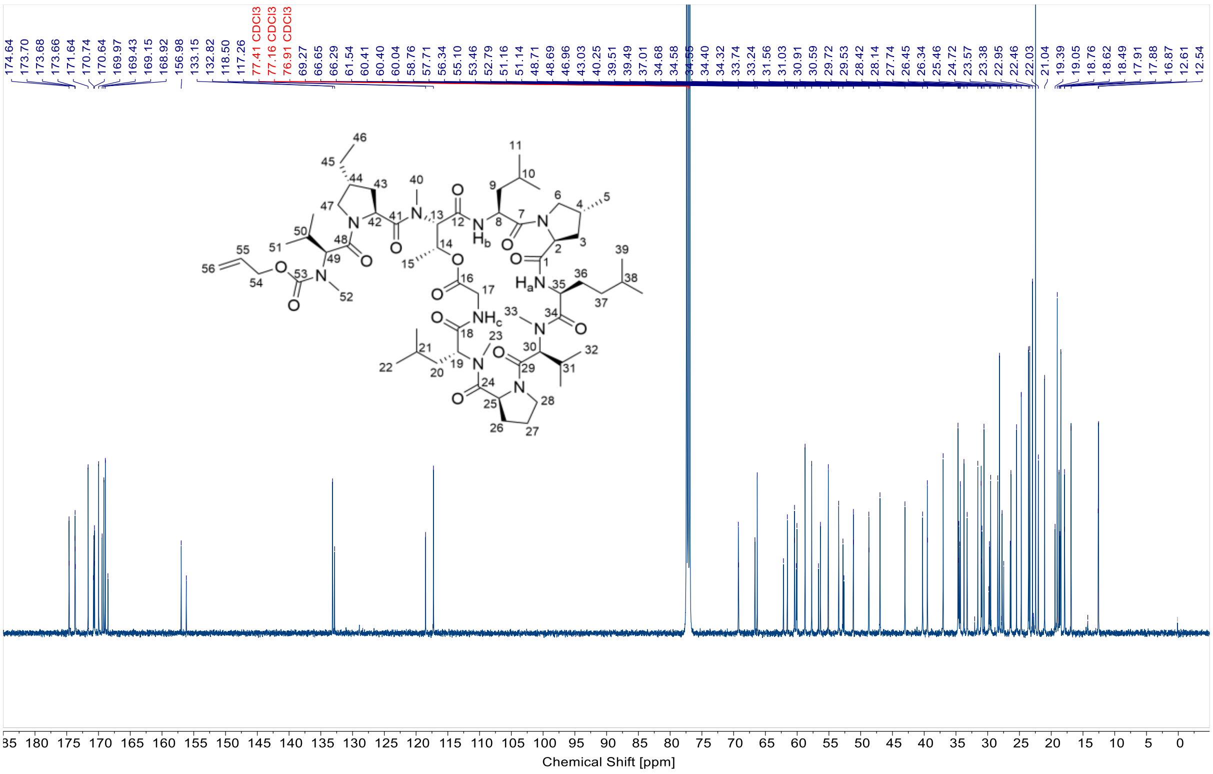

(^{1}H,^{1}H)-COSY ($CDCl_3$):

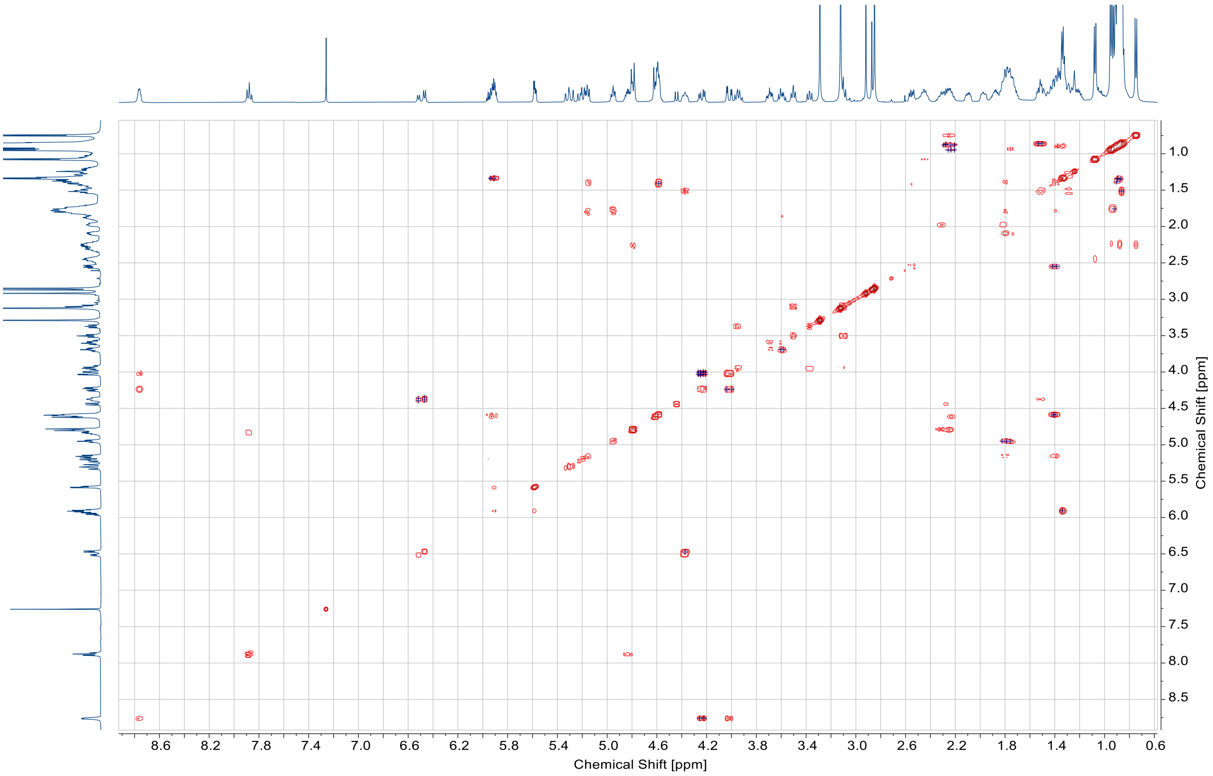

(^{1}H,^{13}C)-HSQC ($CDCl_3$):

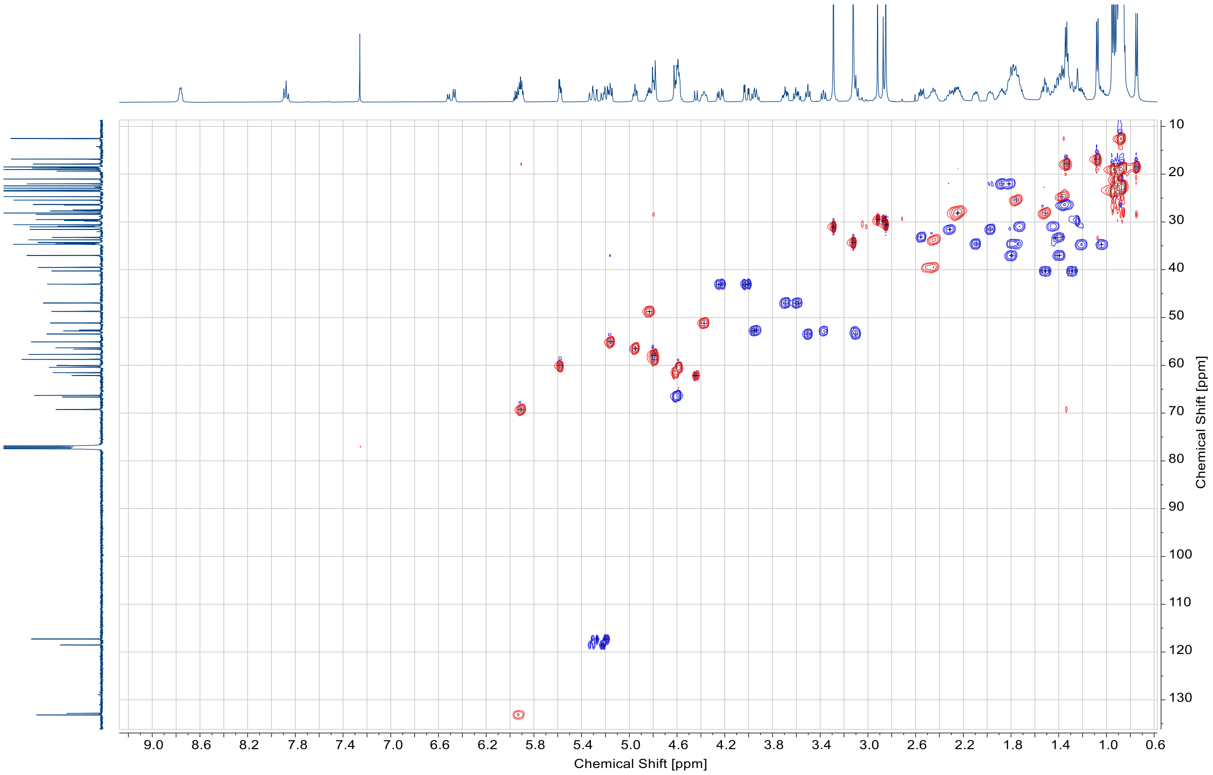

(^{1}H,^{13}C)-HMBC ($CDCl_3$):

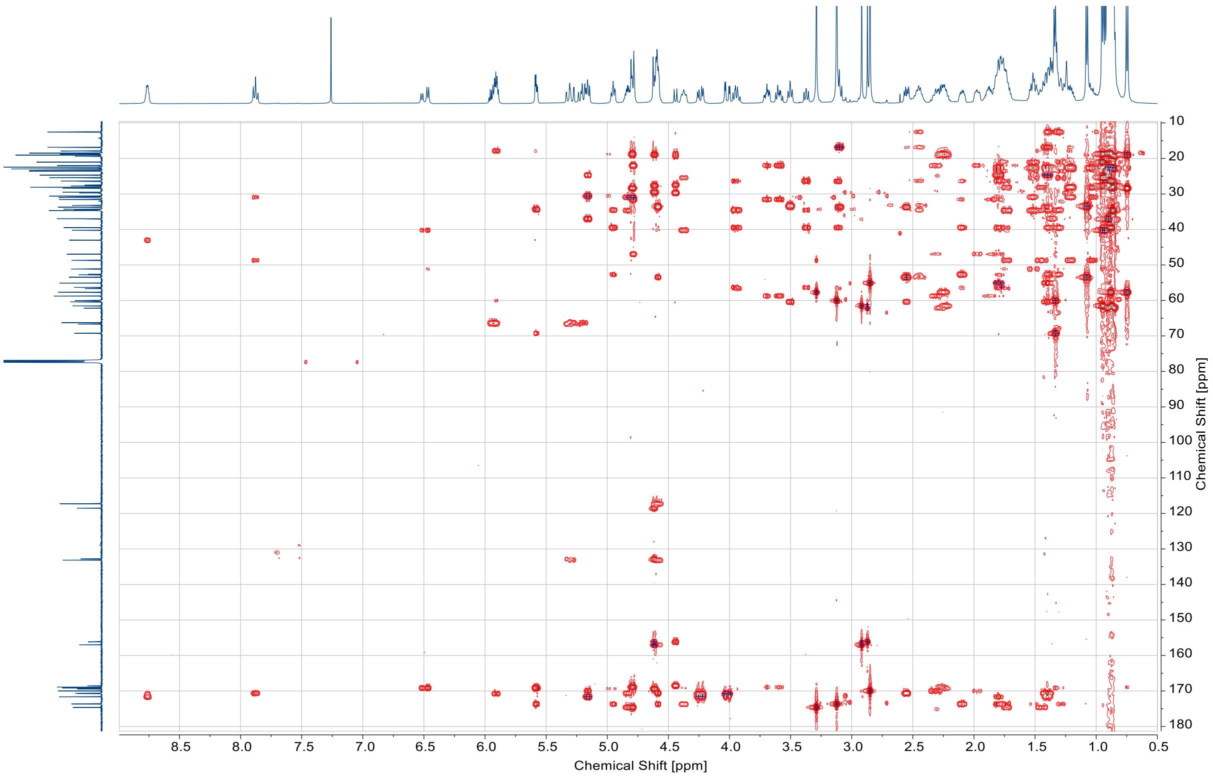

Mycoplanecin A [M45]

^{1}H-NMR (500 MHz, $CDCl_3$):

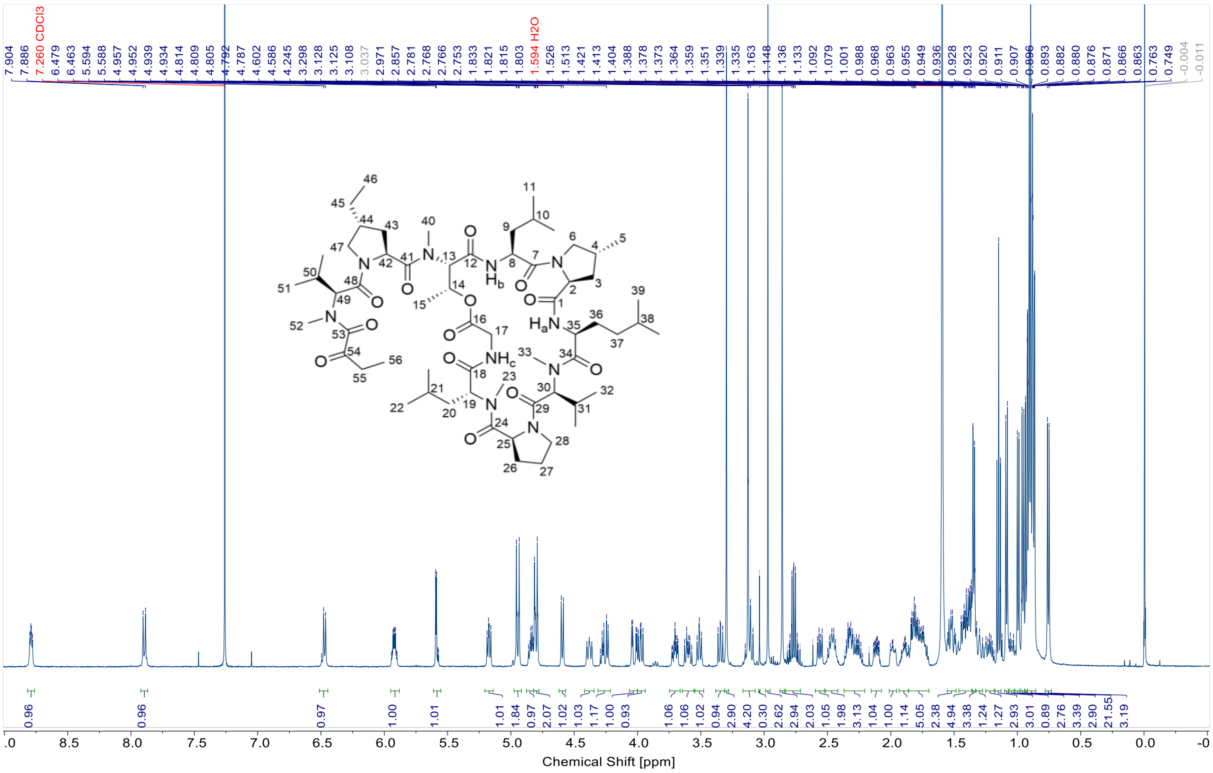

^{13}C-NMR (126 MHz, $CDCl_3$):

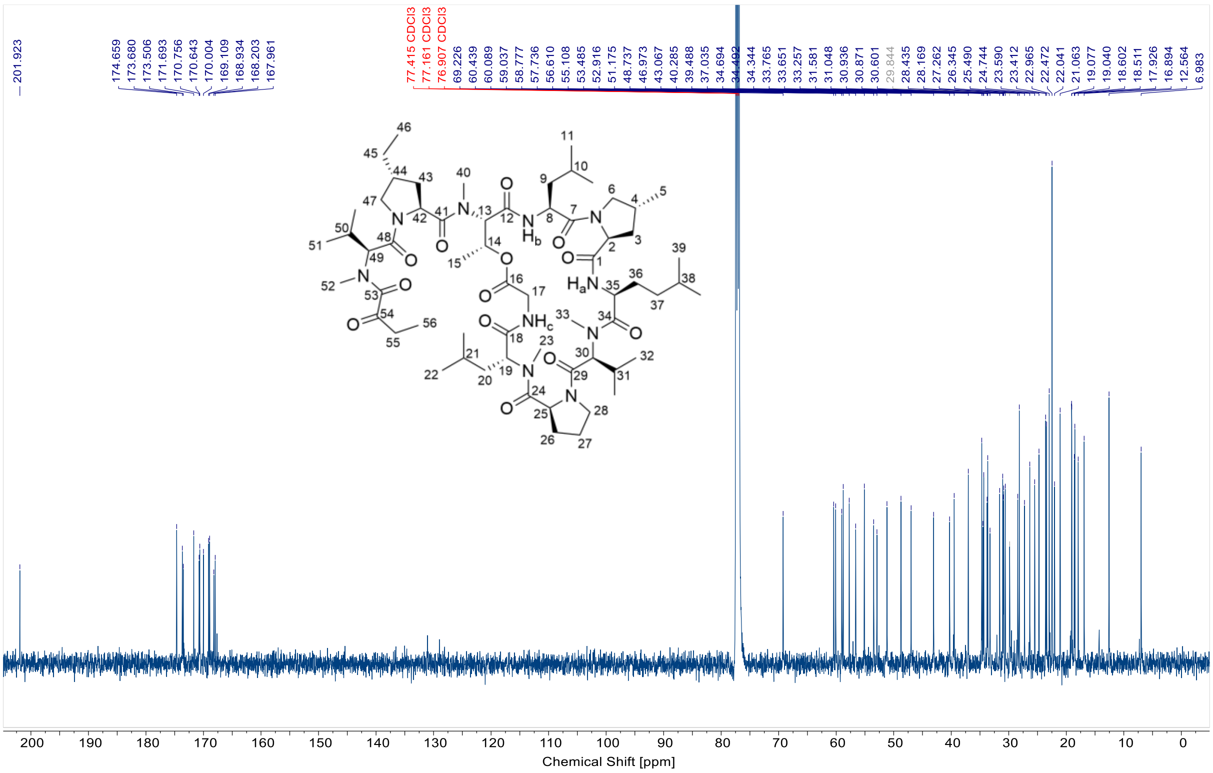

(^{1}H,^{1}H)-COSY ($CDCl_3$):

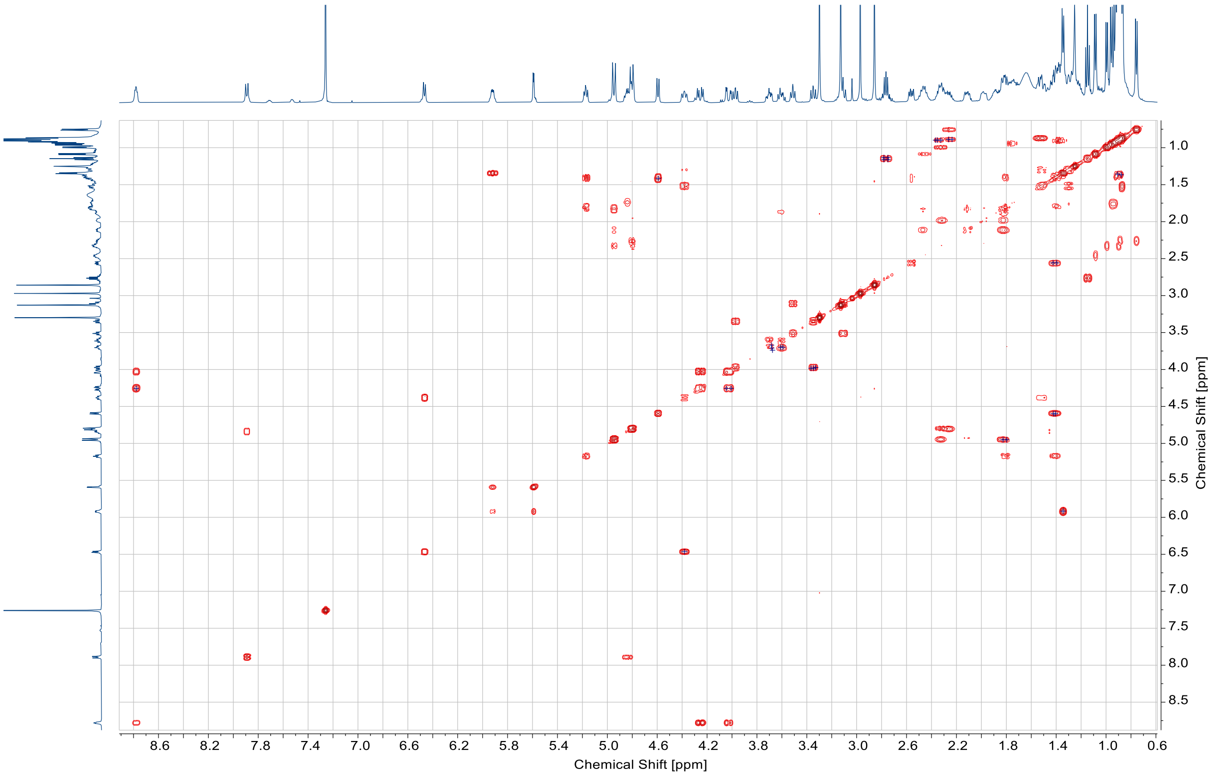

(^{1}H,^{13}C)-HSQC ($CDCl_3$):

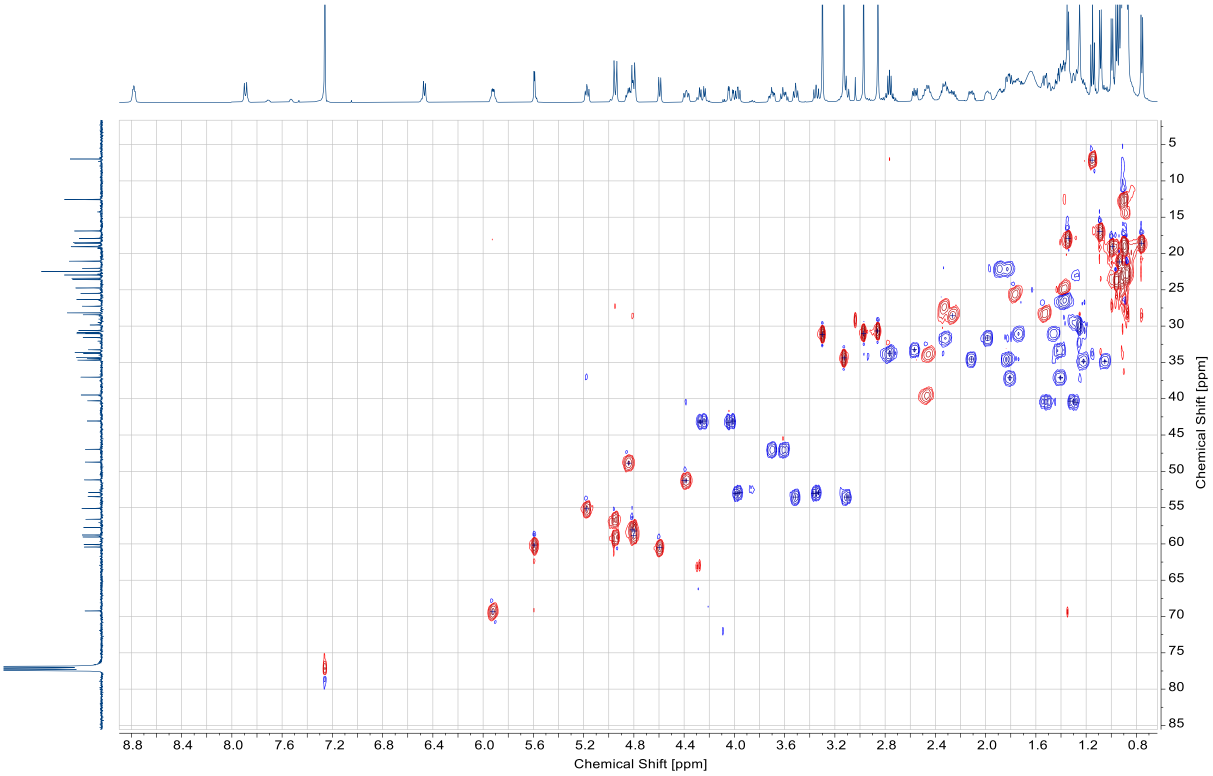

(^{1}H,^{13}C)-HMBC ($CDCl_3$):

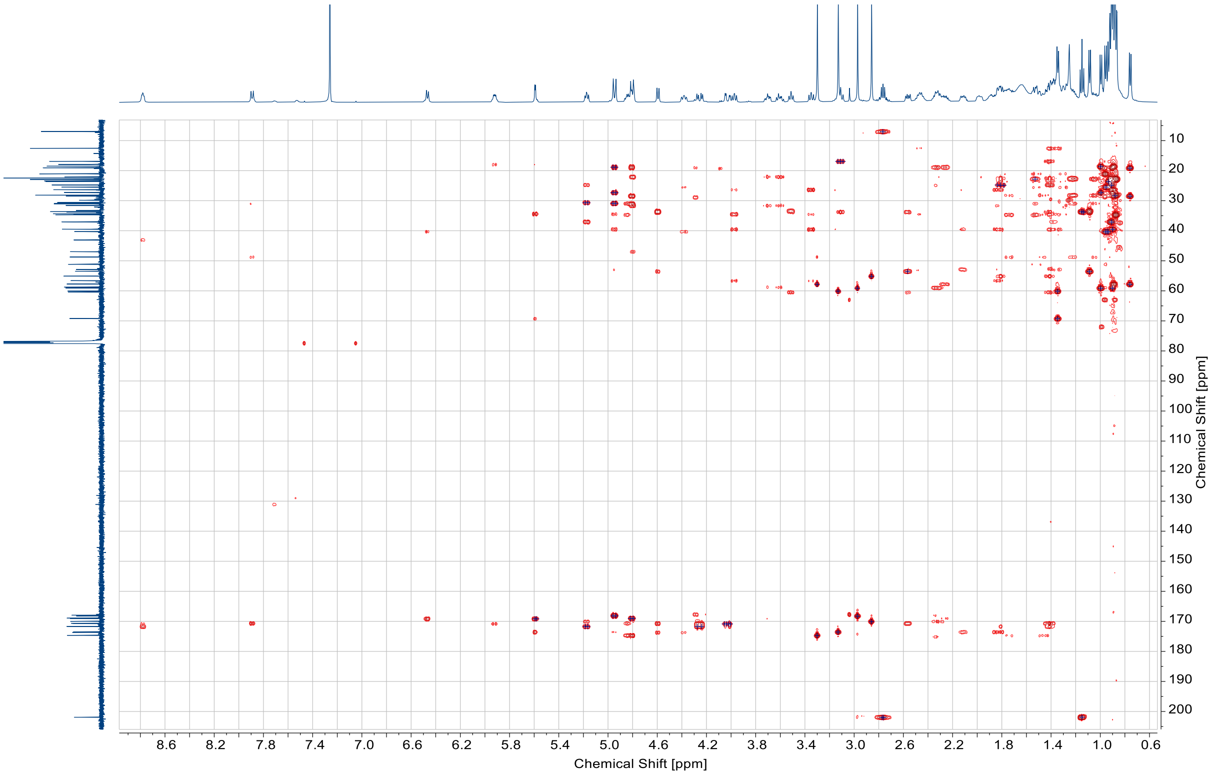

Mycoplanecin A NMR comparison between authentic and synthetic sample

^{1}H-NMR (500 MHz, $CDCl_3$):

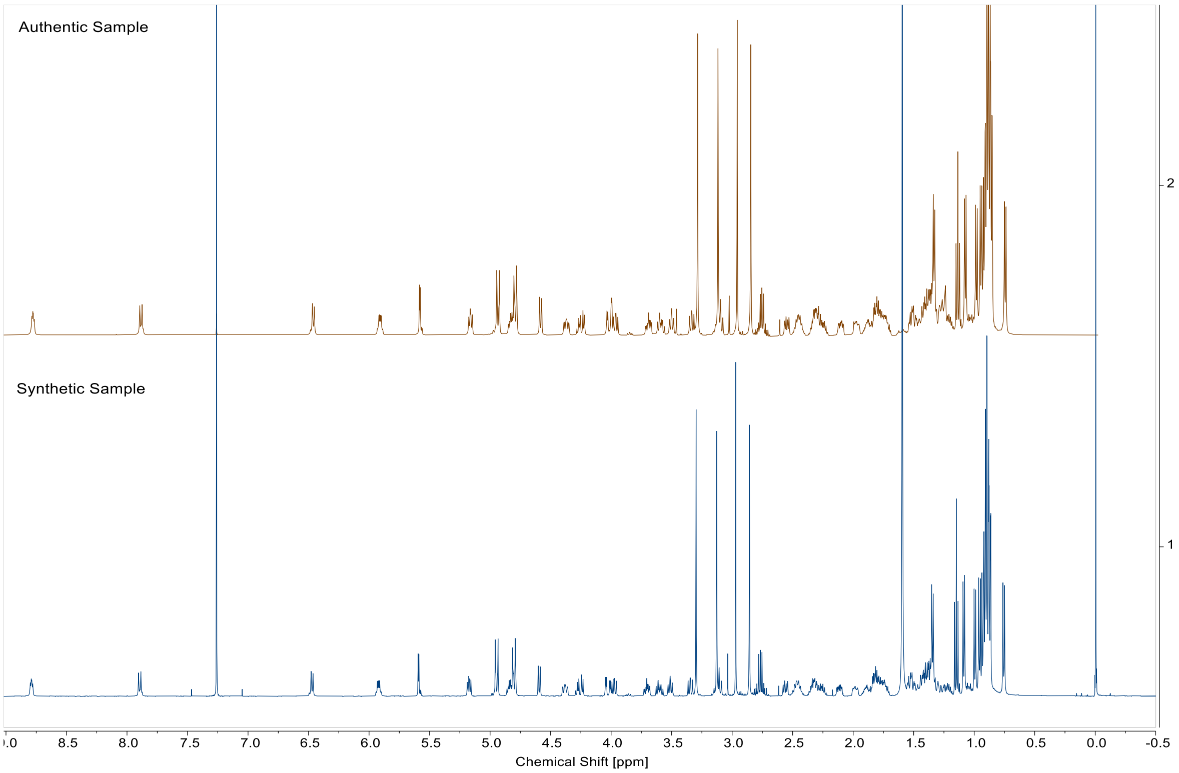

^{13}C-NMR (126 MHz, $CDCl_3$):

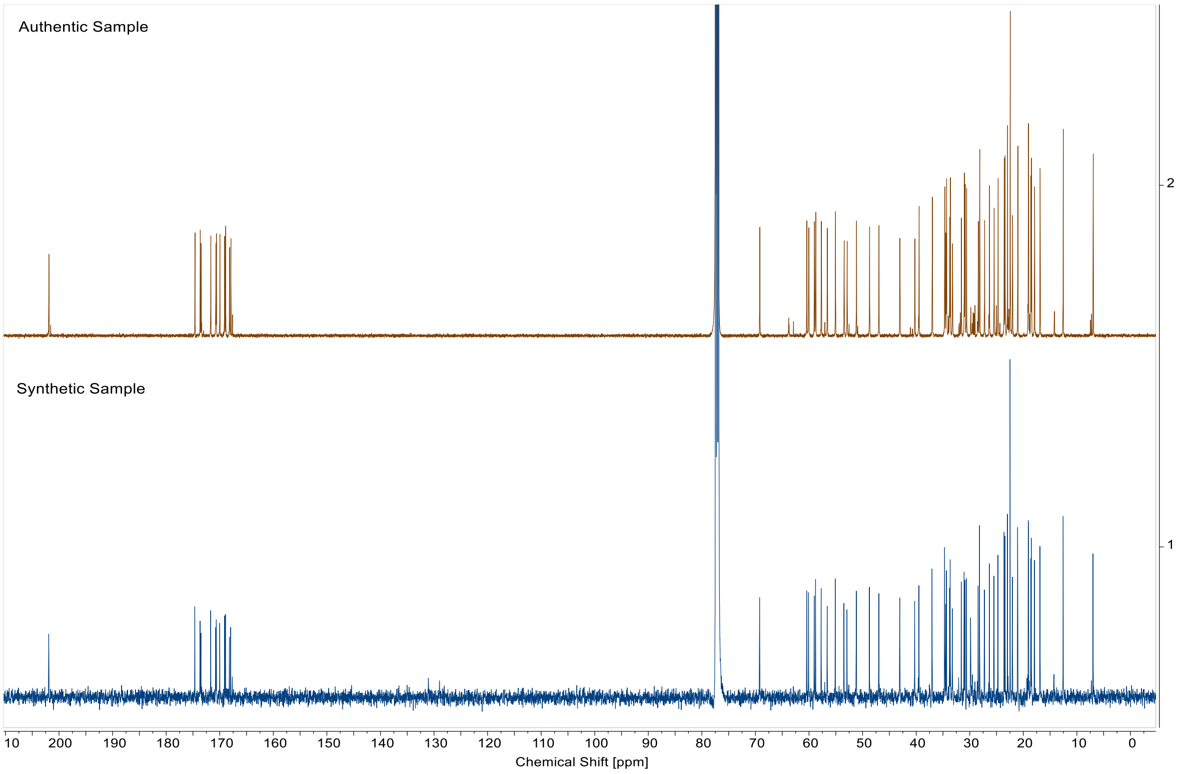

Bibliography

[1] Z. M. Wang, K. B. Sharpless, *J. Org. Chem.* **1994**, *59*, 8302–8303.

[2] W. C. Hiscox, D. S. Matteson, *J. Org. Chem.* **1996**, *61*, 8315–8316.

[3] U. Kazmaier, A. Horn, E. Papadopoulos, T. Kinsinger, J. Greve, E. Bickel, S. Pachoula, *Z. Für Anorg. Allg. Chem.* **2024**, e202400113.

[4] O. C. Ho, R. Soundararajan, J. Lu, D. S. Matteson, Z. Wang, X. Chen, M. Wei, R. D. Willett, *Organometallics* **1995**, *14*, 2855–2860.

[5] A. Horn, U. Kazmaier, *Org. Lett.* **2022**, *24*, 7072–7076.

[6] F. Brackmann, H. Schill, A. De Meijere, *Chem. - Eur. J.* **2005**, *11*, 6593–6600.

[7] G. Franck, K. Brödner, G. Helmchen, *Org. Lett.* **2010**, *12*, 3886–3889.

[8] S. T. Cheung, N. L. Benoiton, *Can. J. Chem.* **1977**, *55*, 906–910.

[9] R. M. Wenger, *Helv Chim Acta* **1983**, *66*, 2672–2702.

[10] N. Kurokawa, Y. Ohfune, *Tetrahedron* **1993**, *49*, 6195–6222.

[11] N. Kurokawa, Y. Ohfune, *J. Am. Chem. Soc.* **1986**, *108*, 6043–6045.

[12] F. Gille, A. Kirschning, *Beilstein J. Org. Chem.* **2016**, *12*, 564–570.

[13] P. M. E. Hawkins, W. Tran, G. Nagalingam, C. Y. Cheung, A. M. Giltrap, G. M. Cook, W. J. Britton, R. J. Payne, *Chem. - Eur. J.* **2020**, *26*, 15200–15205.

[14] Y. Otake, Y. Shibata, Y. Hayashi, S. Kawauchi, H. Nakamura, S. Fuse, *Angew. Chem.* **2020**, *132*, 13025–13030.

[15] A. T. Khan, E. Mondal, *Synlett* **2003**, 694–698.

[16] M. Nakajima, A. Torikata, Y. Ichikawa, T. Katayama, A. Shiraishi, T. Haneishi, A. Mamoru, *J. Antibiot. (Tokyo)* **1983**, *36*, 961–966.
